# Synthesis and Rearrangement of New 1,3-Diamino-2,7-naphthyridines and 1-Amino-3-oxo-2,7-naphthyridines

**DOI:** 10.3390/ijms252211977

**Published:** 2024-11-07

**Authors:** Samvel N. Sirakanyan, Domenico Spinelli, Edoardo Jun Mattioli, Matteo Calvaresi, Athina Geronikaki, Victor G. Kartsev, Elmira K. Hakobyan, Hasmik A. Yegoryan, Hasmik V. Jughetsyan, Mariam E. Manukyan, Anush A. Hovakimyan

**Affiliations:** 1 Institute of Fine Organic Chemistry of A. L. Mnjoyan, Scientific Technological Center of Organic and Pharmaceutical Chemistry of National Academy of Science of Republic of Armenia, Ave. Azatutyan 26, Yerevan 0014, Armenia; hasmik.yegoryan@mail.ru (H.A.Y.); jughetsyan2002@mail.ru (H.V.J.); mariam.manukyan.2002@internet.ru (M.E.M.); aaa.h.87@mail.ru (A.A.H.); 2 Dipartimento di Chimica G. Ciamician, Alma Mater Studiorum-Università di Bologna, Via F. Selmi 2, 40126 Bologna, Italy; domenico.spinelli@unibo.it (D.S.); edoardojun.mattioli2@unibo.it (E.J.M.); matteo.calvaresi3@unibo.it (M.C.); 3 School of Pharmacy, Aristotle University of Thessaloniki, 54124 Thessaloniki, Greece; geronik@pharm.auth.gr; 4 InterBioScreen Ltd., 119019 Moscow, Russia; vkartsev@ibscreen.chg.ru

**Keywords:** 2,7-naphthyridine derivatives, nucleophilic aromatic substitution (S_N_Ar), rearrangement reactions, Smiles rearrangement, NMR, Schiff bases, ESP charges

## Abstract

Herein we describe the synthesis and rearrangement of 1,3-diamino-2,7-naphthyridines and 1-amino-3-oxo-2,7-naphthyridines. In the case of 1,3-diamino-2,7-naphthyridines, it was found that the rearrangement reaction was influenced by both the substituent at the 7th position of the 2,7-naphthyridine ring and by the nature of the cyclic amine at the 1st position. The influence was mainly steric. The reaction of 1-amino-3-oxo-2,7-naphthyridines with amines was studied for the first time. It was revealed that for these substrates, the rearrangement occurs faster and without any influence of the alkyl and cyclic amine groups. We also observed the nucleophilic addition of the amine to the carbonyl group of the rearranged product with the formation of a Schiff base. The calculation of the ESP charges on these substrates indicates a considerable increase in the positive charge on the cyano group that suffers the nucleophilic attack during the rearrangement process, possibly explaining its increased tendency to react and to have a higher reaction velocity.

## 1. Introduction

**Figure 1:**
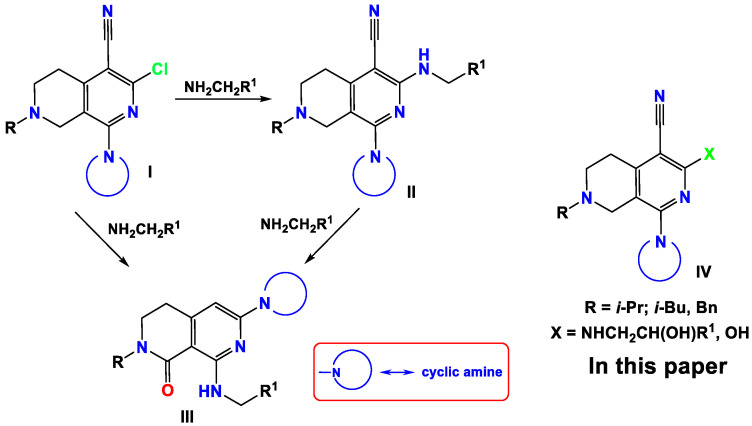
Rearrangement reaction involving 1,3-diamino-2,7-naphthyridines (**II**) and 1-amino-3-oxo-2,7-naphthyridines (**IV**). (**I**–**III**). R = Me, Bn; R^1^ = alkyl; cyclic amine = 1-pyrrolidinyl, 1-piperidinyl, 1-morpholyl, 4-(diphenylmethyl)piperazin-1-yl.

Within our studies on 2,7-naphthyridine derivatives, we discovered a new rearrangement process (Figure 1) [1,2]. The nucleophilic substitution of the chlorine atom in the 1-amino-3-chloro-7-methyl-5,6,7,8-tetrahydro-2,7-naphthyridine-4-carbonitrile (R = Me) compound (**I**) with some primary and secondary cyclic amines [1] (**II**) can trigger an unexpected rearrangement that leads to the formation of 6,8-diamino-2-methyl-2,7-naphthyridin-1-ones (**III**). The following conditions have to be satisfied for the occurrence of this rearrangement:

(a) at C-1 of the 2,7-naphthyridine ring, there must be a cyclic (aliphatic) amine;

(b) the amine at C-3 must be a primary amine (**II**) and should be characterized by a boiling point greater than 145 °C.

The structures of the rearrangement process were confirmed by X-ray crystallography [1].

In a following paper [2], this rearrangement was examined in the case of 1,3-diamino-7-benzyl-5,6,7,8-tetrahydro-2,7-naphthyridine-4-carbonitrile **II** (R = Bn) that has a benzyl group, instead of the methyl group, in the 7th position of the 2,7-naphthyridine ring. This study demonstrated that the replacement of the methyl group with a benzyl group leads to the following consequences: (a) the rearrangement is significantly slower and, in some cases, it does not occur at all; (b) the rearrangement occurs at a higher temperature (*T_bp_* > 160 °C). This fact was explained by the steric difficulties caused by the benzyl group [2].

Nitrogen heterocycles are biologically very important compounds, and there are a large number of papers in which their antioxidant, anti-inflammatory, and antitumor activities were investigated [3,4,5,6]. In particular, derivatives of the 1-oxo-2,7-naphthyridine are physiologically active compounds and may serve as therapeutic agents for addressing inflammatory immune disorders and long-term inflammations [7,8]. This important class of heterocyclic compounds demonstrated considerable biological potential [9,10,11,12,13,14].

However, the methods for the synthetic preparation of 1-oxo-2,7-naphthyridines are limited [15,16,17,18,19]. The presented rearrangement gives the opportunity to develop a new regioselective procedure for the synthesis of 1-oxo-2,7-naphthyridine compounds, with a good yield.

So, the expansion of the scope of the rearrangement has a twofold purpose: (i) to improve the comprehension of its reaction mechanism and (ii) understand the practical value of the new synthetic process.

Here, we determined how the group on the piperidine ring of the 2,7-naphthyridine system can affect the rearrangement, examining the behavior of 7-isopropyl/isobutyl-5,6,7,8-tetrahydro-2,7-naphthyridines **IV** (R = *i*-Pr, *i*-Bu; X = NHCH_2_CH(OH)R^1^, Figure 1). During these studies, azepane was also used for the first time as a cyclic amine.

In addition, we also studied the rearrangement process using 1-amino-3-oxo-2,7-naphthyridines **IV** (R = *i*-Pr, Bn; X = OH, Figure 1), recently synthesized by us.

## 2. Results and Discussions

### 2.1. Synthesis and Rearrangement of 1,3-Diamino-2,7-naphthyridines

**Scheme 1:**
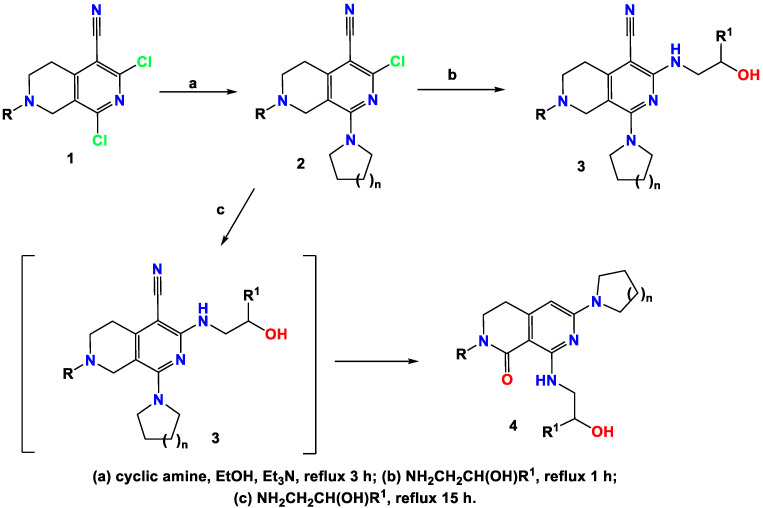
Syntheses and transformations of 1-amino-3-chloro-2,7-naphthyridines **2**. **1**. **a**: R = *i*-Pr; **b**: R = *i*-Bu; **c:** R = Bn; **2**. **a**–**c:** R = *i*-Pr, **a:** n = 1; **b:** n = 2; **c:** n = 3; **d**–**f:** R = *i*-Bu, **d:** n = 1; **e:** n = 2; **f:** n = 3; **g**–**i:** R = Bn, **g:** n = 1; **h:** n = 2; **i:** n = 3.

7-Alkyl-1,3-dichloro-5,6,7,8-tetrahydro-2,7-naphthyridine-4-carbonitriles **1a**–**c** [20] were used as starting compounds. The reaction of these compounds **1a**–**c** with cyclic amines (pyrrolidine, piperidine, and azepane) in absolute ethanol led to the formation of 7-alkyl-1-amino-3-chloro-5,6,7,8-tetrahydro-2,7-naphthyridines **2a**–**i** [21], thereby satisfying the first necessary condition for the rearrangement, that is, the presence at the C-1 of the 2,7-naphthyridine ring of a cyclic amine (Scheme 1).

The relation between electronic charge distribution of a heterocycle and its influence on substitution reactions can be easily established. The electrostatic potential values, which accurately reflect the delocalized nature of electron density, can be used to assess the relative reactivity of heterocycles. The atomic ESP charges in heterocyclic compounds can be correlated with the locations of electrophilic and nucleophilic substitutions [22].

So, when the S_N_Ar reaction is under electronic control, its regioselectivity in multihalogenated compounds is predictable by an ESP analysis [22]. The extent of electron deficiency at the reactive carbon is the key factor in determining S_N_Ar rates, and the corresponding ESP charges are quantitative descriptors of the reactivity of the two sites [22].

We carried out a conformational analysis of the molecules involved in the S_N_Ar reactions, and then we carried out QM calculations on the geometric structure of the minima to obtain the ESP atomic charges on the **1a**–**c** compounds (see Section 3.9). ESP charge calculations revealed a negligible difference between the charges of the two carbon atoms bound to the chlorine atoms (Figure S1), so the different reactivity of the two sites in the 2,7-naphthyridine ring can be explained only by the steric hindrance caused by the cyano group. From the conformational point of view it is in interesting to underline the π-π stacking interaction observed between the 2,7-naphthyridine ring and the benzyl substituent.

In the following step, compounds **2a**–**f** (containing the isopropyl and isobutyl groups) were reacted with the primary amines 2-aminoethanol (*T_bp_* = 170 °C) and 1-aminopropan-2-ol (*T_bp_* = 160 °C) under harsher experimental conditions than the previous step, i.e., by refluxing in the presence of the amine to satisfy the second necessary condition for the rearrangement, that is, the presence of a primary amine with a boiling point greater than 145 °C at C3 (Scheme 1, Table 1).

Considering our previous experience [1,2], the reaction mixture was examined after 1, 5, and 10 h from the start of the reaction. After 1 h, the spectroscopic data showed that only the 1,3-diamino-2,7-naphthyridines **3a**–**l** were formed in high yields (Y = 74–86%) and that no rearrangement occurred (Scheme 1, Table 1). During the examination of the reaction mixture by ^1^H NMR and IR spectroscopy after 5 and 10 h, we observed the mixture of compounds **3** and the corresponding rearrangement of products **4** in different ratios. Therefore, the reaction time was lengthened and the reaction mixture was examined after 15 h. As a result, the corresponding rearrangement products 2,7-naphthyridin-1-ones **4a**–**l** were isolated in moderate yields (Y = 55–67%, Scheme 1, Table 1).

From the results it can be observed that the rearrangement for the derivatives **2a**–**f**, with the isopropyl and isobutyl groups at the 7th position of the 2,7-naphthyridine ring, is more difficult than for compounds **I** with the methyl and benzyl groups at the same position (Figure 1). In fact, for compounds **2a**–**f**, the reaction time necessary for the rearrangement is lengthened from 5 to ≈15 h.

The effect of the alkyl substitution at the 7th position of the 2,7-naphthyridine on the electronic property of the ring is negligible. Considering the ESP charges on the C1 and C3 carbon atoms (Figure S1) that undergo nucleophilic attack by amines, there are no significative variations, so in principle, the different alkylic substitution does not kinetically influence the first two steps of the nucleophilic attack exerted by the amines. On the contrary, taking into account that the rearrangement took place through the cleavage of the C-8/Cpyr bond and the following attack on the cyano group, as reported previously [1,2], the slowdown of the rearrangement due to the presence of the isopropyl and isobutyl groups can be explained by the steric hindrance caused by these groups at the 7th position of the 2,7-naphthyridine system, determining the following velocity of the rearrangement: Me < Bn < *i*-Pr ≈ *i*-Bu. These results are in line with the Taft’s steric parameter Es (Es (Me) = 0; Es (Bn) = −0.38; Es (*i*-Pr) = −0.47; and Es (*i*-Bu) = 0.93).

An influence of the first-position cyclic amine was also observed on the rearrangement; that is, in the case of bulkier amines, the rearrangement reaction is more difficult. Again, the presence of pyrrolidine, piperidine, and azepane does not have an influence on the velocity of the second nucleophilic attacks exerted by the amine (the value of the positive charge on the C3 carbon is the same, Figure S2), but the presence does influence the rearrangement step. This fact can be also explained by the steric hindrances caused by the cyclic amine and determines the following order for the rearrangement step: pyrrolidine < piperidine ≈ azepane, that is proportional to the molar volume of the substituent V (pyrrolidine) = 65.2 cm^3^ mol^−1^; V (piperidine) = 95.7 cm^3^ mol^−1^; and V (azepane) = 94.3 cm^3^ mol^−1^.

The structures of the obtained compounds **3** and **4** were confirmed by IR, NMR, MS spectroscopy, and elemental analysis (see Supplementary Material). The IR spectra of compounds **3a**–**l** always showed strong absorption bands of the cyano group at 2187–2207 cm^−1^, which is the main proof that the rearrangement did not occur. By examining the ^1^H NMR spectra of these compounds, it was found that the singlet signal of the NCH_2_ group at the 8th position of the 2,7-naphthyridine ring at 3.20–3.45 ppm, as well as the signal of the NH group of the amine fragment at 5.78–5.95 ppm was present, which is typical for compounds **3**.

As expected, in the ^1^H NMR spectra of compounds **4a**–**l,** the singlet signal of one proton of the pyridine ring at 5.34–5.60 ppm was present, while the singlet signal of the NCH_2_ group at C-8 was absent. Also, the same signal of the NH group of the amine fragment was shifted to a weaker field at 8.96–9.14 ppm. This shift may be due to the hydrogen bond arising from the interaction between the proton of the NH group and the carbonylic oxygen [1]. The infrared spectra of products **4a**–**l** demonstrated the typical absorption bands at 1600–1607 cm^−1^ characteristic for the carbonyl group, and obviously the absorption bands of the cyano substituent are missing. The structure of compounds **4a**–**l** was also supported by ^13^C NMR spectra and MS data, which are in agreement with the proposed structures.

### 2.2. Synthesis and Rearrangement of 1-Amino-3-oxo-2,7-naphthyridines

**Scheme 2:**
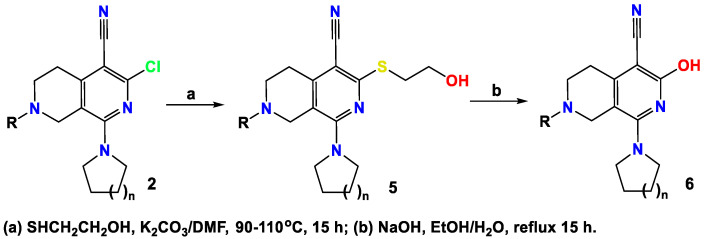
Preparation of 1-amino-3-oxo-2,7-naphthyridines **6a**–**f**. **5, 6**. **a**–**c:** R = *i*-Pr; **a:** n = 1; **b:** n = 2; **c:** n = 3; **d**–**f:** R = Bn; **d:** n = 1; **e:** n = 2; **f:** n = 3.

It is very interesting to investigate the proposed rearrangement in 1-amino-3-oxo-2,3,5,6,7,8-hexahydro-2,7-naphthyridine-4-carbonitriles **6a**–**f**. Compounds **6a**,**c** were recently synthesized by us via a Smiles rearrangement [23,24,25] as synthons for the preparation of novel heterocyclic systems: furo [2,3-*c*]-2,7-naphthyridines [23]. 1-Amino-3-chloro-2,7-naphthyridines **2a**–**f** reacted with 2-mercaptoethanol to give the corresponding 1-amino-3-[(2-hydroxyethyl)thio]-5,6,7,8-tetrahydro-2,7-naphthyridine- 4-carbonitriles **5a**–**f** [23]. Compounds **5** undergo a Smiles rearrangement [23,24,25] resulting in 1-amino-3-oxo-2,7-naphthyridines **6a**–**f,** in high yields (Scheme 2). The structures of the obtained compounds **6** were confirmed by spectroscopic and by elemental analyses.

**Scheme 3:**
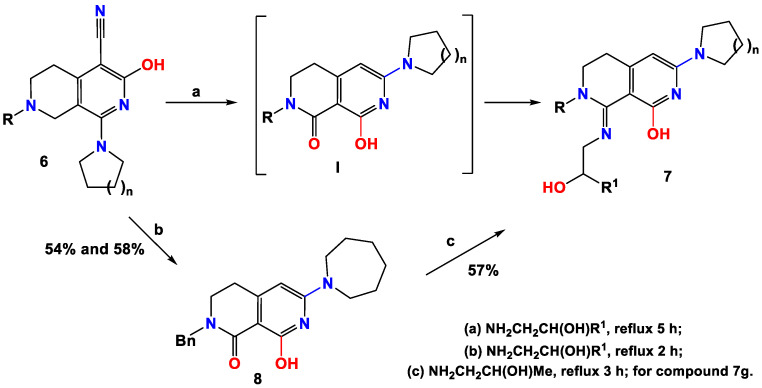
Rearrangement of 1-amino-3-oxo-2,7-naphthyridines **6**.

We examined the possibility of a further rearrangement of compounds **6a**–**f**, using the experimental conditions described in the previous paragraph. Thus, compounds **6a**–**f** were reacted with 2-aminoethanol and 1-aminopropan-2-ol without any solvent and the reaction mixture was examined after 5 h. The spectroscopic data showed that a rearrangement occurs and that the formed carbonyl group undergoes a nucleophilic addition forming amines (Schiff bases) **7a**–**g** in moderate yields (Y = 59–68%, Scheme 3, Table 2).

Only in one case did we succeed in isolating and identifying the rearrangement intermediate product **I** (compound **8**). Thus, the reaction time was reduced to 2 h and during the reaction of compound **6f** with 2-aminoethanol or 1-aminopropan-2-ol the 6-azepan-1-yl-2-benzyl-8-hydroxy-3,4-dihydro-2,7-naphthyridin-1(2*H*)-one, (**8**) was isolated in 54% and 58% yields (Scheme 3, Table 2). When compound **8** is refluxed with 1-aminopropan-2-ol for 3 h, as expected, compound **7g** was formed in a 57% yield, which once again proves that the reaction proceeds through intermediate **I** (Scheme 3).

It should be noted that in the case of 1-amino-3-oxo-2,7-naphthyridines **6a**–**f**, the reaction is so fast that the influence of the alkyl (isopropyl or benzyl) and cyclic amine (pyrrolidinyl, piperidinyl, and azepane) groups in the rearrangement reaction is not observed. The rearrangement reaction in the 1-amino-3-oxo-2,7-naphthyridine series is greatly facilitated and occurs significantly faster than in the case of 1,3-diamino-2,7-naphthyridines **3**.

To identify the most stable conformation of compounds **6a**–**f**, the energy profile for the rotation around the NC-OH bond in 1-amino-3-oxo-2,7-naphthyridines was calculated (Figure S3).

When the ESP charges are calculated for these minima (Figure S4), it is clear that the charge on the carbon atom of the cyano group, which suffers the nucleophilic attack during the rearrangement process, is considerable higher (+0.53/+0.61) in the case of the OH substituent, possibly explaining its increased tendency to react and have a higher velocity of reaction.

The structures of compounds **7a**–**g** and **8** were confirmed by IR, NMR, MS spectroscopy, and elemental analysis. The IR spectra of compounds **7a**–**g** obviously do not show the typical absorption bands of the carbonyl and cyano groups, while display bands at 1567–1579 cm^–1^, which are due to C=N of the azomethine group of the Schiff base [15]. In contrast, the IR spectrum of compound **8** clearly shows the carbonyl group absorption at 1625 cm^−1^, and, at the same time, does not show the absorptions of the C≡N and C=N groups. The simulated IR spectra of **4**, **7,** and **8** (Figure S5) confirm the experimental observations.

**Figure 2:**
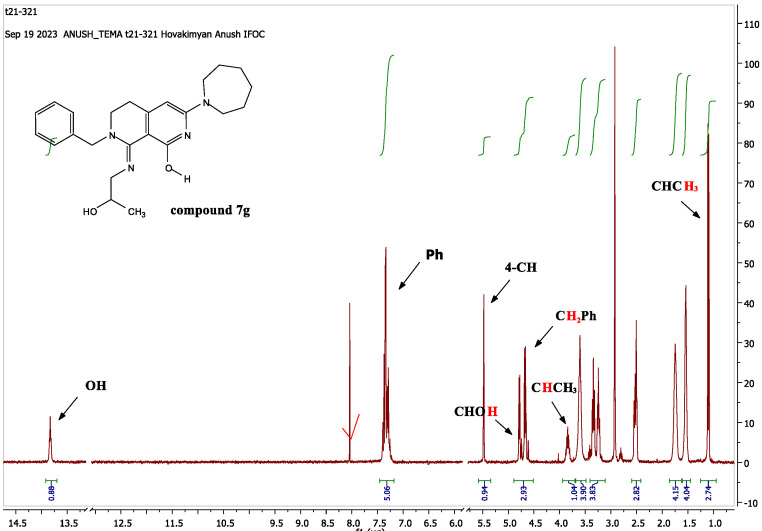
The ^1^H NMR spectrum of compound **7g**.

**Figure 3:**
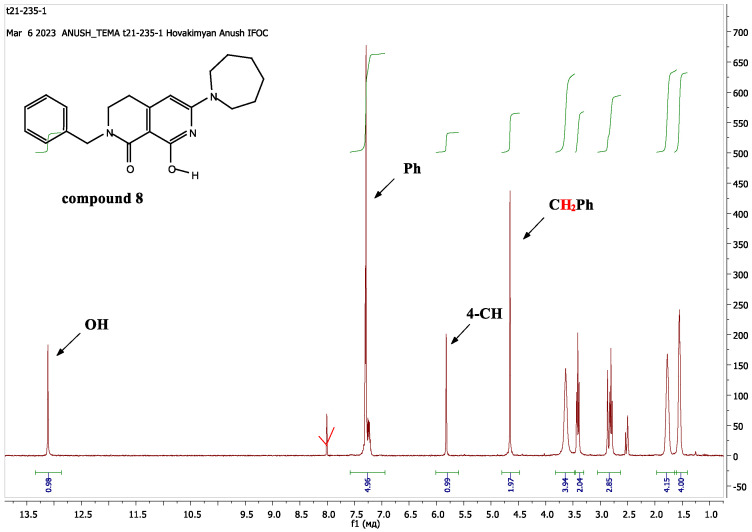
The ^1^H NMR spectrum of compound **8**.

In the ^1^H NMR spectra of compounds **7a**–**g,** the presence of the reacting amine fragment was demonstrated by the detection of protons from the NCH_2_CH(OH)R^1^ group of the singlet signal of the proton of the 4-CH group at 5.35–5.61 ppm and of the broad proton signal of the OH group at 13.13–13.83 ppm. From the spectrum of compound **8,** it is clear that the singlet signal of the CH group of the pyridine ring appears at 5.82 ppm and the singlet signal of the OH group appears at 13.11 ppm, which is typical of the rearranged compounds. For clarity, Figure 2 and Figure 3 show the ^1^H NMR spectra of compounds **7g** and **8**.

**Figure 4:**
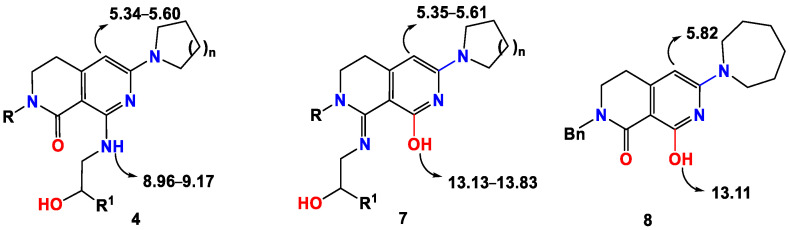
Some ^1^H NMR spectral data of rearrangement products **4**, **7,** and **8**.

In Figure 4, some ^1^H NMR spectral data of the rearrangement products **4**, **7,** and **8** are displayed. As can be seen, in the rearrangement products, the singlet signal of one proton of the pyridine ring almost does not change. Also, the singlet signal of the OH group in compounds **7** and **8** appeared in the similar field. The ^13^C NMR and MS spectra also agree with the structure of compounds **4**, **7,** and **8**.

**Scheme 4:**
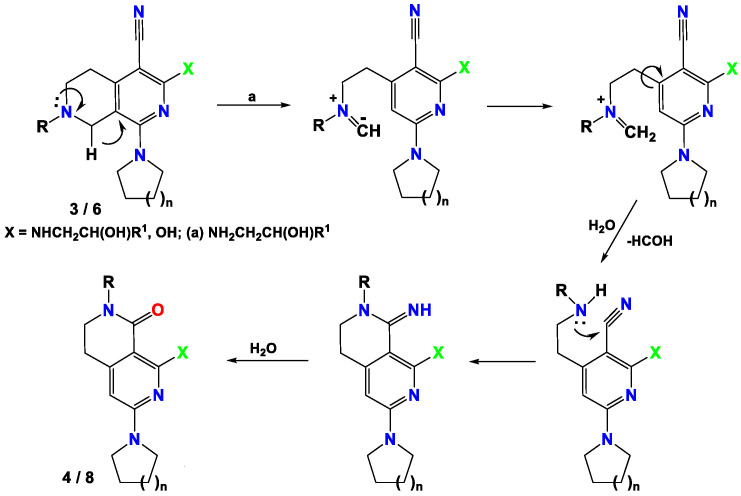
Mechanism proposed for the synthesis of **4/8** from compounds **3/6**.

The tentative pathway of the rearrangement mechanism is previously reported [1] and can be represented as follows (Scheme 4).

## 3. Materials and Methods

^1^H and ^13^C NMR spectra were recorded on a Varian Mercury-300VX spectrometer (Varian Inc., Palo Alto, CA, USA) at 300 MHz for ^1^H and 75 MHz for ^13^C, respectively. The IR spectra were recorded on a Nicolet Avatar 330-FT-IR spectrophotometer (Thermo Nicolet, Madison, CA, USA). High-resolution mass spectra (HRMS) were obtained on a Waters Q-Tof (Waters, Manchester, UK). Melting points were determined on an MP450 melting point apparatus. Elemental analyses were performed on an Elemental Analyzer Euro EA 3000. All reagents were purchased from commercial sources and used without further purification. Compounds **2a,c,g** [23], **5a,c,** and **6a**,**c** [23] are already described.

### 3.1. General Procedure for the Synthesis of Compounds **2b,d**–**f,h,i**

A mixture of compound **1** (10 mmol) of corresponding amine (11 mmol) and of triethylamine (1.53 mL, 11 mmol) in absolute ethanol (50 mL) was refluxed for 3 h. After cooling, water (50 mL) was added and the resulting crystals were filtered off, washed with water, dried, and recrystallized from ethanol.

3-Chloro-7-isopropyl-1-piperidin-1-yl-5,6,7,8-tetrahydro-2,7-naphthyridine-4-carbonitrile (**2b**). Light yellow solid; yield 91%, mp 125–126 °C; IR *ν*/cm^–1^: 2215 (C≡N). ^1^H NMR (300 MHz, DMSO-*d*_6_/CCl_4_, 1/3) δ 1.10 (d, *J =* 6.5 Hz, 6H, CH(CH_3_)_2_), 1.66–1.71 (m, 6H, C_5_H_10_N), 2.73–2.82 (m, 2H, NCH_2_CH_2_), 2.83–2.95 (m, 3H, NCH_2_CH_2_, CH(CH_3_)_2_), 3.27–3.33 (m, 4H, N(CH_2_)_2_), 3.39 (s, 2H, NCH_2_). ^13^C NMR (75 MHz, DMSO-*d*_6_/CCl_4_, 1/3) δ 18.02, 23.78, 25.31, 28.93, 44.24, 48.39, 49.55, 53.21, 99.96, 114.06, 119.87, 147.87, 150.38, 159.94. Anal. calcd. for C_17_H_23_ClN_4_: C 64.04; H 7.27; N 17.57%. Found: C 64.42; H 7.44; N 17.78%.

3-Chloro-7-isobutyl-1-pyrrolidin-1-yl-5,6,7,8-tetrahydro-2,7-naphthyridine-4-carbonitrile (**2d**). Colorless solid; yield 88%, mp 168–170 °C; IR *ν*/cm^–1^: 2219 (C≡N). ^1^H NMR (300 MHz, DMSO-*d*_6_/CCl_4_, 1/3) δ 0.90 (d, *J =* 6.6 Hz, 6H, CH(CH_3_)_2_), 1.76–1.89 (m, 1H, CH(CH_3_)_2_), 1.90–2.00 (m, 4H, C_4_H_8_N), 2.26 (d, *J =* 7.3 Hz, 2H, CHCH_2_), 2.66 (t, *J =* 5.7 Hz, 2H, NCH_2_CH_2_), 2.86 (t, *J =* 5.8 Hz, 2H, NCH_2_CH_2_), 3.51 (s, 2H, NCH_2_), 3.58–3.65 (m, 4H, N(CH_2_)_2_). ^13^C NMR (75 MHz, DMSO-*d*_6_/CCl_4_, 1/3) δ 20.33, 24.88, 24.93, 25.09, 28.50, 48.38, 49.58, 53.36, 65.46, 96.36, 114.66, 147.95, 148.77, 156.92. Anal. calcd. for C_17_H_23_ClN_4_: C 64.04; H 7.27; N 17.57%. Found: C 63.69; H 7.46; N 17.79%.

3-Chloro-7-isobutyl-1-piperidin-1-yl-5,6,7,8-tetrahydro-2,7-naphthyridine-4-carbonitrile (**2e**). Yellow solid; yield 85%, mp 91–93 °C. ^1^H NMR (300 MHz, DMSO-*d*_6_/CCl_4_, 1/3) δ 0.90 (d, *J =* 6.5 Hz, 6H, CH(CH_3_)_2_), 1.64–1.70 (m, 6H, C_5_H_10_N), 1.75–1.90 (m, 1H, CH(CH_3_)_2_), 2.25 (d, *J =* 7.3 Hz, 2H, CHCH_2_), 2.69 (t, *J =* 6.1 Hz, 2H, NCH_2_CH_2_), 2.89–2.95 (m, 2H, NCH_2_CH_2_), 3.26–3.31 (m, 6H, NCH_2_, N(CH_2_)_2_). ^13^C NMR (75 MHz, DMSO-*d*_6_/CCl_4_, 1/3) δ 20.35, 23.77, 25.21, 25.30, 28.45, 48.84, 49.51, 53.33, 65.38, 100.00, 114.05, 119.07, 147.99, 150.18, 159.92. Anal. calcd. for C_18_H_25_ClN_4_: C 64.95; H 7.57; N 16.83%. Found: C 65.32; H 7.73; N 17.07%.

1-Azepan-1-yl-3-chloro-7-isobutyl-5,6,7,8-tetrahydro-2,7-naphthyridine-4-carbonitrile (**2f**). Colorless solid; yield 82%, mp 123–125 °C. ^1^H NMR (300 MHz, DMSO-*d*_6_/CCl_4_, 1/3) δ 0.90 (d, *J =* 6.5 Hz, 6H, CH(CH_3_)_2_), 1.54–1.63 (m, 4H, C_6_H_12_N), 1.75–1.89 (m, 5H, CH(CH_3_)_2_, C_6_H_12_N), 2.25 (d, *J =* 7.3 Hz, 2H, CHCH_2_), 2.67 (t, *J* = 5.9 Hz, 2H, NCH_2_CH_2_), 2.90 (t, *J =* 5.8 Hz, 2H, NCH_2_CH_2_), 3.33 (s, 2H, NCH_2_), 3.56–3.61 (m, 4H, N(CH_2_)_2_). ^13^C NMR (75 MHz, DMSO-*d*_6_/CCl_4_, 1/3) δ 20.32, 25.20, 26.23, 27.78, 28.74, 48.53, 50.92, 54.34, 65.46, 97.50, 114.47, 115.76, 147.48, 149.81, 158.94. Anal. calcd. for C_19_H_27_ClN_4_: C 65.78; H 7.85; N 16.15%. Found: C 66.11; H 7.67; N 16.35%.

7-Benzyl-3-chloro-1-piperidin-1-yl-5,6,7,8-tetrahydro-2,7-naphthyridine-4-carbonitrile (**2h**). Colorless solid; yield 84%, mp 128–129 °C; IR *ν*/cm^–1^: 2219 (C≡N). ^1^H NMR (300 MHz, DMSO-*d*_6_/CCl_4_, 1/3) δ 1.55–1.67 (m, 6H, C_5_H_110_N), 2.74 (t, *J* = 6.0 Hz, 2H, NCH_2_CH_2_), 2.93 (t, *J* = 6.0 Hz, 2H, NCH_2_CH_2_), 3.23–3.28 (m, 4H, N(CH_2_)_2_), 3.33 (s, 2H, NCH_2_), 3.67 (s, 2H, NCH_2_Ph), 7.19–7.33 (m, 5H, Ph). ^13^C NMR (75 MHz, DMSO-*d*_6_/CCl_4_, 1/3) δ 23.73, 25.21, 28.35, 48.21, 49.42, 52.55, 61.34, 99.82, 114.03, 118.81, 126.67, 127.67, 128.26, 137.10, 148.02, 150.02, 159.77. Anal. calcd. for C_21_H_23_ClN_4_: C 68.75; H 6.32; N 15.27%. Found: C 68.39; H 6.51; N 15.04%.

1-Azepan-1-yl-7-benzyl-3-chloro-5,6,7,8-tetrahydro-2,7-naphthyridine-4-carbonitrile (**2i**). Brown solid; yield 87%, mp 117–119 °C; IR *ν*/cm^–1^: 2209 (C≡N). ^1^H NMR (300 MHz, DMSO-*d*_6_/CCl_4_, 1/3) δ 1.46–1.54 (m, 4H, C_6_H_12_N), 1.66–1.76 (m, 4H, C_6_H_12_N), 2.73 (t, *J* = 6.0 Hz, 2H, NCH_2_CH_2_), 2.88–2.94 (m, 2H, NCH_2_CH_2_), 3.36 (s, 2H, NCH_2_), 3.50–3.56 (m, 4H, N(CH_2_)_2_), 3.67 (s, 2H, NCH_2_Ph), 7.18–7.32 (m, 5H, Ph). ^13^C NMR (75 MHz, DMSO-*d*_6_/CCl_4_, 1/3) δ 26.07, 27.76, 28.73, 48.10, 50.87, 53.50, 61.52, 97.42, 114.47, 115.64, 126.67, 127.72, 128.22, 137.21, 147.53, 149.69, 158.87. Anal. calcd. for C_22_H_25_ClN_4_: C 69.37; H 6.62; N 14.71%. Found: C 69.03; H 6.79; N 14.95%.

### 3.2. General Procedure for the Synthesis of Compounds **3a**–**l**

A mixture of compound **2** (1 mmol) and of corresponding amine (5 mL) was refluxed for 1 h. The reaction mixture was cooled, and water (50 mL) was added, and the separated crystals were filtered off, washed with water, dried, and recrystallized from a mixture of ethanol–water (2:1).

3-[(2-Hydroxyethyl)amino]-7-isopropyl-1-pyrrolidin-1-yl-5,6,7,8-tetrahydro-2,7-naphthyridine-4-carbonitrile (**3a**). Yellow solid; yield 78%, mp 157–159 °C; IR *ν*/cm^–1^: 2191 (C≡N), 3358 (NH). ^1^H NMR (300 MHz, DMSO-*d*_6_/CCl_4_, 1/3) δ 1.07 (d, *J =* 6.5 Hz, 6H, CH(CH_3_)_2_), 1.88–1.94 (m, 4H, C_4_H_8_N), 2.62–2.74 (m, 4H, NCH_2_CH_2_), 2.81 (sp, *J =* 6.5 Hz, 1H, CH(CH_3_)_2_), 3.41–3.47 (m, 2H, CH_2_OH), 3.45 (s, 2H, NCH_2_), 3.51–3.58 (m, 6H, NHCH_2_, N(CH_2_)_2_), 4.34 (t, *J =* 5.4 Hz, 1H, OH), 5.79 (t, *J =* 5.3 Hz, 1H, NH). ^13^C NMR (75 MHz, DMSO-*d*_6_/CCl_4_, 1/3) δ 18.16, 24.97, 28.95, 43.18, 44.42, 48.72, 49.12, 53.37, 60.12, 77.90, 105.75, 117.40, 147.86, 156.36, 157.54. Anal. calcd. for C_18_H_27_N_5_O: C 65.62; H 8.26; N 21.26%. Found: C 65.24; H 8.44; N 21.52%.

3-[(2-Hydroxyethyl)amino]-7-isopropyl-1-piperidin-1-yl-5,6,7,8-tetrahydro-2,7-naphthyridine-4-carbonitrile (**3b**). Cream-colored solid; yield 83%, mp 124–125 °C; IR *ν*/cm^–1^: 2198 (C≡N), 3144, 3396 (NH). ^1^H NMR (300 MHz, DMSO-*d*_6_/CCl_4_, 1/3) δ 1.07 (d, *J =* 6.5 Hz, 6H, CH(CH_3_)_2_), 1.61–1.69 (m, 6H, C_5_H_10_N), 2.66–2.68 (m, 4H, NCH_2_CH_2_), 2.80 (sp, *J =* 6.5 Hz, 1H, CH(CH_3_)_2_), 3.13–3.19 (m, 4H, N(CH_2_)_2_), 3.29 (s, 2H, NCH_2_), 3.42–3.49 (m, 2H, CH_2_OH), 3.52–3.58 (m, 2H, NHCH_2_), 4.38 (t, *J =* 5.4 Hz, 1H, OH), 5.93 (t, *J =* 5.4 Hz, 1H, NH). ^13^C NMR (75 MHz, DMSO-*d*_6_/CCl_4_, 1/3) δ 18.23, 24.12, 25.43, 28.72, 43.22, 44.97, 48.19, 49.55, 53.21, 60.06, 81.33, 109.54, 116.59, 148.78, 156.51, 160.67. Anal. calcd. for C_19_H_29_N_5_O: C 66.44; H 8.51; N 20.39%. Found: C 66.05; H 8.69; N 20.66%. ESI HRMS [C_19_H_29_N_5_O+H^+^] Calculated: 344.2450. Found: 344.2464.

1-Azepan-1-yl-3-[(2-hydroxyethyl)amino]-7-isopropyl-5,6,7,8-tetrahydro-2,7-naphthyridine-4-carbonitrile (**3c**). Yellow solid; yield 77%, mp 120–122 °C; IR *ν*/cm^–1^: 2190 (C≡N), 3171, 3415, 3438 (NH). ^1^H NMR (300 MHz, DMSO-*d*_6_/CCl_4_, 1/3) δ 1.07 (d, *J =* 6.5 Hz, 6H, CH(CH_3_)_2_), 1.57–1.63 (m, 4H, C_6_H_12_N), 1.74–1.84 (m, 4H, C_6_H_12_N), 2.63–2.77 (m, 4H, NCH_2_CH_2_), 2.80 (sp, *J =* 6.5 Hz, 1H, CH(CH_3_)_2_), 3.32 (s, 2H, NCH_2_), 3.41–3.58 (m, 8H, NHCH_2_CH_2_, N(CH_2_)_2_), 4.35 (t, *J =* 5.4 Hz, 1H, OH), 5.80 (t, *J =* 5.3 Hz, 1H, NH). ^13^C NMR (75 MHz, DMSO-*d*_6_/CCl_4_, 1/3) δ 18.23, 26.33, 28.31, 29.09, 43.24, 44.71, 49.50, 50.78, 53.33, 60.00, 79.38, 106.95, 117.07, 148.59, 156.00, 159.73. Anal. calcd. for C_20_H_31_N_5_O: C 67.19; H 8.74; N 19.59%. Found: C 67.54; H 8.58; N 19.36%.

3-[(2-Hydroxypropyl)amino]-7-isopropyl-1-pyrrolidin-1-yl-5,6,7,8-tetrahydro-2,7-naphthyridine-4-carbonitrile (**3d**). Yellow solid; yield 81%, mp 122–124 °C; IR *ν*/cm^–1^: 2192 (C≡N), 3349 (NH). ^1^H NMR (300 MHz, DMSO-*d*_6_/CCl_4_, 1/3) δ 1.07 (d, *J =* 6.5 Hz, 6H, CH(CH_3_)_2_), 1.08 (d, *J =* 6.3 Hz, 3H, CHCH_3_), 1.87–1.95 (m, 4H, C_4_H_8_N), 2.62–2.74 (m, 4H, NCH_2_CH_2_), 2.81 (sp, *J =* 6.5 Hz, 1H, CH(CH_3_)_2_), 3.11 (ddd, *J =* 13.3, 7.5, 4.7 Hz, 1H, NHCH_2_), 3.45 (s, 2H, NCH_2_), 3.49 (ddd, *J =* 13.3, 6.5, 3.7 Hz, 1H, NHCH_2_), 3.52–3.57 (m, 4H, N(CH_2_)_2_), 3.73–3.84 (m, 1H, CHCH_3_), 4.46 (d, *J =* 4.4 Hz, 1H, OH), 5.78 (dd, *J =* 6.5, 4.7 Hz, 1H, NH). ^13^C NMR (75 MHz, DMSO-*d*_6_/CCl_4_, 1/3) δ 18.14, 20.84, 24.96, 28.96, 44.39, 48.04, 48.75, 49.10, 53.36, 65.37, 77.90, 105.77, 117.36, 147.87, 156.42, 157.50. Anal. calcd. for C_19_H_29_N_5_O: C 66.44; H 8.51; N 20.39%. Found: C 66.75; H 8.36; N 20.18%.

3-[(2-Hydroxypropyl)amino]-7-isopropyl-1-piperidin-1-yl-5,6,7,8-tetrahydro-2,7-naphthyridine-4-carbonitrile (**3e**). Yellow solid; yield 81%, mp 115–117 °C; IR *ν*/cm^–1^: 2196 (C≡N), 3407, 3419, 3482 (NH). ^1^H NMR (300 MHz, DMSO-*d*_6_/CCl_4_, 1/3) δ 1.07 (d, *J =* 6.5 Hz, 6H, CH(CH_3_)_2_), 1.08 (d, *J =* 6.2 Hz, 3H, CHCH_3_), 1.61–1.69 (m, 6H, C_5_H_10_N), 2.65–2.76 (m, 4H, NCH_2_CH_2_), 2.80 (sp, *J =* 6.5 Hz, 1H, CH(CH_3_)_2_), 3.12 (ddd, *J =* 13.0, 7.3, 4.5 Hz, 1H, NHCH_2_), 3.12–3.19 (m, 4H, N(CH_2_)_2_), 3.29 (s, 2H, NCH_2_), 3.52 (ddd, *J =* 13.2, 7.5, 4.5 Hz, 1H, NHCH_2_), 3.73–3.85 (m, 1H, CHCH_3_), 4.47 (d, *J =* 4.4 Hz, 1H, OH), 5.92 (dd, *J =* 6.5, 4.5 Hz, 1H, NH). ^13^C NMR (75 MHz, DMSO-*d*_6_/CCl_4_, 1/3) δ 18.23, 20.79, 24.11, 25.43, 28.73, 44.97, 48.07, 48.18, 49.55, 53.22, 65.29, 81.32, 109.60, 116.57, 148.80, 156.56, 160.65. Anal. calcd. for C_20_H_31_N_5_O: C 67.19; H 8.74; N 19.59%. Found: C 66.83; H 8.56; N 19.83%.

1-Azepan-1-yl-3-[(2-hydroxypropyl)amino]-7-isopropyl-5,6,7,8-tetrahydro-2,7-naphthyridine-4-carbonitrile (**3f**). Colorless solid; yield 85%, mp 116–118 °C; IR *ν*/cm^–1^: 2188 (C≡N), 3178, 3418 (NH). ^1^H NMR (300 MHz, DMSO-*d*_6_/CCl_4_, 1/3) δ 1.07 (d, *J =* 6.4 Hz, 3H, CHCH_3_), 1.09 (d, *J =* 6.5 Hz, 6H, CH(CH_3_)_2_), 1.55–1.64 (m, 4H, C_6_H_12_N), 1.75–1.83 (m, 4H, C_6_H_12_N), 2.63–2.69 (m, 2H, NCH_2_CH_2_), 2.72–2.78 (m, 2H, NCH_2_CH_2_), 2.80 (sp, *J =* 6.5 Hz, 1H, CH(CH_3_)_2_), 3.08 (ddd, *J =* 12.6, 8.0, 4.7 Hz, 1H, NHCH_2_), 3.31 (s, 2H, NCH_2_), 3.47–3.55 (m, 5H, NHCH_2_, N(CH_2_)_2_), 3.72–3.84 (m, 1H, CHCH_3_), 4.43 (d, *J =* 4.4 Hz, 1H, OH), 5.79 (dd, *J =* 6.5, 4.7 Hz, 1H, NH). ^13^C NMR (75 MHz, DMSO-*d*_6_/CCl_4_, 1/3) δ 18.24, 20.80, 26.36, 28.33, 29.12, 44.71, 48.11, 49.53, 50.77, 53.36, 65.22, 79.35, 106.98, 117.10, 148.63, 156.05, 159.75. Anal. calcd. for C_21_H_33_N_5_O: C 67.89; H 8.95; N 18.85%. Found: C 67.55; H 9.09; N 18.58%.

3-[(2-Hydroxyethyl)amino]-7-isobutyl-1-pyrrolidin-1-yl-5,6,7,8-tetrahydro-2,7-naphthyridine-4-carbonitrile (**3g**). Colorless solid; yield 79%, mp 147–149 °C; IR *ν*/cm^–1^: 2194 (C≡N), 3347 (NH). ^1^H NMR (300 MHz, DMSO-*d*_6_/CCl_4_, 1/3) δ 0.89 (d, *J =* 6.5 Hz, 6H, CH(CH_3_)_2_), 1.73–1.86 (m, 1H, CH(CH_3_)_2_), 1.86–1.94 (m, 4H, C_4_H_8_N), 2.20 (d, *J =* 7.4 Hz, 2H, CHCH_2_), 2.82 (t, *J =* 5.9 Hz, 2H, NCH_2_CH_2_), 2.74 (t, *J =* 5.8 Hz, 2H, NCH_2_CH_2_), 3.35 (s, 2H, NCH_2_), 3.41–3.47 (m, 2H, CH_2_OH), 3.51–3.57 (m, 6H, NHCH_2_, N(CH_2_)_2_), 4.35 (t, *J =* 5.4 Hz, 1H, OH), 5.82 (t, *J =* 5.4 Hz, 1H, NH). ^13^C NMR (75 MHz, DMSO-*d*_6_/CCl_4_, 1/3) δ 20.42, 24.93, 25.21, 28.38, 43.17, 49.09, 49.25, 53.82, 60.13, 65.98, 77.97, 105.18, 117.32, 147.63, 156.41, 157.46. Anal. calcd. for C_19_H_29_N_5_O: C 66.44; H 8.51; N 20.39%. Found: C 66.07; H 8.69; N 20.16%.

3-[(2-Hydroxyethyl)amino]-7-isobutyl-1-piperidin-1-yl-5,6,7,8-tetrahydro-2,7-naphthyridine-4-carbonitrile (**3h**). Colorless solid; yield 76%, mp 111–113 °C; IR *ν*/cm^–1^: 2196 (C≡N), 3343 (NH). ^1^H NMR (300 MHz, DMSO-*d*_6_/CCl_4_, 1/3) δ 0.90 (d, *J =* 6.5 Hz, 6H, CH(CH_3_)_2_), 1.61–1.67 (m, 6H, C_5_H_10_N), 1.74–1.89 (m, 1H, CH(CH_3_)_2_), 2.20 (d, *J =* 7.2 Hz, 2H, CHCH_2_), 2.61 (t, *J =* 5.8 Hz, 2H, NCH_2_CH_2_), 2.78 (t, *J =* 5.8 Hz, 2H, NCH_2_CH_2_), 3.13–3.20 (m, 6H, NCH_2_, N(CH_2_)_2_), 3.43–3.49 (m, 2H, CH_2_OH), 3.52–3.59 (m, 2H, NHCH_2_), 4.39 (t, *J =* 5.4 Hz, 1H, OH), 5.97 (t, *J =* 5.4 Hz, 1H, NH). ^13^C NMR (75 MHz, DMSO-*d*_6_/CCl_4_, 1/3) δ 20.45, 24.11, 25.23, 25.39, 28.22, 43.21, 49.48, 49.62, 53.30, 60.03, 65.82, 81.25, 108.93, 116.58, 148.53, 156.55, 160.61. Anal. calcd. for C_20_H_31_N_5_O: C 67.19; H 8.74; N 19.59%. Found: C 66.80; H 8.91; N 19.33%. ESI HRMS [C_20_H_31_N_5_O+H^+^] Calculated: 358.2607. Found: 358.2630.

1-Azepan-1-yl-3-[(2-hydroxyethyl)amino]-7-isobutyl-5,6,7,8-tetrahydro-2,7-naphthyridine-4-carbonitrile (**3i**). Colorless solid; yield 83%, mp 100–102 °C; IR *ν*/cm^–1^: 2198 (C≡N), 3350 (NH). ^1^H NMR (300 MHz, DMSO-*d*_6_/CCl_4_, 1/3) δ 0.89 (d, *J =* 6.5 Hz, 6H, CH(CH_3_)_2_), 1.51–1.62 (m, 4H, C_6_H_12_N, CH(CH_3_)_2_), 1.72–1.87 (m, 5H, C_6_H_12_N), 2.21 (d, *J =* 7.4 Hz, 2H, CHCH_2_), 2.57 (t, *J =* 6.0 Hz, 2H, NCH_2_CH_2_), 2.77 (t, *J =* 5.8 Hz, 2H, NCH_2_CH_2_), 3.20 (s, 2H, NCH_2_), 3.41–3.59 (m, 8H, NHCH_2_CH_2_, N(CH_2_)_2_), 4.36 (t, *J =* 5.4 Hz, 1H, OH), 5.82 (t, *J =* 5.3 Hz, 1H, NH). ^13^C NMR (75 MHz, DMSO-*d*_6_/CCl_4_, 1/3) δ 20.43, 25.26, 26.33, 28.25, 28.57, 43.24, 49.34, 50.71, 54.68, 59.98, 65.95, 79.26, 106.23, 117.08, 148.33, 156.04, 159.50. Anal. calcd. for C_21_H_33_N_5_O: C 67.89; H 8.95; N 18.85%. Found: C 68.22; H 9.17; N 19.11%.

3-[(2-Hydroxypropyl)amino]-7-isobutyl-1-pyrrolidin-1-yl-5,6,7,8-tetrahydro-2,7-naphthyridine-4-carbonitrile (**3j**). Colorless solid; yield 74%, mp 123–125 °C; IR *ν*/cm^–1^: 2187 (C≡N), 3369, 3481 (NH). ^1^H NMR (300 MHz, DMSO-*d*_6_/CCl_4_, 1/3) δ 0.89 (d, *J =* 6.5 Hz, 6H, CH(CH_3_)_2_), 1.03 (d, *J =* 6.3 Hz, 3H, CHCH_3_), 1.73–1.87 (m, 1H, CH(CH_3_)_2_, 1.85–1.97 (m, 4H, C_4_H_8_N), 2.20 (d, *J =* 7.4 Hz, 2H, CHCH_2_), 2.58 (t, *J =* 6.0 Hz, 2H, NCH_2_CH_2_), 2.74 (t, *J =* 5.8 Hz, 2H, NCH_2_CH_2_), 3.12 (ddd, *J =* 13.3, 7.5, 4.7 Hz, 1H, NHCH_2_), 3.35 (s, 2H, NCH_2_), 3.48 (ddd, *J =* 13.3, 6.5, 3.7 Hz, 1H, NHCH_2_), 3.50–3.58 (m, 4H, N(CH_2_)_2_), 3.72–3.86 (m, 1H, CHCH_3_), 4.45 (d, *J =* 4.4 Hz, 1H, OH), 5.80 (dd, *J =* 6.3, 4.8 Hz, 1H, NH). ^13^C NMR (75 MHz, DMSO-*d*_6_/CCl_4_, 1/3) δ 20.41, 20.83, 24.93, 25.21, 28.39, 48.03, 49.08, 49.23, 53.82, 65.35, 65.98, 77.93, 105.20, 117.29, 147.64, 156.46, 157.43. Anal. calcd. for C_20_H_31_N_5_O: C 67.19; H 8.74; N 19.59%. Found: C 67.54; H 8.89; N 19.84%. ESI HRMS [C_20_H_31_N_5_O+H^+^] Calculated: 358.2607. Found: 358.2612.

3-[(2-Hydroxypropyl)amino]-7-isobutyl-1-piperidin-1-yl-5,6,7,8-tetrahydro-2,7-naphthyridine-4-carbonitrile (**3k**). Colorless solid; yield 80%, mp 105–107 °C; IR *ν*/cm^–1^: 2207 (C≡N), 3355, 3456 (NH). ^1^H NMR (300 MHz, DMSO-*d*_6_/CCl_4_, 1/3) δ 0.90 (d, *J =* 6.5 Hz, 6H, CH(CH_3_)_2_), 1.09 (d, *J =* 6.3 Hz, 3H, CHCH_3_), 1.61–1.69 (m, 6H, C_5_H_10_N), 1.74–1.89 (m, 1H, CH(CH_3_)_2_, 2.20 (d, *J =* 7.4 Hz, 2H, CHCH_2_), 2.61 (t, *J =* 5.8 Hz, 2H, NCH_2_CH_2_), 2.78 (t, *J =* 5.7 Hz, 2H, NCH_2_CH_2_), 3.08–3.20 (m, 7H, NHCH_2_ N(CH_2_)_2_, NCH_2_), 3.52 (ddd, *J =* 13.3, 6.7, 3.5 Hz, 1H, NHCH_2_), 3.73–3.85 (m, 1H, CHCH_3_), 4.48 (d, *J =* 4.4 Hz, 1H, OH), 5.95 (dd, *J =* 6.2, 4.9 Hz, 1H, NH). ^13^C NMR (75 MHz, DMSO-*d*_6_/CCl_4_, 1/3) δ 20.43, 20.78, 24.08, 25.23, 25.38, 28.22, 48.05, 49.47, 49.60, 53.26, 65.25, 65.82, 81.26, 108.97, 116.54, 148.54, 156.58, 160.59. Anal. calcd. for C_21_H_33_N_5_O: C 67.89; H 8.95; N 18.85%. Found: C 67.51; H 8.76; N 18.61%.

1-Azepan-1-yl-3-[(2-hydroxypropyl)amino]-7-isobutyl-5,6,7,8-tetrahydro-2,7-naphthyridine-4-carbonitrile (**3l**). Yellow solid; yield 86%, mp 103–105 °C; IR *ν*/cm^–1^: 2196 (C≡N), 3365, 3458 (NH). ^1^H NMR (300 MHz, DMSO-*d*_6_/CCl_4_, 1/3) δ 0.89 (d, *J =* 6.5 Hz, 6H, CH(CH_3_)_2_), 1.09 (d, *J =* 6.4 Hz, 3H, CHCH_3_), 1.51–1.62 (m, 4H, C_6_H_12_N), 1.72–1.86 (m, 5H, CH(CH_3_)_2_, C_6_H_12_N), 2.19 (d, *J =* 7.4 Hz, 2H, CHCH_2_), 2.58 (t, *J =* 6.0 Hz, 2H, NCH_2_CH_2_), 2.77 (t, *J =* 5.9 Hz, 2H, NCH_2_CH_2_), 3.09 (ddd, *J =* 12.3, 8.1, 4.8 Hz, 1H, NHCH_2_), 3.20 (s, 2H, NCH_2_), 3.46–3.55 (m, 5H, NHCH_2_, N(CH_2_)_2_), 3.73–3.84 (m, 1H, CHCH_3_), 4.45 (d, *J =* 4.4 Hz, 1H, OH), 5.80 (dd, *J =* 6.5, 4.7 Hz, 1H, NH). ^13^C NMR (75 MHz, DMSO-*d*_6_/CCl_4_, 1/3) δ 20.46, 20.81, 25.30, 26.36, 28.26, 28.60, 48.10, 49.36, 50.72, 54.71, 65.21, 65.99, 79.28, 106.28, 117.10, 148.37, 156.10, 159.54. Anal. calcd. for C_22_H_35_N_5_O: C 68.54; H 9.15; N 18.16%. Found: C 68.18; H 9.31; N 17.92%.

### 3.3. General Procedures for the Synthesis of Compounds **4a**–**l**

A mixture of compound **2** (1 mmol) and of the corresponding amine (5 mL) was refluxed for 15 h. The reaction mixture was cooled, and water (50 mL) was added, and the separated crystals (apart from compounds **4e** and **4f,** which were isolated as a light yellow oil) were filtered off, washed with water, dried, and recrystallized from a mixture of ethanol–water (2:1).

8-[(2-Hydroxyethyl)amino]-2-isopropyl-6-pyrrolidin-1-yl-3,4-dihydro-2,7-naphthyridin-1(2*H*)-one (**4a**). Colorless solid; yield 57%, mp 157–159 °C; IR *ν*/cm^–1^: 1601 (C=O), 3304, 3411 (NH). ^1^H NMR (300 MHz, DMSO-*d*_6_/CCl_4_, 1/3) δ 1.13 (d, *J =* 6.8 Hz, 6H, CH(CH_3_)_2_), 1.94–2.04 (m, 4H, C_4_H_8_N), 2.67 (t, *J =* 6.5 Hz, 2H, NCH_2_CH_2_), 3.25 (t, *J =* 6.5 Hz, 2H, NCH_2_CH_2_), 3.41–3.61 (m, 8H, NHCH_2_CH_2_, N(CH_2_)_2_), 4.47 (t, *J =* 4.9 Hz, 1H, OH), 4.86 (sp, *J =* 6.8 Hz, 1H, CH(CH_3_)_2_), 5.34 (s, 1H, 5-CH), 9.09 (t, *J =* 5.2 Hz, 1H, NH). ^13^C NMR (75 MHz, DMSO-*d*_6_/CCl_4_, 1/3) δ 19.33, 24.79, 28.94, 37.50, 41.68, 42.83, 45.79, 61.25, 91.49, 94.68, 148.69, 156.37, 158.13, 164.94. Anal. calcd. for C_17_H_26_N_4_O_2_: C 64.12; H 8.23; N 17.60%. Found: C 63.78; H 8.05; N 17.39%. ESI HRMS [C_17_H_26_N_4_O_2_+H^+^] Calculated: 319.2134. Found: 319.2145.

8-[(2-Hydroxyethyl)amino]-2-isopropyl-6-piperidin-1-yl-3,4-dihydro-2,7-naphthyridin-1(2*H*)-one (**4b**). Cream-colored solid; yield 61%, mp 154–156 °C; IR *ν*/cm^–1^: 1600 (C=O), 3319, 3436 (NH). ^1^H NMR (300 MHz, DMSO-*d*_6_/CCl_4_, 1/3) δ 1.13 (d, *J =* 6.8 Hz, 6H, CH(CH_3_)_2_), 1.53–1.71 (m, 6H, C_5_H_10_N), 2.66 (t, *J =* 6.5 Hz, 2H, NCH_2_CH_2_), 3.25 (t, *J =* 6.5 Hz, 2H, NCH_2_CH_2_), 3.43–3.50 (m, 2H, CH_2_OH), 3.53–3.62 (m, 6H, CH_2_OH, N(CH_2_)_2_), 4.85 (sp, *J =* 6.8 Hz, 1H, CH(CH_3_)_2_), 4.29 (t, *J =* 4.9 Hz, 1H, OH), 5.59 (s, 1H, 5-CH), 9.00 (t, *J =* 5.4 Hz, 1H, NH). ^13^C NMR (75 MHz, DMSO-*d*_6_/CCl_4_, 1/3) δ 19.31, 24.35, 25.01, 29.06, 37.51, 41.69, 42.70, 44.94, 60.61, 91.02, 91.06, 95.13, 149.10, 157.75, 157.94, 164.67. Anal. calcd. for C_18_H_28_N_4_O_2_: C 65.03; H 8.49; N 16.85%. Found: C 65.40; H 8.34; N 17.11%. ESI HRMS [C_18_H_28_N_4_O_2_+H^+^] Calculated: 333.2291. Found: 333.2313.

6-Azepan-1-yl-8-[(2-hydroxyethyl)amino]-2-isopropyl-3,4-dihydro-2,7-naphthyridin-1(2*H*)-one (**4c**). Cream-colored solid; yield 56%, mp 115–117 °C; IR *ν*/cm^–1^: 1600 (C=O), 3310, 3433 (NH). ^1^H NMR (300 MHz, DMSO-*d*_6_/CCl_4_, 1/3) δ 1.12 (d, *J =* 6.8 Hz, 6H, CH(CH_3_)_2_), 1.50–1.59 (m, 4H, C_6_H_12_N), 1.72–1.81 (m, 4H, C_6_H_12_N), 2.66 (t, *J =* 6.5 Hz, 2H, NCH_2_CH_2_), 3.26 (t, *J =* 6.5 Hz, 2H, NCH_2_CH_2_), 3.44–3.50 (m, 2H, CH_2_OH), 3.55–3.64 (m, 6H, NHCH_2_, N(CH_2_)_2_), 4.23 (br, 1H, OH), 4.85 (sp, *J =* 6.8 Hz, 1H, CH(CH_3_)_2_), 5.47 (s, 1H, 5-CH), 9.00 (br, 1H, NH). ^13^C NMR (75 MHz, DMSO-*d*_6_/CCl_4_, 1/3) δ 19.33, 26.33, 27.29, 29.11, 37.51, 41.65, 42.71, 47.04, 60.64, 90.13, 90.17, 94.68, 148.86, 157.31, 157.88, 164.83. Anal. calcd. for C_19_H_30_N_4_O_2_: C 65.87; H 8.73; N 16.17%. Found: C 65.54; H 8.59; N 15.90%. ESI HRMS [C_19_H_30_N_4_O_2_+H^+^] Calculated: 347.2447. Found: 347.2449.

8-[(2-Hydroxypropyl)amino]-2-isopropyl-6-pyrrolidin-1-yl-3,4-dihydro-2,7-naphthyridin-1(2*H*)-one (**4d**). Yellow solid; yield 60%, mp 128–130 °C; IR *ν*/cm^–1^: 1602 (C=O), 3319 (NH). ^1^H NMR (300 MHz, DMSO-*d*_6_/CCl_4_, 1/3) δ 1.11 (d, *J =* 6.8 Hz, 3H, CHCH_3_), 1.13 (d, *J =* 6.8 Hz, 6H, CH(CH_3_)_2_), 1.94–2.03 (m, 4H, C_4_H_8_N), 2.67 (t, *J =* 6.5 Hz, 2H, NCH_2_CH_2_), 3.25 (t, *J =* 6.5 Hz, 2H, NCH_2_CH_2_), 3.29 (ddd, *J =* 13.5, 6.6, 5.5 Hz, 1H, NHCH_2_), 3.43 (ddd, *J =* 13.5, 6.3, 4.0 Hz, 1H, NHCH_2_), 3.41–3.47 (m, 4H, N(CH_2_)_2_), 3.75–3.86 (m, 1H, CHCH_3_), 4.68 (d, *J =* 4.4 Hz, 1H, OH), 4.86 (sp, *J =* 6.8 Hz, 1H, CH(CH_3_)_2_), 5.34 (s, 1H, 5-CH), 9.14 (t, *J =* 5.8 Hz, 1H, NH). ^13^C NMR (75 MHz, DMSO-*d*_6_/CCl_4_, 1/3) δ 19.30, 21.02, 24.75, 28.91, 37.46, 41.67, 45.79, 47.91, 66.42, 91.51, 91.56, 94.63, 148.76, 156.25, 158.23, 164.89. Anal. calcd. for C_18_H_28_N_4_O_2_: C 65.03; H 8.49; N 16.85%. Found: C 65.41; H 8.66; N 17.08%.

8-[(2-Hydroxypropyl)amino]-2-isopropyl-6-piperidin-1-yl-3,4-dihydro-2,7-naphthyridin-1(2*H*)-one (**4e**). Light yellow oil; yield 55%. ^1^H NMR (300 MHz, DMSO-*d*_6_/CCl_4_, 1/3) δ 1.12 (d, *J =* 6.7 Hz, 6H, CH(CH_3_)_2_), 1.13 (d, *J =* 6.5 Hz, 3H, CHCH_3_), 1.53–1.71 (m, 6H, C_5_H_10_N), 2.66 (t, *J =* 6.4 Hz, 2H, NCH_2_CH_2_), 3.25 (t, *J =* 6.4 Hz, 2H, NCH_2_CH_2_), 3.28 (ddd, *J =* 13.5, 6.6, 5.5 Hz, 1H, NHCH_2_), 3.35–3.46 (m, 1H, NHCH_2_), 3.52–3.58 (m, 4H, N(CH_2_)_2_), 3.77–3.88 (m, 1H, CHCH_3_), 4.39 (br, 1H, OH), 4.86 (sp, *J =* 6.8 Hz, 1H, CH(CH_3_)_2_), 5.60 (s, 1H, 5-CH), 9.06 (t, *J =* 5.6 Hz, 1H, NH). ^13^C NMR (75 MHz, DMSO-*d*_6_/CCl_4_, 1/3) δ 19.34, 21.13, 24.39, 25.05, 29.09, 37.53, 41.74, 45.00, 47.77, 65.89, 95.09, 95.55, 149.24, 157.89, 157.94, 164.73. Anal. calcd. for C_19_H_30_N_4_O_2_: C 65.87; H 8.73; N 16.17%. Found: C 66.25; H 8.91; N 16.38%.

6-Azepan-1-yl-8-[(2-hydroxypropyl)amino]-2-isopropyl-3,4-dihydro-2,7-naphthyridin-1(2*H*)-one (**4f**). Light yellow oil; yield 58%. ^1^H NMR (300 MHz, DMSO-*d*_6_/CCl_4_, 1/3) δ 1.12 (d, *J =* 6.2 Hz, 3H, CHCH_3_), 1.12 (d, *J =* 6.8 Hz, 6H, CH(CH_3_)_2_), 1.51–1.57 (m, 4H, C_6_H_12_N), 1.71–1.82 (m, 4H, C_6_H_12_N), 2.66 (t, *J =* 6.4 Hz, 2H, NCH_2_CH_2_), 3.21–3.32 (m, 3H, NCH_2_CH_2_, NHCH_2_), 3.37–3.46 (m, 1H, NHCH_2_), 3.57–3.63 (m, 4H, N(CH_2_)_2_), 3.75–3.88 (m, 1H, CHCH_3_), 4.31 (d, *J =* 4.4 Hz, 1H, OH), 4.86 (sp, *J =* 6.8 Hz, 1H, CH(CH_3_)_2_), 5.46 (s, 1H, 5-CH), 9.08 (t, *J =* 5.7 Hz, 1H, NH). ^13^C NMR (75 MHz, DMSO-*d*_6_/CCl_4_, 1/3) δ 19.36, 21.12, 26.36, 27.30, 29.12, 37.50, 41.65, 47.05, 47.76, 65.85, 90.23, 90.23, 94.62, 148.94, 157.30, 157.99, 164.86. Anal. calcd. for C_20_H_32_N_4_O_2_: C 66.63; H 8.95; N 15.54%. Found: C 66.28; H 8.82; N 15.34%.

8-[(2-Hydroxyethyl)amino]-2-isobutyl-6-pyrrolidin-1-yl-3,4-dihydro-2,7-naphthyridin-1(2*H*)-one (**4g**). Cream-colored solid; yield 64%, mp 132–134 °C; IR *ν*/cm^–1^: 1601 (C=O), 3317, 3409 (NH). ^1^H NMR (300 MHz, DMSO-*d*_6_/CCl_4_, 1/3) δ 0.92 (d, *J =* 6.6 Hz, 6H, CH(CH_3_)_2_), 1.91–2.04 (m, 5H, CH(CH_3_)_2_, C_4_H_8_N), 2.71 (t, *J =* 6.1 Hz, 2H, NCH_2_CH_2_), 3.18 (d, *J =* 7.2 Hz, 2H, CHCH_2_), 3.37 (t, *J =* 6.3 Hz, 2H, NCH_2_CH_2_), 3.39–3.61 (m, 8H, NHCH_2_CH_2_OH, N(CH_2_)_2_), 4.48 (t, *J =* 4.5 Hz, 1H, OH), 5.34 (s, 1H, 5-CH), 9.05 (t, *J =* 4.5 Hz, 1H, NH). ^13^C NMR (75 MHz, DMSO-*d*_6_/CCl_4_, 1/3) δ 19.86, 24.76, 26.78, 28.67, 42.82, 45.43, 45.77, 53.38, 61.23, 91.56, 94.32, 148.82, 156.41, 158.07, 165.59. Anal. calcd. for C_18_H_28_N_4_O_2_: C 65.03; H 8.49; N 16.85%. Found: C 65.39; H 8.65; N 16.60%. ESI HRMS [C_18_H_28_N_4_O_2_+H^+^] Calculated: 333.2291. Found: 333.2313.

8-[(2-Hydroxyethyl)amino]-2-isobutyl-6-piperidin-1-yl-3,4-dihydro-2,7-naphthyridin-1(2*H*)-one (**4h**). Colorless solid; yield 66%, mp 130–132 °C. IR *ν*/cm^–1^: 1607 (C=O), 3300, 3424 (NH). ^1^H NMR (300 MHz, DMSO-*d*_6_/CCl_4_, 1/3) δ 0.92 (d, *J =* 6.7 Hz, 6H, CH(CH_3_)_2_), 1.53–1.71 (m, 6H, C_5_H_10_N), 1.87–2.01 (m, 1H, CH(CH_3_)_2_), 2.70 (br t, *J =* 6.5 Hz, 2H, NCH_2_CH_2_), 3.19 (d, *J =* 7.4 Hz, 2H, CHCH_2_), 3.38 (br t, *J =* 6.5 Hz, 2H, NCH_2_CH_2_), 3.42–3.49 (m, 2H, NHCH_2_CH_2_OH), 3.53–3.61 (m, 6H, NHCH_2_CH_2_OH, N(CH_2_)_2_), 4.30 (d, *J =* 5.0 Hz, 1H, OH), 5.60 (s, 1H, 5-CH), 8.96 (t, *J =* 5.3 Hz, 1H, NH). ^13^C NMR (75 MHz, DMSO-*d*_6_/CCl_4_, 1/3) δ 19.86, 24.34, 25.00, 26.77, 28.79, 42.69, 44.93, 45.42, 53.35, 60.52, 91.14, 94.74, 149.28, 157.67, 157.98, 165.33. Anal. calcd. for C_19_H_30_N_4_O_2_: C 65.87; H 8.73; N 16.17%. Found: C 66.24; H 8.91; N 16.43%. ESI HRMS [C_19_H_30_N_4_O_2_+H^+^] Calculated: 347.2447. Found: 347.2468.

6-Azepan-1-yl-8-[(2-hydroxyethyl)amino]-2-isobutyl-3,4-dihydro-2,7-naphthyridin-1(2*H*)-one (**4i**). Colorless solid; yield 62%, mp 112–114 °C. IR *ν*/cm^–1^: 1601 (C=O), 3325, 3393 (NH). ^1^H NMR (300 MHz, DMSO-*d*_6_/CCl_4_, 1/3) δ 0.92 (d, *J =* 6.5 Hz, 6H, CH(CH_3_)_2_), 1.49–1.59 (m, 4H, C_6_H_12_N), 1.71–1.82 (m, 4H, C_6_H_12_N), 1.86–2.01 (m, 1H, CH(CH_3_)_2_), 2.70 (br t, *J =* 6.5 Hz, 2H, NCH_2_CH_2_), 3.19 (d, *J =* 7.3 Hz, 2H, CHCH_2_), 3.38 (br t, *J =* 6.5 Hz, 2H, NCH_2_CH_2_), 3.42–3.50 (m, 2H, NHCH_2_CH_2_OH), 3.54–3.65 (m, 6H, NHCH_2_CH_2_OH, N(CH_2_)_2_), 4.28 (d, *J =* 4.9 Hz, 1H, OH), 5.47 (s, 1H, 5-CH), 8.97 (t, *J =* 5.3 Hz, 1H, NH). ^13^C NMR (75 MHz, DMSO-*d*_6_/CCl_4_, 1/3) δ 19.87, 26.33, 26.80, 27.29, 28.85, 42.70, 45.44, 47.04, 53.35, 60.58, 90.26, 94.30, 149.04, 157.35, 157.81, 165.49. Anal. calcd. for C_20_H_32_N_4_O_2_: C 66.63; H 8.95; N 15.54%. Found: C 66.25; H 8.81; N 15.31%. ESI HRMS [C_20_H_32_N_4_O_2_+H^+^] Calculated: 361.2603. Found: 361.2628.

8-[(2-Hydroxypropyl)amino]-2-isobutyl-6-pyrrolidin-1-yl-3,4-dihydro-2,7-naphthyridin-1(2*H*)-one (**4j**). Colorless solid; yield 59%, mp 101–102 °C. IR *ν*/cm^–1^: 1602 (C=O), 3325, 3445 (NH). ^1^H NMR (300 MHz, DMSO-*d*_6_/CCl_4_, 1/3) δ 0.92 (d, *J =* 6.6 Hz, 6H, CH(CH_3_)_2_), 1.11 (d, *J =* 6.2 Hz, 3H, CHCH_3_), 1.87–2.04 (m, 5H, C_4_H_8_N, CH(CH_3_)_2_), 2.71 (t, *J =* 6.7 Hz, 2H, NCH_2_CH_2_), 3.19 (d, *J =* 7.3 Hz, 2H, CHCH_2_), 3.28 (ddd, *J =* 13.5, 6.6, 5.5 Hz, 1H, NHCH_2_), 3.35–3.49 (m, 5H, NHCH_2_, N(CH_2_)_2_) 3.38 (t, *J =* 6.6 Hz, 2H, NCH_2_CH_2_), 3.75–3.86 (m, 1H, CHCH_3_), 4.68 (br, 1H, OH), 5.35 (s, 1H, 5-CH), 9.10 (t, *J =* 5.5 Hz, 1H, NH). ^13^C NMR (75 MHz, DMSO-*d*_6_/CCl_4_, 1/3) δ 19.87, 21.04, 24.78, 26.79, 28.68, 45.42, 45.82, 47.92, 53.40, 66.44, 91.65, 94.29, 148.92, 156.33, 158.20, 165.60. Anal. calcd. for C_19_H_30_N_4_O_2_: C 65.87; H 8.73; N 16.17%. Found: C 65.51; H 8.87; N 16.43%. ESI HRMS [C_19_H_30_N_4_O_2_+H^+^] Calculated: 347.2447. Found: 347.2461.

8-[(2-Hydroxypropyl)amino]-2-isobutyl-6-piperidin-1-yl-3,4-dihydro-2,7-naphthyridin-1(2*H*)-one (**4k**). Cream-colored solid; yield 67%, mp 111–113 °C. IR *ν*/cm^–1^: 1600 (C=O), 3307, 3464 (NH). ^1^H NMR (300 MHz, DMSO-*d*_6_/CCl_4_, 1/3) δ 0.92 (d, *J =* 6.7 Hz, 6H, CH(CH_3_)_2_), 1.12 (d, *J =* 6.2 Hz, 3H, CHCH_3_), 1.53–1.72 (m, 6H, C_5_H_10_N), 1.87–2.02 (m, 1H, CH(CH_3_)_2_), 2.70 (t, *J =* 6.6 Hz, 2H, NCH_2_CH_2_), 3.19 (d, *J =* 7.3 Hz, 2H, CHCH_2_), 3.27 (ddd, *J =* 13.5, 6.6, 5.5 Hz, 1H, NHCH_2_), 3.38 (t, *J =* 6.6 Hz, 2H, NCH_2_CH_2_), 3.40 (ddd, *J =* 13.5, 6.3, 4.0 Hz, 1H, NHCH_2_), 3.52–3.59 (m, 4H, N(CH_2_)_2_), 3.75–3.88 (m, 1H, CHCH_3_), 4.34 (d, *J =* 4.3 Hz, 1H, OH), 5.60 (s, 1H, 5-CH), 9.01 (t, *J =* 5.6 Hz, 1H, NH). ^13^C NMR (75 MHz, DMSO-*d*_6_/CCl_4_, 1/3) δ 19.86, 21.09, 24.34, 25.00, 26.78, 28.79, 44.96, 45.42, 47.69, 53.36, 65.76, 91.17, 94.69, 149.33, 157.77, 157.94, 165.36. Anal. calcd. for C_20_H_32_N_4_O_2_: C 66.63; H 8.95; N 15.54%. Found: C 66.29; H 9.13; N 15.79%.

6-Azepan-1-yl-8-[(2-hydroxypropyl)amino]-2-isobutyl-3,4-dihydro-2,7-naphthyridin-1(2*H*)-one (**4l**). Cream-colored solid; yield 65%, mp 105–107 °C; IR *ν*/cm^–1^: 1602 (C=O), 3349, 3455 (NH). ^1^H NMR (300 MHz, DMSO-*d*_6_/CCl_4_, 1/3) δ 0.92 (d, *J =* 6.4 Hz, 6H, CH(CH_3_)_2_), 1.11 (d, *J =* 6.0 Hz, 3H, CHCH_3_), 1.48–1.60 (m, 4H, C_6_H_12_N), 1.70–1.83 (m, 4H, C_6_H_12_N), 1.86–2.02 (m, 1H, CH(CH_3_)_2_), 2.66–2.75 (m, 2H, NCH_2_CH_2_), 3.19 (d, *J =* 7.1 Hz, 2H, CHCH_2_), 3.17–3.32 (m, 1H, NHCH_2_), 3.33–3.45 (m, 3H, NHCH_2_, NCH_2_CH_2_), 3.51–3.68 (m, 4H, N(CH_2_)_2_), 3.74–3.87 (m, 1H, CHCH_3_), 4.31 (d, *J =* 3.6 Hz, 1H, OH), 5.46 (s, 1H, 5-CH), 9.02 (t, *J =* 4.5 Hz, 1H, NH). ^13^C NMR (75 MHz, DMSO-*d*_6_/CCl_4_, 1/3) δ 19.85, 21.07, 26.34, 26.79, 27.28, 28.85, 45.44, 47.03, 47.73, 53.36, 65.82, 90.29, 94.27, 149.06, 157.32, 157.92, 165.50. Anal. calcd. for C_21_H_34_N_4_O_2_: C 67.35; H 9.15; N 14.96%. Found: C 67.01; H 8.98; N 15.18%. ESI HRMS [C_21_H_34_N_4_O_2_+H^+^] Calculated: 375.2760. Found: 375.2786.

### 3.4. General Procedure for the Synthesis of Compounds **5b,d**–**f**

To a stirred suspension of compound **2a**,**c** (10 mmol) and potassium carbonate (2.76 g, 20 mmol) in absolute DMF (50 mL), 2-mercaptoethanol (0.84 mL, 12 mmol) was added and the reaction mixture was stirred at 90–110 °C for 15 h. After cooling, water was added (50 mL). The resulting crystals were filtered off, washed with water, dried, and recrystallized from ethanol.

3-[(2-Hydroxyethyl)thio]-7-isopropyl-1-piperidin-1-yl-5,6,7,8-tetrahydro-2,7-naphthyridine-4-carbonitrile (**5b**). Colorless solid; yield 85%, mp 156–158 °C; IR *ν*/cm^–1^: 2211 (C≡N), 3165 (OH). ^1^H NMR (300 MHz, DMSO-*d*_6_/CCl_4_, 1/3) δ 1.08 (d, *J =* 6.5 Hz, 6H, CH(CH_3_)_2_), 1.65–1.72 (m, 6H, C_5_H_110_N), 2.70–2.76 (m, 2H, NCH_2_CH_2_), 2.79–2.90 (m, 3H, NCH_2_CH_2_, CH(CH_3_)_2_), 3.23–3.30 (m, 6H, SCH_2_, N(CH_2_)_2_), 3.36 (s, 2H, NCH_2_), 3.62 (q, *J =* 6.3 Hz, 2H, SCH_2_CH_2_), 4.58 (t, *J =* 5.7 Hz, 1H, OH). ^13^C NMR (75 MHz, DMSO-*d*_6_/CCl_4_, 1/3) δ 18.10, 23.94, 25.43, 28.56, 31.73, 44.57, 48.24, 49.60, 53.19, 60.13, 97.67, 114.85, 116.76, 148.63, 157.47, 159.80. Anal. calcd. for C_19_H_28_N_4_OS: C 63.30; H 7.83; N 15.54%. Found: C 63.62; H 7.98; N 15.78%.

7-Benzyl-3-[(2-hydroxyethyl)thio]-1-pyrrolidin-1-yl-5,6,7,8-tetrahydro-2,7-naphthyridine-4-carbonitrile (**5d**). Light yellow solid; yield 87%, mp 163–165 °C. ^1^H NMR (300 MHz, DMSO-*d*_6_/CCl_4_, 1/3) δ 1.88–1.96 (m, 4H, C_4_H_8_N), 2.67 (t, *J* = 5.8 Hz, 2H, NCH_2_CH_2_), 2.80 (t, *J* = 5.8 Hz, 2H, NCH_2_CH_2_), 3.22 (t, *J =* 6.8 Hz, 2H, SCH_2_), 3.52 (s, 2H, NCH_2_), 3.55–3.64 (m, 6H, N(CH_2_)_2_, SCH_2_CH_2_), 3.65 (s, 2H, NCH_2_Ph), 4.55 (t, *J =* 5.7 Hz, 1H, OH), 7.18–7.32 (m, 5H, Ph). ^13^C NMR (75 MHz, DMSO-*d*_6_/CCl_4_, 1/3) δ 24.93, 28.13, 31.55, 47.98, 49.27, 53.04, 60.27, 61.60, 93.94, 111.49, 115.53, 126.59, 127.70, 128.16, 137.44, 147.16, 156.41, 157.51. Anal. calcd. for C_22_H_26_N_4_OS: C 66.97; H 6.64; N 14.20%. Found: C 67.34; H 6.82; N 14.46%.

7-Benzyl-3-[(2-hydroxyethyl)thio]-1-piperidin-1-yl-5,6,7,8-tetrahydro-2,7-naphthyridine-4-carbonitrile (**5e**). Colorless solid; yield 83%, mp 148–150 °C; IR *ν*/cm^–1^: 2209 (C≡N), 3194 (OH). ^1^H NMR (300 MHz, DMSO-*d*_6_/CCl_4_, 1/3) δ 1.56–1.65 (m, 6H, C_5_H_10_N), 2.71 (t, *J* = 6.0 Hz, 2H, NCH_2_CH_2_), 2.86 (t, *J* = 6.0 Hz, 2H, NCH_2_CH_2_), 3.20–3.29 (m, 6H, N(CH_2_)_2_, SCH_2_), 3.31 (s, 2H, NCH_2_), 3.62 (q, *J =* 6.5 Hz, 2H, SCH_2_CH_2_), 3.65 (s, 2H, NCH_2_Ph), 4.57 (t, *J =* 5.6 Hz, 1H, OH), 7.19–7.32 (m, 5H, Ph). ^13^C NMR (75 MHz, DMSO-*d*_6_/CCl_4_, 1/3) δ 23.89, 25.32, 27.93, 31.73, 48.52, 49.48, 52.57, 60.12, 61.56, 97.52, 114.81, 115.85, 126.60, 127.65, 128.25, 137.32, 148.23, 157.75, 159.63. Anal. calcd. for C_23_H_28_N_4_OS: C 67.61; H 6.91; N 13.71%. Found: C 67.28; H 6.78; N 13.50%.

1-Azepan-1-yl-7-benzyl-3-[(2-hydroxyethyl)thio]-5,6,7,8-tetrahydro-2,7-naphthyridine-4-carbonitrile (**5f**). Light yellow solid; yield 79%, mp 134–136 °C; 2204 (C≡N), 3209 (OH). ^1^H NMR (300 MHz, DMSO-*d*_6_/CCl_4_, 1/3) δ 1.47–1.57 (m, 4H, C_6_H_12_N), 1.67–1.78 (m, 4H, C_6_H_12_N), 2.70 (t, *J* = 5.8 Hz, 2H, NCH_2_CH_2_), 2.85 (t, *J* = 5.8 Hz, 2H, NCH_2_CH_2_), 3.22 (t, *J =* 6.8 Hz, 2H, SCH_2_), 3.32 (s, 2H, NCH_2_), 3.51–3.57 (m, 4H, N(CH_2_)_2_), 3.60 (q, *J =* 6.8 Hz, 2H, SCH_2_CH_2_), 3.65 (s, 2H, NCH_2_Ph), 4.56 (t, *J =* 5.6 Hz, 1H, OH), 7.18–7.32 (m, 5H, Ph). ^13^C NMR (75 MHz, DMSO-*d*_6_/CCl_4_, 1/3) δ 26.26, 28.05, 28.31, 31.56, 48.40, 50.60, 60.21, 61.71, 95.16, 112.68, 115.32, 126.61, 127.69, 128.22, 137.41, 147.99, 152.47, 157.09, 158.67. Anal. calcd. for C_24_H_30_N_4_OS: C 68.21; H 7.16; N 13.26%. Found: C 67.86; H 7.02; N 13.49%.

### 3.5. General Procedure for the Synthesis of Compounds **6b,d**–**f**

Sodium hydroxide (4 g, 100 mmol) was dissolved in 10 mL of water and added to a solution of compound **5** (10 mmol) in absolute ethanol (50 mL), and the mixture was refluxed for 15 h. After cooling, water was added, the mixture was filtered off, and the filtrate was neutralized with HCl. The formed crystals were filtered off, washed with water, and recrystallized from ethanol.

7-Isopropyl-3-oxo-1-piperidin-1-yl-2,3,5,6,7,8-hexahydro-2,7-naphthyridine-4-carbonitrile (**6b**). Colorless solid; yield 77%, mp 233–235 °C. ^1^H NMR (300 MHz, DMSO-*d*_6_/CCl_4_, 1/3) δ 1.09 (d, *J =* 6.5 Hz, 6H, CH(CH_3_)_2_), 1.63–1.69 (m, 6H, C_5_H_110_N), 2.68–2.89 (m, 5H, NCH_2_CH_2_, CH(CH_3_)_2_), 3.14–3.20 (m, 4H, N(CH_2_)_2_), 3.32 (s, 2H, NCH_2_), 11.20 (br, 1H, NH). ^13^C NMR (75 MHz, DMSO-*d*_6_/CCl_4_, 1/3) δ 16.58, 23.69, 25.23, 25.39, 43.68, 45.66, 49.98, 56.11, 85.93, 102.95, 115.16, 149.82, 158.46, 162.13. Anal. calcd. for C_17_H_24_N_4_O: C 67.97; H 8.05; N 18.65%. Found: C 68.29; H 8.21; N 18.87%.

7-Benzyl-3-oxo-1-pyrrolidin-1-yl-2,3,5,6,7,8-hexahydro-2,7-naphthyridine-4-carbonitrile (**6d**). Colorless solid; yield 74%, mp 228–230 °C; IR *ν*/cm^–1^: 1630 (C=O), 2197 (C≡N), 3114, 3236 (NH). ^1^H NMR (300 MHz, DMSO-*d*_6_/CCl_4_, 1/3) δ 1.85–1.95 (m, 4H, C_4_H_8_N), 2.64 (t, *J* = 6.0 Hz, 2H, NCH_2_CH_2_), 2.78 (t, *J* = 6.0 Hz, 2H, NCH_2_CH_2_), 3.45 (s, 2H, NCH_2_), 3.47–3.55 (m, 4H, N(CH_2_)_2_), 3.64 (s, 2H, NCH_2_Ph), 7.17–7.34 (m, 5H, Ph), 10.70 (br, 1H, NH). ^13^C NMR (75 MHz, DMSO-*d*_6_/CCl_4_, 1/3) δ 24.95, 28.55, 47.99, 49.61, 53.01, 61.54, 82.26, 116.33, 126.62, 127.72, 128.28, 137.37, 137.40, 151.23, 151.25, 154.80, 154.83, 161.21. Anal. calcd. for C_20_H_22_N_4_O: C 71.83; H 6.63; N 16.75%. Found: C 71.49; H 6.78; N 16.50%.

7-Benzyl-3-oxo-1-piperidin-1-yl-2,3,5,6,7,8-hexahydro-2,7-naphthyridine-4-carbonitrile (**6e**). Light yellow solid; yield 71%, mp 226–228 °C; IR *ν*/cm^–1^: 1638 (C=O), 2212 (C≡N), 3087, 3228 (NH). ^1^H NMR (300 MHz, DMSO-*d*_6_/CCl_4_, 1/3) δ 1.50–1.62 (m, 6H, C_5_H_10_N), 2.68 (t, *J* = 6.0 Hz, 2H, NCH_2_CH_2_), 2.84 (t, *J* = 6.0 Hz, 2H, NCH_2_CH_2_), 3.10–3.15 (m, 4H, N(CH_2_)_2_), 3.22 (s, 2H, NCH_2_), 3.63 (s, 2H, NCH_2_Ph), 7.18–7.31 (m, 5H, Ph), 11.23 (br, 1H, NH). ^13^C NMR (75 MHz, DMSO-*d*_6_/CCl_4_, 1/3) δ 23.64, 25.29, 28.36, 48.73, 49.59, 52.05, 61.56, 115.53, 126.60, 127.37, 127.65, 128.35, 129.30, 137.4, 152.70, 157.2, 161.36. Anal. calcd. for C_21_H_24_N_4_O: C 72.39; H 6.94; N 16.08%. Found: C 72.77; H 7.11; N 16.32%.

1-Azepan-1-yl-7-benzyl-3-oxo-2,3,5,6,7,8-hexahydro-2,7-naphthyridine-4-carbonitrile (**6f**). Yellow solid; yield 75%, mp 227–229 °C; IR *ν*/cm^–1^: 1629 (C=O), 2203 (C≡N), 3107, 3235 (NH). ^1^H NMR (300 MHz, DMSO-*d*_6_/CCl_4_, 1/3) δ 1.46–1.56 (m, 4H, C_6_H_12_N), 1.62–1.71 (m, 4H, C_6_H_12_N), 2.68 (t, *J* = 6.0 Hz, 2H, NCH_2_CH_2_), 2.84 (t, *J* = 6.0 Hz, 2H, NCH_2_CH_2_), 3.25 (s, 2H, NCH_2_), 3.39–3.45 (m, 4H, N(CH_2_)_2_), 3.63 (s, 2H, NCH_2_Ph), 7.17–7.33 (m, 5H, Ph), 10.90 (br, 1H, NH). ^13^C NMR (75 MHz, DMSO-*d*_6_/CCl_4_, 1/3) δ 26.29, 28.00, 28.60, 48.66, 51.11, 53.36, 61.79, 107.65, 115.75, 126.56, 127.67, 128.26, 137.55, 151.39, 157.98, 161.18. Anal. calcd. for C_22_H_26_N_4_O: C 72.90; H 7.23; N 15.46%. Found: C 72.51; H 7.05; N 15.22%. ESI HRMS [C_22_H_26_N_4_O+H^+^] Calculated: 363.2185. Found: 363.2195.

### 3.6. General Procedure for the Synthesis of Compounds **7a**–**f**

A mixture of compound **6** (1 mmol) and of the corresponding amine (5 mL) was refluxed for 5 h. The reaction mixture was cooled, and water (50 mL) was added. Separated crystals were filtered off, washed with water, dried, and recrystallized from a mixture of ethanol–water (2:1).

8-[(2-Hydroxyethyl)imino]-7-isopropyl-3-pyrrolidin-1-yl-5,6,7,8-tetrahydro-2,7-naphthyridin-1-ol (**7a**). Colorless solid; yield 64%, mp 257–259 °C; IR *ν*/cm^–1^: 1576 (C=N), 3179 (OH). ^1^H NMR (300 MHz, DMSO-*d*_6_/CCl_4_, 1/3) δ 1.30 (d, *J =* 6.5 Hz, 6H, CH(CH_3_)_2_), 1.88–1.99 (m, 4H, C_4_H_8_N), 2.61 (t, *J =* 6.0 Hz, 2H, NCH_2_CH_2_), 3.32 (t, *J =* 6.0 Hz, 2H, NCH_2_CH_2_), 3.34–3.45 (m, 6H, CH_2_OH, N(CH_2_)_2_), 3.62–3.69 (m, 2H, NCH_2_), 4.13 (sp, *J =* 6.5 Hz, 1H, CH(CH_3_)_2_), 4.97 (br, 1H, CH_2_OH), 5.38 (s, 1H, 4-CH), 13.24 (br, 1H, 1-OH). ^13^C NMR (75 MHz, DMSO-*d*_6_/CCl_4_, 1/3) δ 20.34, 24.79, 28.73, 38.95, 45.98, 48.70, 50.90, 60.15, 93.53, 93.62, 148.06, 158.26, 163.55, 170.95. Anal. calcd. for C_17_H_26_N_4_O_2_: C 64.12; H 8.23; N 17.60%. Found: C 64.44; H 8.05; N 17.34%. ESI HRMS [C_17_H_26_N_4_O_2_+H^+^] Calculated: 319.2134. Found: 319.2154.

8-[(2-Hydroxypropyl)imino]-7-isopropyl-3-pyrrolidin-1-yl-5,6,7,8-tetrahydro-2,7-naphthyridin-1-ol (**7b**). Cream-colored solid; yield 60%, mp 207–209 °C; IR *ν*/cm^–1^: 1579 (C=N), 3186 3430 (OH). ^1^H NMR (300 MHz, DMSO-*d*_6_/CCl_4_, 1/3) δ 1.18 (d, *J =* 6.2 Hz, 3H, CHCH_3_), 1.31 (d, *J =* 6.6 Hz, 6H, CH(CH_3_)_2_), 1.91–2.01 (m, 4H, C_4_H_8_N), 2.62 (t, *J =* 6.1 Hz, 2H, NCH_2_CH_2_), 3.23 (t, *J =* 5.1 Hz, 2H, CH_2_CH), 3.33 (t, *J =* 6.1 Hz, 2H, NCH_2_CH_2_), 3.39–3.48 (m, 4H, N(CH_2_)_2_), 3.82–3.94 (m, 1H, CHCH_3_), 4.12 (sp, *J =* 6.5 Hz, 1H, CH(CH_3_)_2_), 4.91 (br, 1H, CHOH), 5.37 (s, 1H, 4-CH), 13.13 (br, 1H, 1-OH). ^13^C NMR (75 MHz, DMSO-*d*_6_/CCl_4_, 1/3) δ 20.28, 20.56, 24.75, 28.73, 46.02, 50.88, 53.90, 65.21, 93.38, 93.59, 148.22, 157.81, 163.48, 170.39. Anal. calcd. for C_18_H_28_N_4_O_2_: C 65.03; H 8.49; N 16.85%. Found: C 64.66; H 8.33; N 16.59%. ESI HRMS [C_18_H_28_N_4_O_2_+H^+^] Calculated: 333.2291. Found: 333.2294.

8-[(2-Hydroxypropyl)imino]-7-isopropyl-3-piperidin-1-yl-5,6,7,8-tetrahydro-2,7-naphthyridin-1-ol (**7c**). Cream-colored solid; yield 59%, mp 172–174 °C; IR *ν*/cm^–1^: 1575 (C=N), 3210 (OH). ^1^H NMR (300 MHz, DMSO-*d*_6_/CCl_4_, 1/3) δ 1.19 (d, *J =* 6.2 Hz, 3H, CHCH_3_), 1.30 (d, *J =* 6.5 Hz, 6H, CH(CH_3_)_2_), 1.51–1.1.71 (m, 6H, C_5_H_10_N), 2.61 (t, *J =* 6.1 Hz, 2H, NCH_2_CH_2_), 3.23 (t, *J =* 4.8 Hz, 2H, CH_2_CH), 3.32 (t, *J =* 6.2 Hz, 2H, NCH_2_CH_2_), 3.53–3.62 (m, 4H, N(CH_2_)_2_), 3.82–3.94 (m, 1H, CHCH_3_), 4.12 (sp, *J =* 6.5 Hz, 1H, CH(CH_3_)_2_), 4.89 (br, 1H, CHOH), 5.60 (s, 1H, 4-CH), 13.26 (br, 1H, 1-OH). ^13^C NMR (75 MHz, DMSO-*d*_6_/CCl_4_, 1/3) δ 20.29, 20.58, 24.41, 25.30, 28.88, 38.90, 44.82, 50.82, 53.87, 65.25, 92.42, 93.67, 148.22, 159.59, 163.32, 170.97. Anal. calcd. for C_19_H_30_N_4_O_2_: C 65.87; H 8.73; N 16.17%. Found: C 65.54; H 8.88; N 15.92%. ESI HRMS [C_19_H_30_N_4_O_2_+H^+^] Calculated: 347.2447. Found: 347.2470.

3-Azepan-1-yl-8-[(2-hydroxypropyl)imino]-7-isopropyl-5,6,7,8-tetrahydro-2,7-naphthyridin-1-ol (**7d**). Cream-colored solid; yield 61%, mp 205–207 °C; IR *ν*/cm^–1^: 1567 (C=N), 3283 (OH). ^1^H NMR (300 MHz, DMSO-*d*_6_/CCl_4_, 1/3) δ 1.19 (d, *J =* 6.2 Hz, 3H, CHCH_3_), 1.30 (d, *J =* 6.5 Hz, 6H, CH(CH_3_)_2_), 1.49–1.58 (m, 4H, C_6_H_12_N), 1.69–1.79 (m, 4H, C_6_H_12_N), 2.60 (t, *J =* 6.2 Hz, 2H, NCH_2_CH_2_), 3.21 (t, *J =* 5.2 Hz, 2H, CH_2_CH), 3.32 (t, *J =* 6.3 Hz, 2H, NCH_2_CH_2_), 3.55–3.64 (m, 4H, N(CH_2_)_2_), 3.82–3.93 (m, 1H, CHCH_3_), 4.10 (sp, *J =* 6.5 Hz, 1H, CH(CH_3_)_2_), 4.88 (br, 1H, CHOH), 5.48 (s, 1H, 4-CH), 13.33 (br, 1H, 1-OH). ^13^C NMR (75 MHz, DMSO-*d*_6_/CCl_4_, 1/3) δ 20.28, 20.30, 20.58, 26.31, 27.45, 28.90, 46.87, 50.86, 53.91, 65.27, 92.16, 93.56, 148.07, 159.31, 163.55, 171.03. Anal. calcd. for C_20_H_32_N_4_O_2_: C 66.63; H 8.95; N 15.54%. Found: C 66.98; H 9.12; N 15.31%. ESI HRMS [C_20_H_32_N_4_O_2_+H^+^] Calculated: 361.2603. Found: 361.2611.

7-Benzyl-8-[(2-hydroxypropyl)imino]-3-pyrrolidin-1-yl-5,6,7,8-tetrahydro-2,7-naphthyridin-1-ol (**7e**). Light yellow solid; yield 65%, mp 237–239 °C; IR *ν*/cm^–1^: 1571 (C=N), 3186 (OH). ^1^H NMR (300 MHz, DMSO-*d*_6_/CCl_4_, 1/3) δ 1.09 (d, *J =* 6.2 Hz, 3H, CHCH_3_), 1.90–2.00 (m, 4H, C_4_H_8_N), 2.53 (t, *J =* 6.3 Hz, 2H, NCH_2_CH_2_), 3.21–3.27 (m, 2H, CH_2_CH), 3.33 (t, *J =* 6.3 Hz, 2H, NCH_2_CH_2_), 3.39–3.48 (m, 4H, N(CH_2_)_2_), 3.80–3.91 (m, 1H, CHCH_3_), 4.60–4.75 (m, 2H, CH_2_Ph), 4.91 (br, 1H, CHOH), 5.35 (s, 1H, 4-CH), 7.25–7.41 (m, 5H, Ph), 13.74 (br, 1H, 1-OH). ^13^C NMR (75 MHz, DMSO-*d*_6_/CCl_4_, 1/3) δ 20.55, 24.72, 27.13, 46.25, 46.57, 52.51, 55.22, 65.13, 93.13, 93.37, 126.53, 127.08, 128.33, 136.55, 148.78, 157.13, 162.99, 169.45. Anal. calcd. for C_22_H_28_N_4_O_2_: C 69.45; H 7.42; N 14.73%. Found: C 69.83; H 7.56; N 14.95%. ESI HRMS [C_22_H_28_N_4_O_2_+H^+^] Calculated: 381.2291. Found: 381.2292.

7-Benzyl-8-[(2-hydroxypropyl)imino]-3-piperidin-1-yl-5,6,7,8-tetrahydro-2,7-naphthyridin-1-ol (**7f**). Cream-colored solid; yield 63%, mp 218–220 °C; IR *ν*/cm^–1^: 1575 (C=N), 3151, 3413 (OH). ^1^H NMR (300 MHz, DMSO-*d*_6_/CCl_4_, 1/3) δ 1.09 (d, *J =* 6.2 Hz, 3H, CHCH_3_), 1.50–1.71 (m, 6H, C_5_H_10_N), 2.53 (t, *J =* 6.3 Hz, 2H, NCH_2_CH_2_), 3.21–3.29 (m, 2H, CH_2_CH), 3.33 (t, *J =* 6.0 Hz, 2H, NCH_2_CH_2_), 3.53–3.62 (m, 4H, N(CH_2_)_2_), 3.78–3.90 (m, 1H, CHCH_3_), 4.61–4.77 (m, 2H, CH_2_Ph), 4.86 (br, 1H, CHOH), 5.61 (s, 1H, 4-CH), 7.24–7.41 (m, 5H, Ph), 13.64 (br, 1H, 1-OH). ^13^C NMR (75 MHz, DMSO-*d*_6_/CCl_4_, 1/3) δ 20.60, 24.40, 25.35, 27.33, 45.02, 46.63, 52.57, 55.23, 65.25, 92.37, 93.54, 126.58, 127.08, 128.34, 136.64, 148.52, 159.31, 162.90, 170.47. Anal. calcd. for C_23_H_30_N_4_O_2_: C 70.02; H 7.66; N 14.20%. Found: C 69.66; H 7.85; N 13.96%. ESI HRMS [C_23_H_30_N_4_O_2_+H^+^] Calculated: 395.2447. Found: 395.2444.

### 3.7. Procedures for the Synthesis of Compound **7g**

***A***. The same method for the synthesis of compounds **7a**–**f**.

***B***. A mixture of compound **8** (1 mmol) and of the 1-aminopropan-2-ol in (5 mL) was refluxed for 3 h. The reaction mixture was cooled, and water (50 mL) was added. Separated crystals were filtered off, washed with water, dried, and recrystallized from a mixture of ethanol–water (2:1).

3-Azepan-1-yl-7-benzyl-8-[(2-hydroxypropyl)imino]-5,6,7,8-tetrahydro-2,7-naphthyridin-1-ol (**7g**). Cream-colored solid; yield 68%/57%, mp 238–240 °C; IR *ν*/cm^–1^: 1573 (C=N), 3183, 3454 (OH). ^1^H NMR (300 MHz, DMSO-*d*_6_/CCl_4_, 1/3) δ 1.10 (d, *J =* 6.2 Hz, 3H, CHCH_3_), 1.50–1.58 (m, 4H, C_6_H_12_N), 1.70–1.80 (m, 4H, C_6_H_12_N), 2.53 (t, *J =* 6.4 Hz, 2H, NCH_2_CH_2_), 3.21–3.27 (m, 2H, CH_2_CH), 3.34 (t, *J =* 6.3 Hz, 2H, NCH_2_CH_2_), 3.55–3.67 (m, 4H, N(CH_2_)_2_), 3.80–3.90 (m, 1H, CHCH_3_), 4.61–4.75 (m, 2H, CH_2_Ph), 4.78 (d, *J =* 4.5 Hz, 1H, CHOH), 5.48 (s, 1H, 4-CH), 7.25–7.40 (m, 5H, Ph), 13.83 (br, 1H, 1-OH). ^13^C NMR (75 MHz, DMSO-*d*_6_/CCl_4_, 1/3) δ 20.56, 26.32, 27.21, 27.49, 46.44, 46.76, 52.54, 55.09, 65.29, 92.07, 93.38, 126.53, 126.98, 128.24, 136.66, 147.55, 159.72, 163.32, 169.99. Anal. calcd. for C_24_H_32_N_4_O_2_: C 70.56; H 7.90; N 13.71%. Found: C 70.20; H 7.74; N 13.44%. ESI HRMS [C_24_H_32_N_4_O_2_+H^+^] Calculated: 409.2603. Found: 409.2606.

### 3.8. Procedure for the Synthesis of Compound **8**

A mixture of compound **6f** (1 mmol) and of the 2-aminoethanol/1-aminopropan-2-ol (5 mL) was refluxed for 2 h. The reaction mixture was cooled, and water (50 mL) was added, and the separated crystals were filtered off, washed with water, dried, and recrystallized from a mixture of ethanol–water (2:1).

**6-Azepan-1-yl-2-benzyl-8-hydroxy-3,4-dihydro-2,7-naphthyridin-1**(**2*H***)**-one** (**8**). Cream-colored solid; yield 54%/58%, mp 137–139 °C; IR *ν*/cm^–1^: 1606, 1625 (C=O). ^1^H NMR (300 MHz, DMSO-*d*_6_/CCl_4_, 1/3) δ 1.51–1.60 (m, 4H, C_6_H_12_N), 1.72–1.82 (m, 4H, C_6_H_12_N), 2.80 (t, *J =* 6.7 Hz, 2H, NCH_2_CH_2_), 3.41 (t, *J =* 6.7 Hz, 2H, NCH_2_CH_2_), 3.57–3.69 (m, 4H, N(CH_2_)_2_), 4.65 (s, 2H, CH_2_Ph), 5.82 (s, 1H, 5-CH), 7.19–7.35 (m, 5H, Ph), 13.11 (s, 1H, 1-OH). ^13^C NMR (75 MHz, DMSO-*d*_6_/CCl_4_, 1/3) δ 26.26, 27.01, 27.24, 44.16, 47.08, 48.82, 94.10, 94.59, 126.73, 127.45, 127.94, 136.81, 148.79, 158.78, 165.17, 166.93. Anal. calcd. for C_21_H_25_N_3_O_2_: C 71.77; H 7.17; N 11.96%. Found: C 71.41; H 7.03; N 11.76%. ESI HRMS [C_21_H_25_N_3_O_2_+H^+^] Calculated: 352.2025. Found: 352.2032.

### 3.9. Computational Details

*Conformational Analysis*. All compounds were modeled using the GAFF force field [26]. The atomic charges were determined using the Merz–Singh–Kollman scheme [27]. The corresponding parameters were generated by the standard procedure that is reported for an antechamber [28], as implemented in AMBER 23 [29]. A total of 2500 steps of a steepest descent minimization, followed by an additional 7500 steps of conjugate gradient minimization, were performed with SANDER, distributed with the AMBER [29] package.

To sample the conformational space, a quenched molecular dynamics approach was used. A total of 10 ns of high-temperature MD simulations was carried out for each compound. H atoms were considered using the SHAKE algorithm [30], and the Langevin temperature equilibration scheme was used at 500 K. The conformational landscape of the molecules is visualized onto a two-dimensional space formed by the PCA1/PCA2 axes [31,32]. These coordinates are derived from a principal component analysis (PCA). The program “ptraj” in the AMBER package [29] was used in the PCA.

A total of 100 snapshots per simulation were minimized following the same minimization protocol described previously. The lowest energy-minimized snap was used for QM calculations.

*QM Calculations*. All reported DFT computations were performed with the Gaussian 16 series of programs [33] using the M06-2X functional [34] and the 6-311++G** basis set [35]. The geometries of the various critical points on the potential energy surface were fully optimized with the gradient method available in Gaussian 16, and harmonic vibrational frequencies were computed to evaluate the nature of all critical points. The energy profile for rotation around the C-OH bond in 1-amino-3-oxo-2,7-naphthyridines was carried out following reference [36]. ESP charges of the optimized structures were determined as implemented in Gaussian 16 [33] according to a population analysis following the Merz–Singh–Kollman scheme [27,37].

## 4. Conclusions

In this paper, a new class of 1,3-diamino-2,7-naphthyridines was synthesized to study their rearrangement reaction. It was found that by replacing the methyl/benzyl groups in the piperidine ring of the 2,7-naphthyridine system with the isopropyl and isobutyl groups, the rearrangement is more difficult, showing the following order of reactivity: Me < Bn < *i*-Pr ≈ *i*-Bu. It was also found that with a bulkier cyclic amine, the rearrangement reaction proceeded slightly slower, showing the following reactivity sequence: pyrrolidine < piperidine ≈ azepane. These facts can be related to the steric hindrance, the estimated quantitatively by the Taft’s steric parameter Es, or the molar volume of the substituents, caused by alkyl and amino groups during the rearrangement process.

With the aim of enlarging the scope of this rearrangement, the synthesis of 1-amino-3-oxo-2,7-naphthyridines **6** was performed. The reaction of these compounds with amines leads to the formation of Schiff base **7**, obtained by the addition of the amine to the carbonyl group of the intermediate rearrangement product. In this case, the rearrangement reaction is significantly faster than 1,3-diamino-2,7-naphthyridines and does not depend on the presence of groups at the 7th and 1st positions.

QM calculations provided the values of the ESP charges of the atoms involved in the nucleophilic attacks, suggesting the steps where electronic effects govern the observed reactivity, as in the case of the fast rearrangement of the 1-amino-3-oxo-2,7-naphthyridines. The increase in the positive charge on the cyano group in the 1-oxo-2,7-naphthyridines, compared to 1,3-diamino-2,7-naphthyridines, explains its higher velocity in the rearrangement process.

The expansion of the scope of the rearrangement showed in this paper allowed an improvement of the understanding of its reaction mechanism and provided a chance to develop a new regioselective process for the synthesis of 2,7-naphthyridine derivatives.

## Figures and Tables

**Table 1:** 1,3-Dimaino-2,7-naphthyridines **3** and 6,8-dimaino-2,7-naphthyridin-1-ones **4**.

Compound	R	Cyclic Amine	R^1^	Yield *, %
**3a/4a**	*i*-Pr	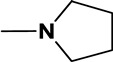	H	78/57
**3b/4b**	*i*-Pr	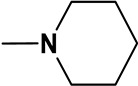	H	83/61
**3c/4c**	*i*-Pr	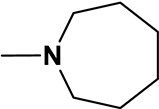	H	77/56
**3d/4d**	*i*-Pr	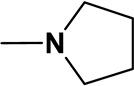	Me	81/60
**3e/4e**	i-Pr	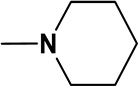	Me	76/55
**3f/4f**	*i*-Pr	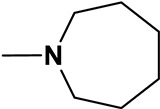	Me	85/58
**3g/4g**	*i*-Bu	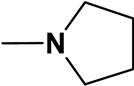	H	79/64
**3h/4h**	*i*-Bu	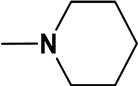	H	76/66
**3i/4i**	*i*-Bu	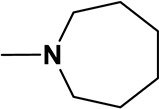	H	83/62
**3j/4j**	*i*-Bu	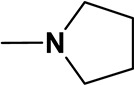	Me	74/59
**3k/4k**	*i*-Bu	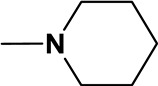	Me	80/67
**3l/4l**	*i*-Bu	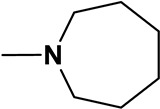	Me	86/65

* Yields after recrystallization.

**Table 2:** Rearrangement of products **7a**–**g**.

Compound	R	Cyclic Amine	R^1^	Yield *, %
**7a**	*i*-Pr	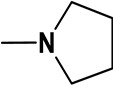	H	64
**7b**	*i*-Pr	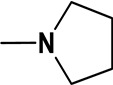	Me	60
**7c**	*i*-Pr	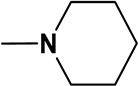	Me	59
**7d**	*i*-Pr	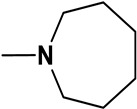	Me	61
**7e**	Bn	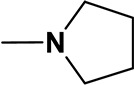	Me	65
**7f**	Bn	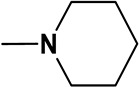	Me	63
**7g**	Bn	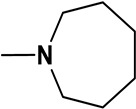	Me	68/57

* Yields after recrystallization.

## Data Availability

Not applicable Data are contained within the article and Supplementary Materials.

## Supplementary Materials

The following supporting information can be downloaded at https://www.mdpi.com/article/10.3390/ijms252211977/s1: the copies of ^1^H-NMR, ^13^C-NMR, and MS spectra for new synthesized compounds.
