# Supplementary material for: Synthesis and Rearrangement of New 1,3-Diamino-2,7-naphthyridines and 1-Amino-3-oxo-2,7-naphthyridines
Source: Int J Mol Sci. 2024 Nov 7;25(22):11977. doi: 10.3390/ijms252211977 (PMC11593454; doi:10.3390/ijms252211977)
Supplement: Supplementary file 1 [file ijms-25-11977-s001.zip › ijms-3284500-supplementary.pdf]

Supplementary Information File

Table of Contents

Figure S1. (Top) 3D optimized structures. (Middle) ESP charges on the different atoms. (Bottom) PCA conformational landscape generated in the MD simulations. Each point represents a unique conformation……………………………………………………………………………………………2

Figure S2. (Top) 3D optimized structures. (Middle) ESP charges on the different atoms. (Bottom) PCA conformational landscape generated in the MD simulations. Each point represents a unique conformation……………………………………………………………………………………………3

Figure S3. The energy profile for rotation around C-O bond of the most stable conformers of 3-oxo-2,7-naphthyridines derivatives……………………………………………………………………………4

Figure S4. (Top) 3D optimized structures. (Bottom) ESP charges on the different atoms…………………5

Figure S5. Simulated IR spectra from compounds **4**, **7** and **8**……………………………………………6

Figures S6–S11. Spectroscopic data for compounds **2b,d–f,h,i**………………………………………7

Figures S12–S23. Spectroscopic data for compounds **3a–l**……………………………………………13

Figures S24–S35. Spectroscopic data for compounds **4a–l**……………………………………………26

Figures S36–S39. Spectroscopic data for compounds **5b,d–f**…………………………………………42

Figures S40–S43. Spectroscopic data for compounds **6b,d–f**…………………………………………46

Figures S44–S50. Spectroscopic data for compounds **7a–f**……………………………………………50

Figure S51. Spectroscopic data for compound **8**………………………………………………………61

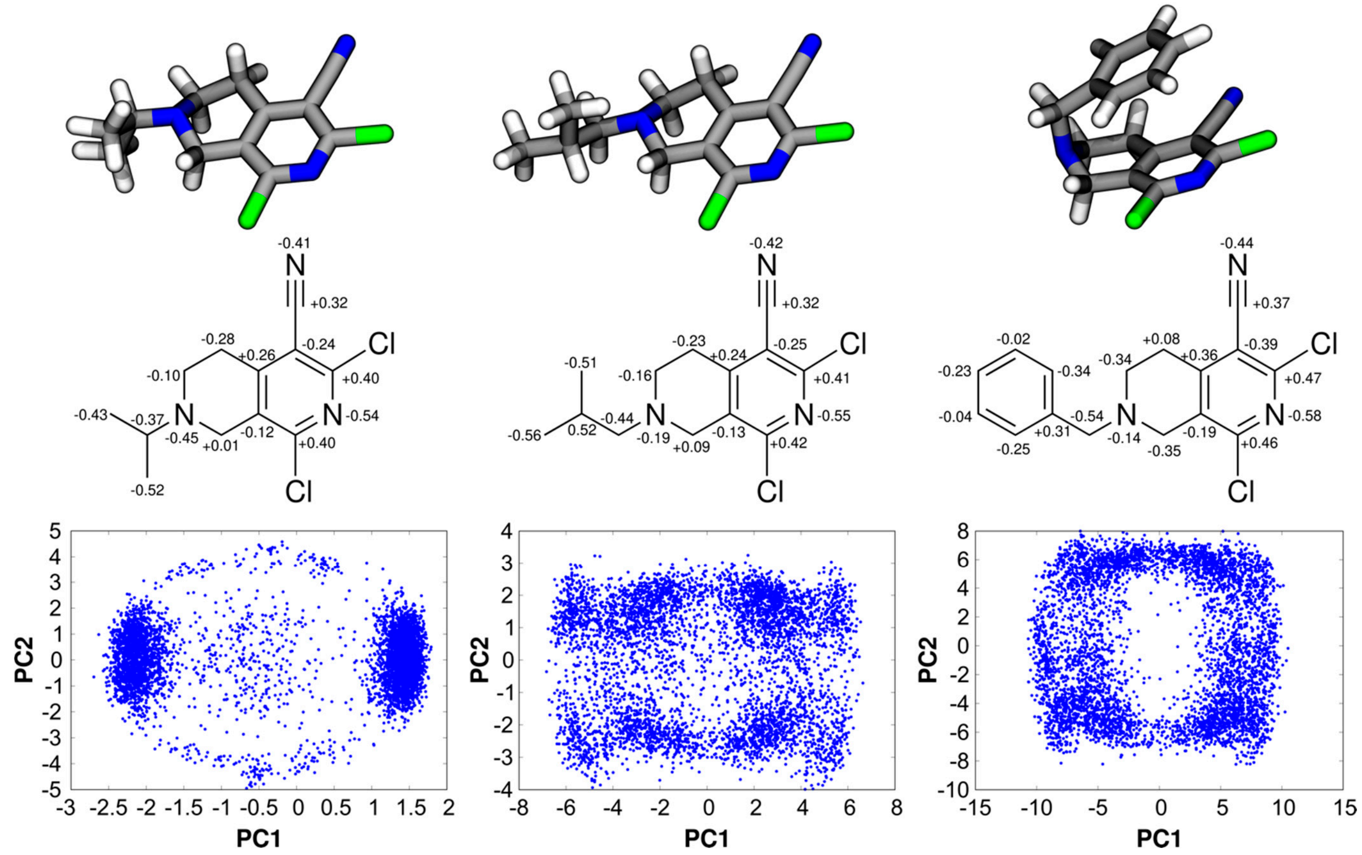

Figure S1. (Top) 3D optimized structures. (Middle) ESP charges on the different atoms. (Bottom) PCA conformational landscape generated in the MD simulations. Each point represents a unique conformation.

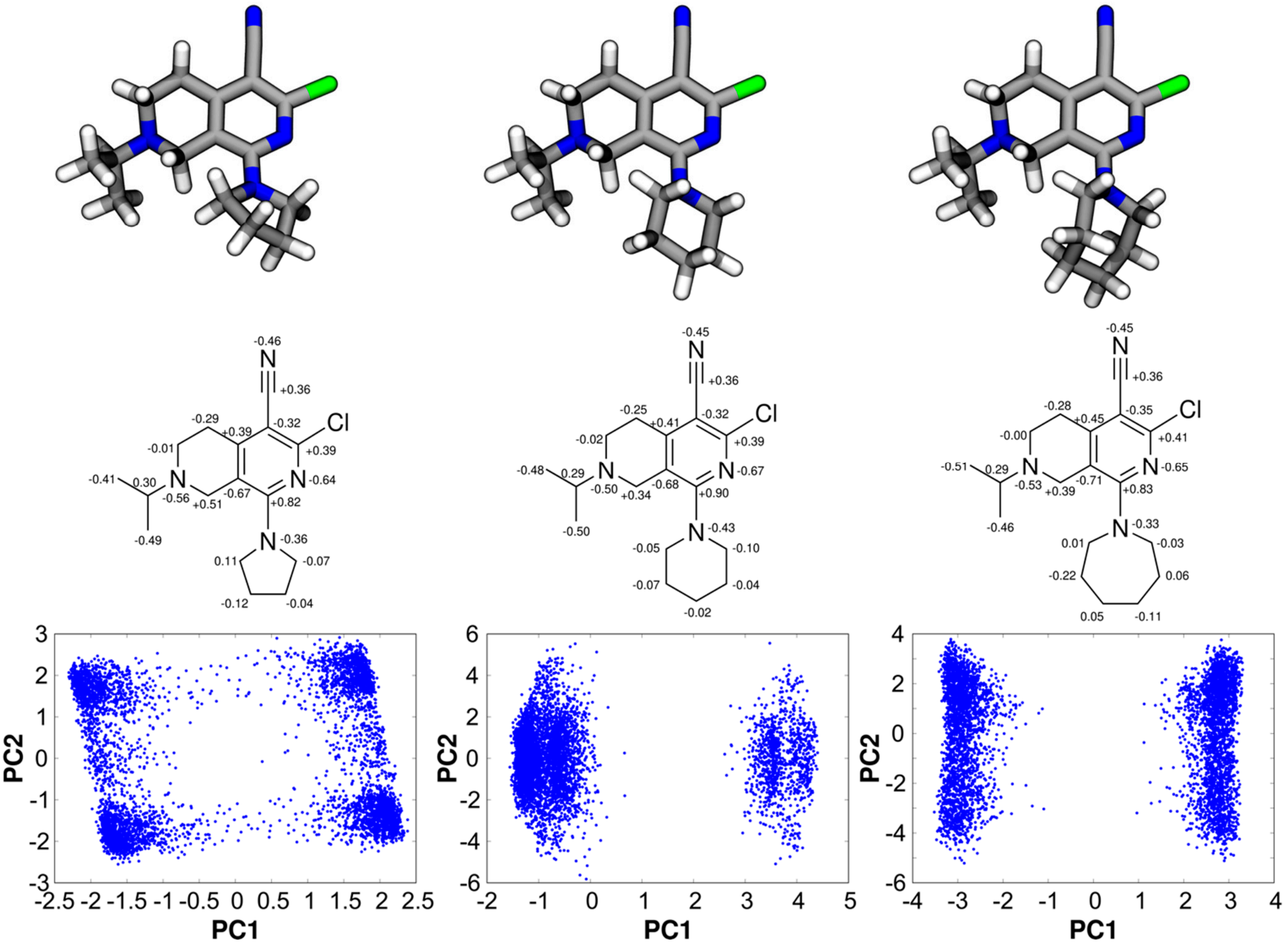

Figure S2. (Top) 3D optimized structures. (Middle) ESP charges on the different atoms. (Bottom) PCA conformational landscape generated in the MD simulations. Each point represents a unique conformation.

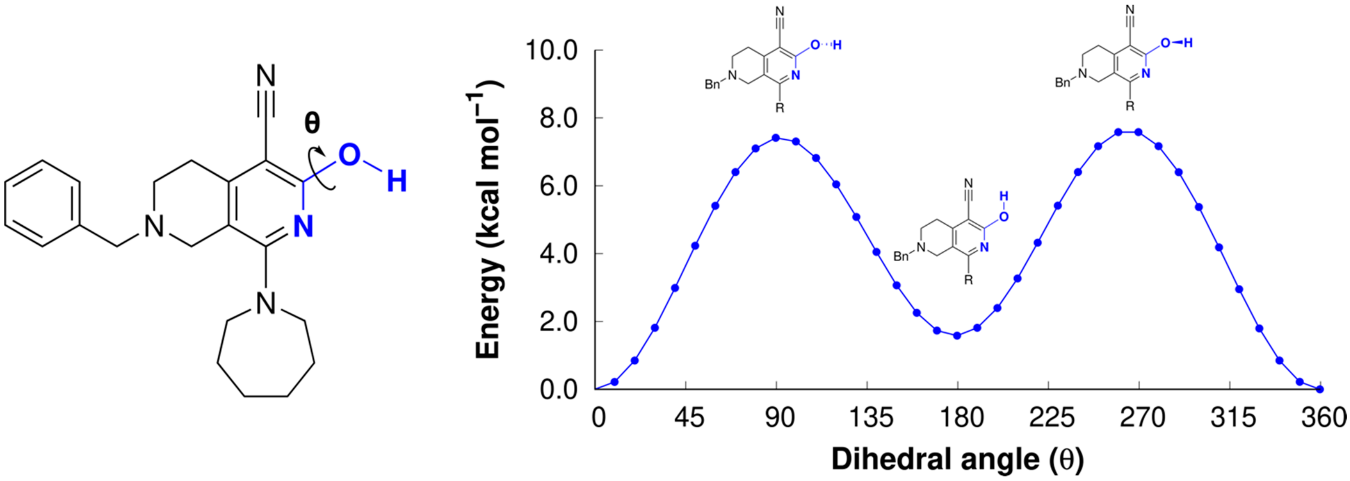

Figure S3. The energy profile for rotation around C-O bond of the most stable conformers of 3-oxo-2,7-naphthyridines derivatives.

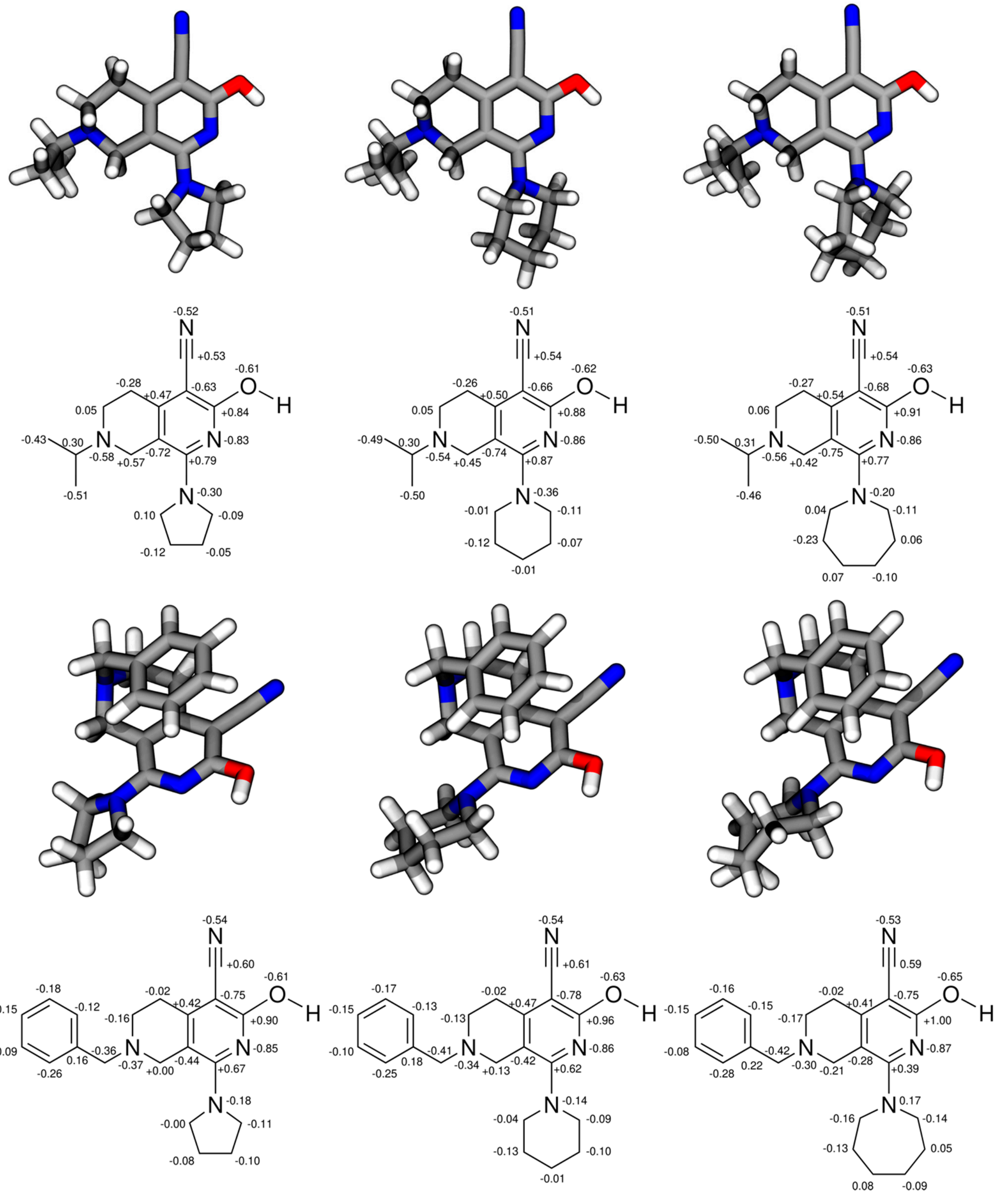

Figure S4. (Top) 3D optimized structures. (Bottom) ESP charges on the different atoms.

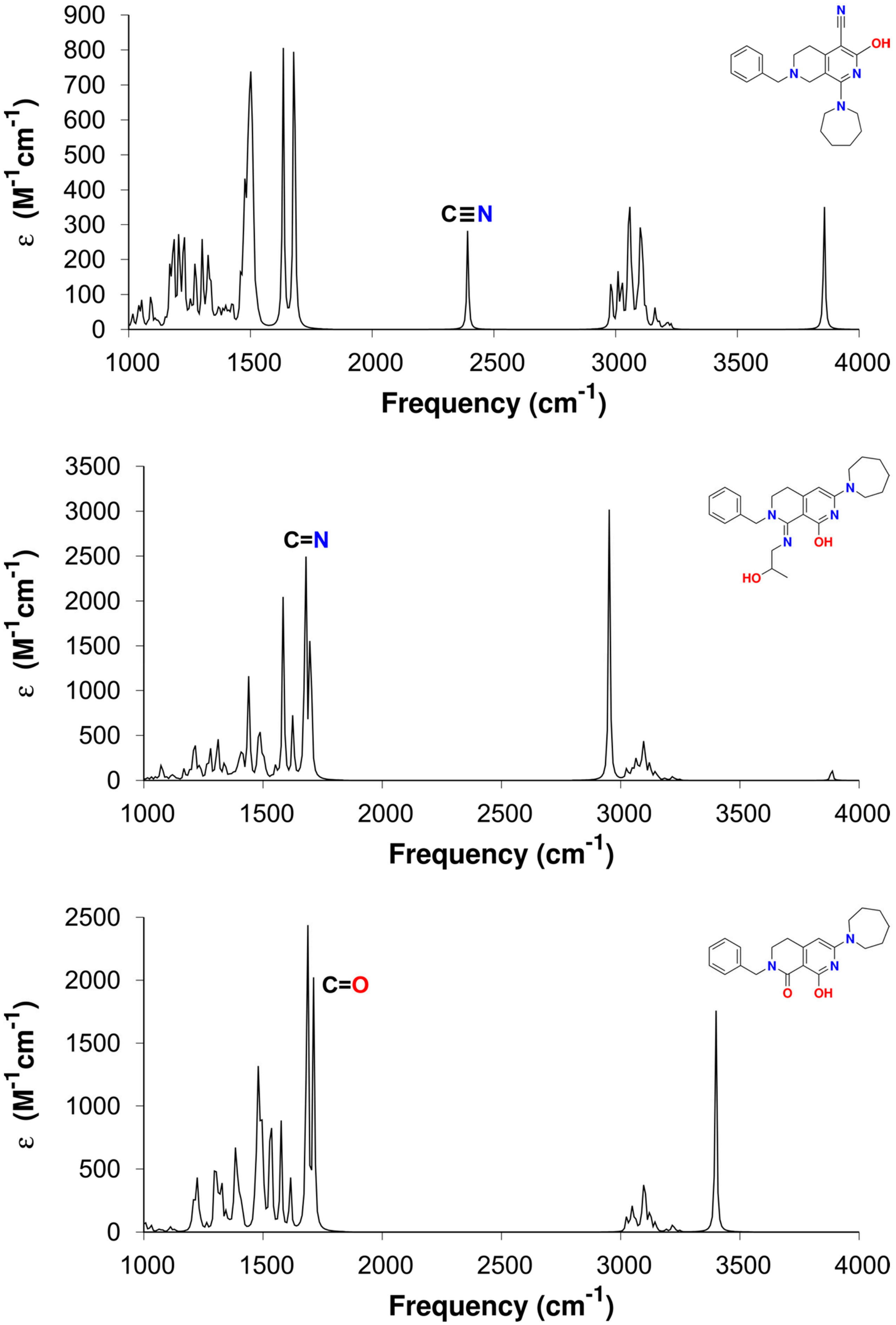

Figure S5. Simulated IR spectra from compounds **4**, **7** and **8**

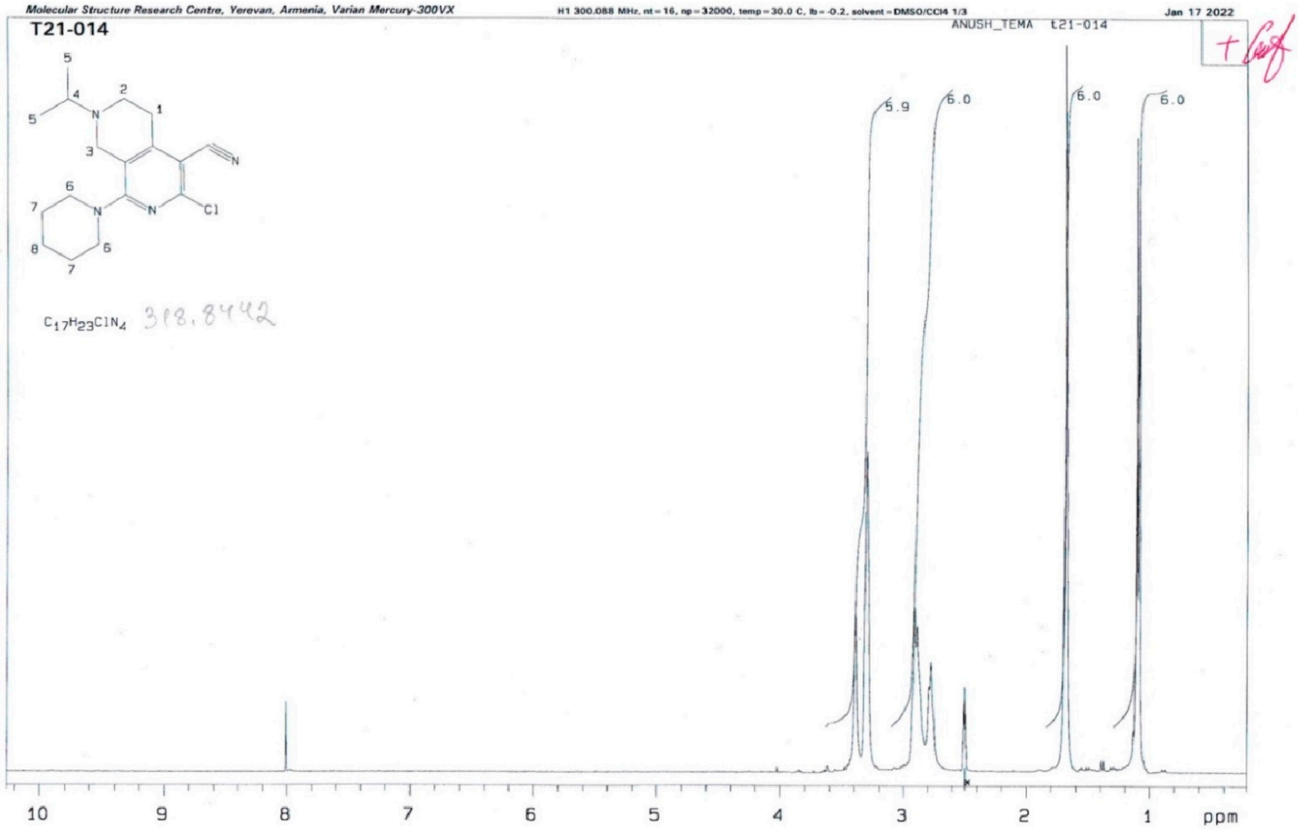

Figure S6. ¹H NMR spectrum of compound **2b**

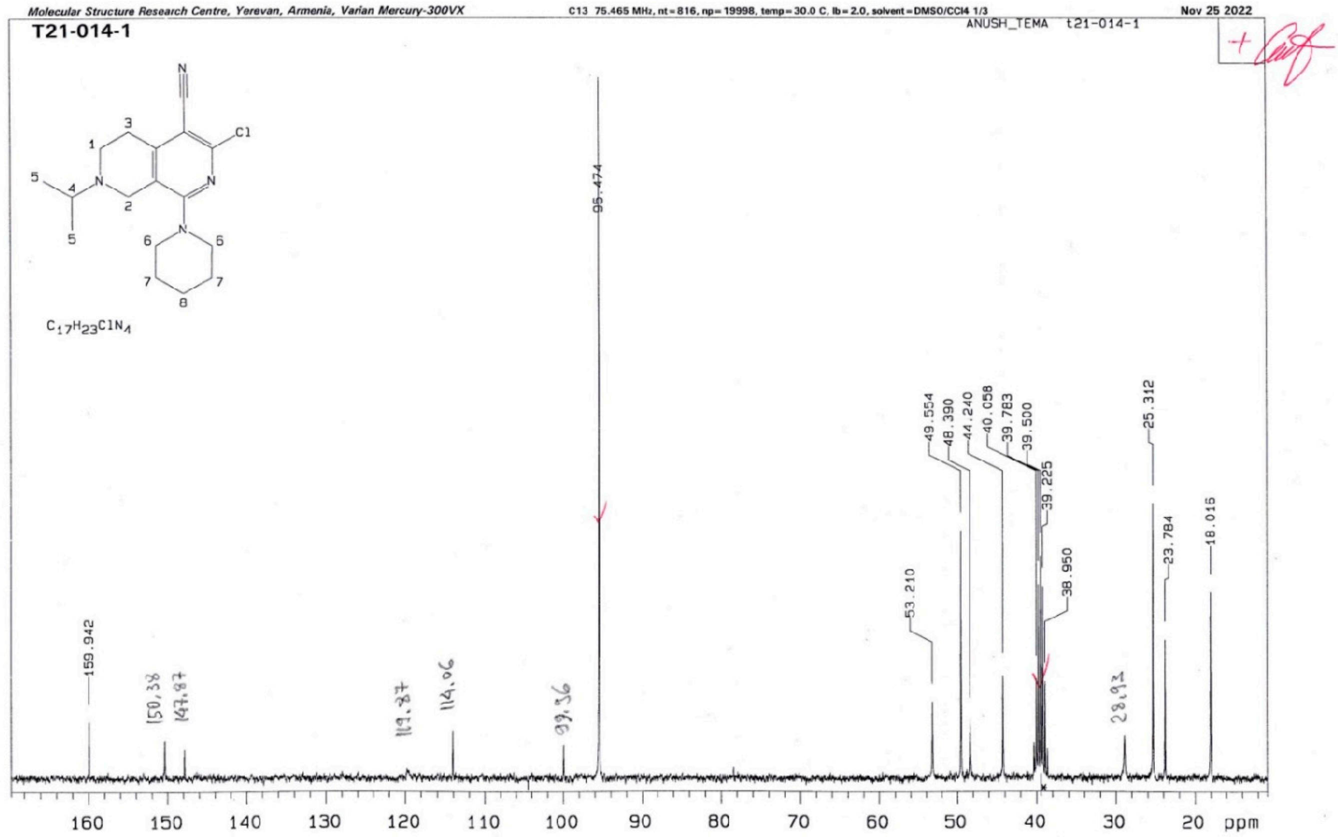

Figure S6.1. ¹³C NMR spectrum of compound **2b**

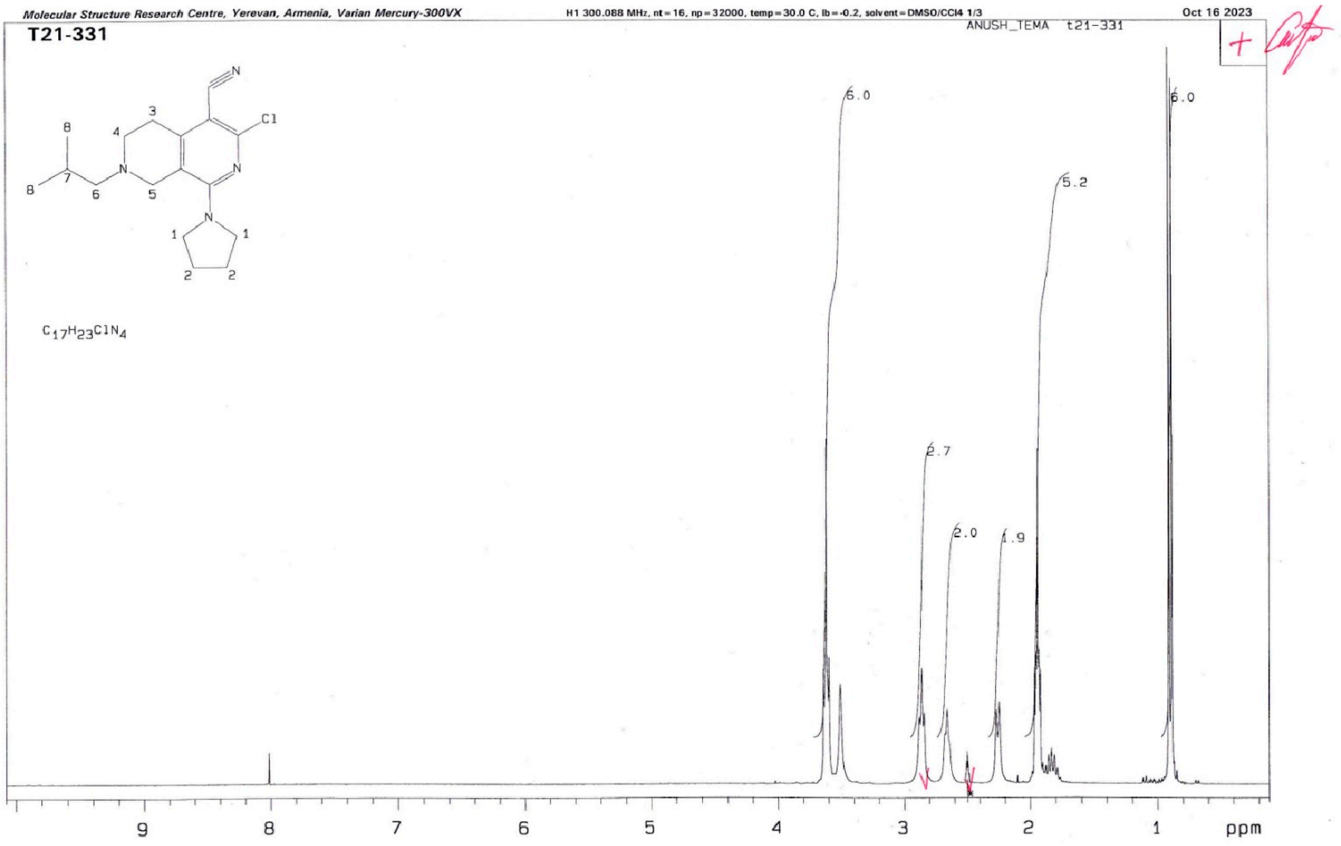

Figure S7. ^{1}H NMR spectrum of compound **2d**

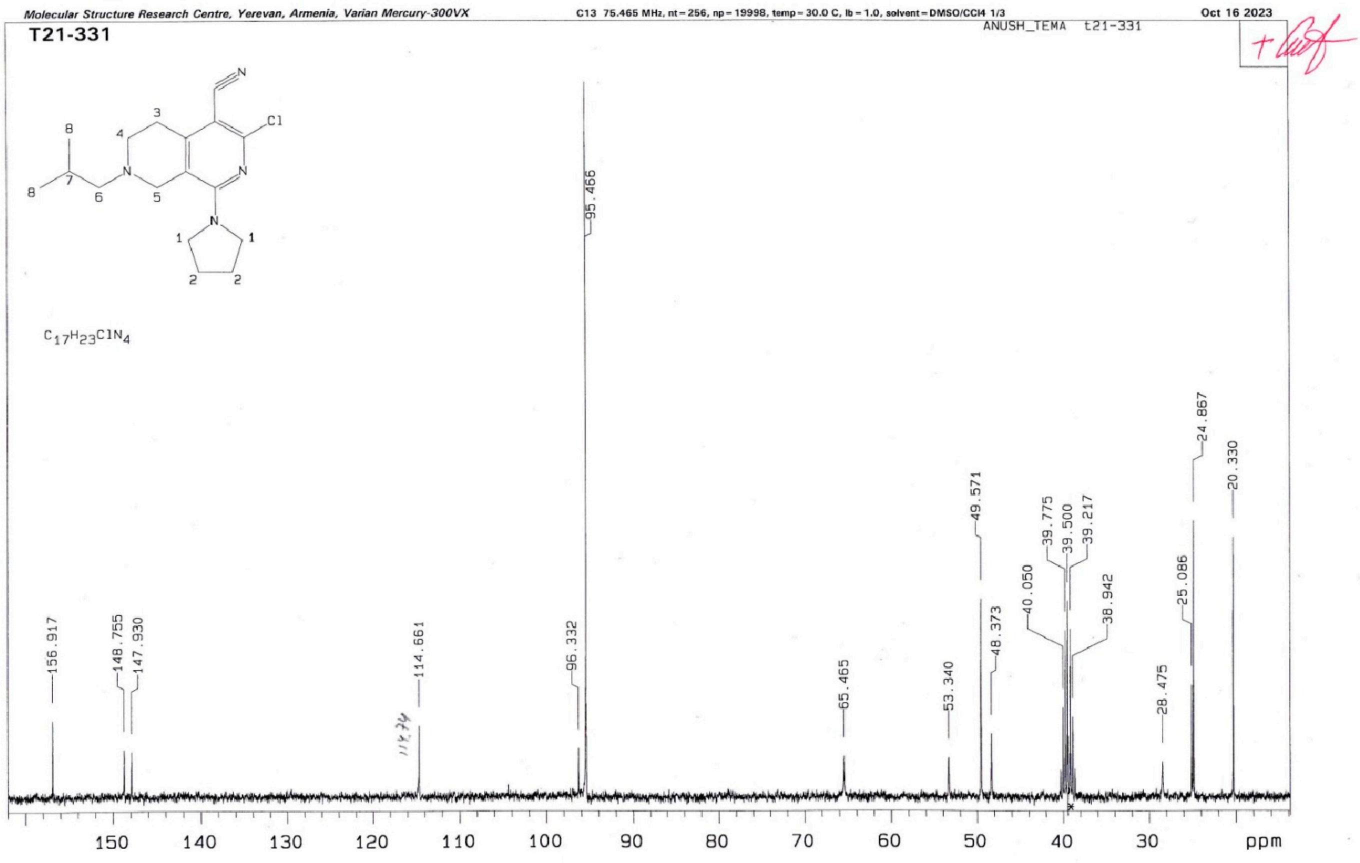

Figure S7.1. ^{13}C NMR spectrum of compound **2d**

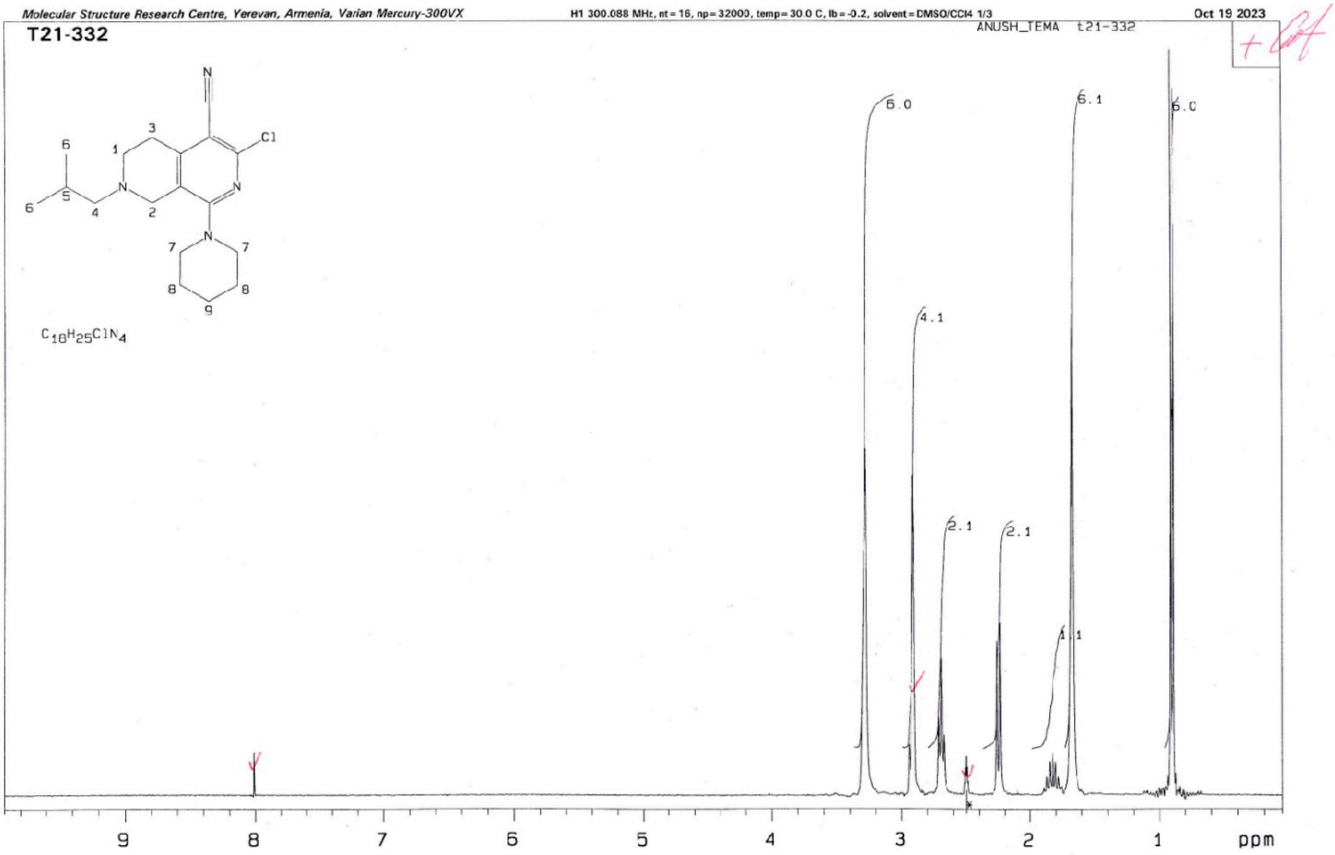

Figure S8. ^{1}H NMR spectrum of compound **2e**

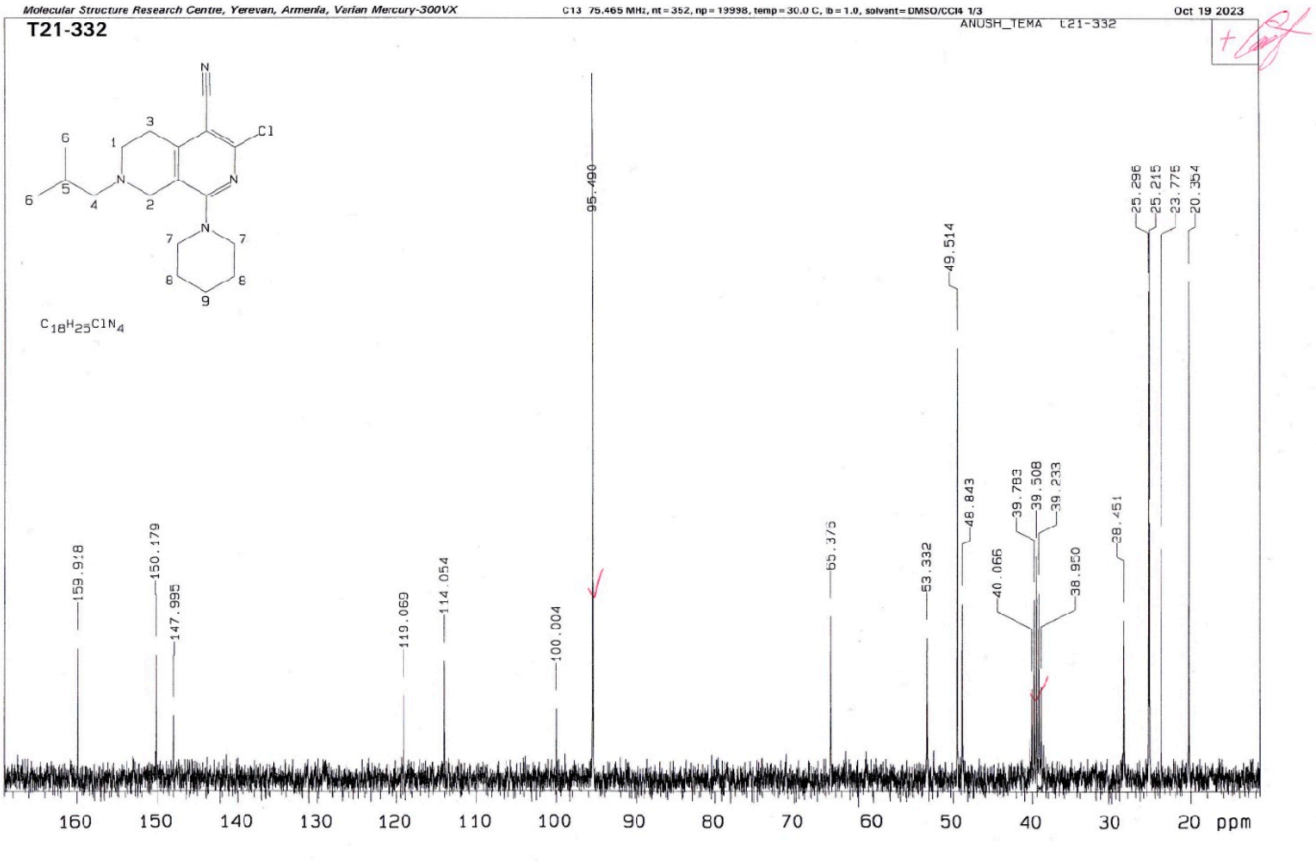

Figure S8.1. ^{13}C NMR spectrum of compound **2e**

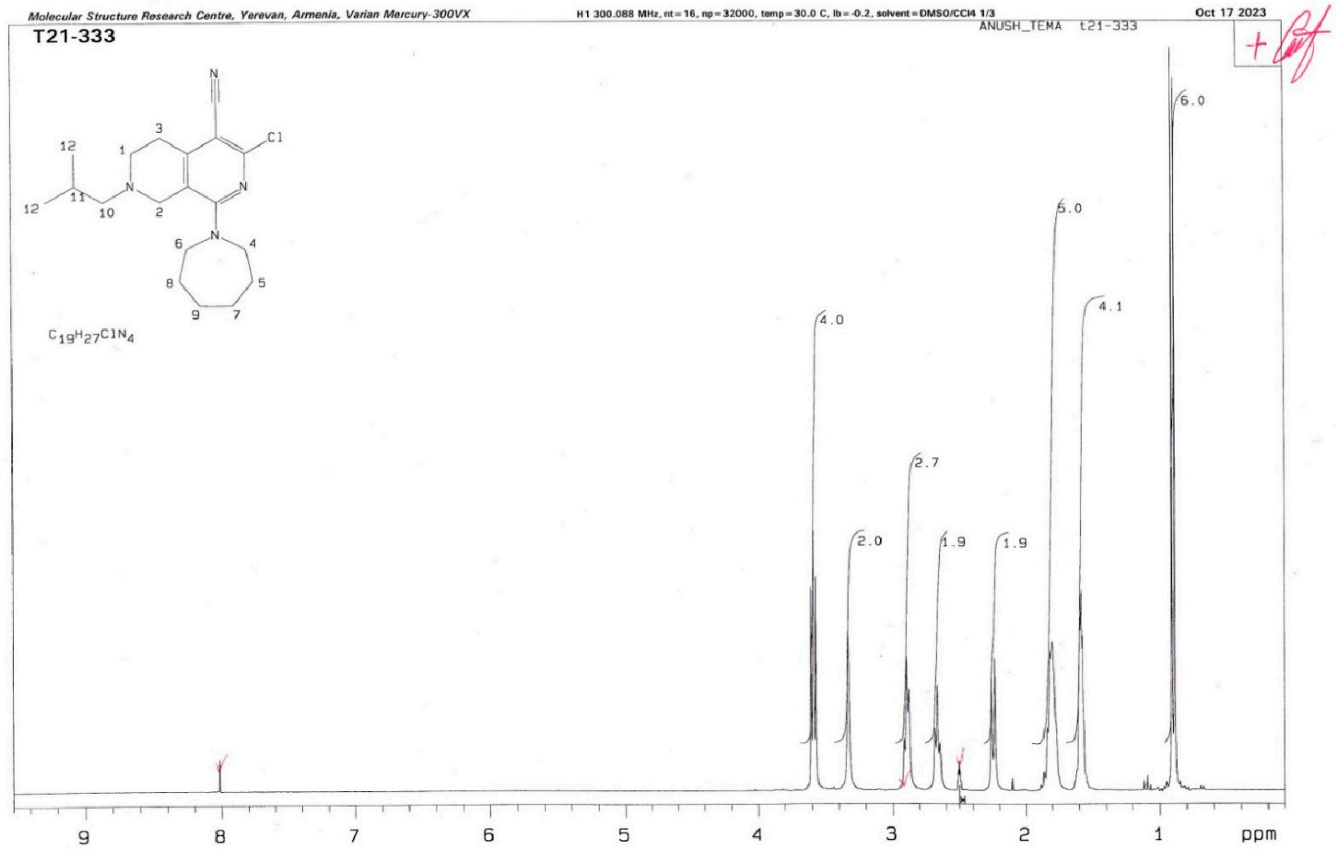

Figure S9. ^{1}H NMR spectrum of compound **2f**

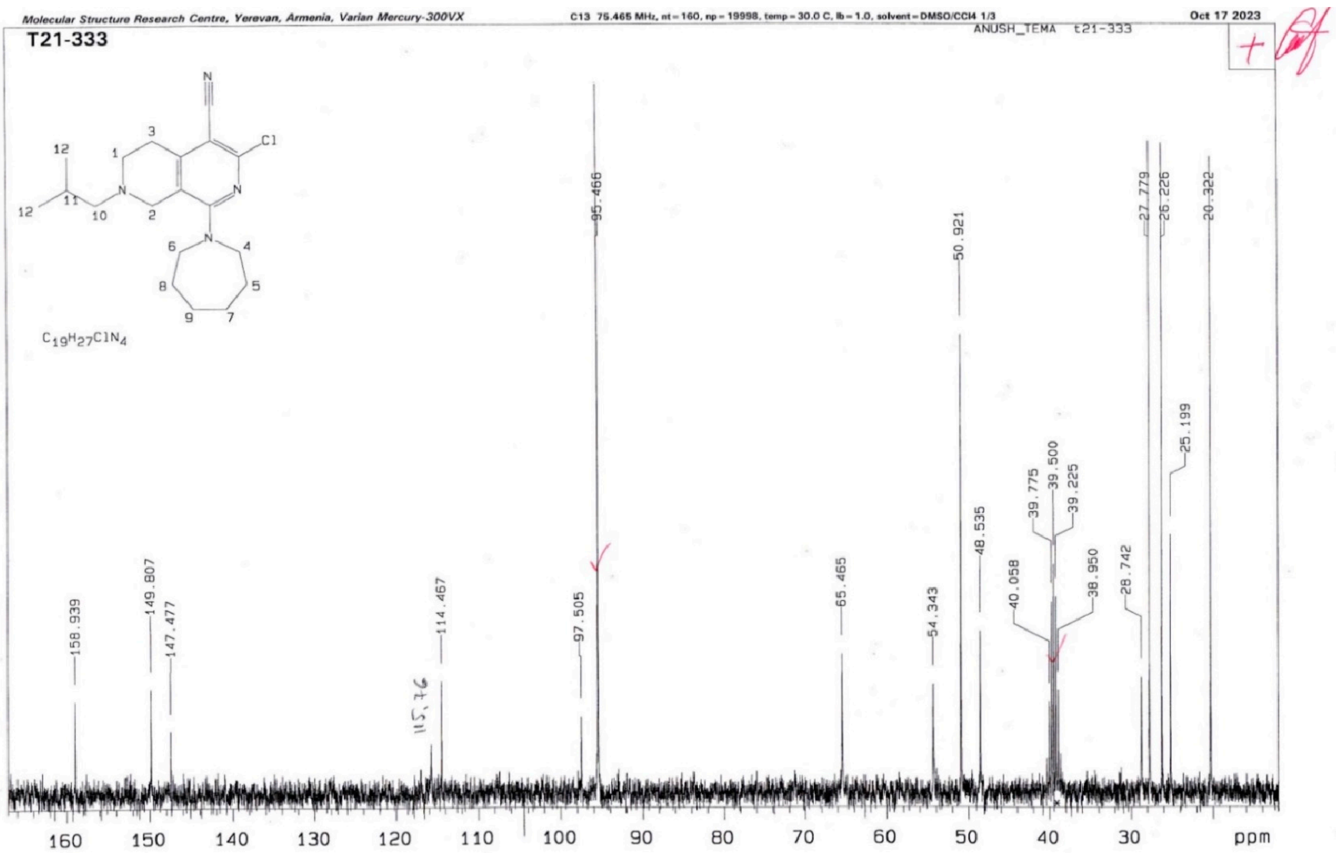

Figure S9.1. ^{13}C NMR spectrum of compound **2f**

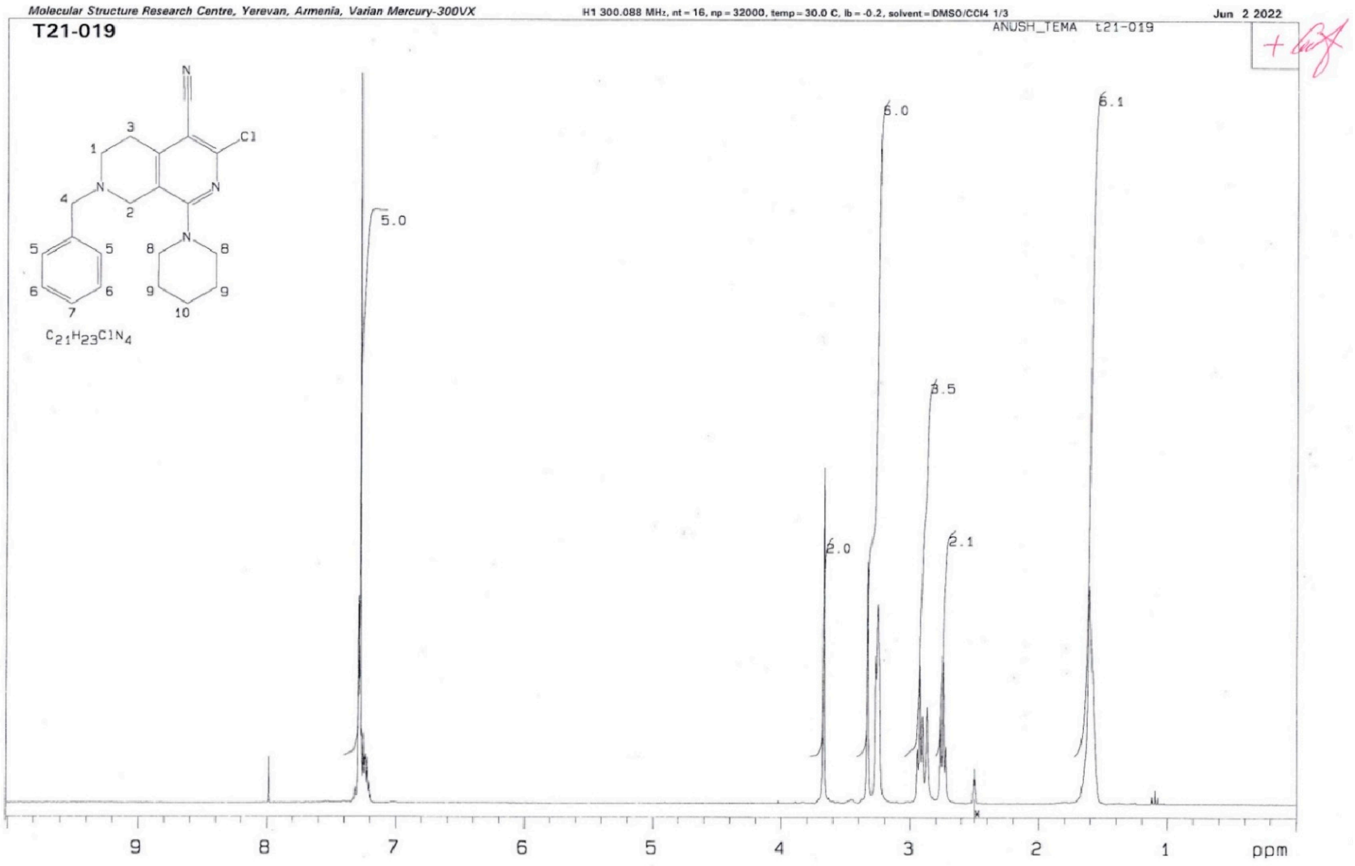

Figure S10. ^{1}H NMR spectrum of compound **2h**

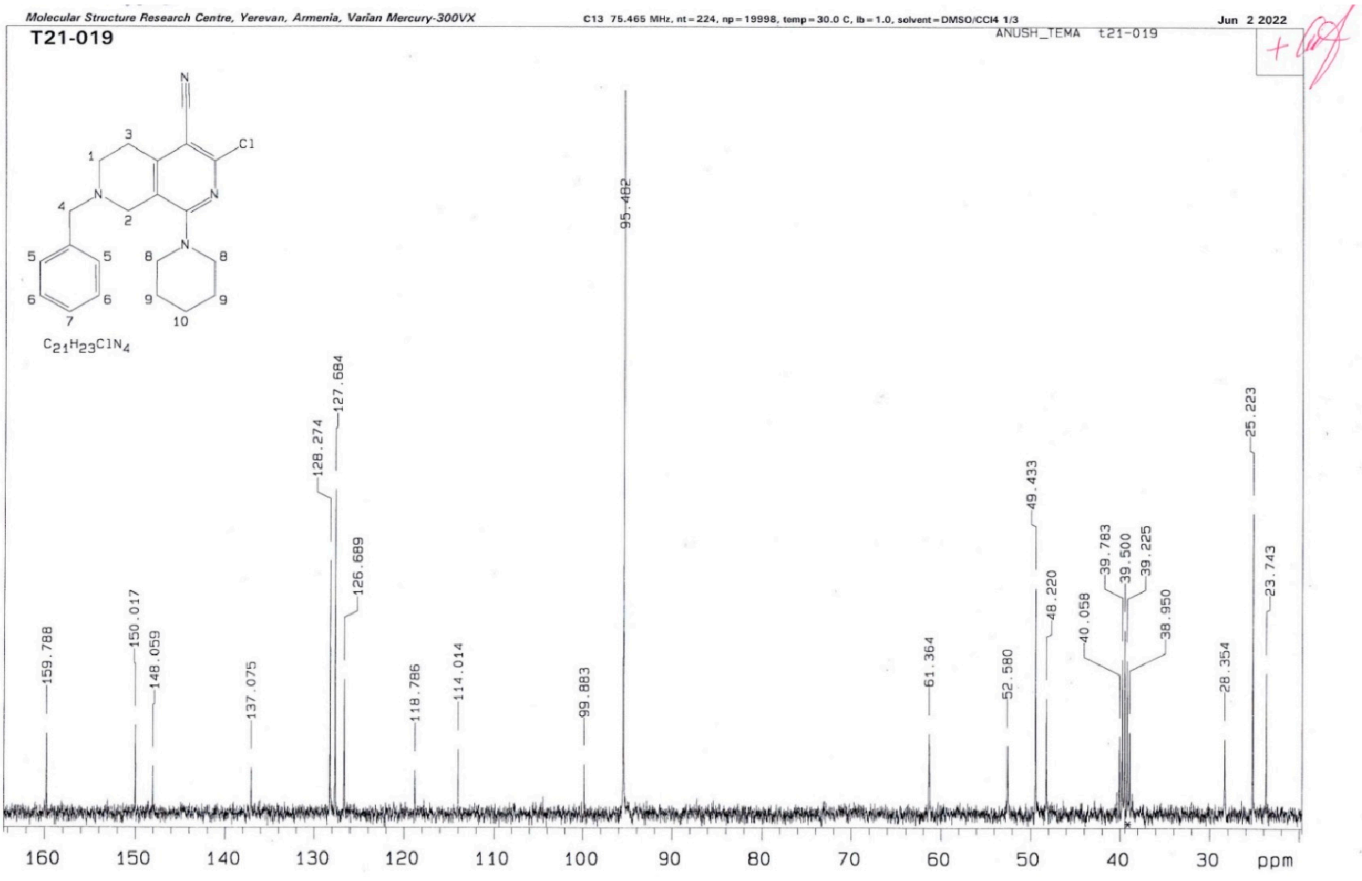

Figure S10.1. ^{13}C NMR spectrum of compound **2h**

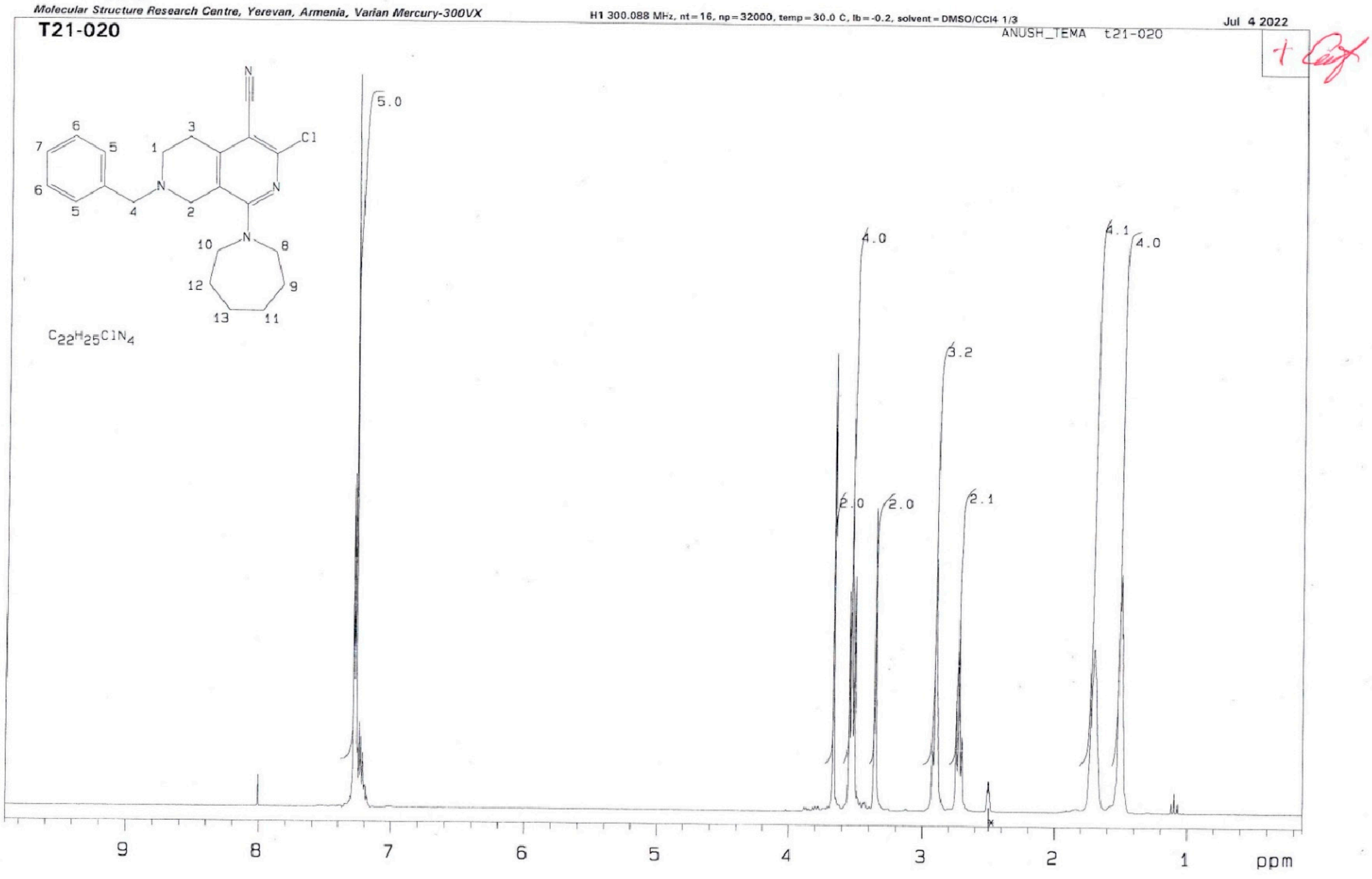

Figure S11. ^{1}H NMR spectrum of compound **2i**

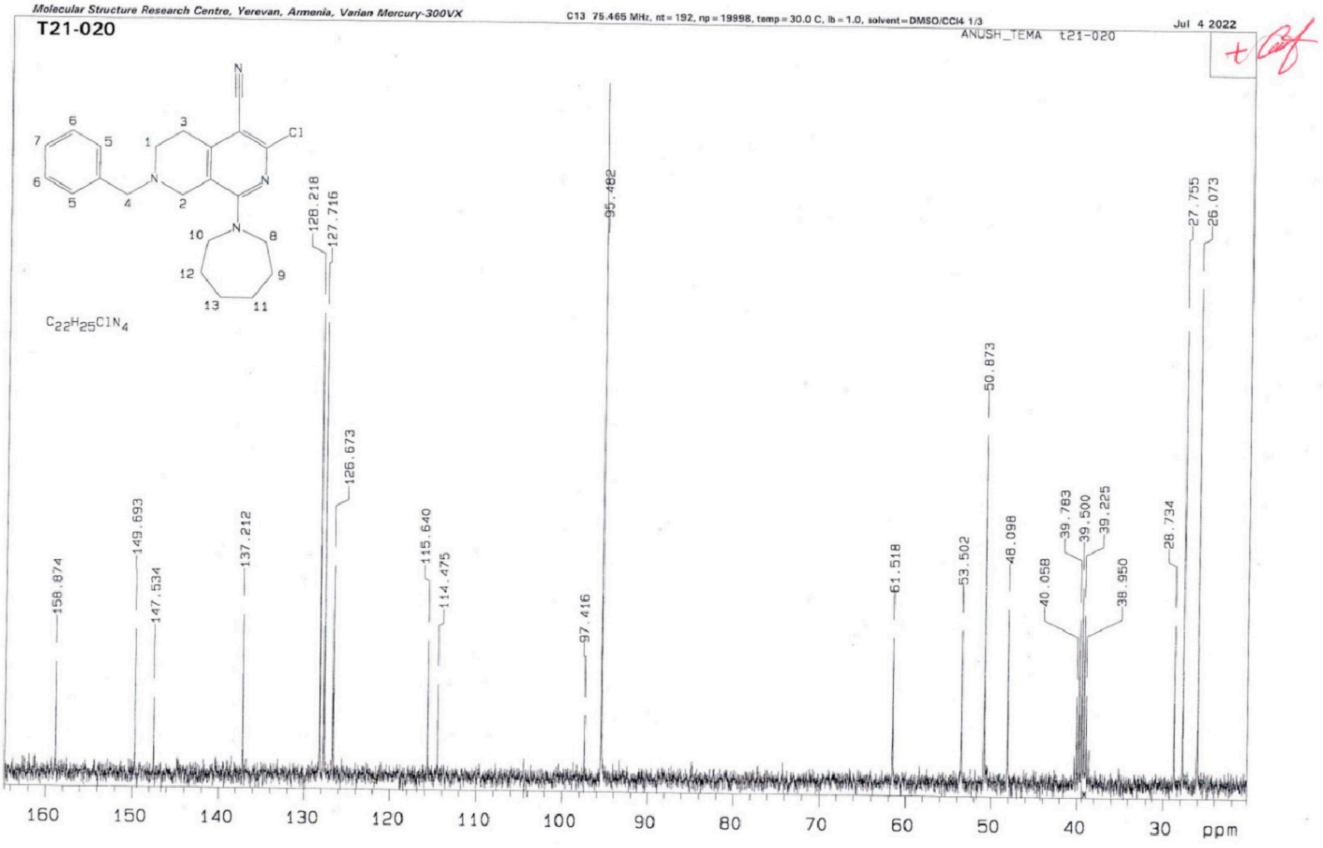

Figure S11.1. ^{13}C NMR spectrum of compound **2i**

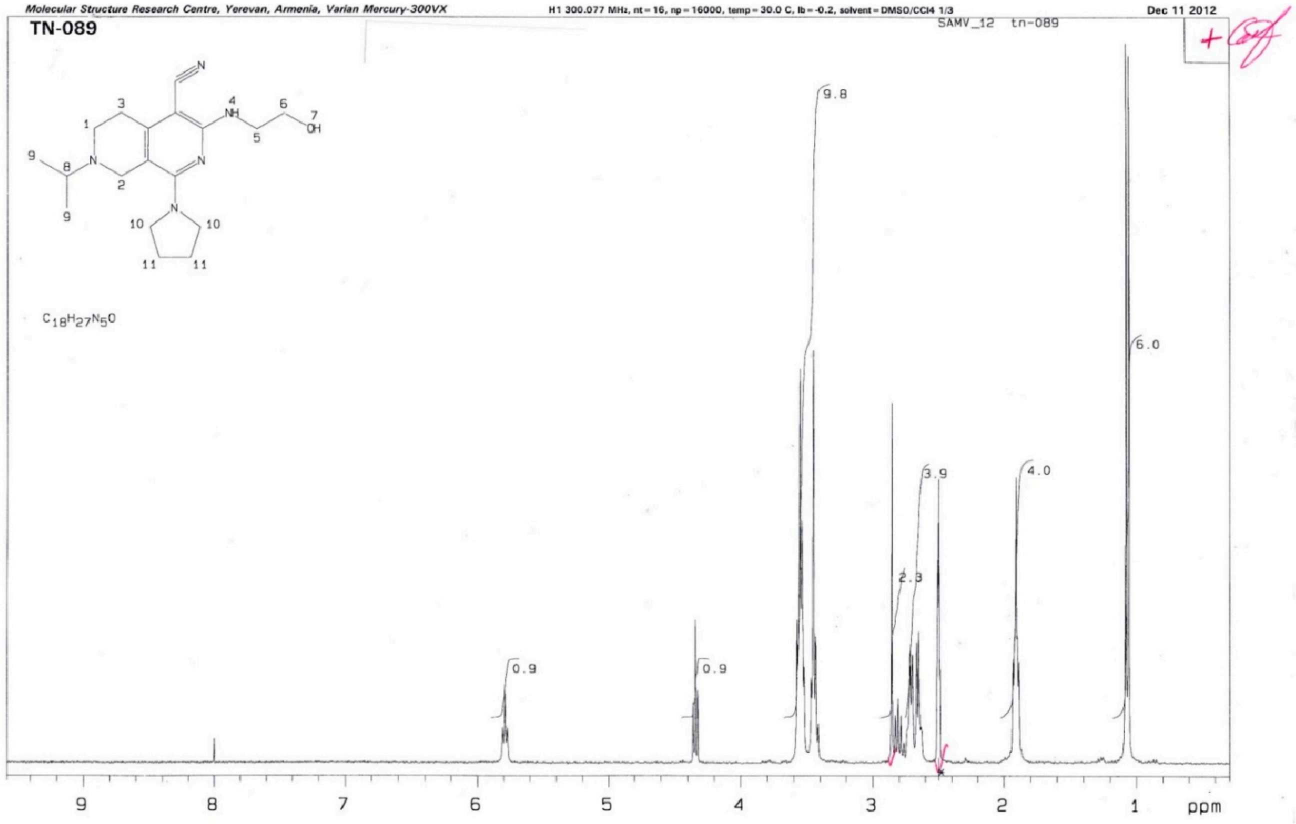

Figure S12. ^{1}H NMR spectrum of compound **3a**

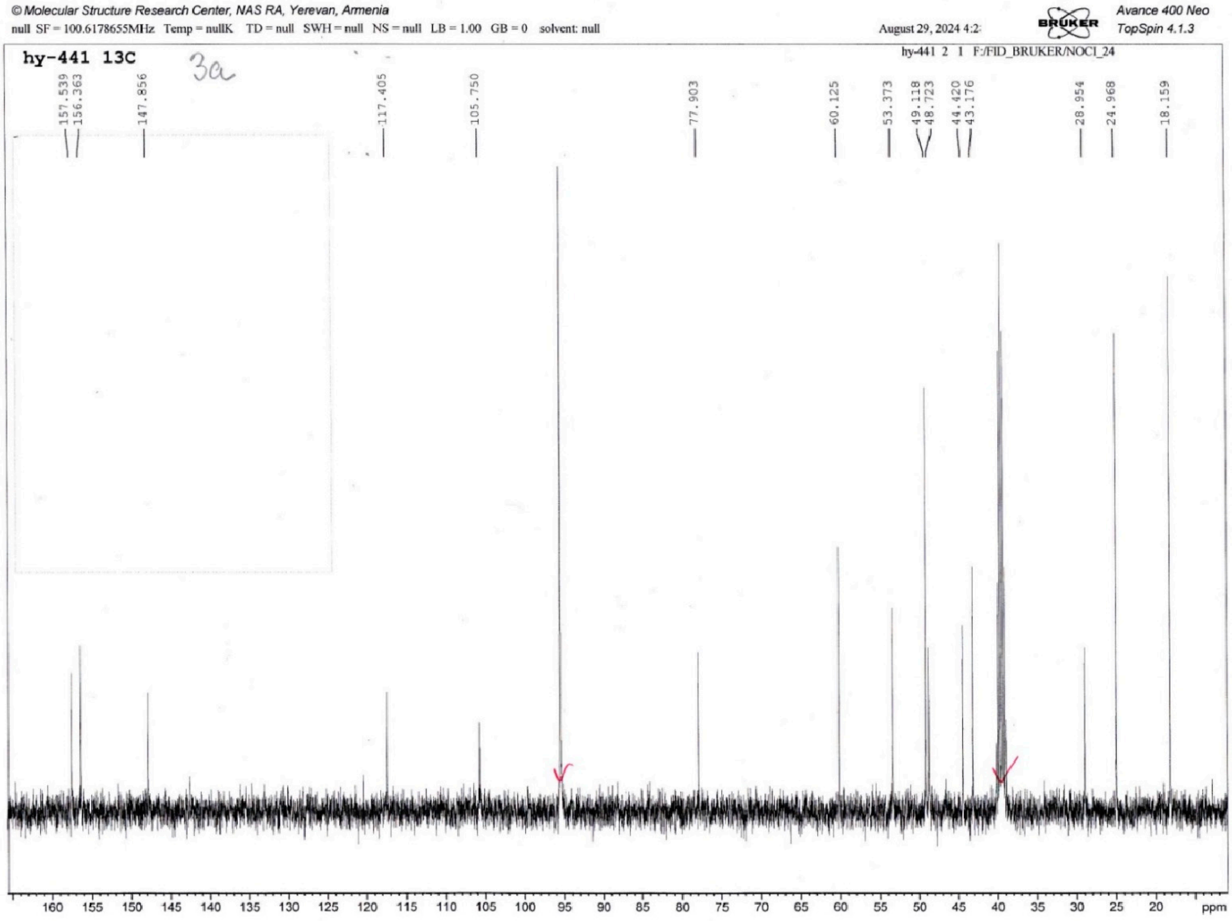

Figure S12.1. ^{13}C NMR spectrum of compound **3a**

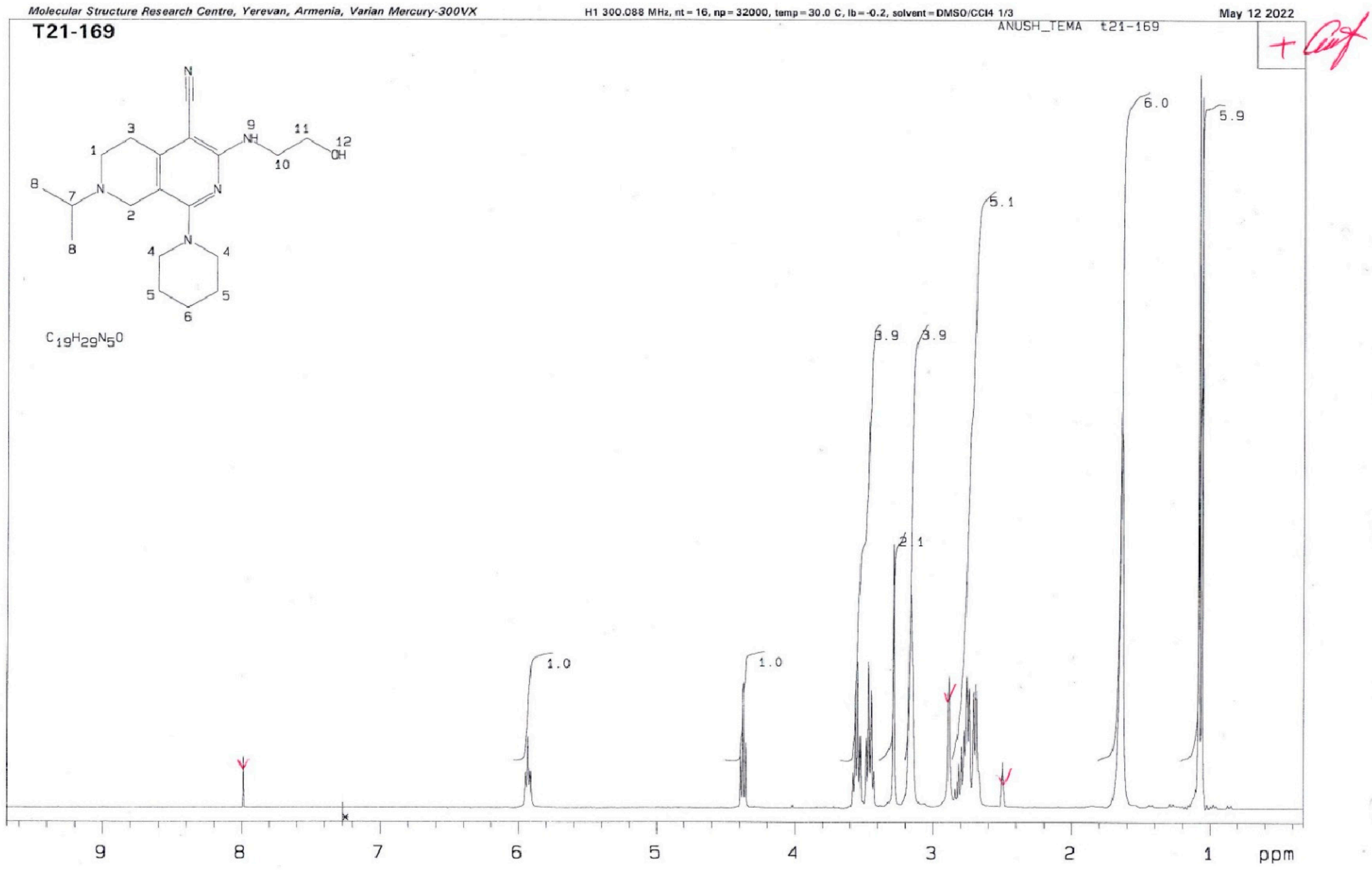

Figure S13. ^{1}H NMR spectrum of compound **3b**

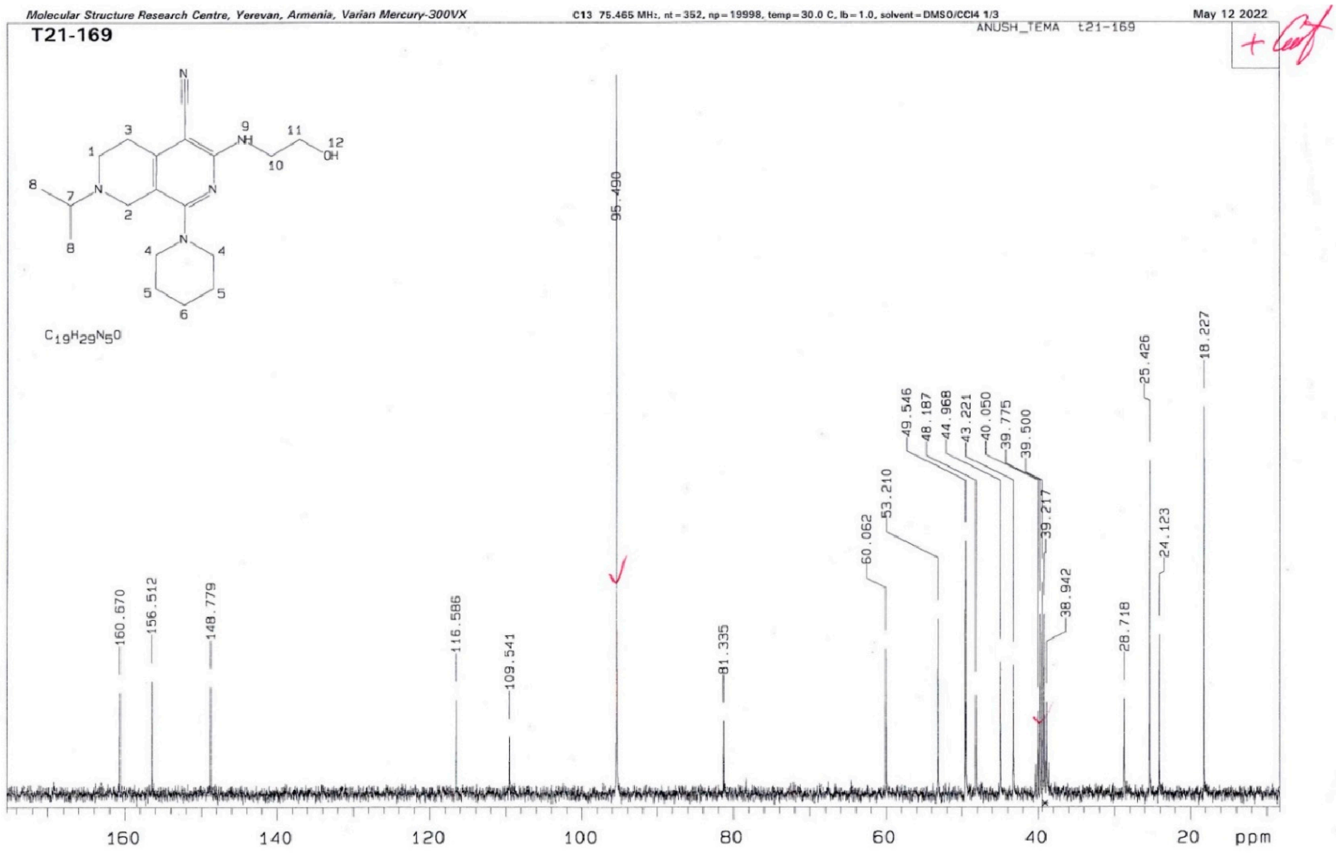

Figure S13.1. ^{13}C NMR spectrum of compound **3b**

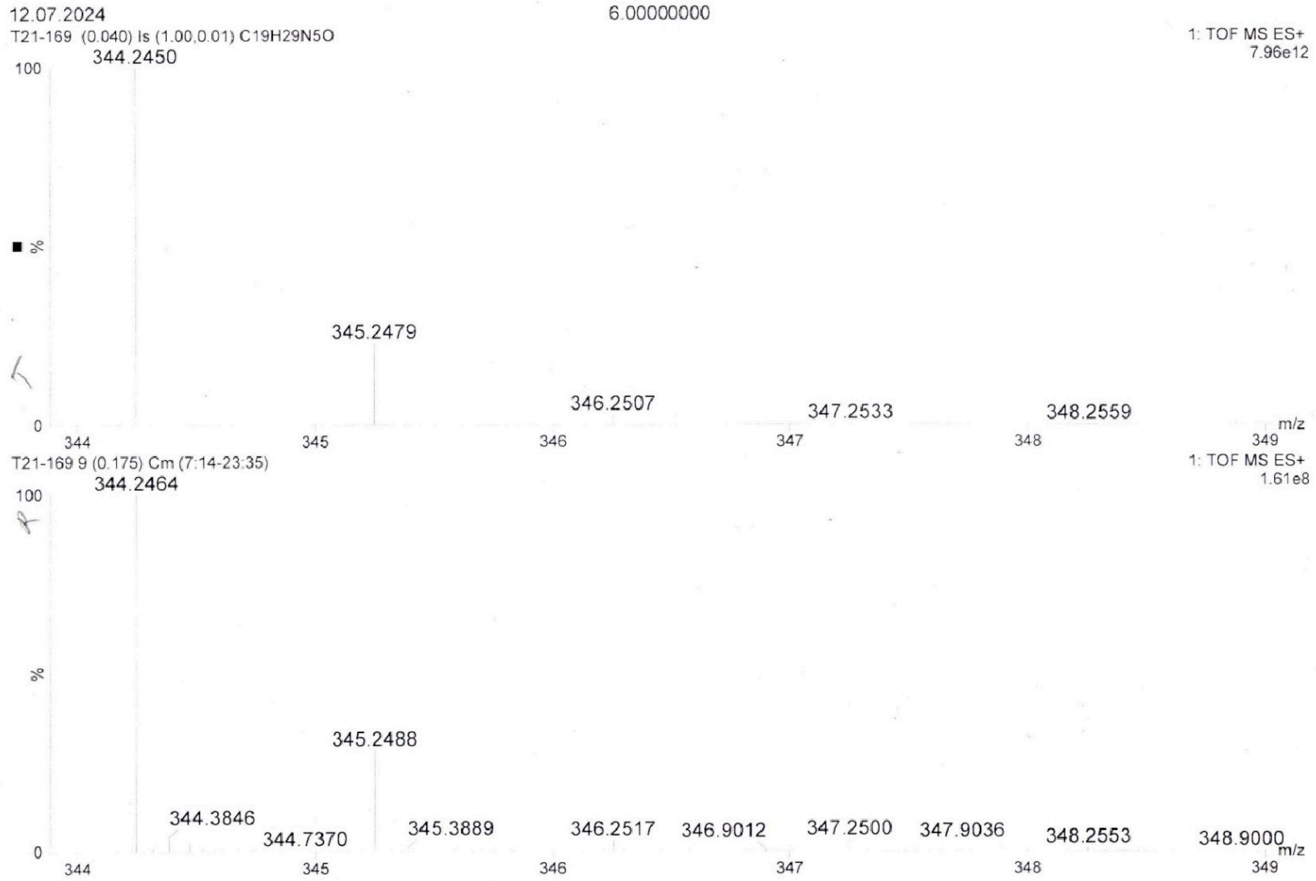

Figure S13.2. MS spectrum of compound **3b**

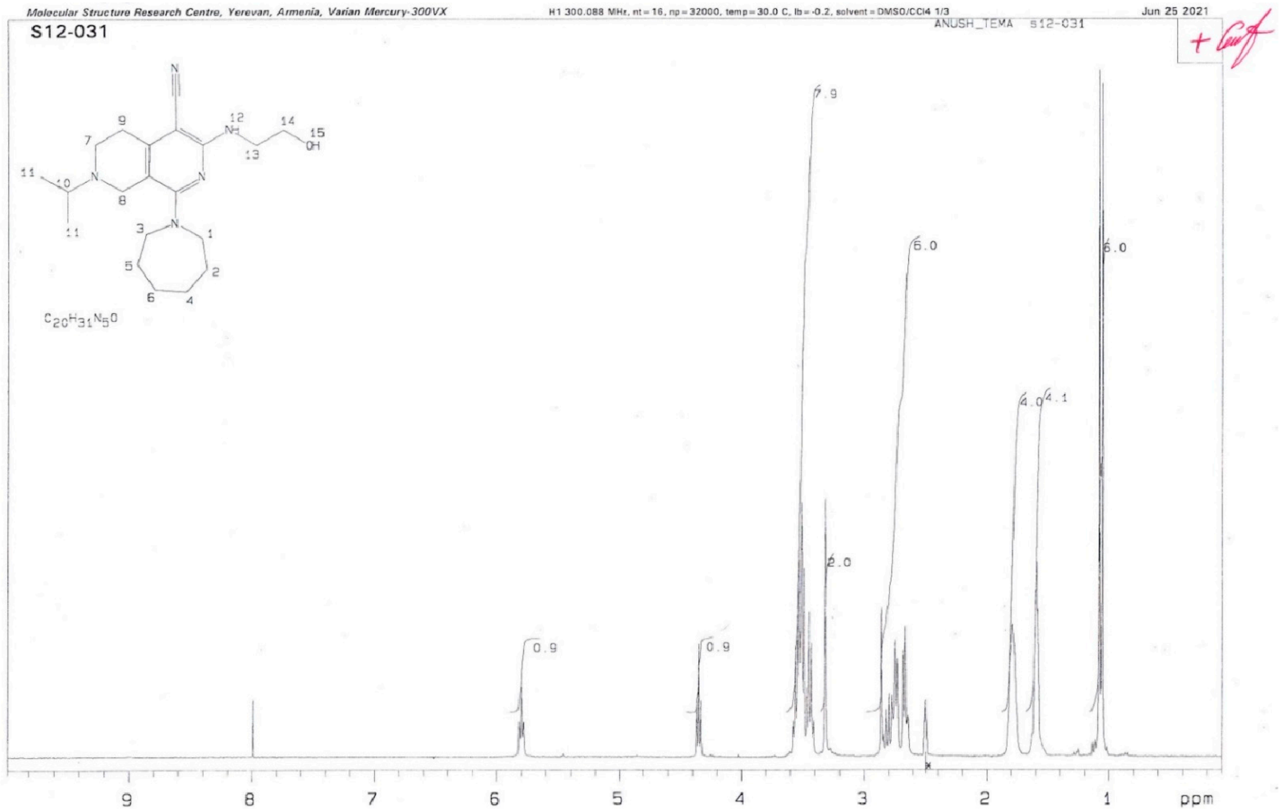

Figure S14. ^{1}H NMR spectrum of compound **3c**

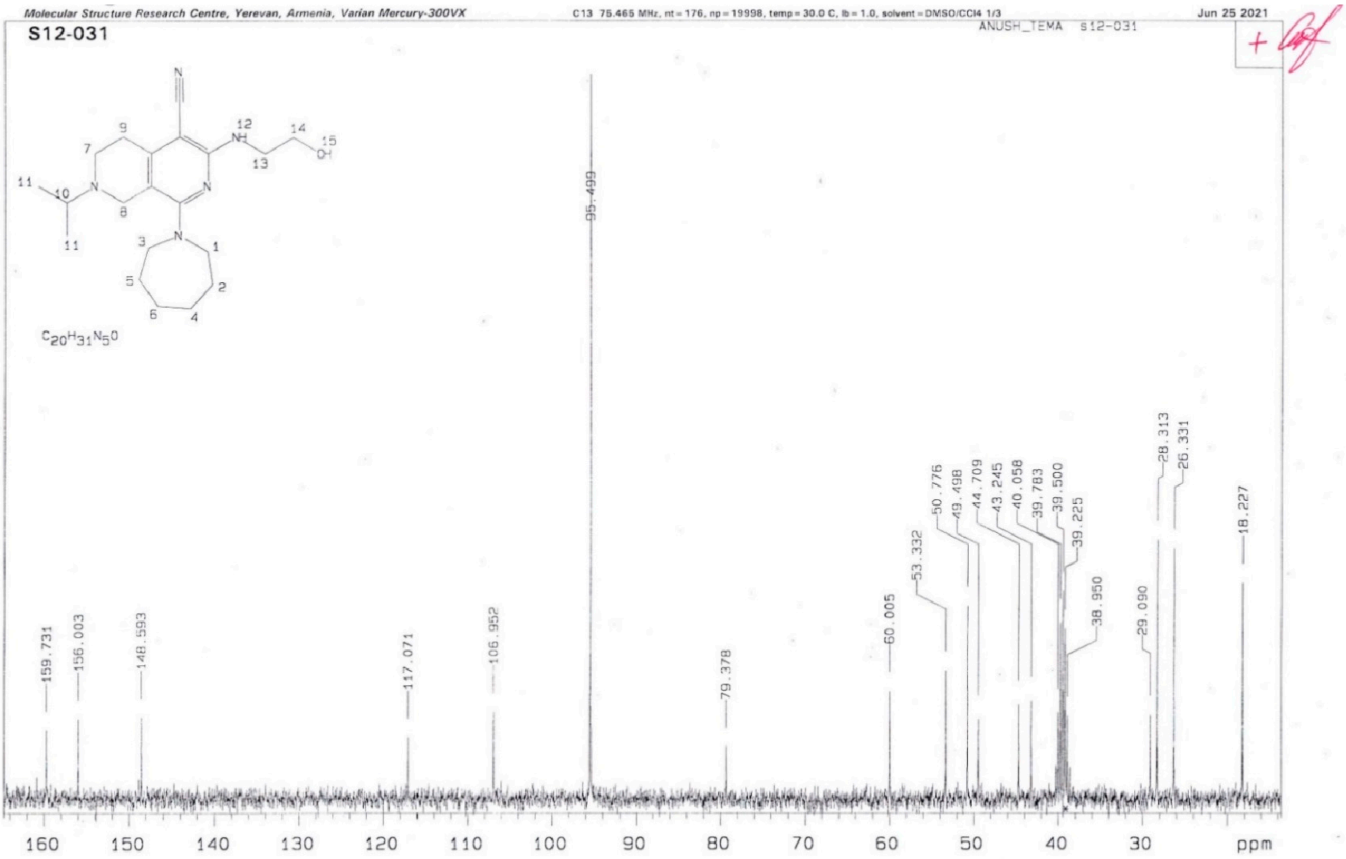

Figure S14.1 ^{13}C NMR spectrum of compound **3c**

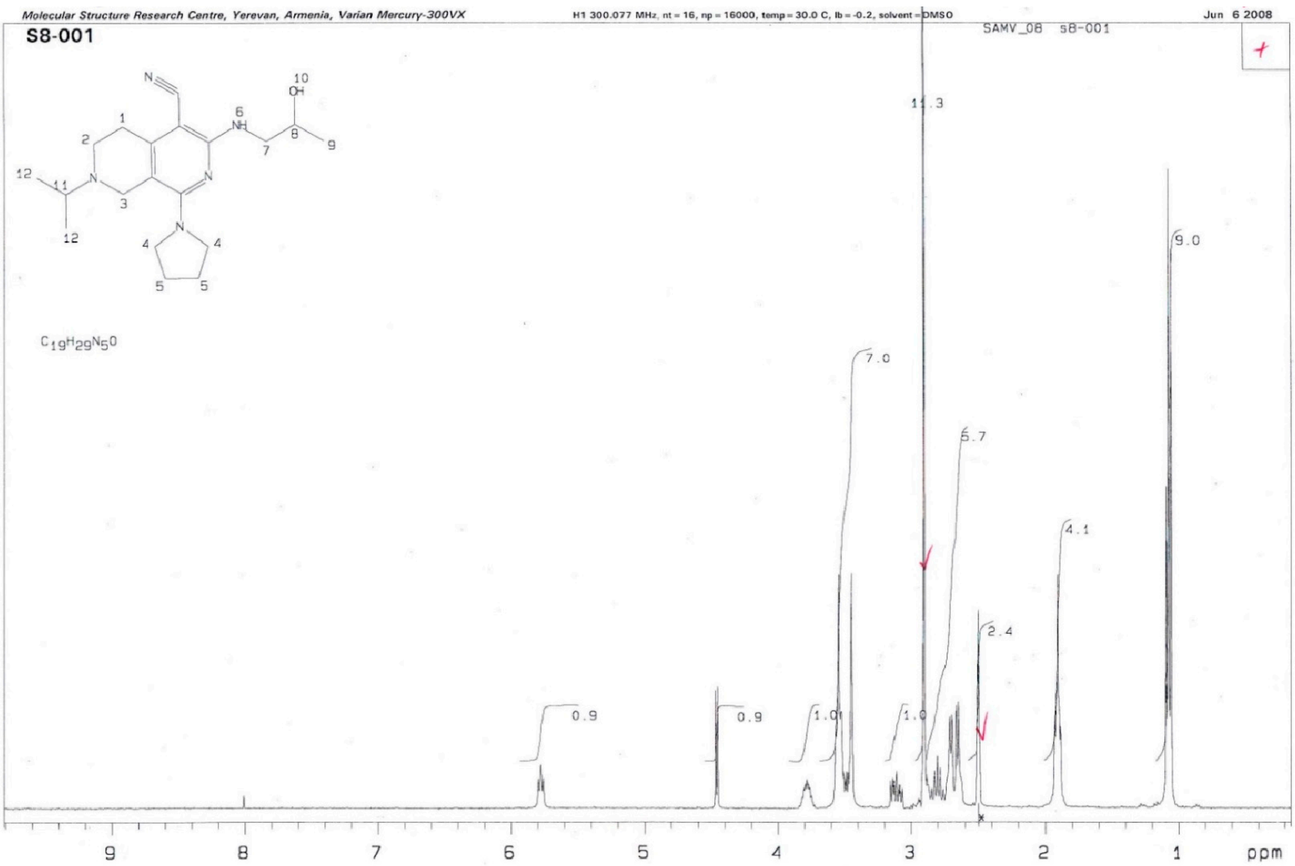

Figure S15. ^{1}H NMR spectrum of compound **3d**

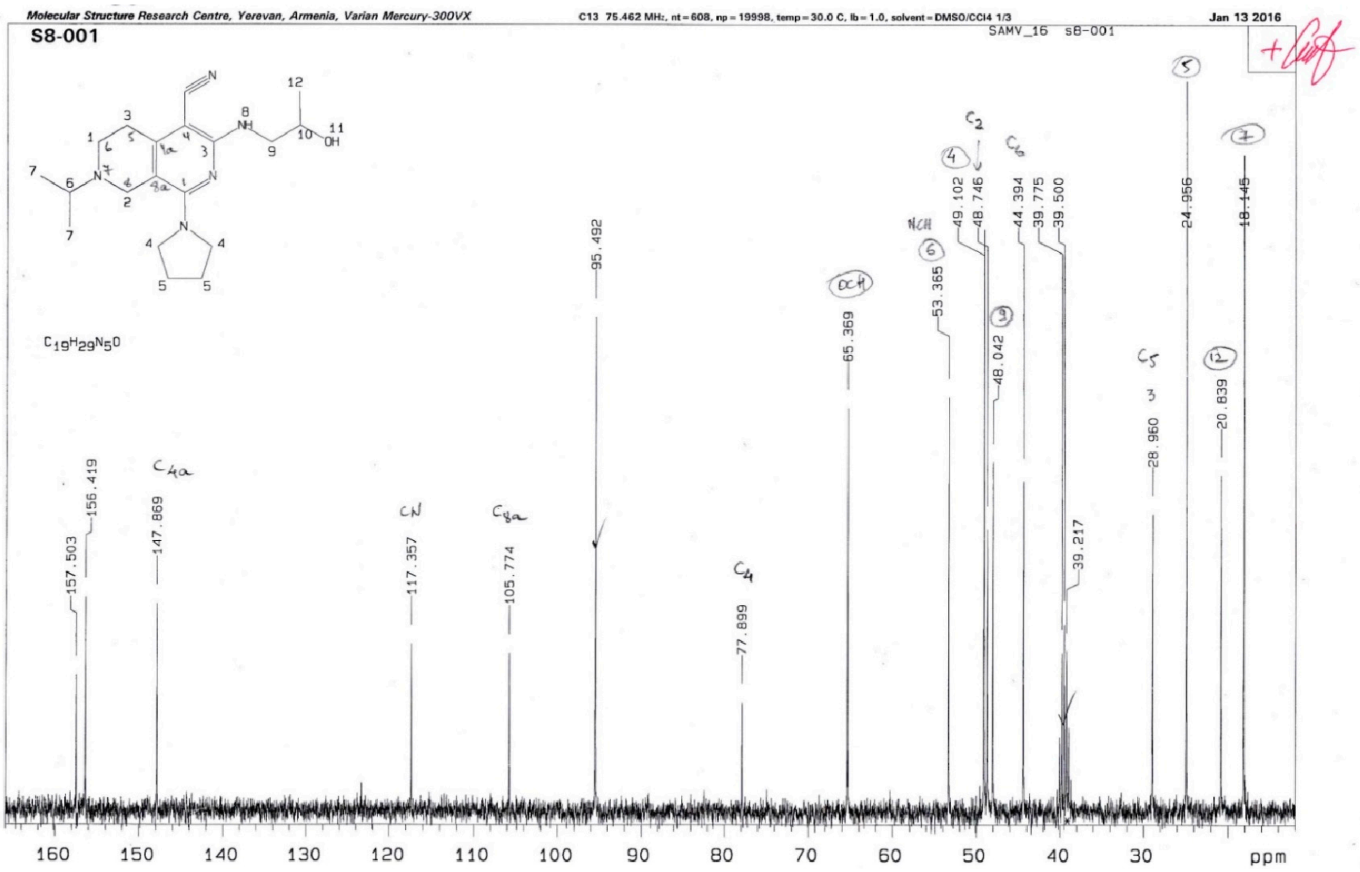

Figure S15.1. ^{13}C NMR spectrum of compound **3d**

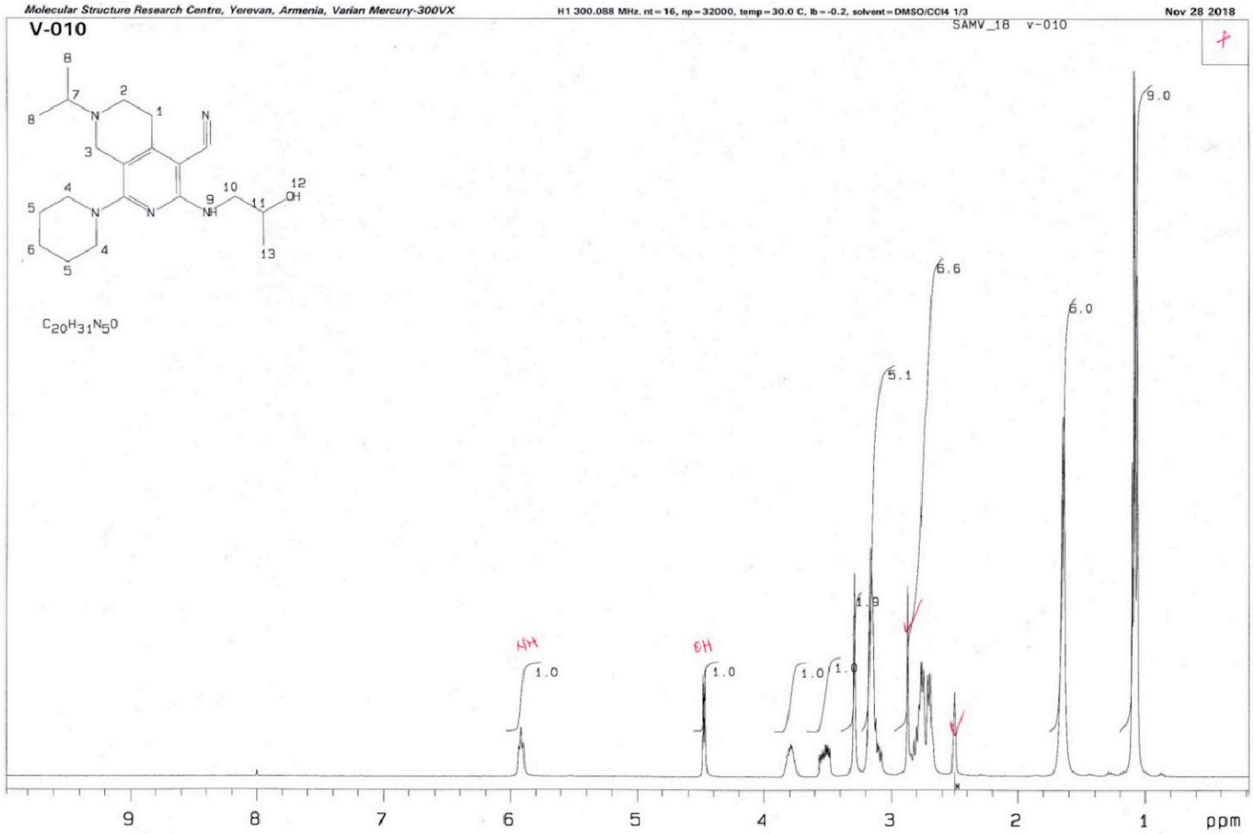

Figure S16. ^{1}H NMR spectrum of compound **3e**

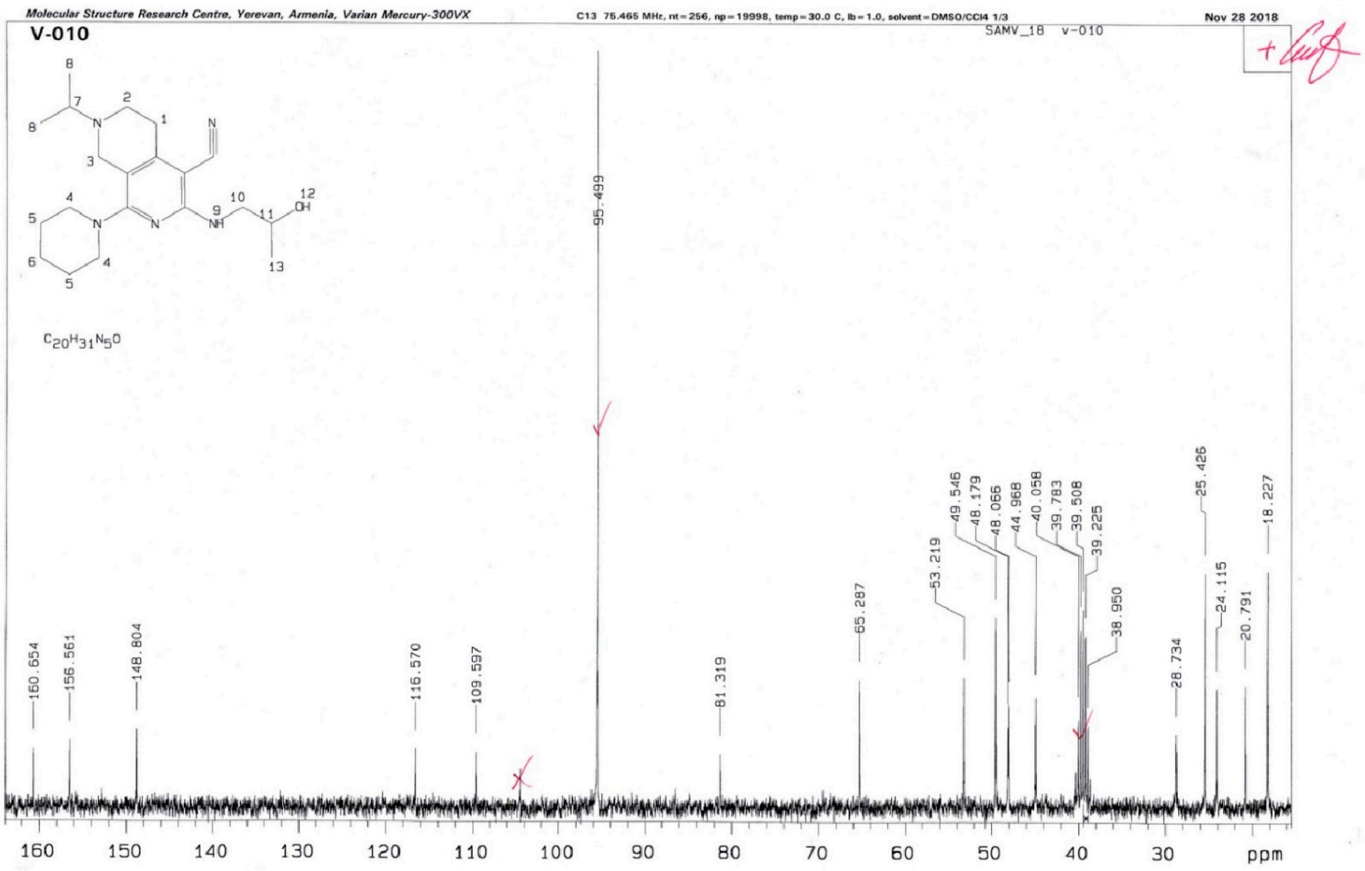

Figure S16.1. ^{13}C NMR spectrum of compound **3e**

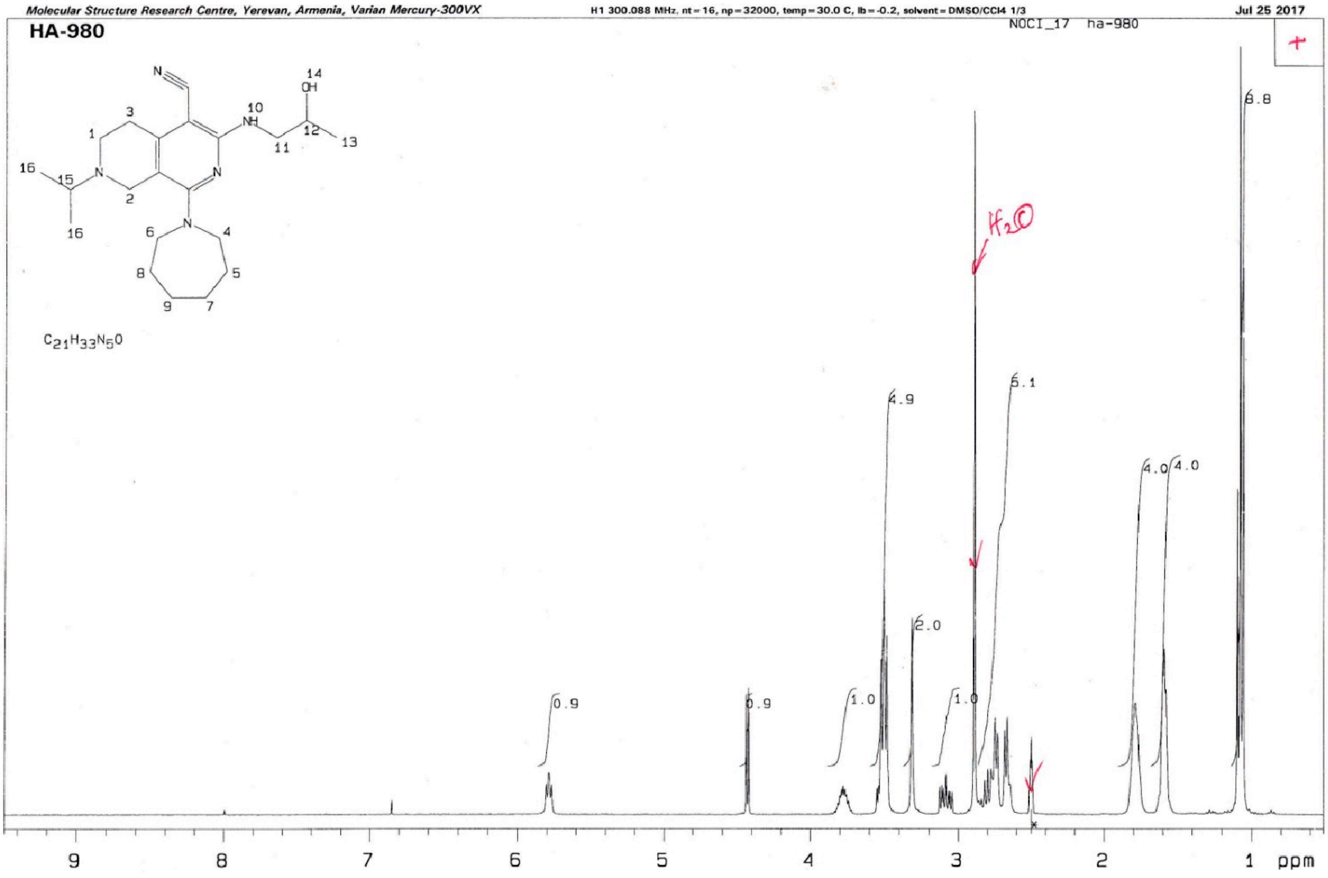

Figure S17. ^{1}H NMR spectrum of compound **3f**

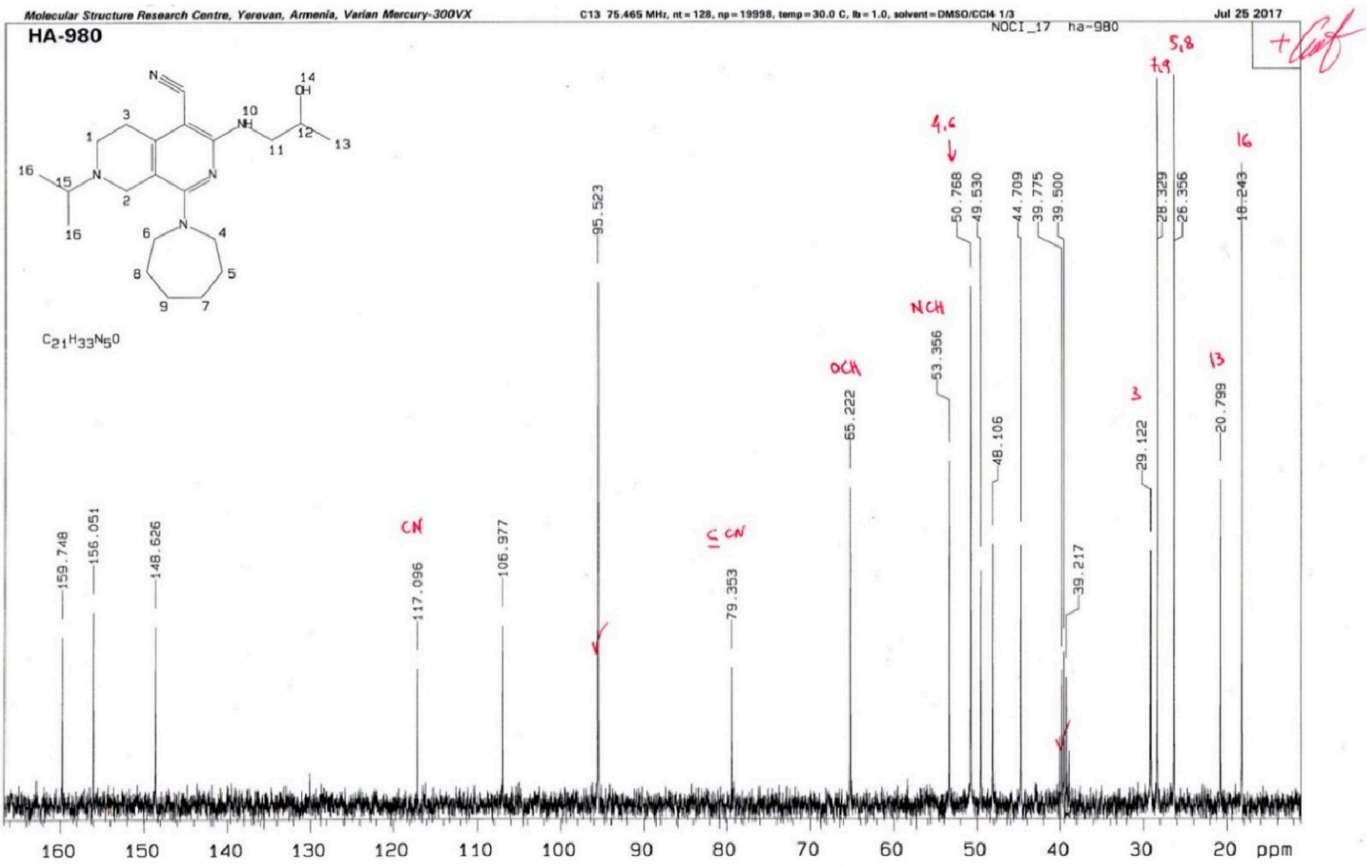

Figure S17.1. ^{13}C NMR spectrum of compound **3f**

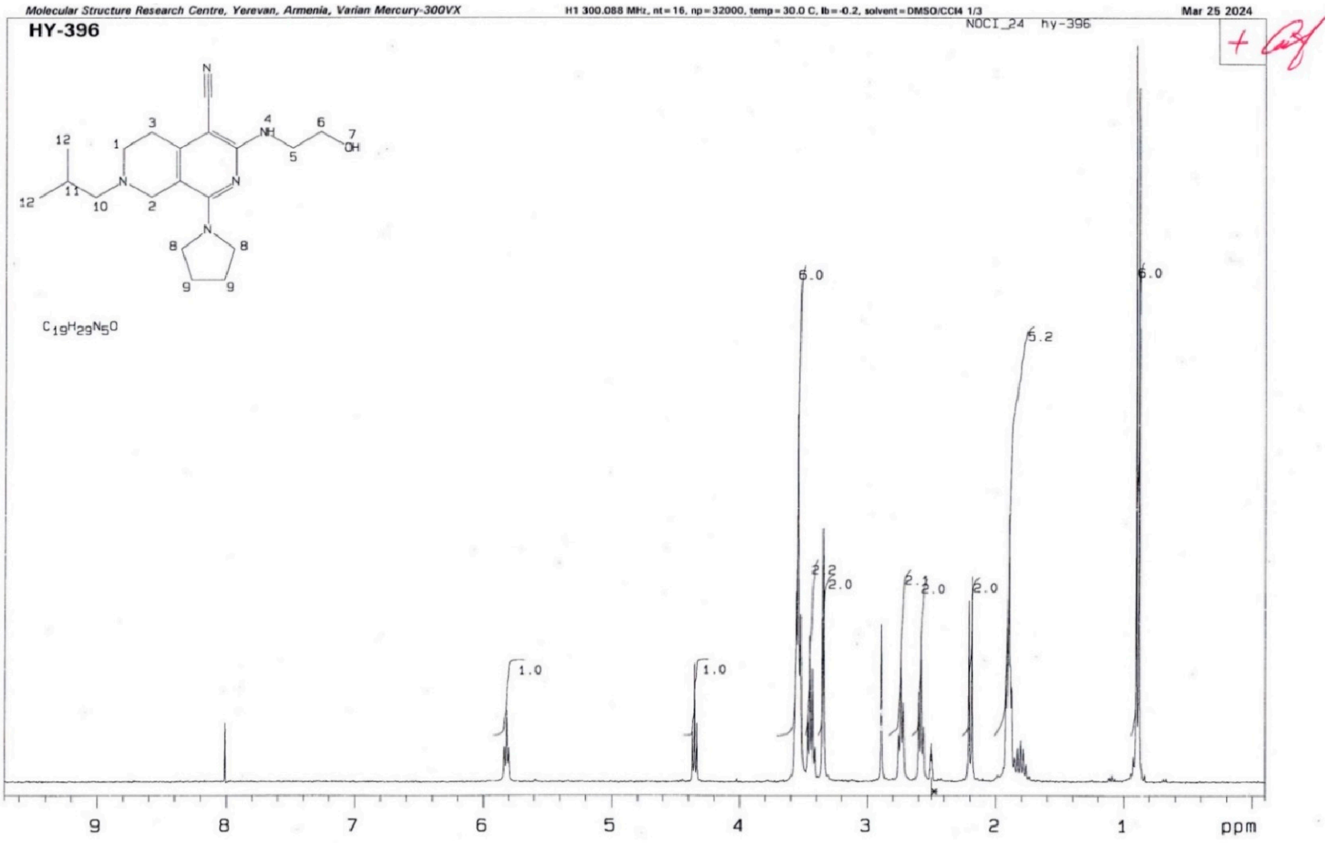

Figure S18. ^{1}H NMR spectrum of compound **3g**

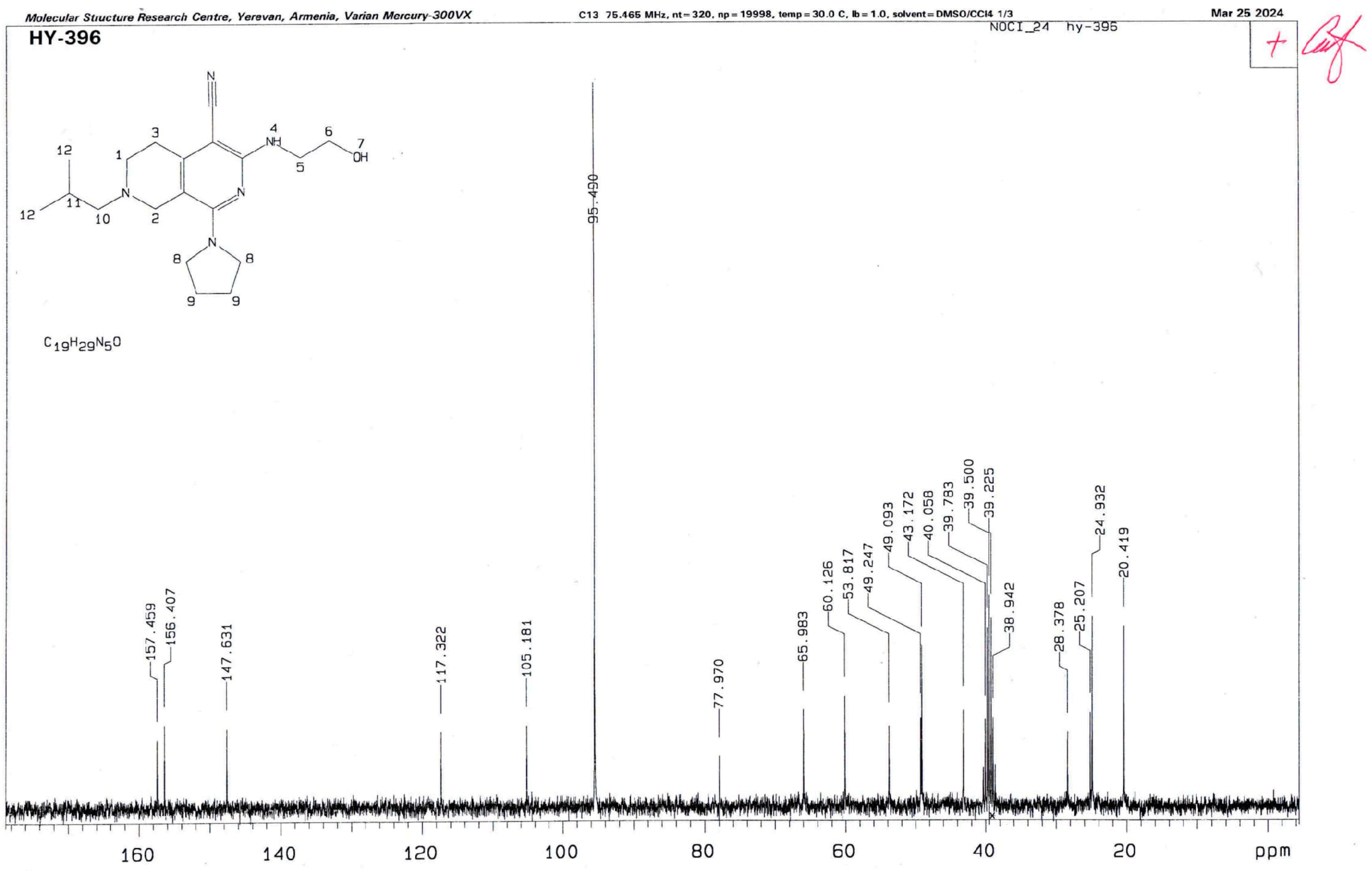

Figure S18.1. ^{13}C NMR spectrum of compound **3g**

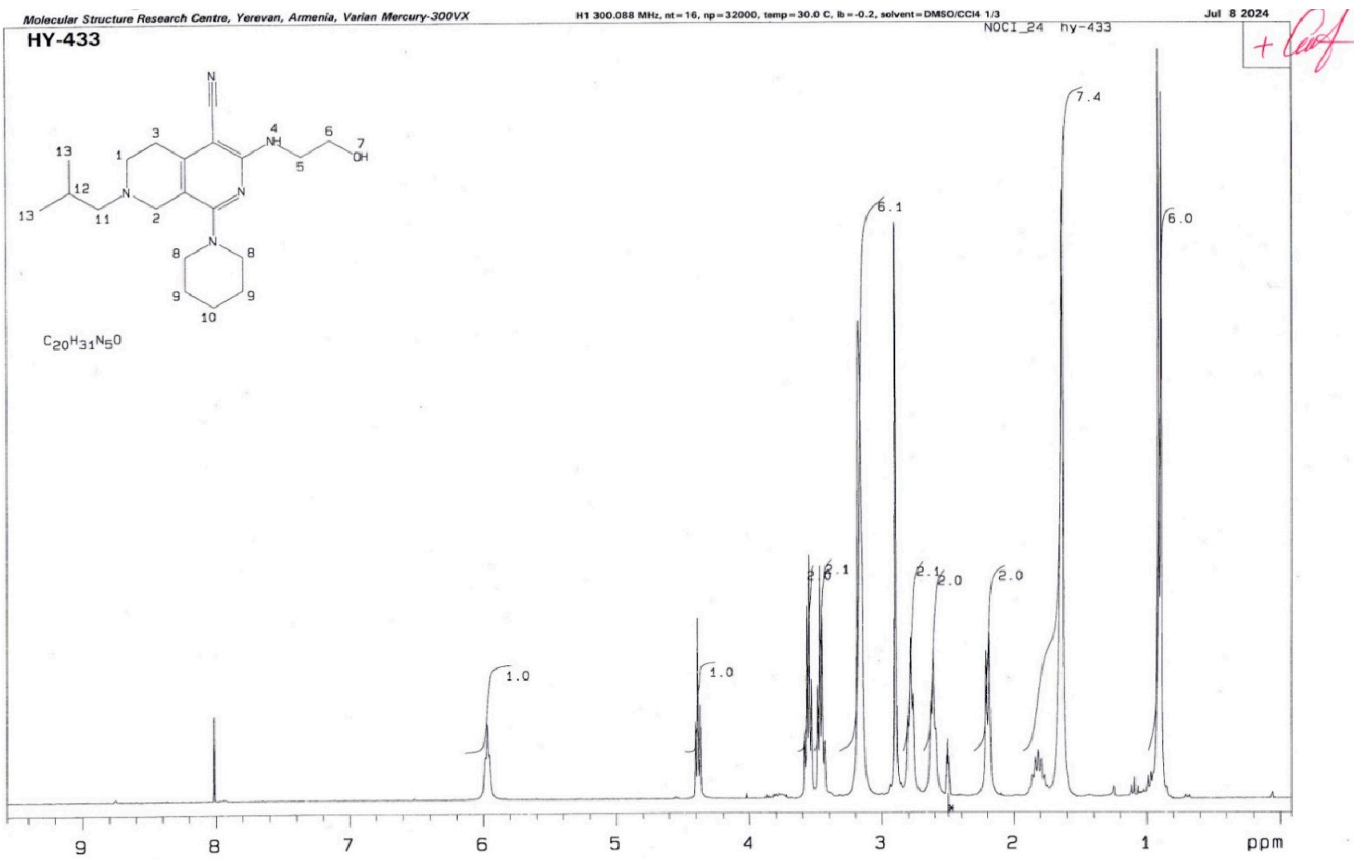

Figure S19. ^{1}H NMR spectrum of compound **3h**

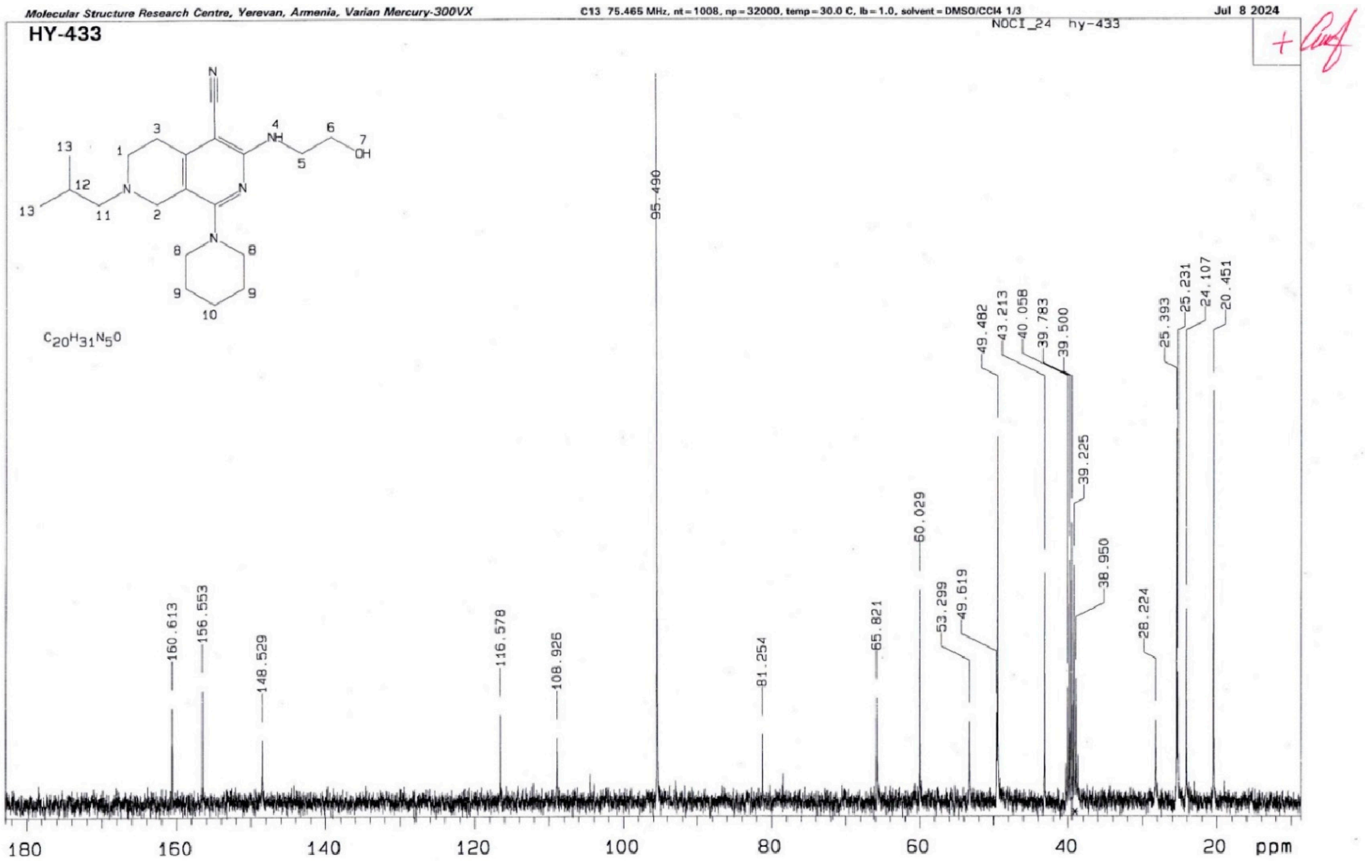

Figure S19.1. ^{13}C NMR spectrum of compound **3h**

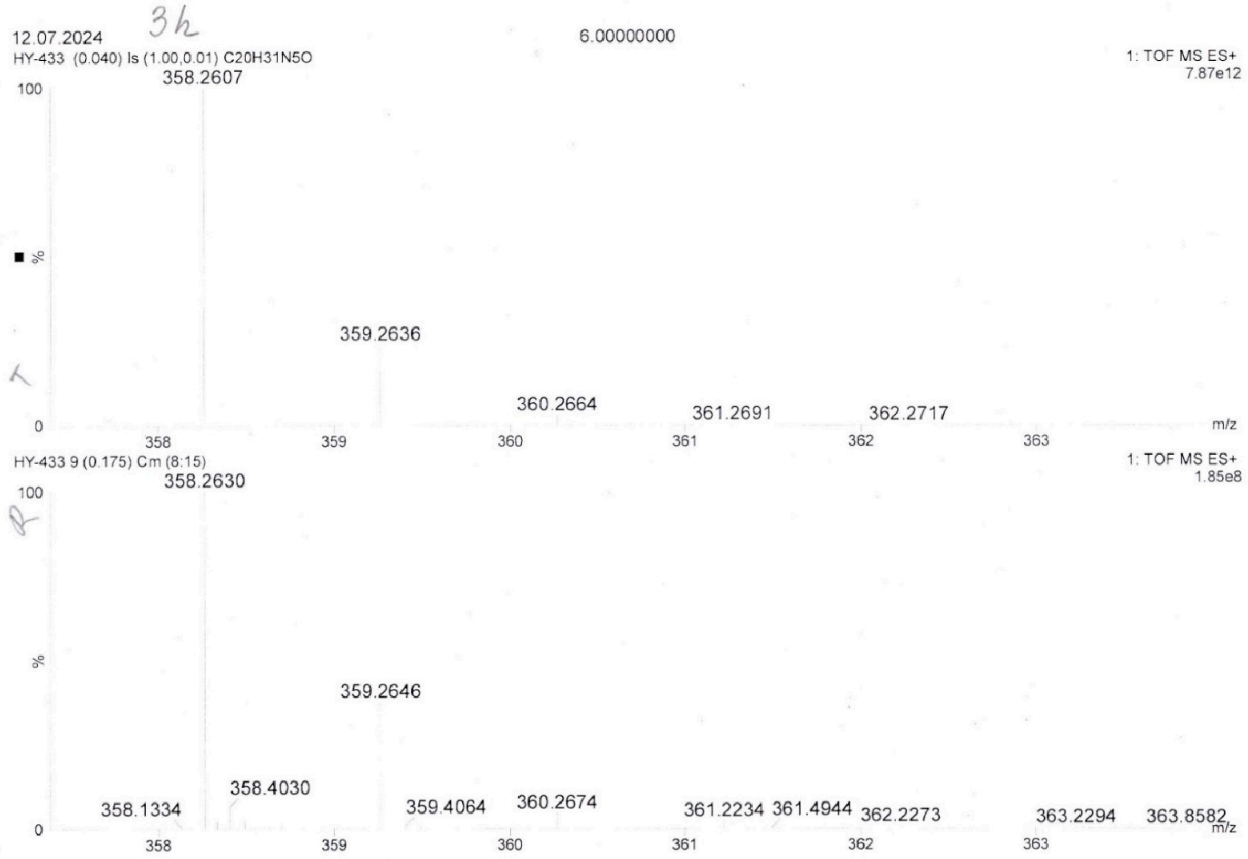

Figure S19.2. MS spectrum of compound **3h**

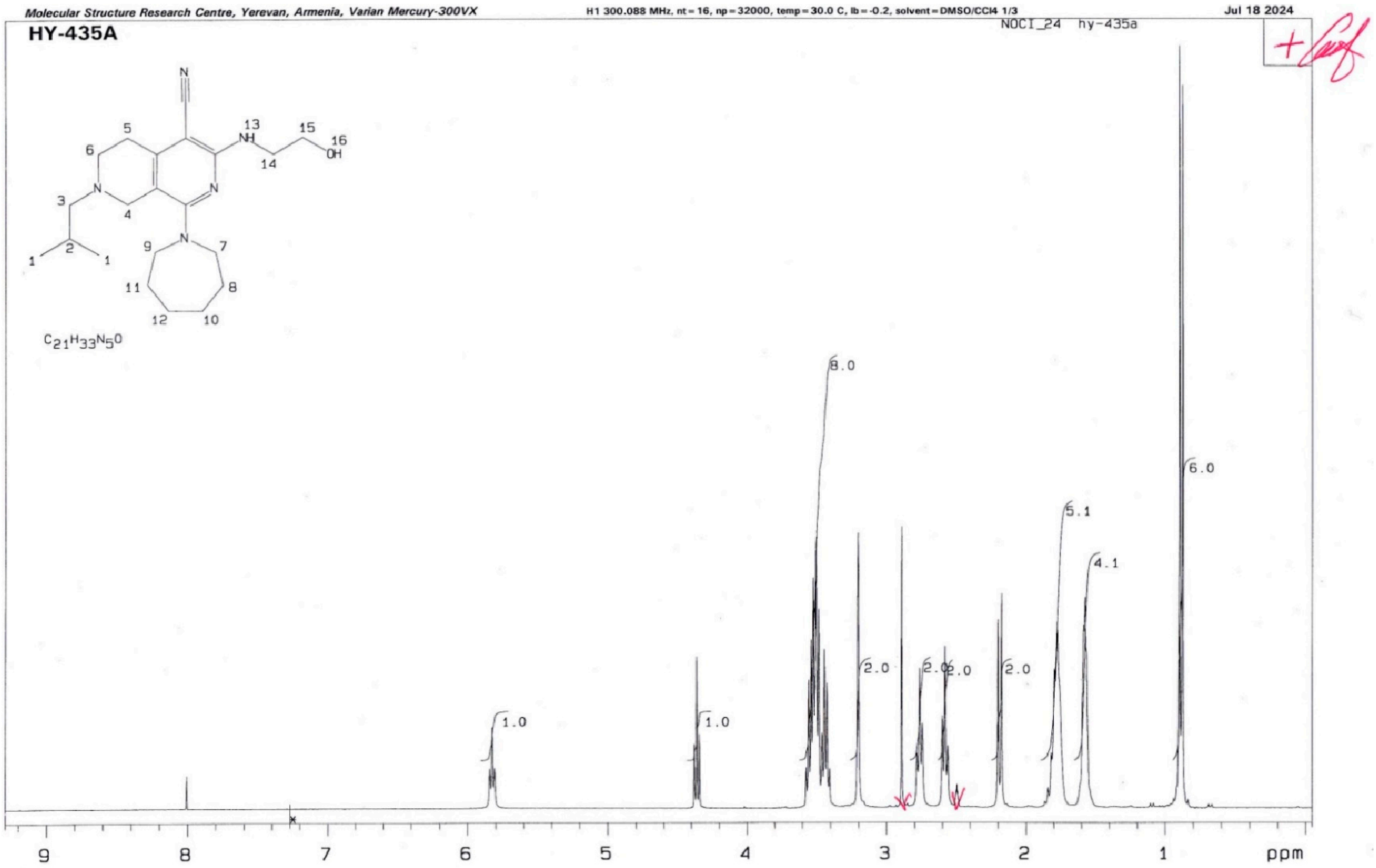

Figure S20. ^{1}H NMR spectrum of compound **3i**

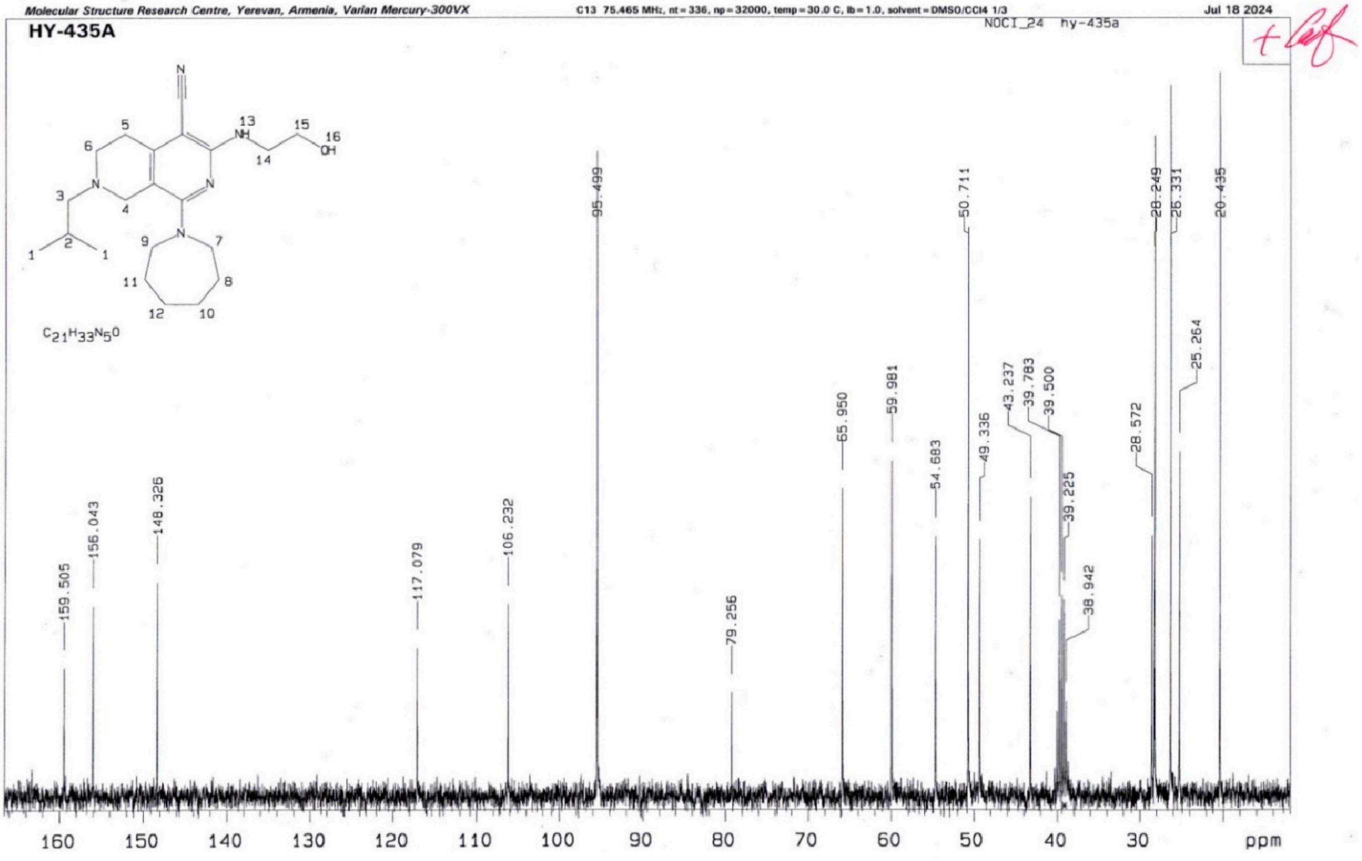

Figure S20.1. ^{13}C NMR spectrum of compound **3i**

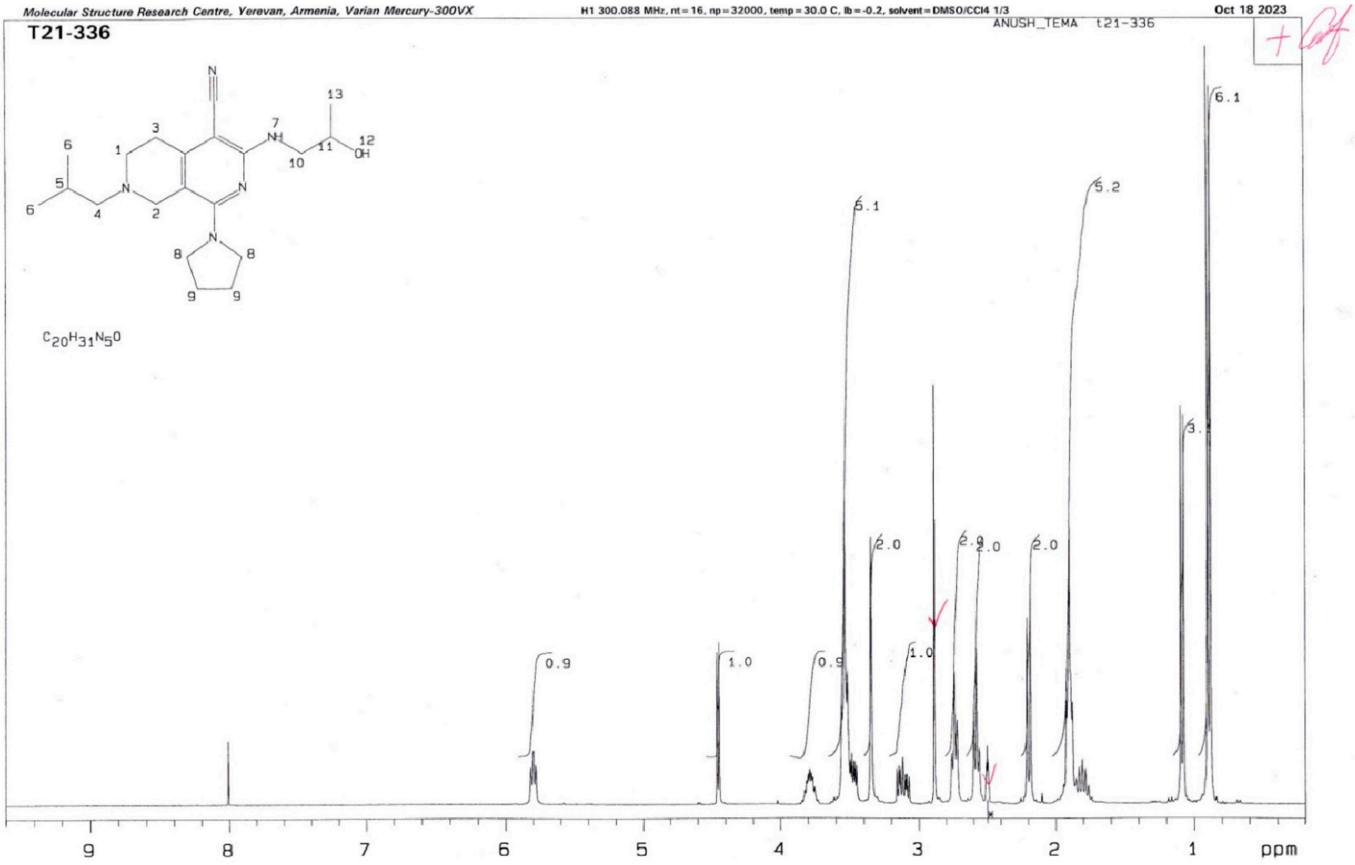

Figure S21. ^{1}H NMR spectrum of compound **3j**

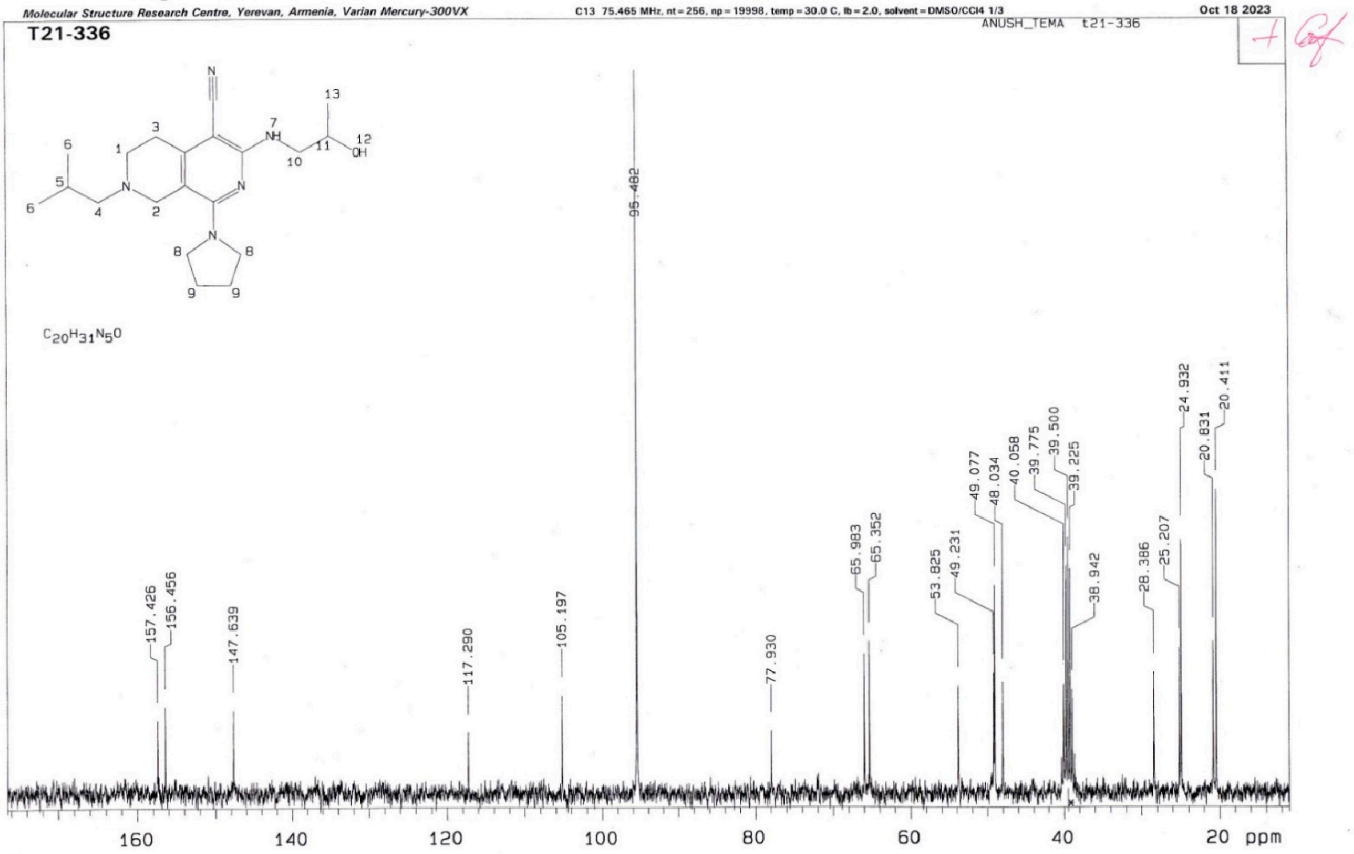

Figure S21.1. ^{13}C NMR spectrum of compound **3j**

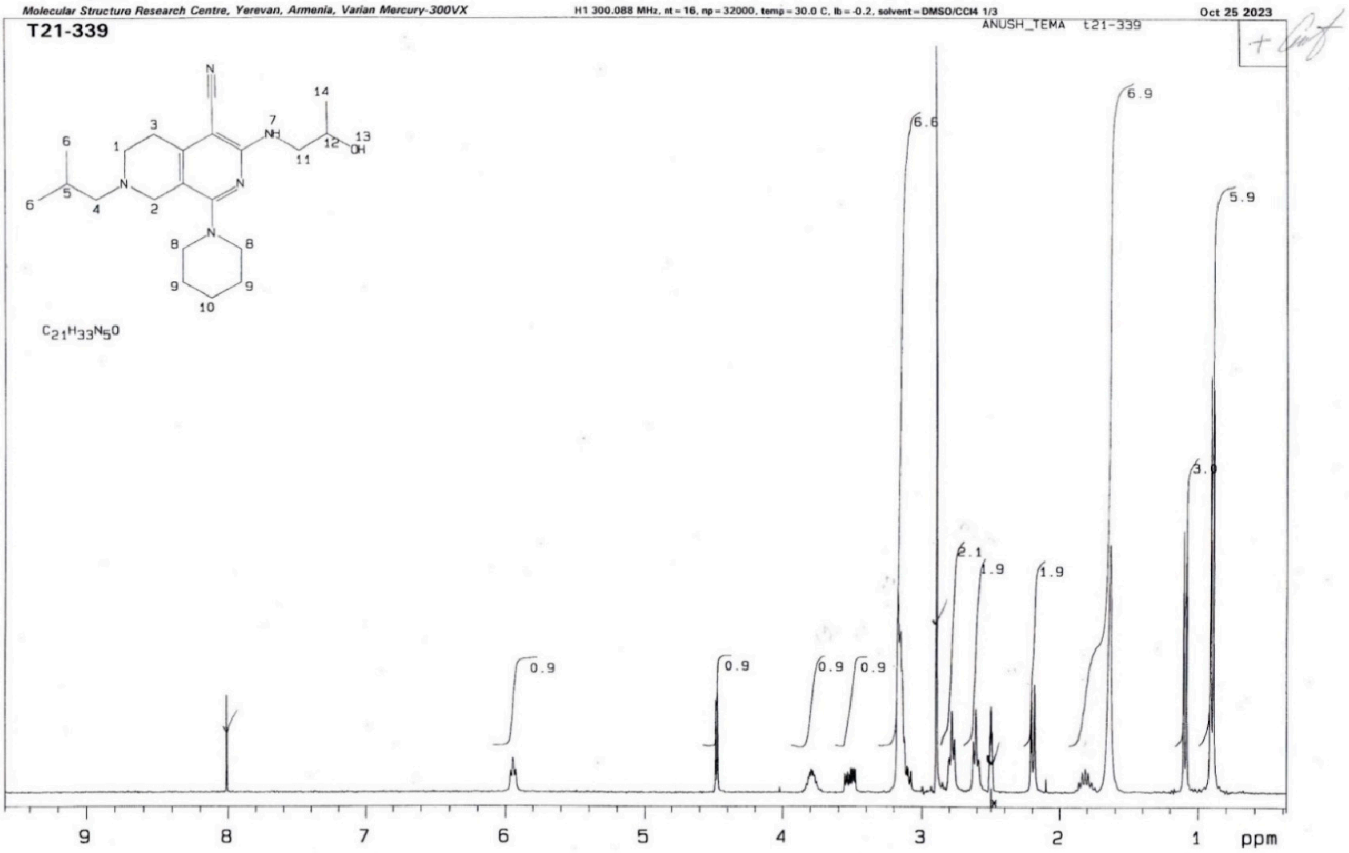

Figure S22. ^{1}H NMR spectrum of compound **3k**

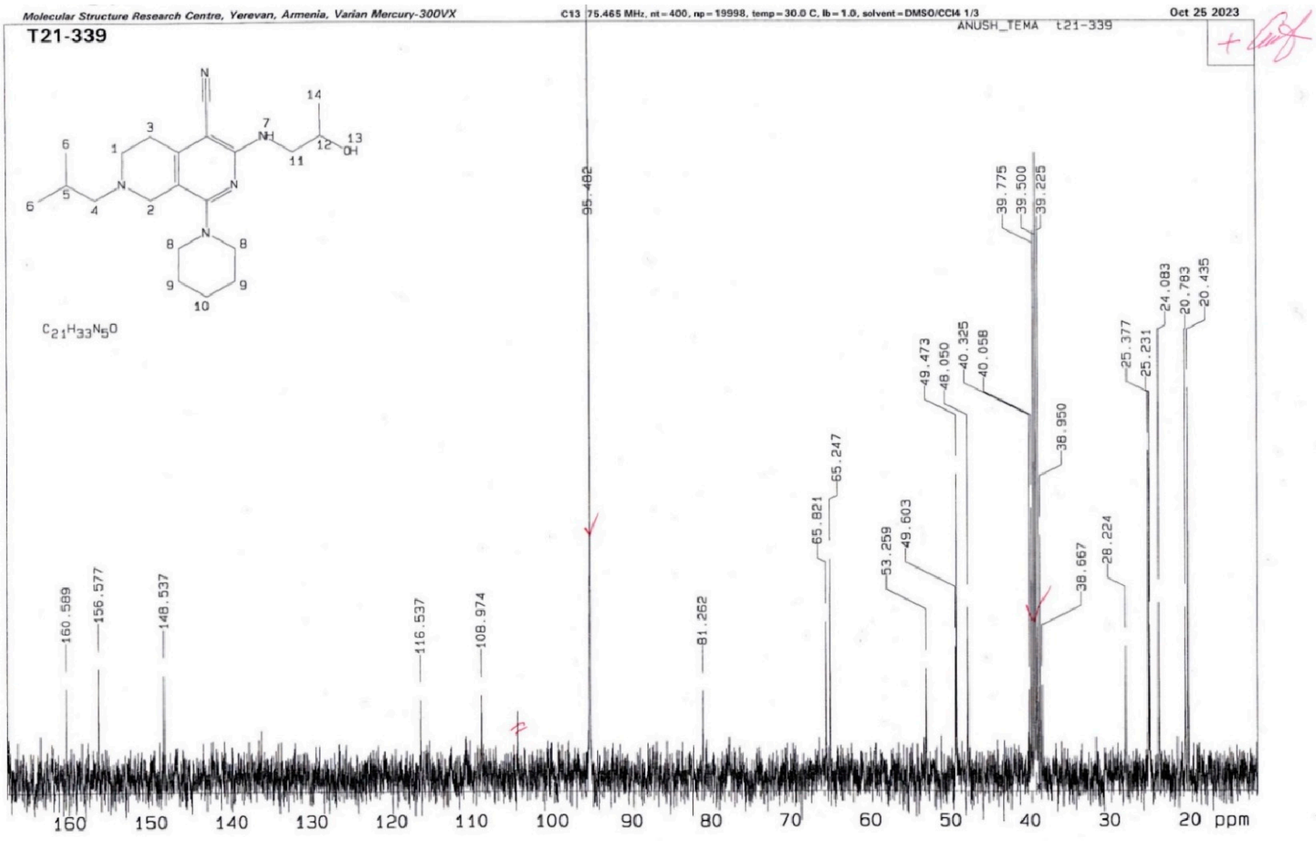

Figure S22.1. ^{13}C NMR spectrum of compound **3k**

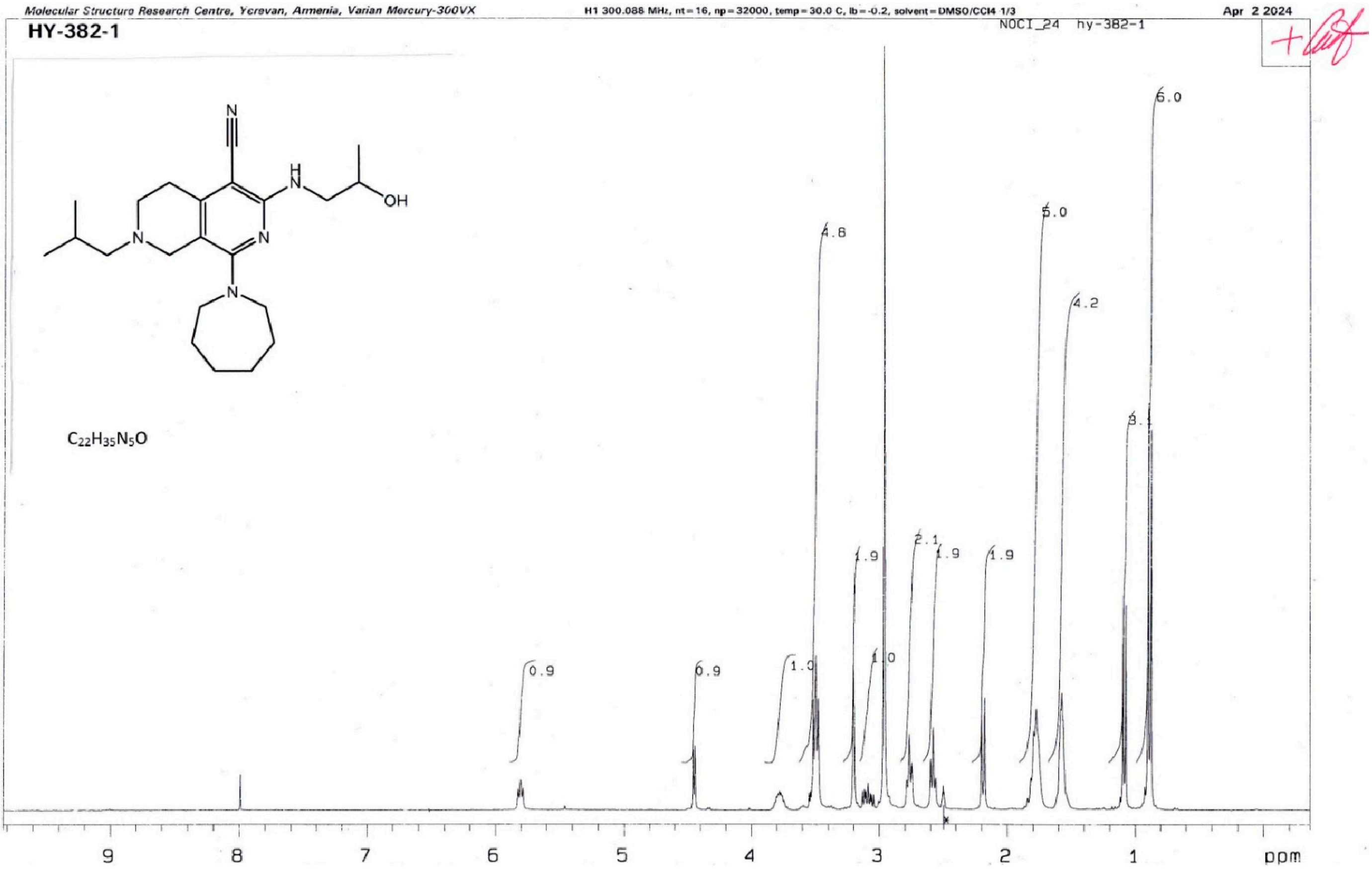

Figure S23. ^{1}H NMR spectrum of compound **3l**

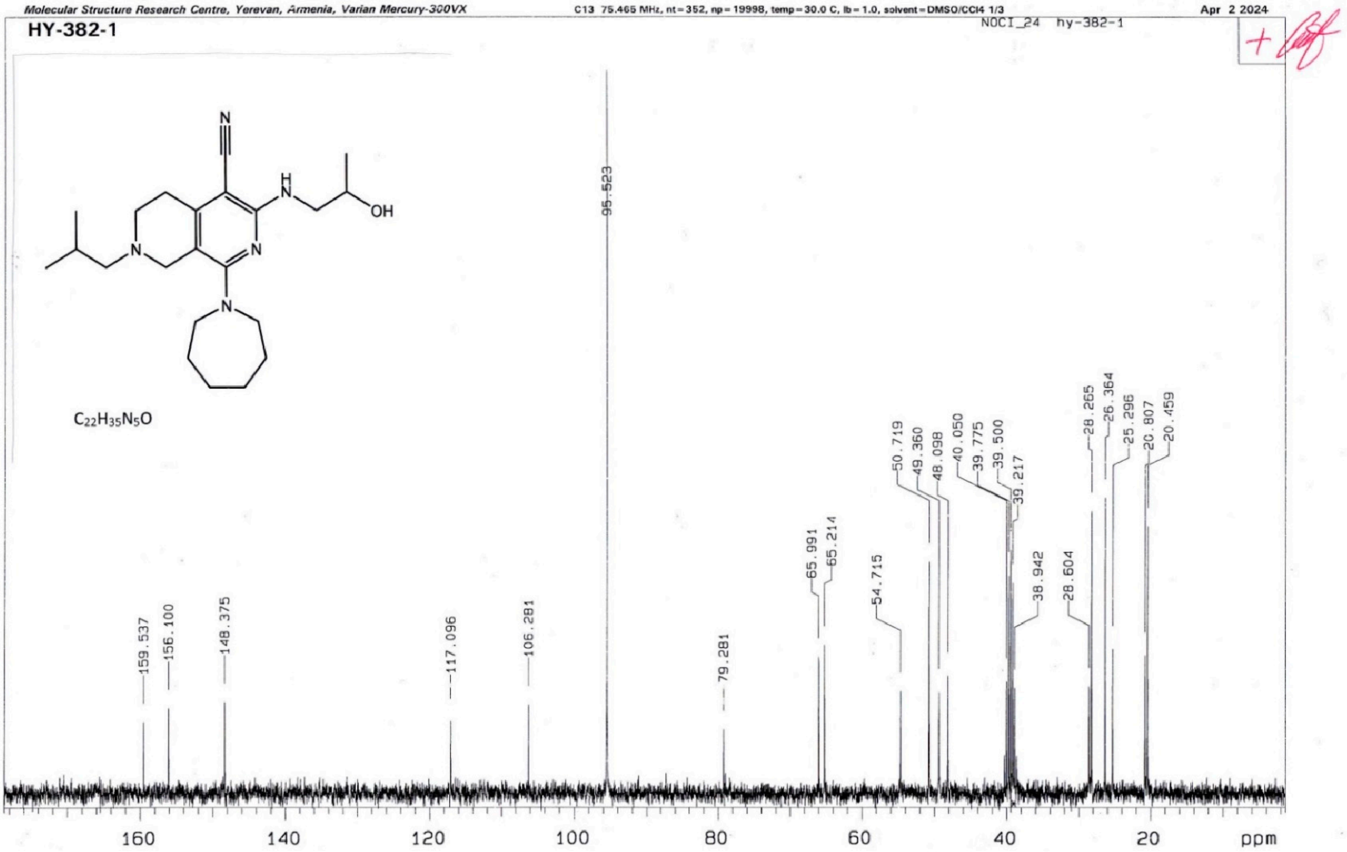

Figure S23.1. ^{13}C NMR spectrum of compound **3l**

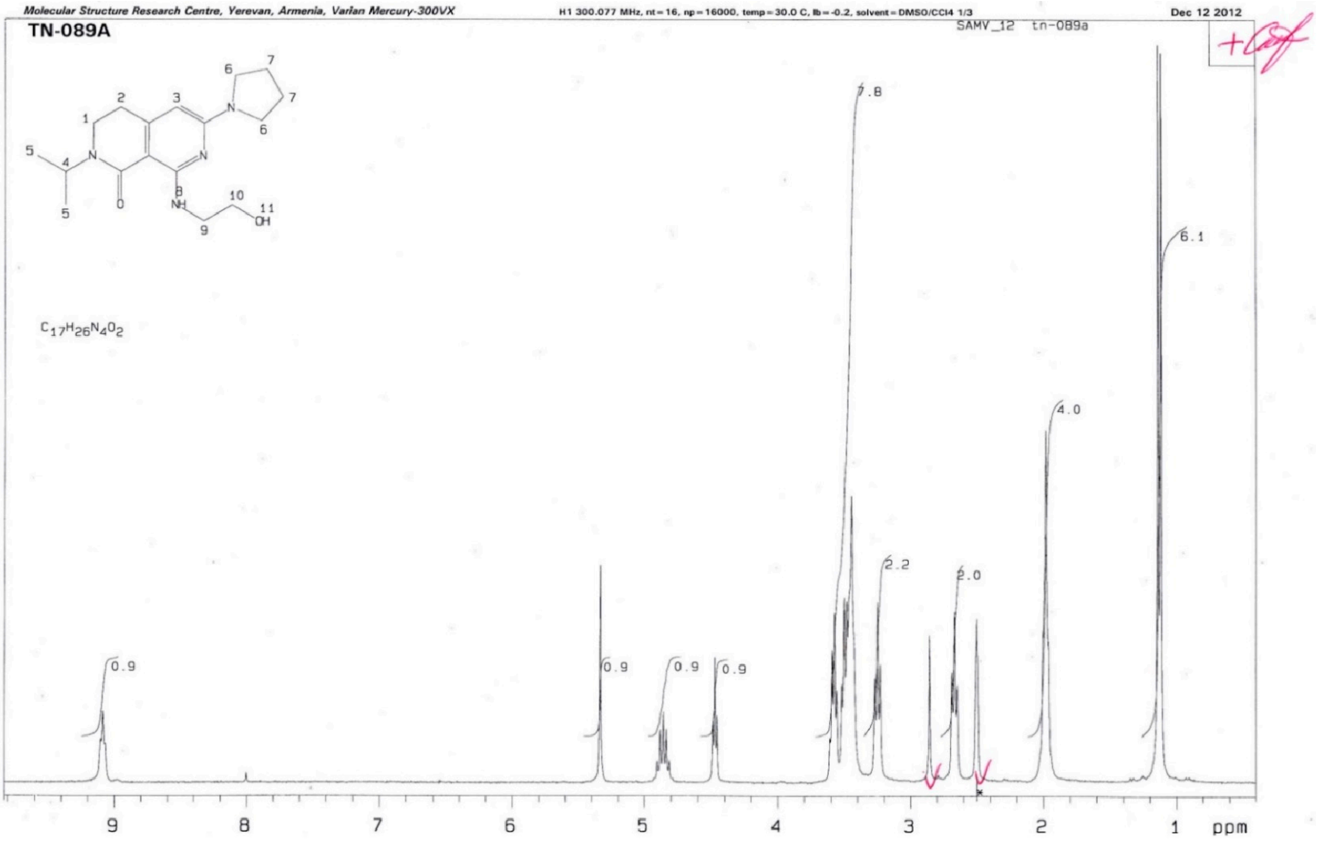

Figure S24. ^{1}H NMR spectrum of compound **4a**

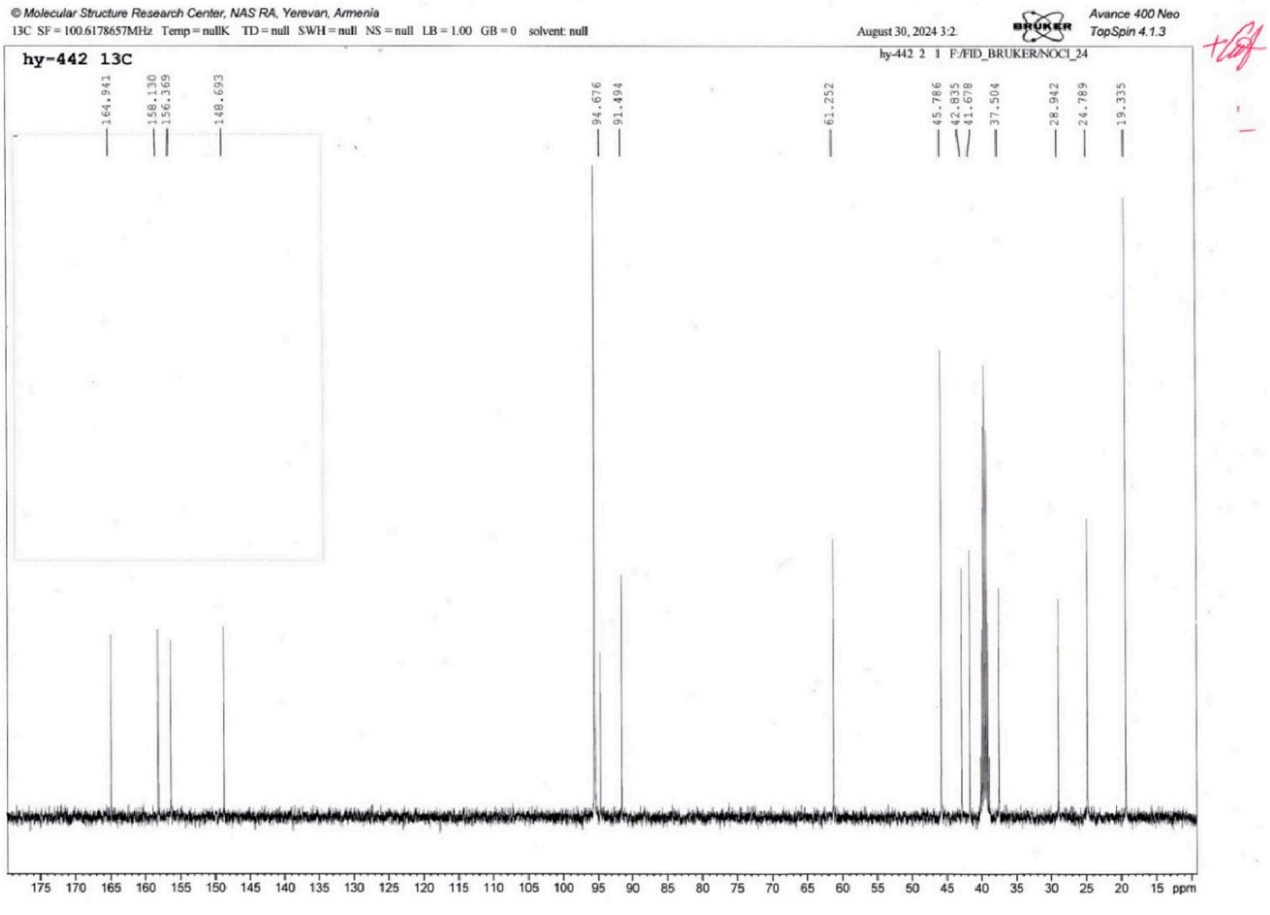

Figure S24.1. ^{13}C NMR spectrum of compound **4a**

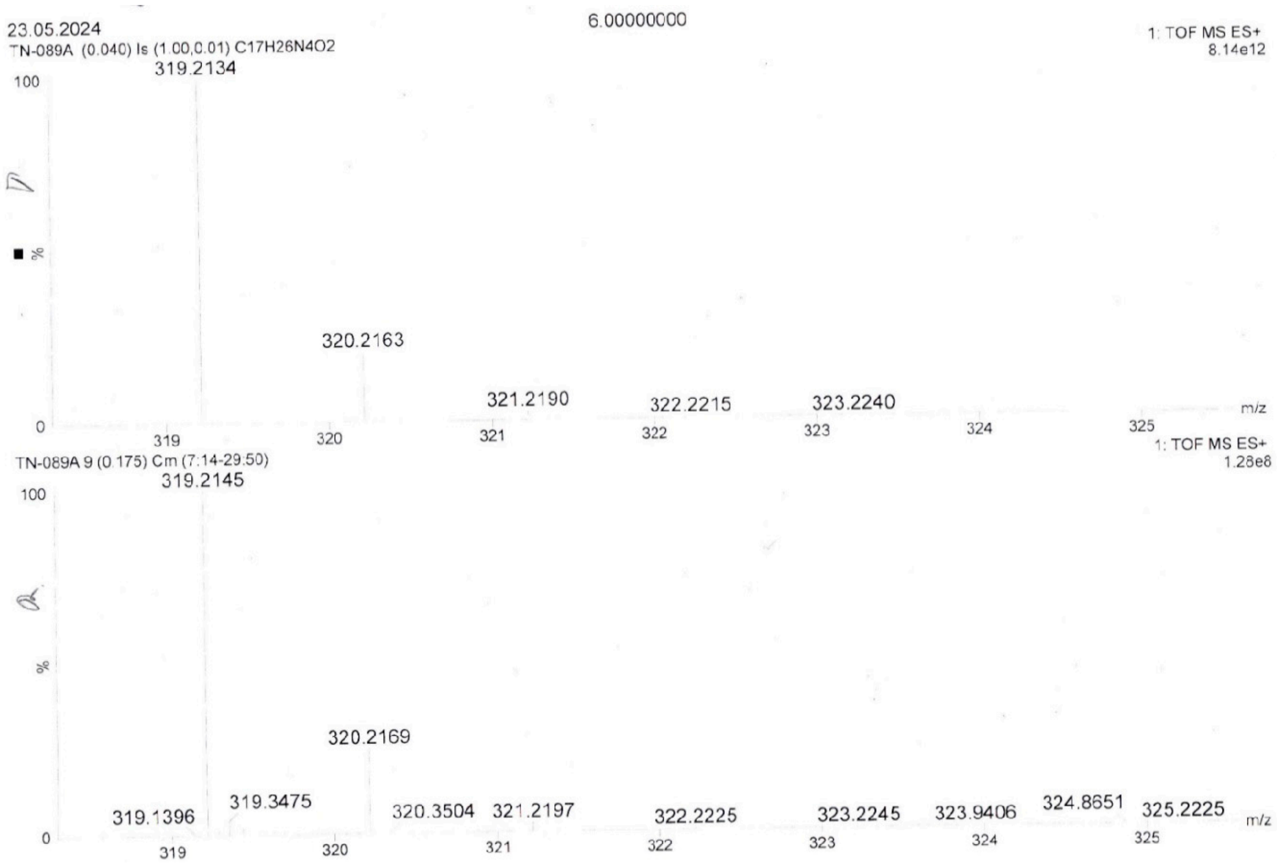

Figure S24.2. MS spectrum of compound **4a**

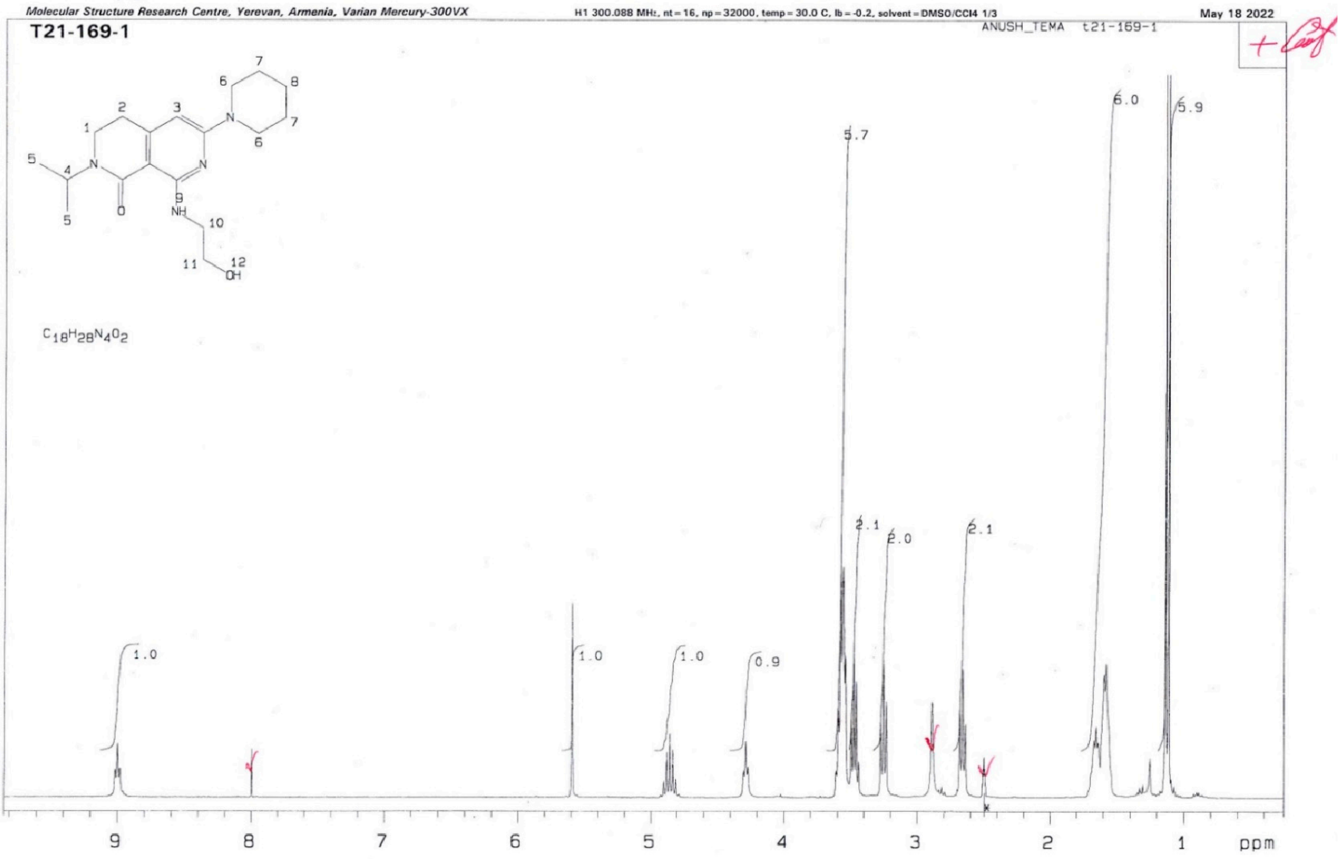

Figure S25. ^{1}H NMR spectrum of compound **4b**

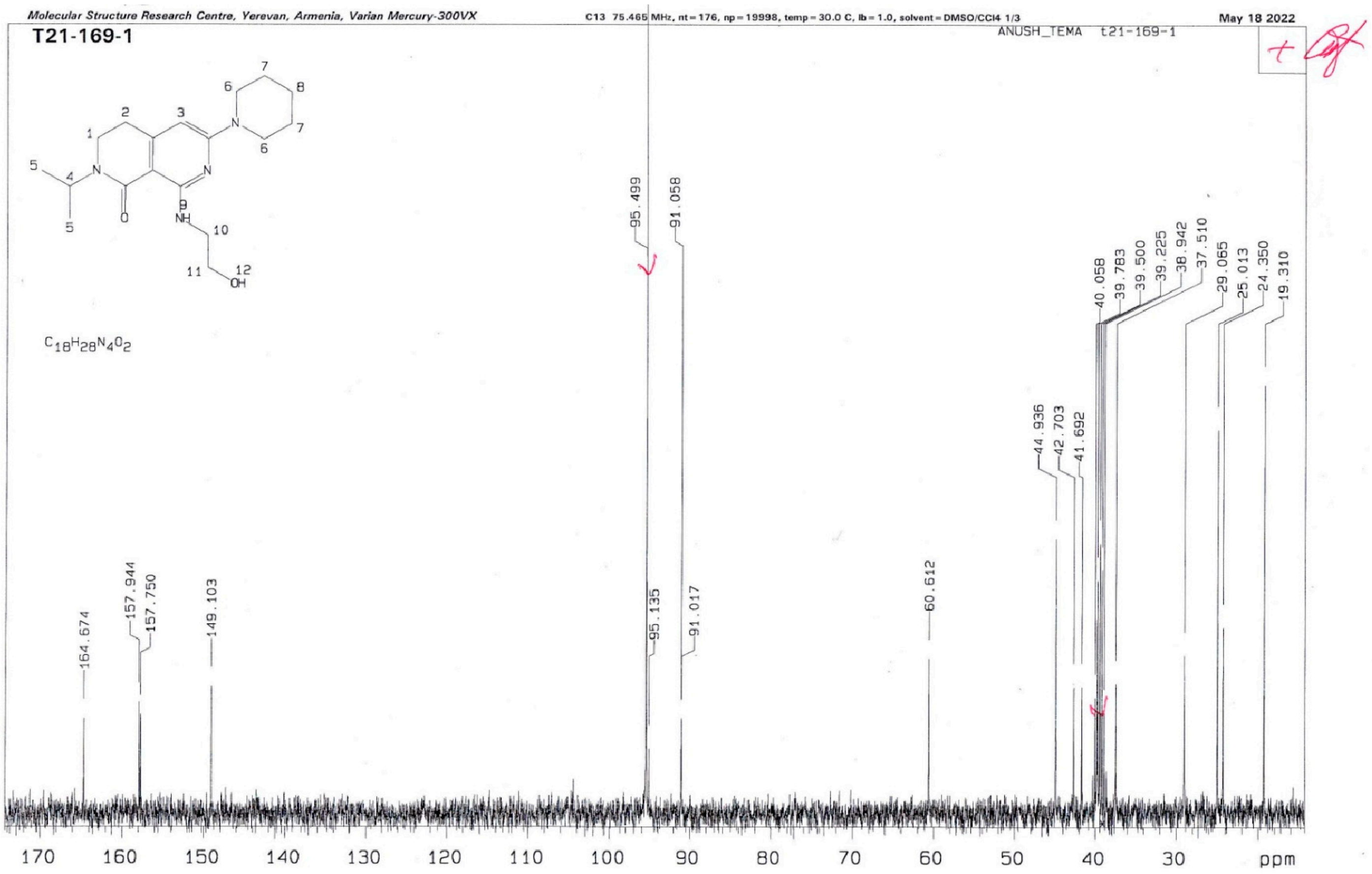

Figure S25.1. ^{13}C NMR spectrum of compound **4b**

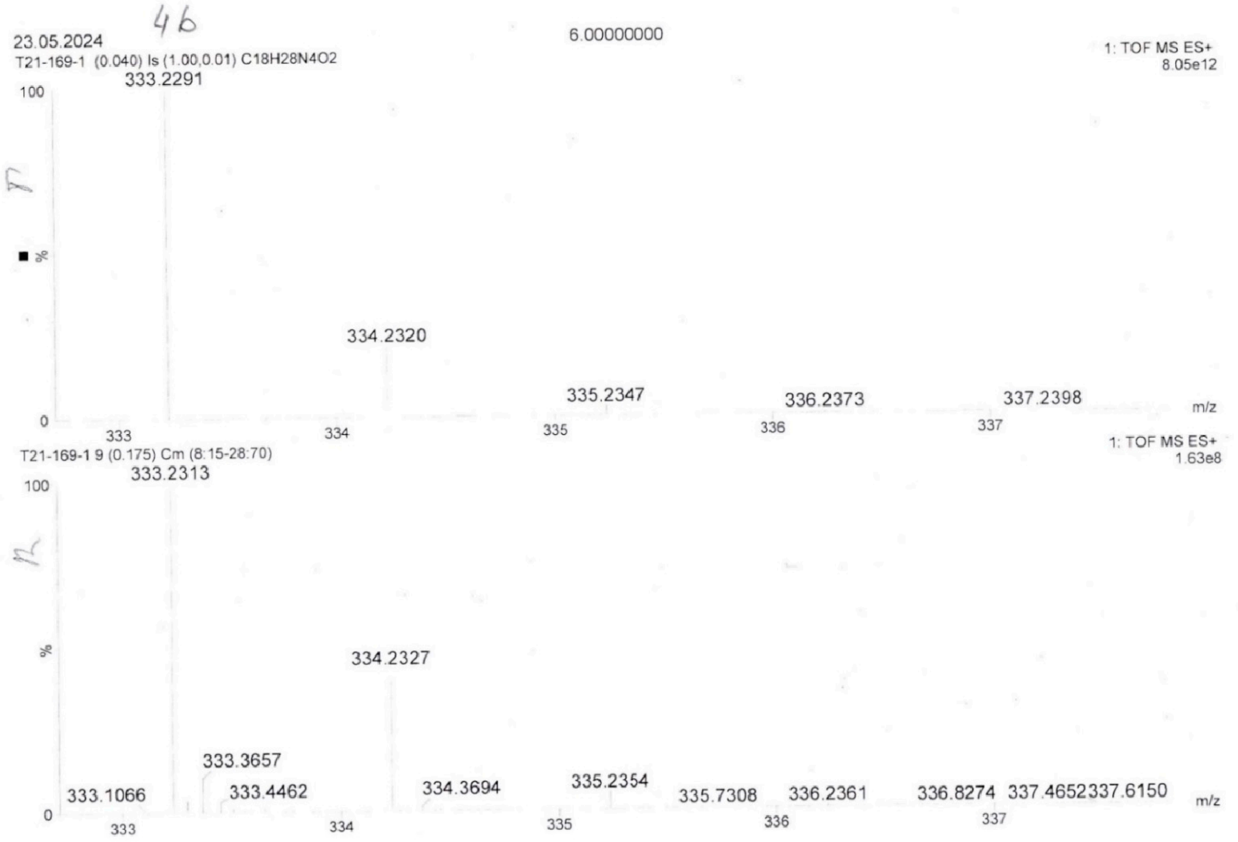

Figure S25.2. MS spectrum of compound **4b**

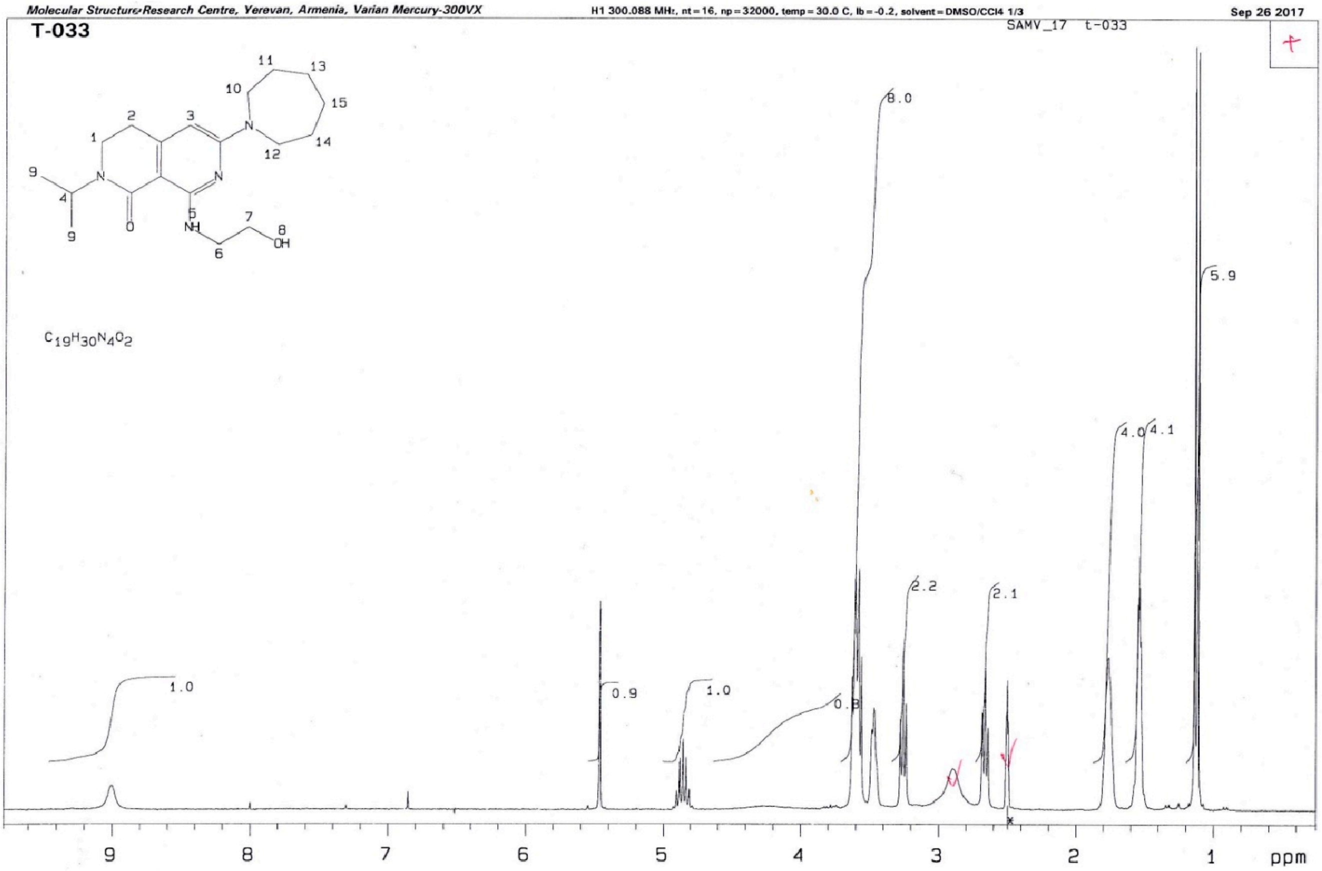

Figure S26. ^{1}H NMR spectrum of compound **4c**

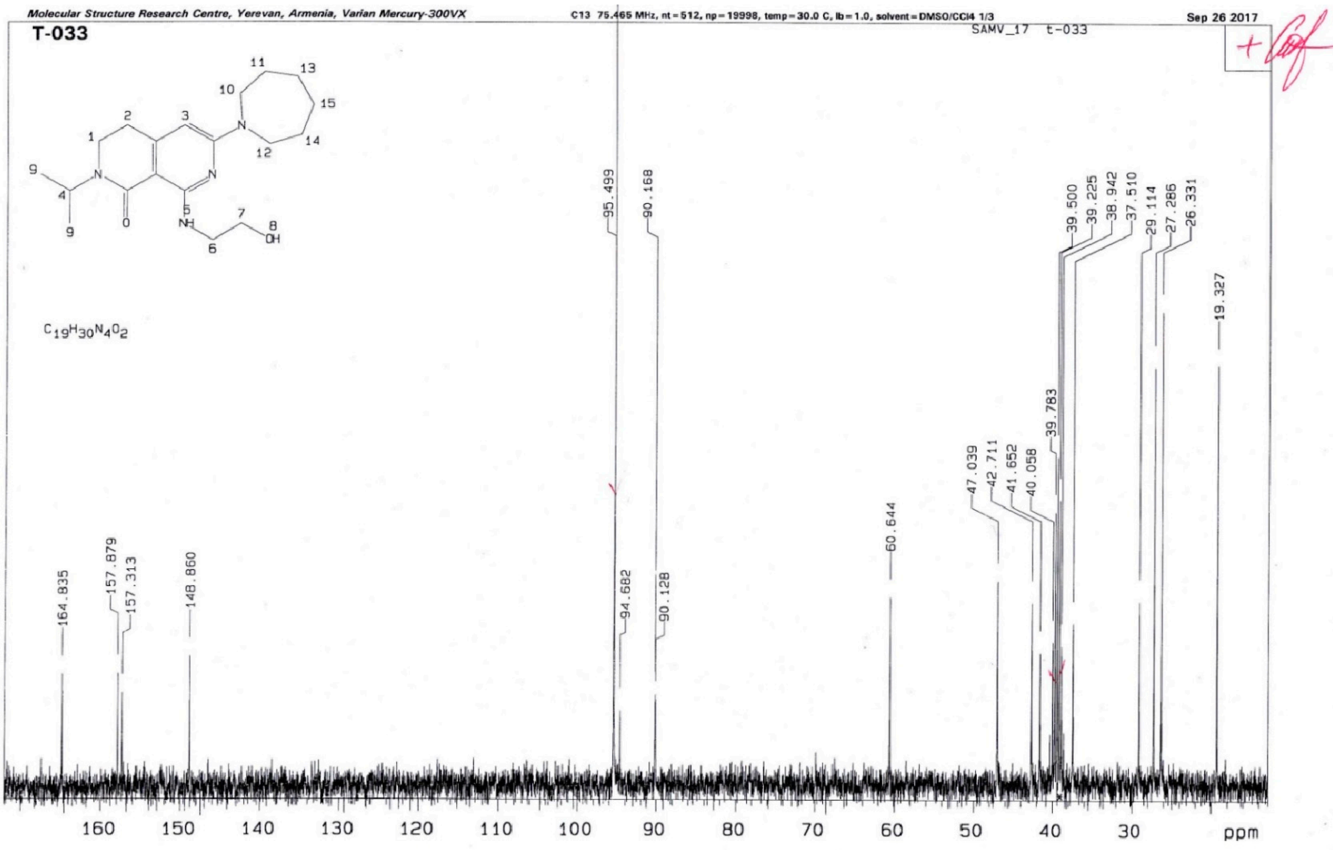

Figure S26.1. ^{13}C NMR spectrum of compound **4c**

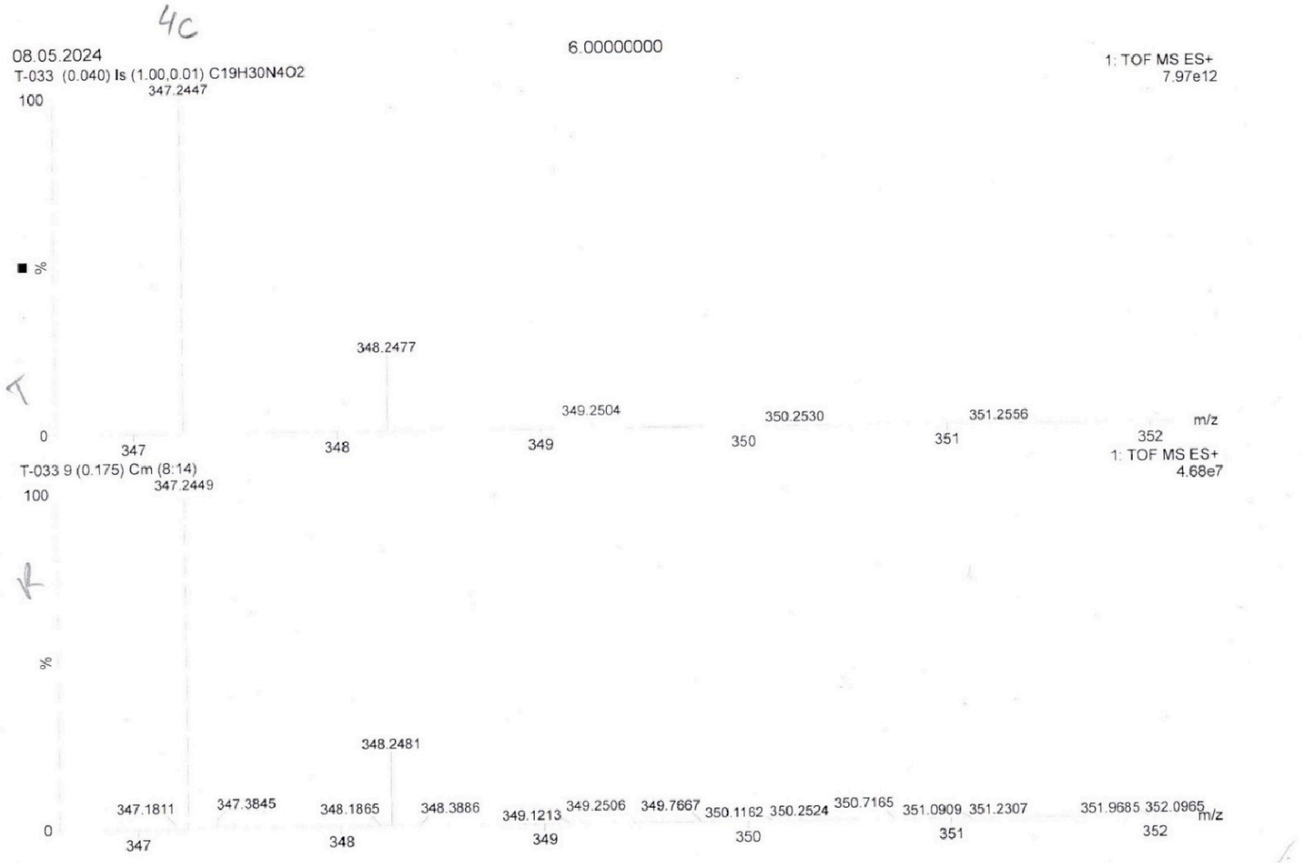

Figure S26.2. MS spectrum of compound **4c**

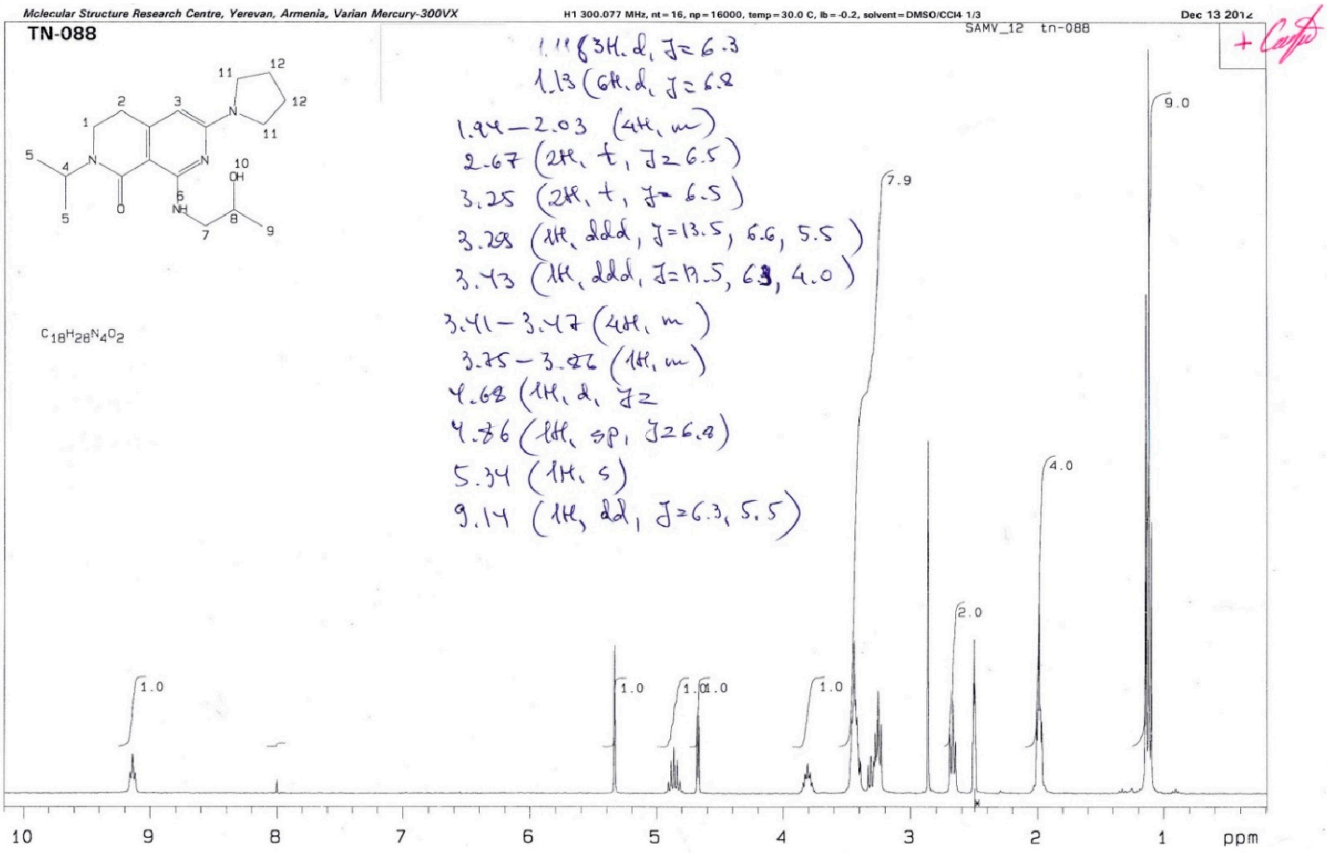

Figure S27. ^{1}H NMR spectrum of compound **4d**

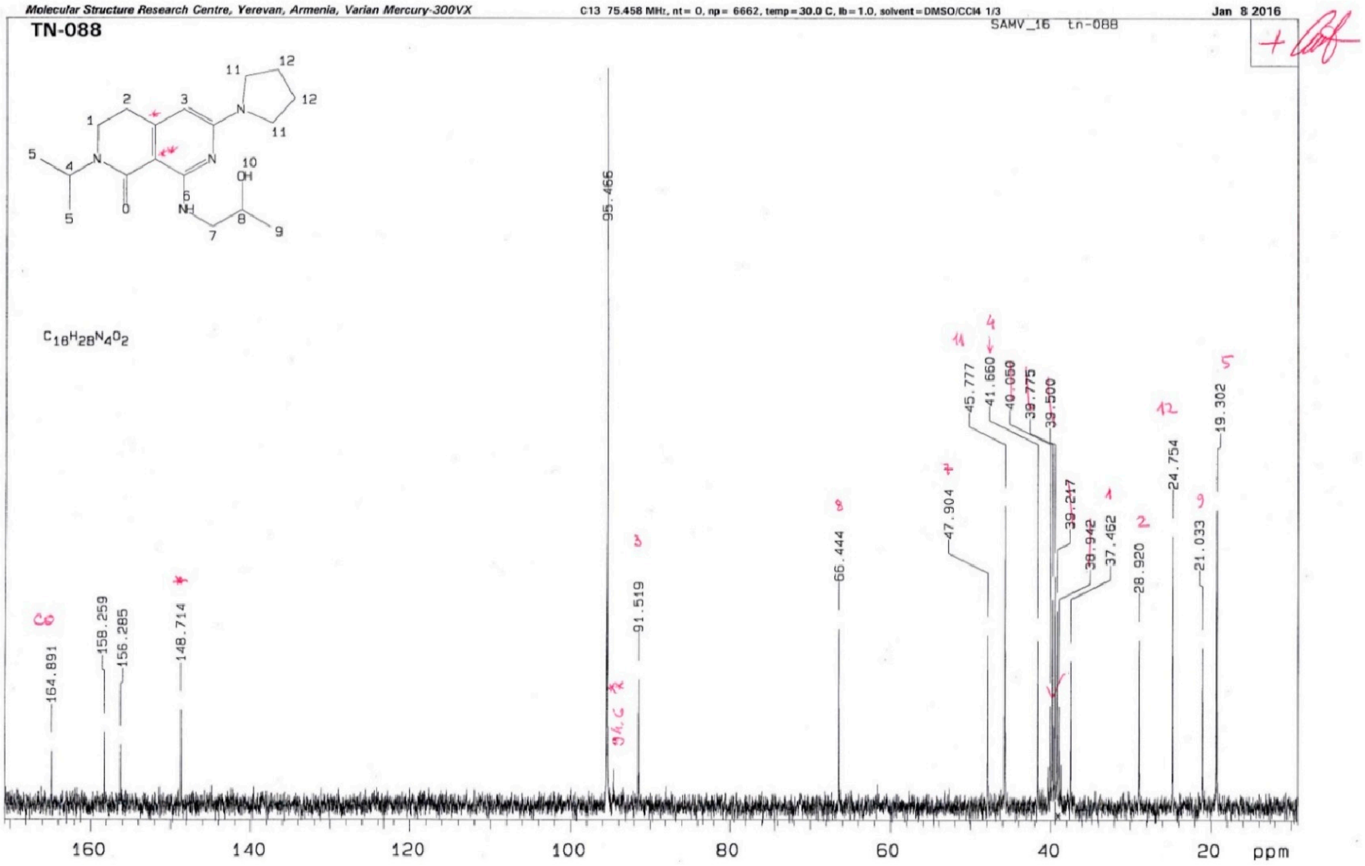

Figure S27.1. ^{13}C NMR spectrum of compound **4d**

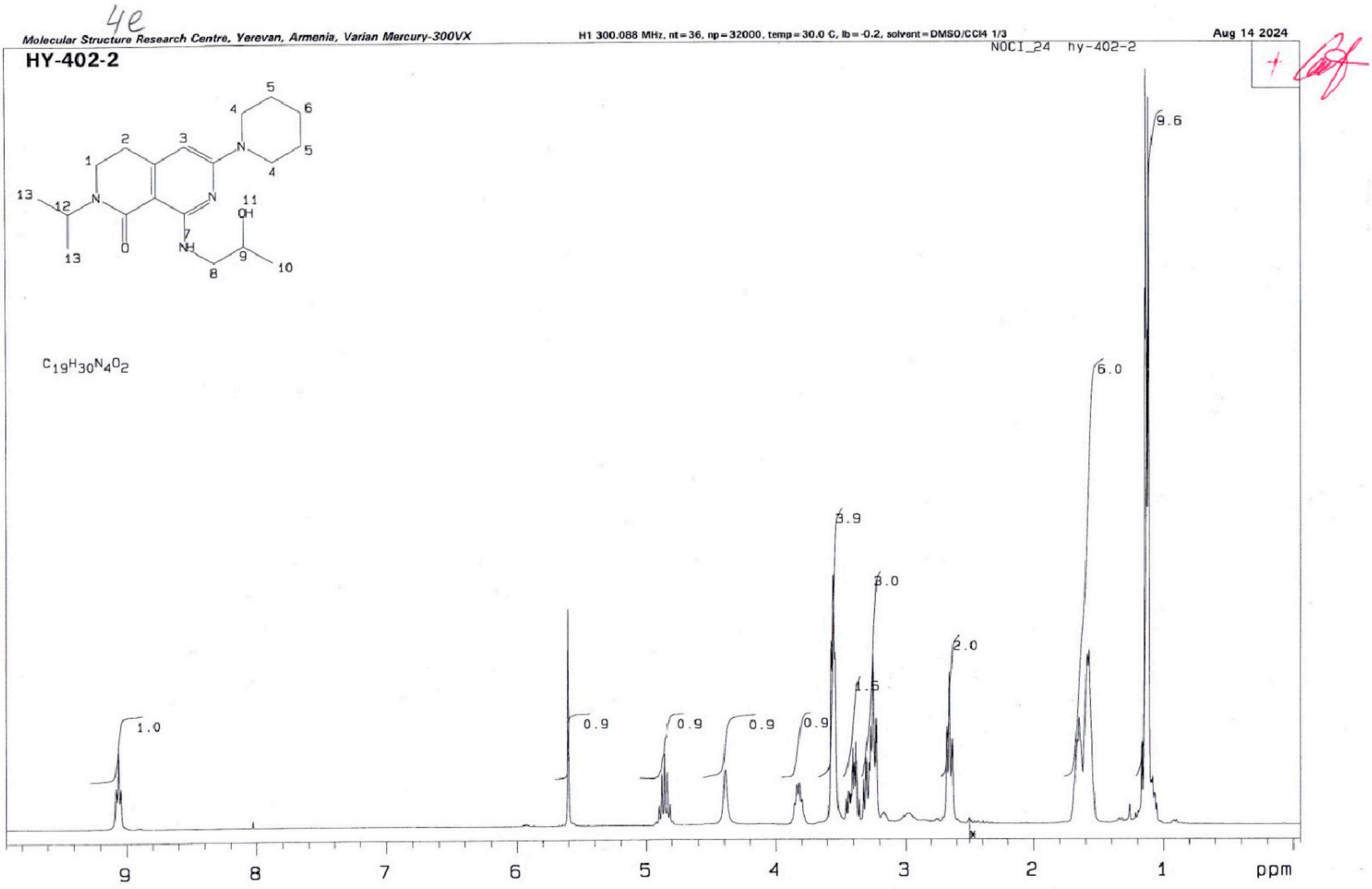

Figure S28. ^{1}H NMR spectrum of compound **4e**

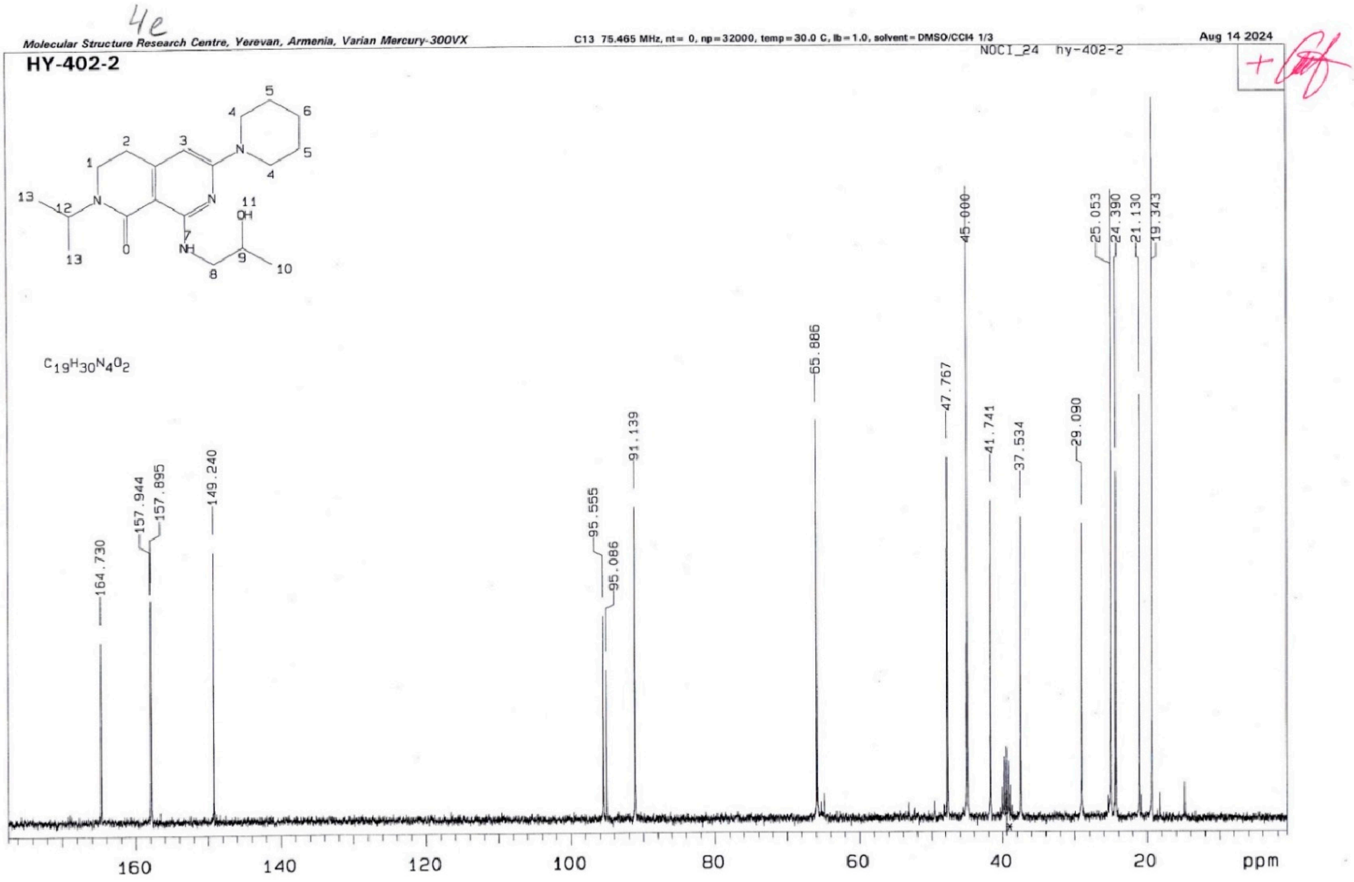

Figure S28.1. ^{13}C NMR spectrum of compound **4e**

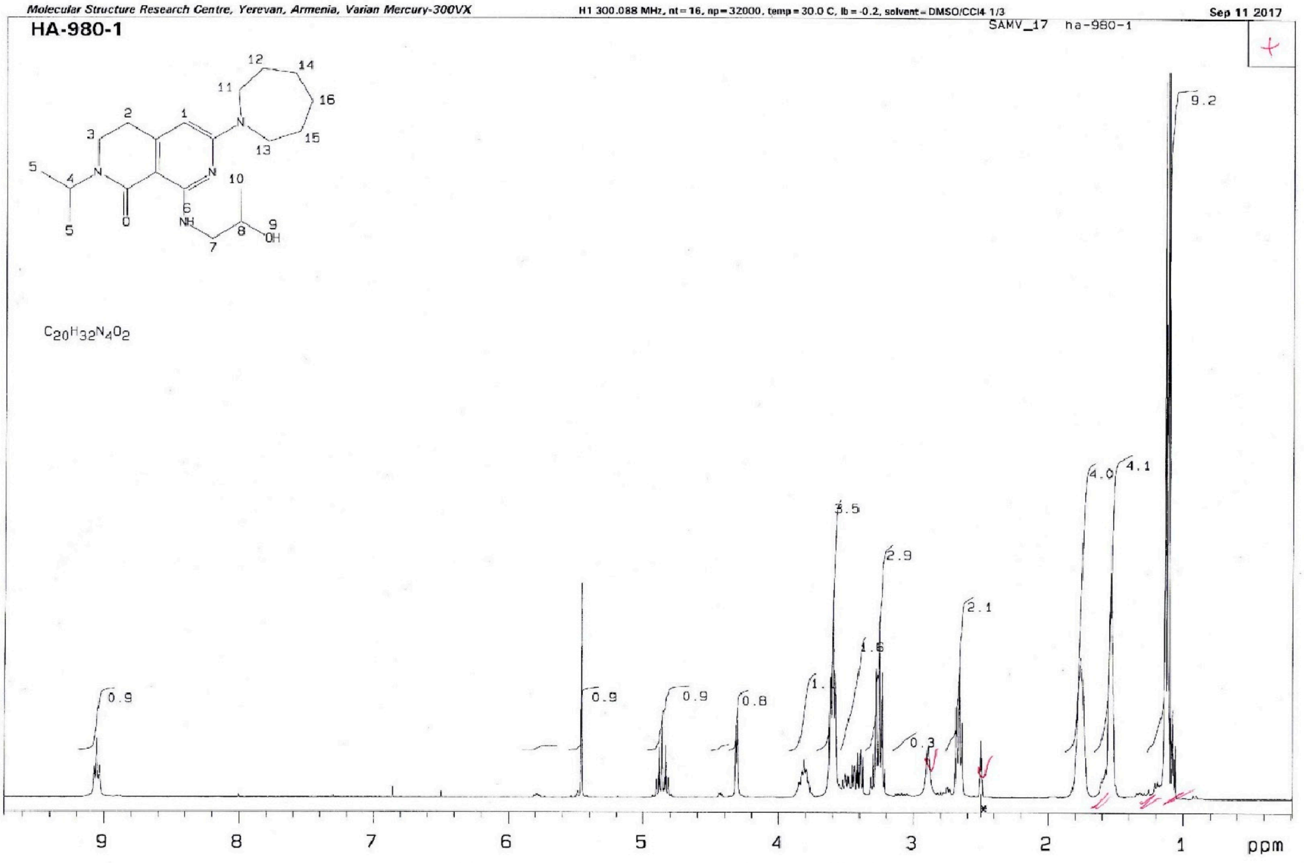

Figure S29. ^{1}H NMR spectrum of compound **4f**

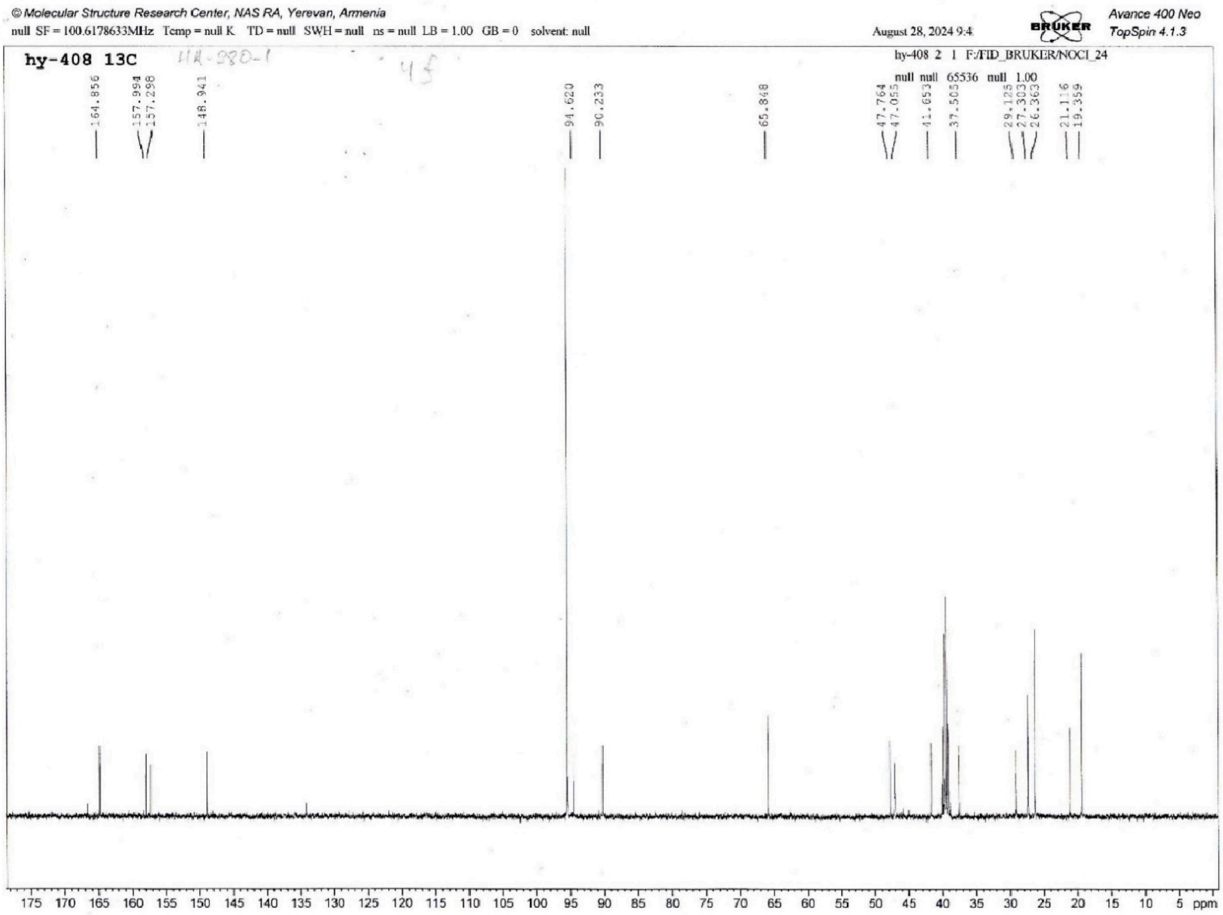

Figure S29.1. ^{13}C NMR spectrum of compound **4f**

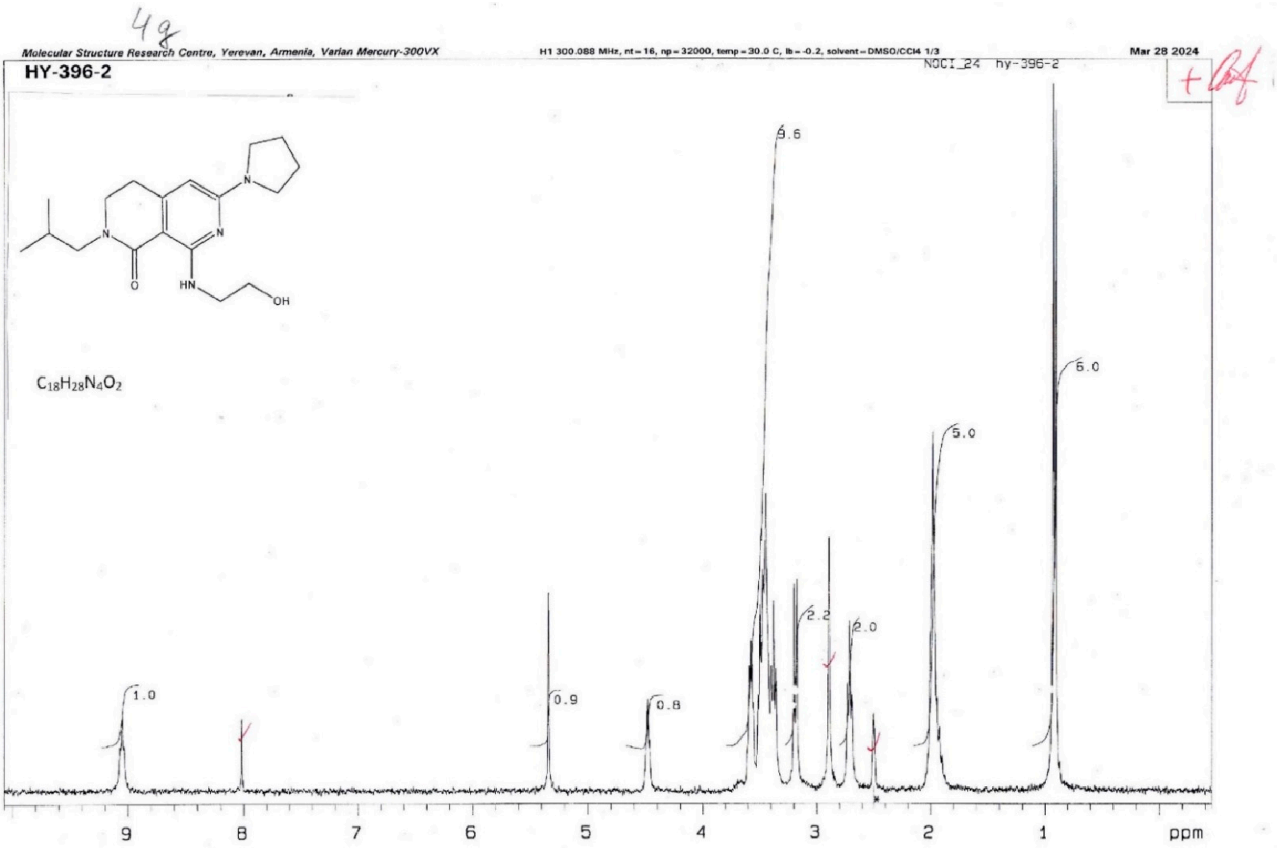

Figure S30. ^{1}H NMR spectrum of compound **4g**

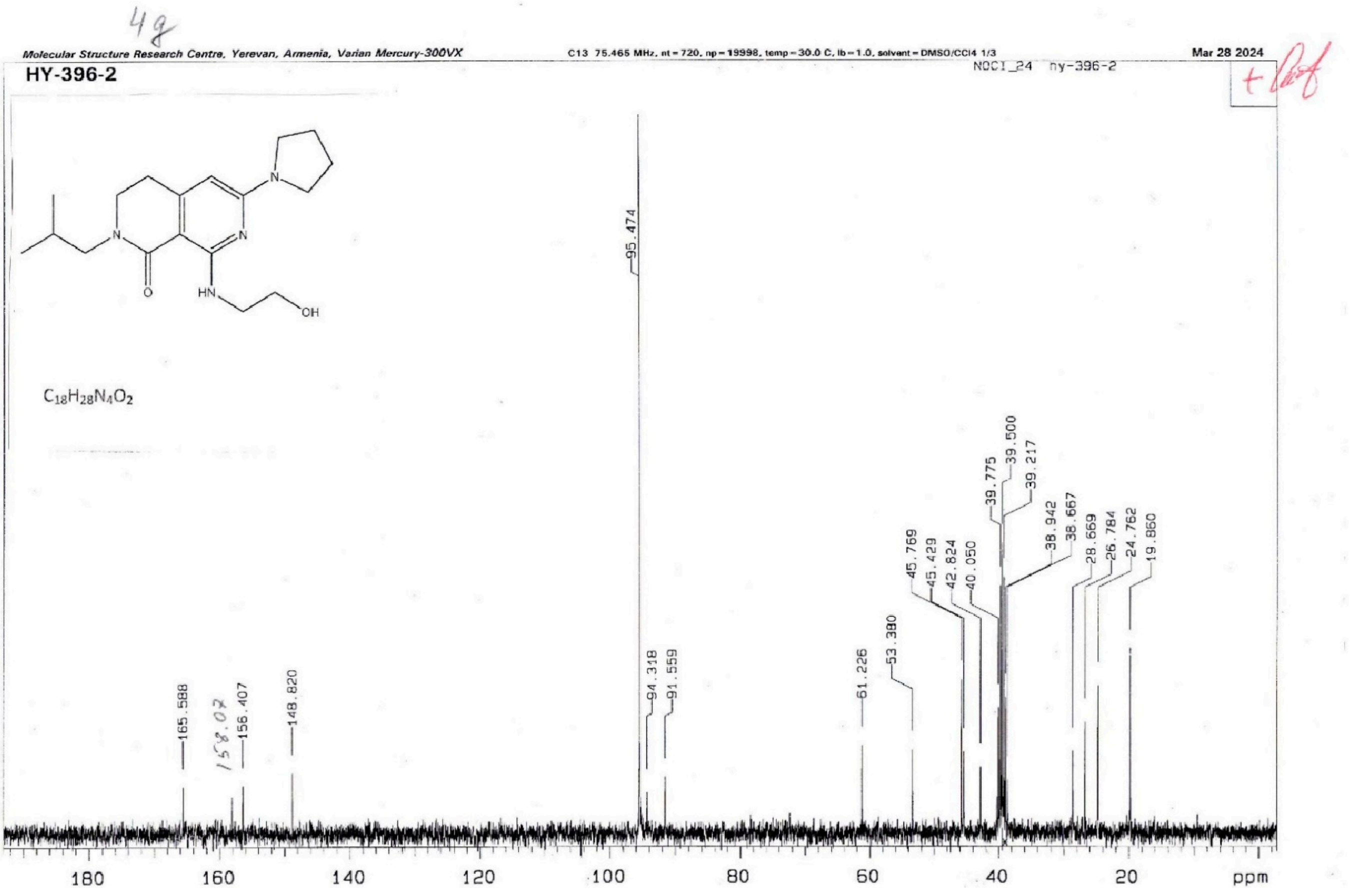

Figure S30.1. ^{13}C NMR spectrum of compound **4g**

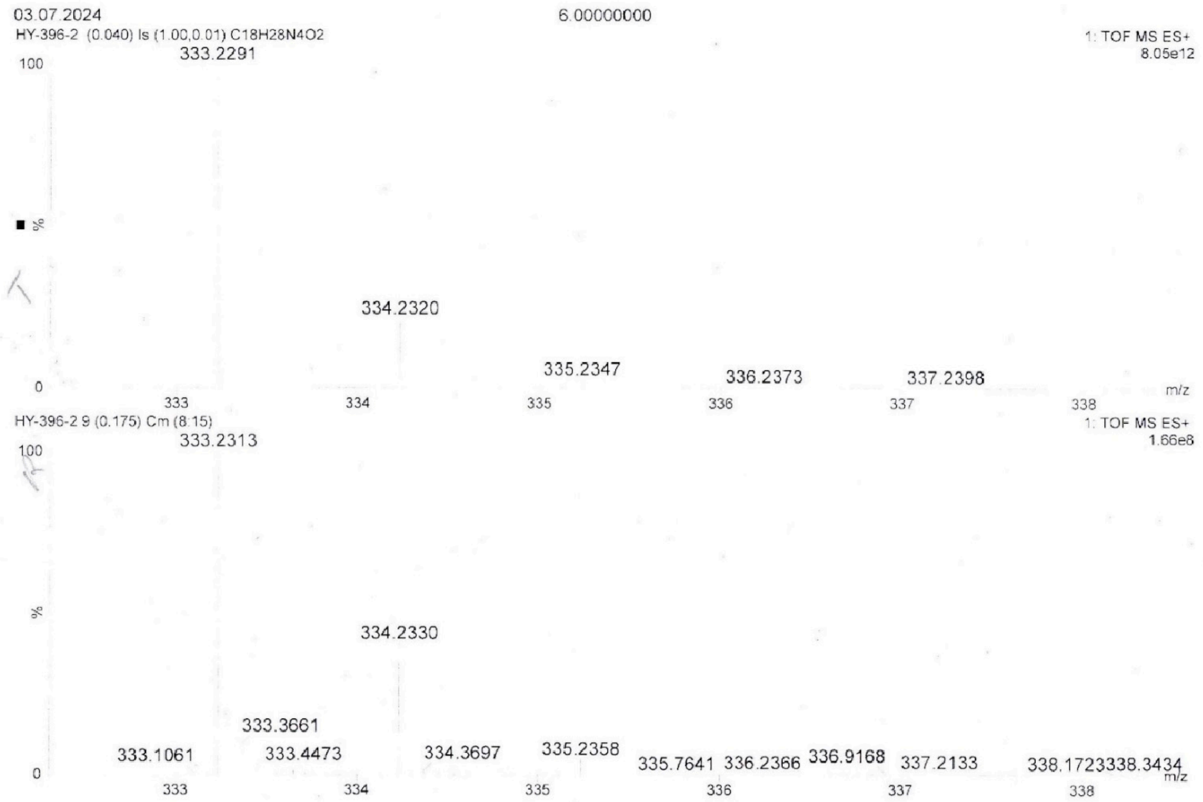

Figure S30.2. MS spectrum of compound **4g**

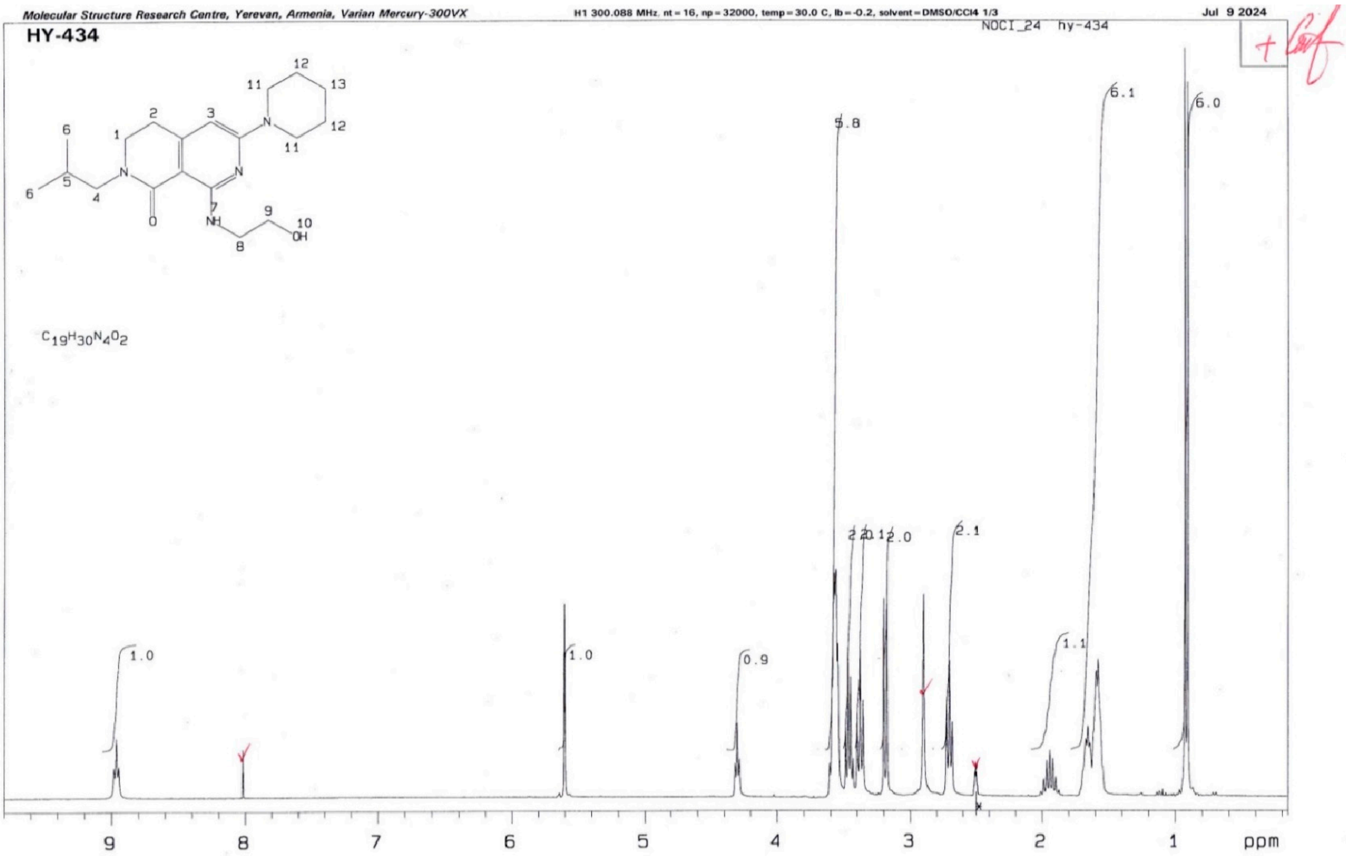

Figure S31. ^{1}H NMR spectrum of compound **4h**

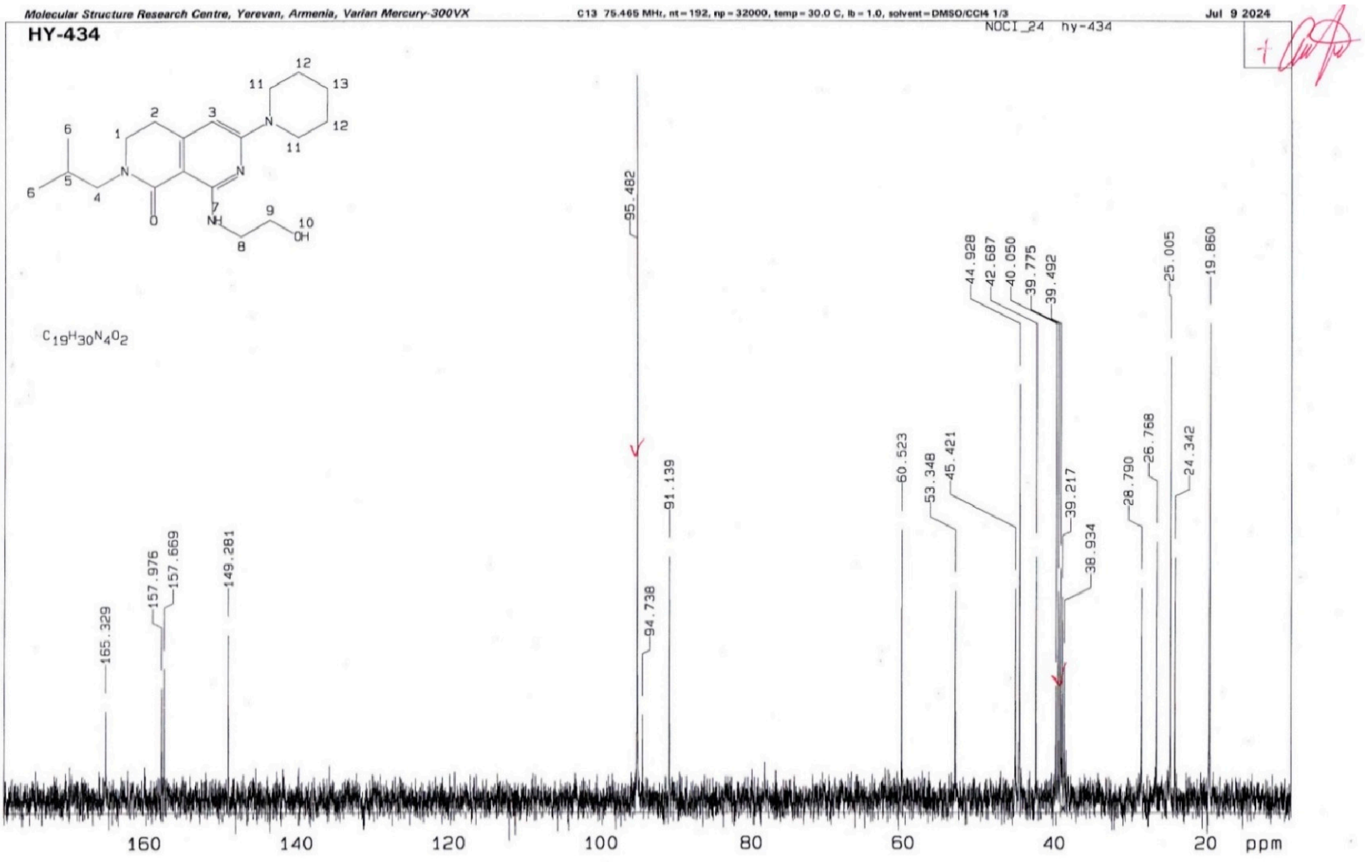

Figure S31.1. ^{13}C NMR spectrum of compound **4h**

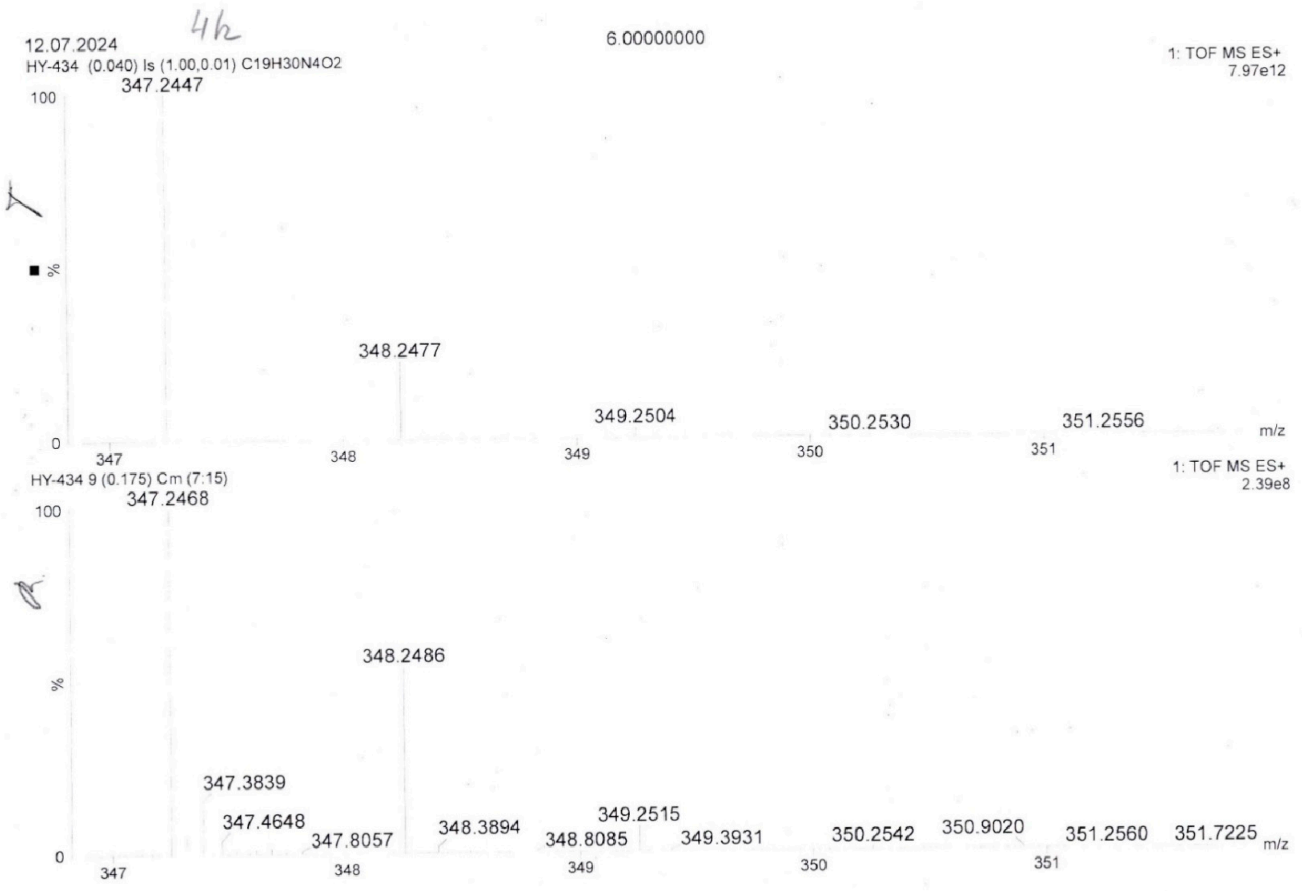

Figure S31.2. MS spectrum of compound **4h**

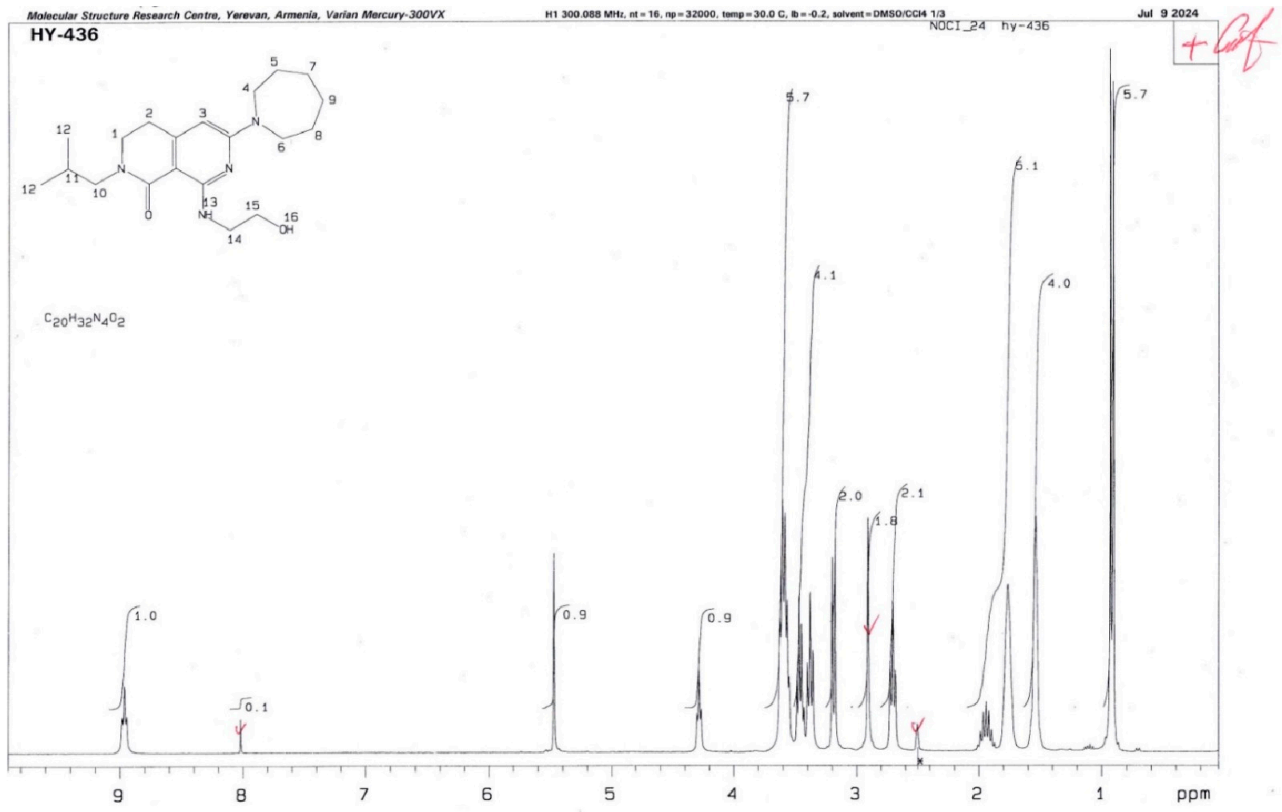

Figure S32. ^{1}H NMR spectrum of compound **4i**

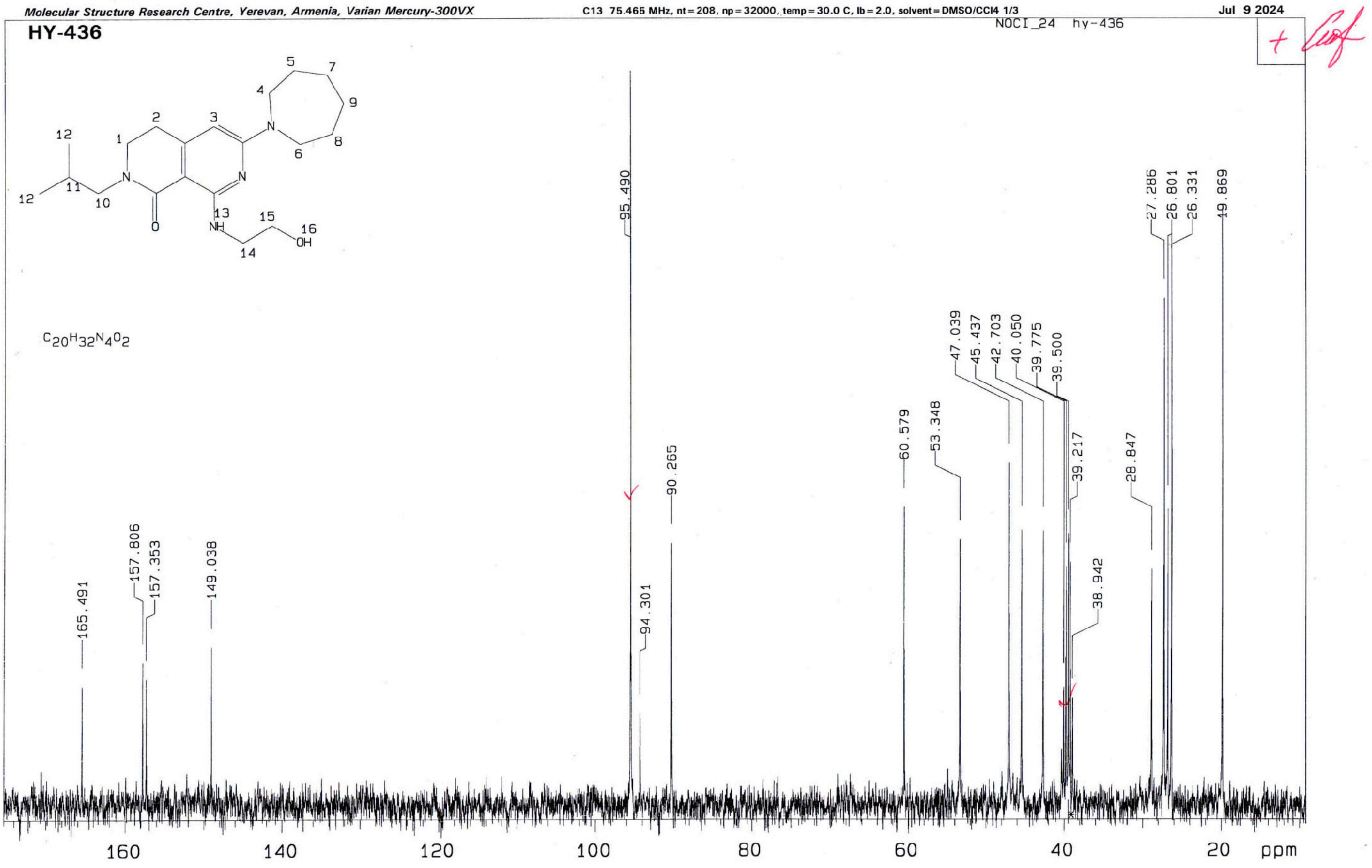

Figure S32.1. ^{13}C NMR spectrum of compound **4i**

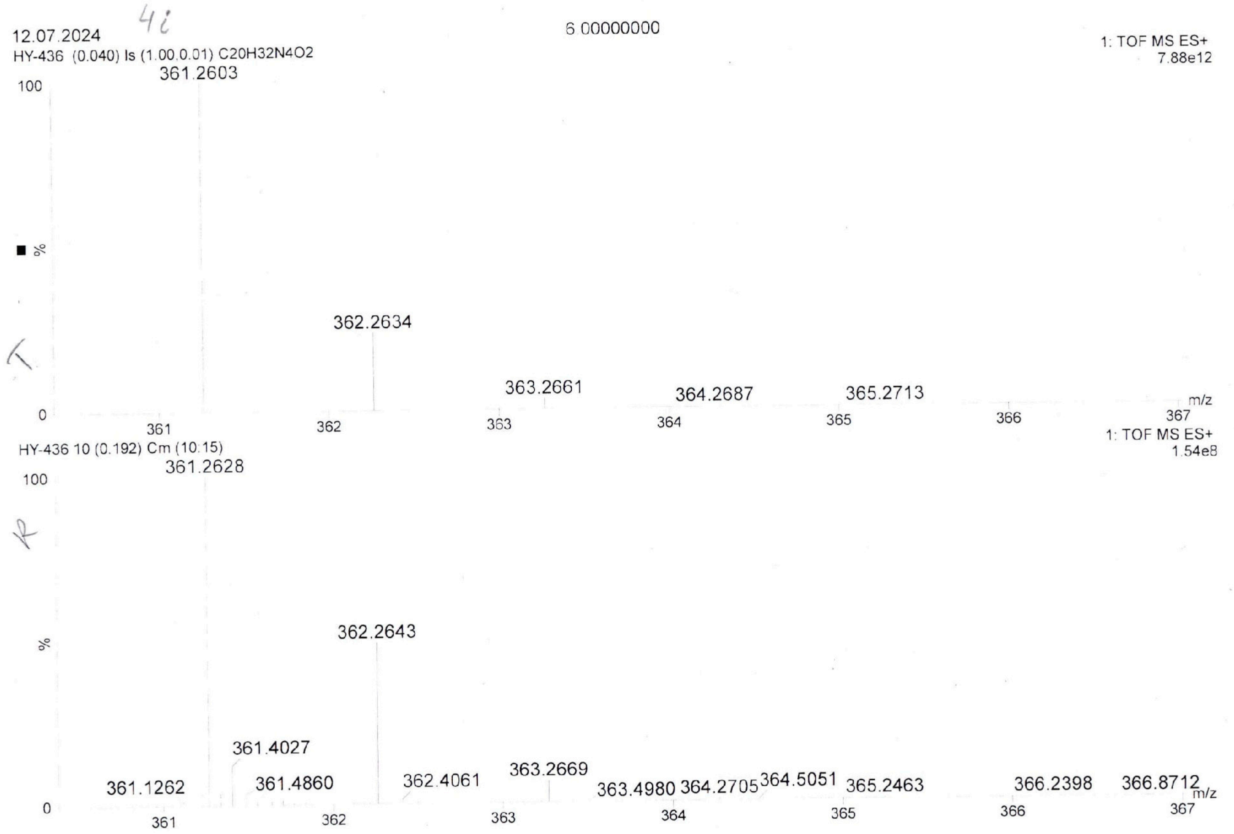

Figure S32.2. MS spectrum of compound **4i**

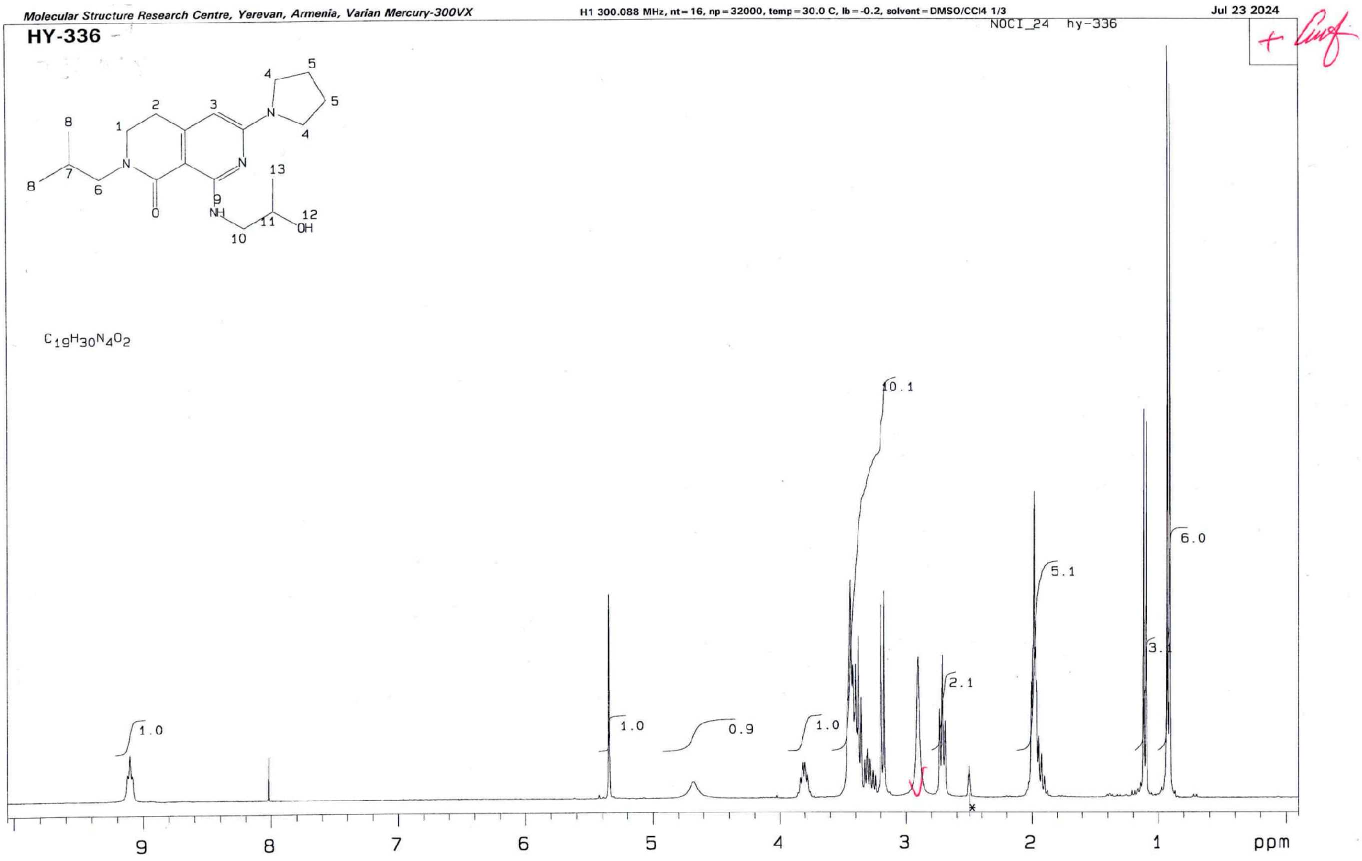

Figure S33. ^{1}H NMR spectrum of compound **4j**

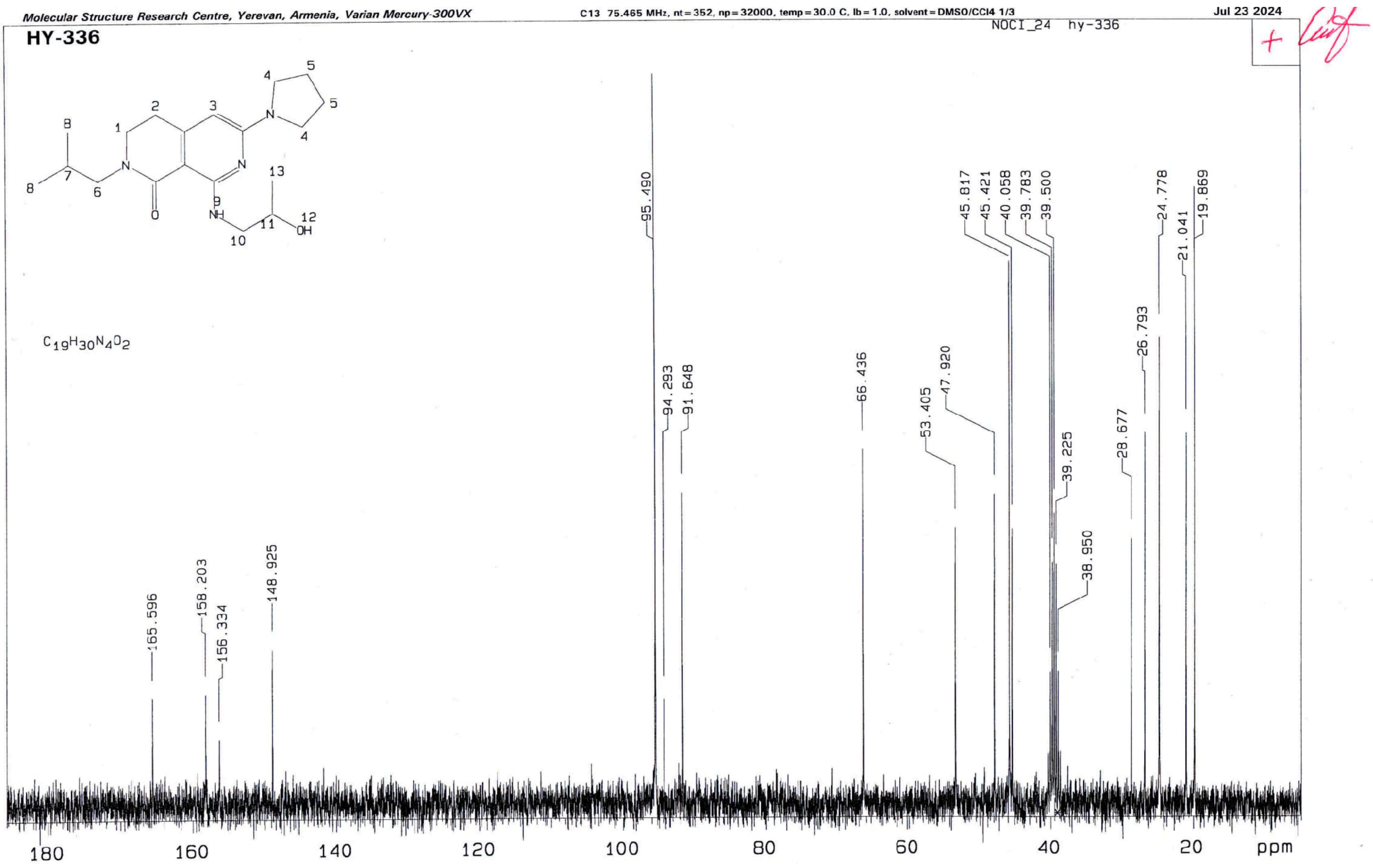

Figure S33.1. ^{13}C NMR spectrum of compound **4j**

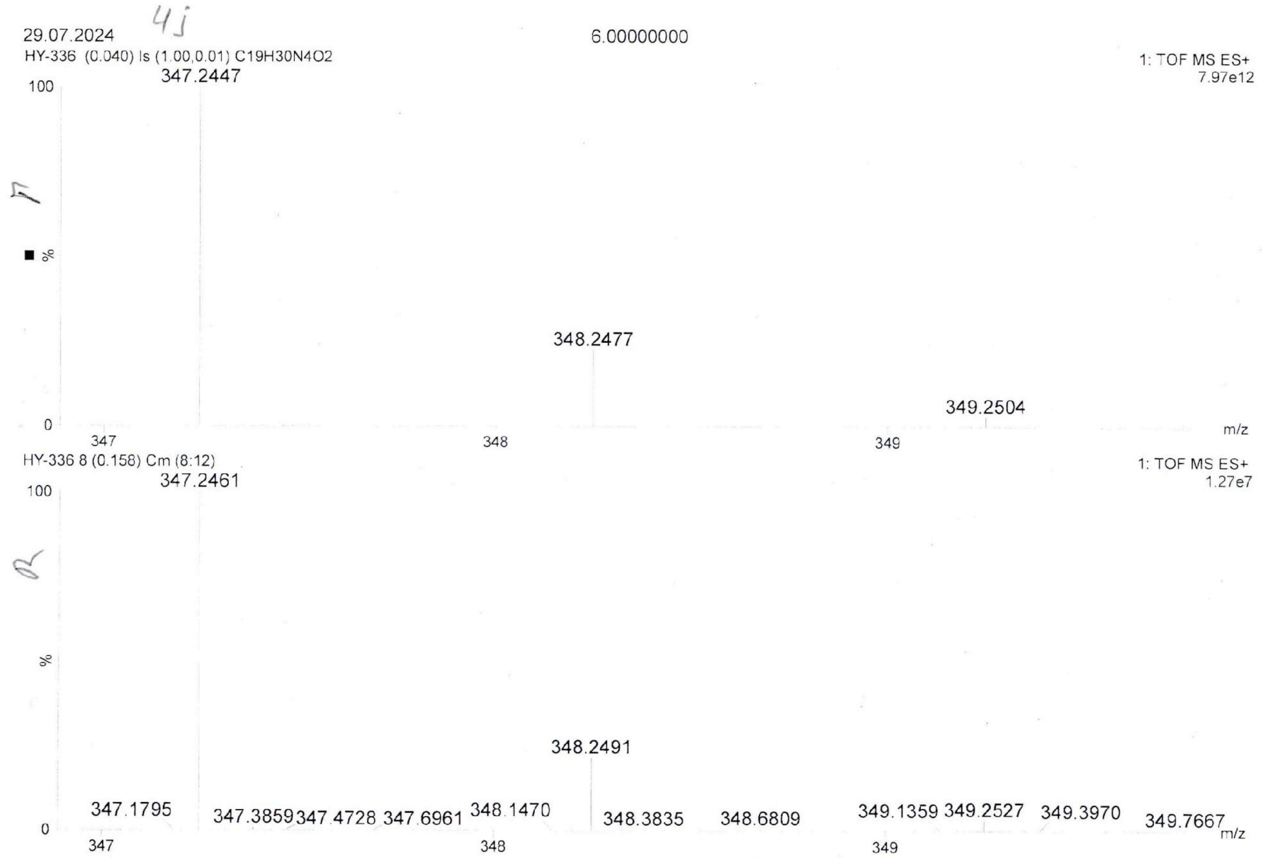

Figure S33.2. MS spectrum of compound **4j**

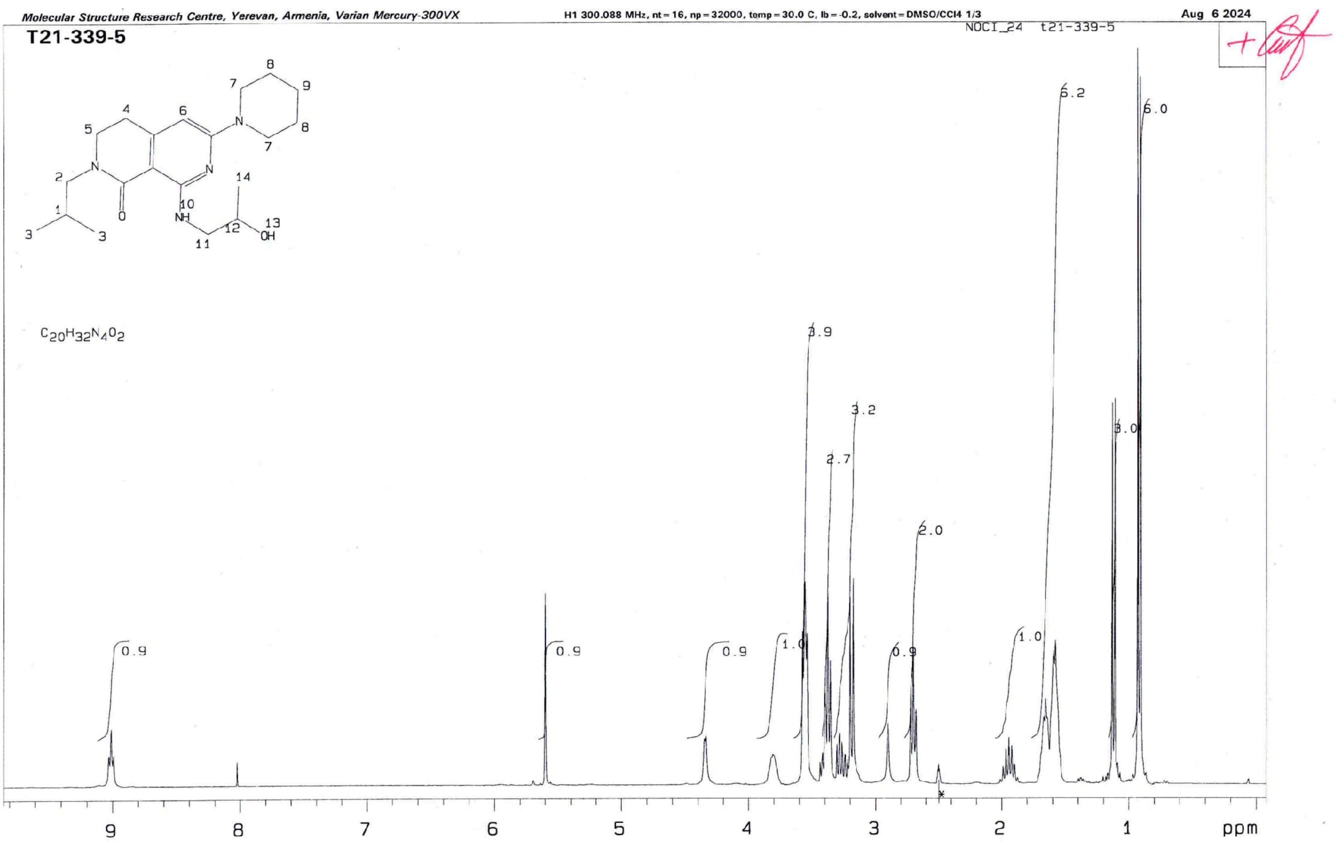

Figure S34. ^{1}H NMR spectrum of compound **4k**

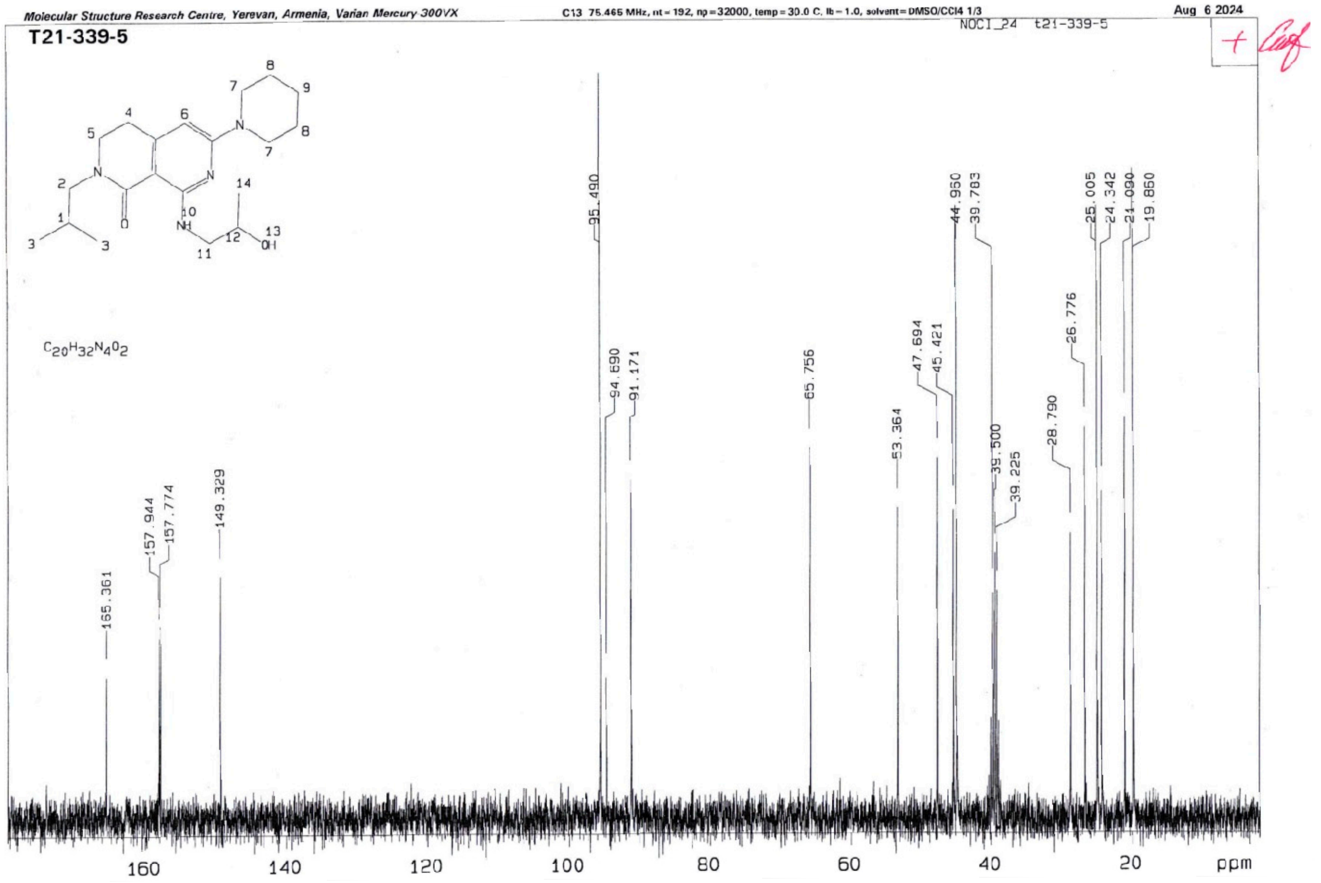

Figure S34.1. ^{13}C NMR spectrum of compound **4k**

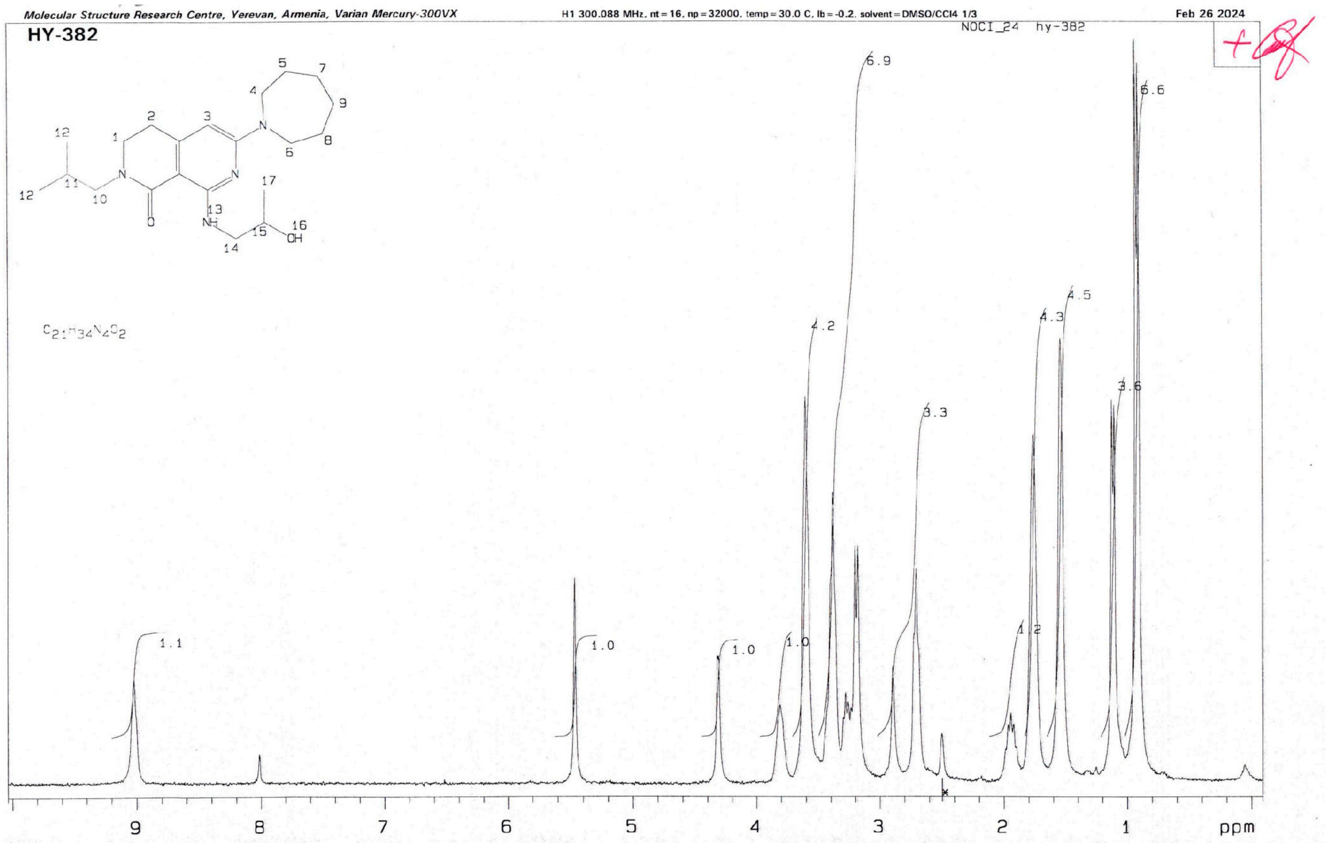

Figure S35. ^{1}H NMR spectrum of compound **4l**

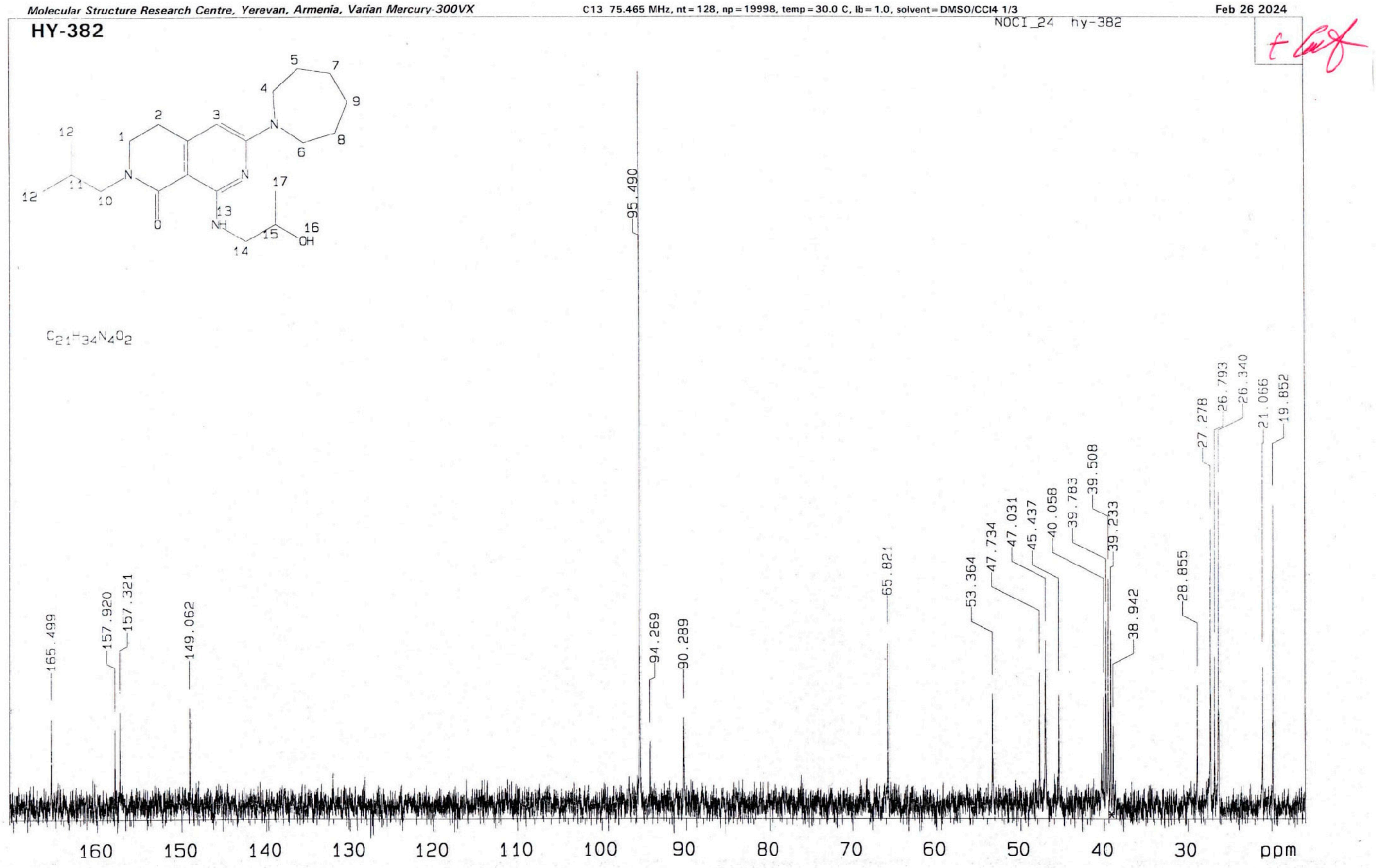

Figure S35.1. ^{13}C NMR spectrum of compound **4l**

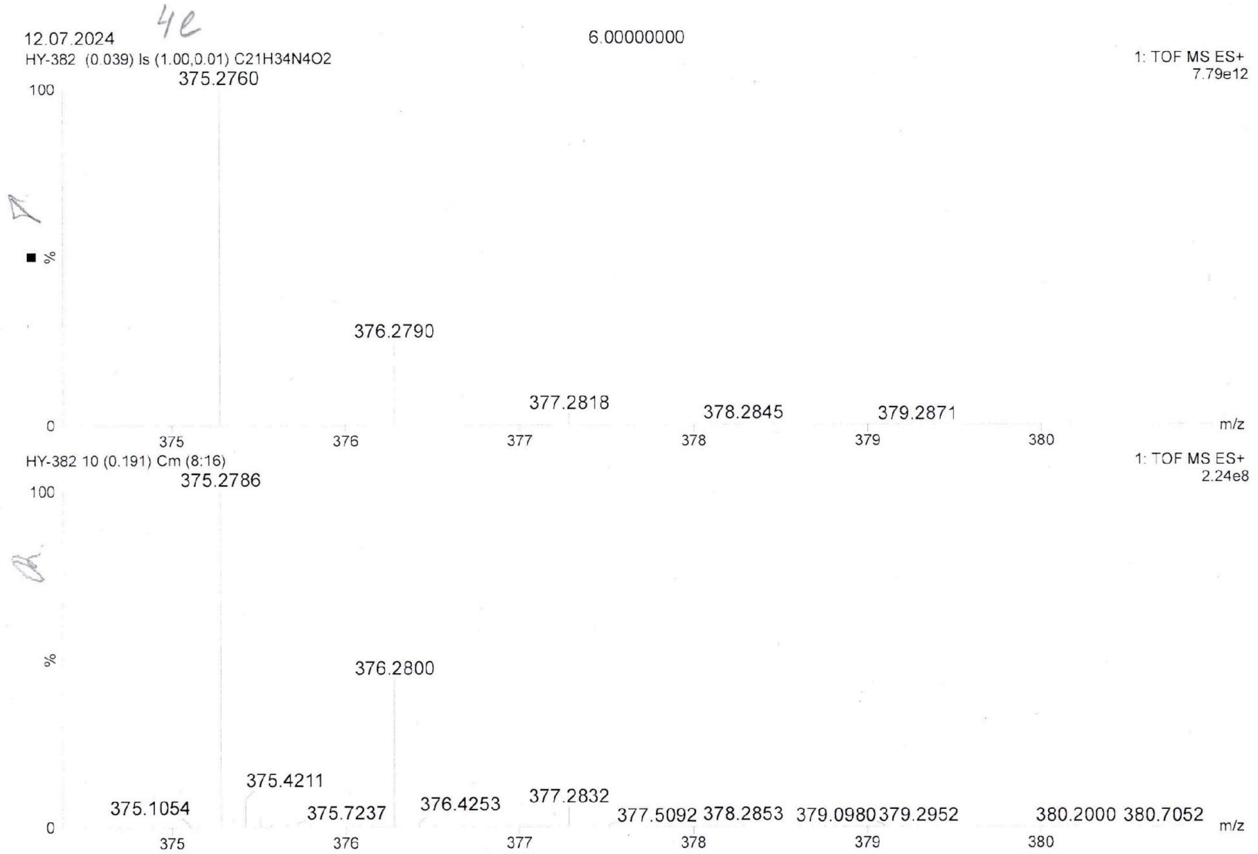

Figure S35.2. MS spectrum of compound **4l**

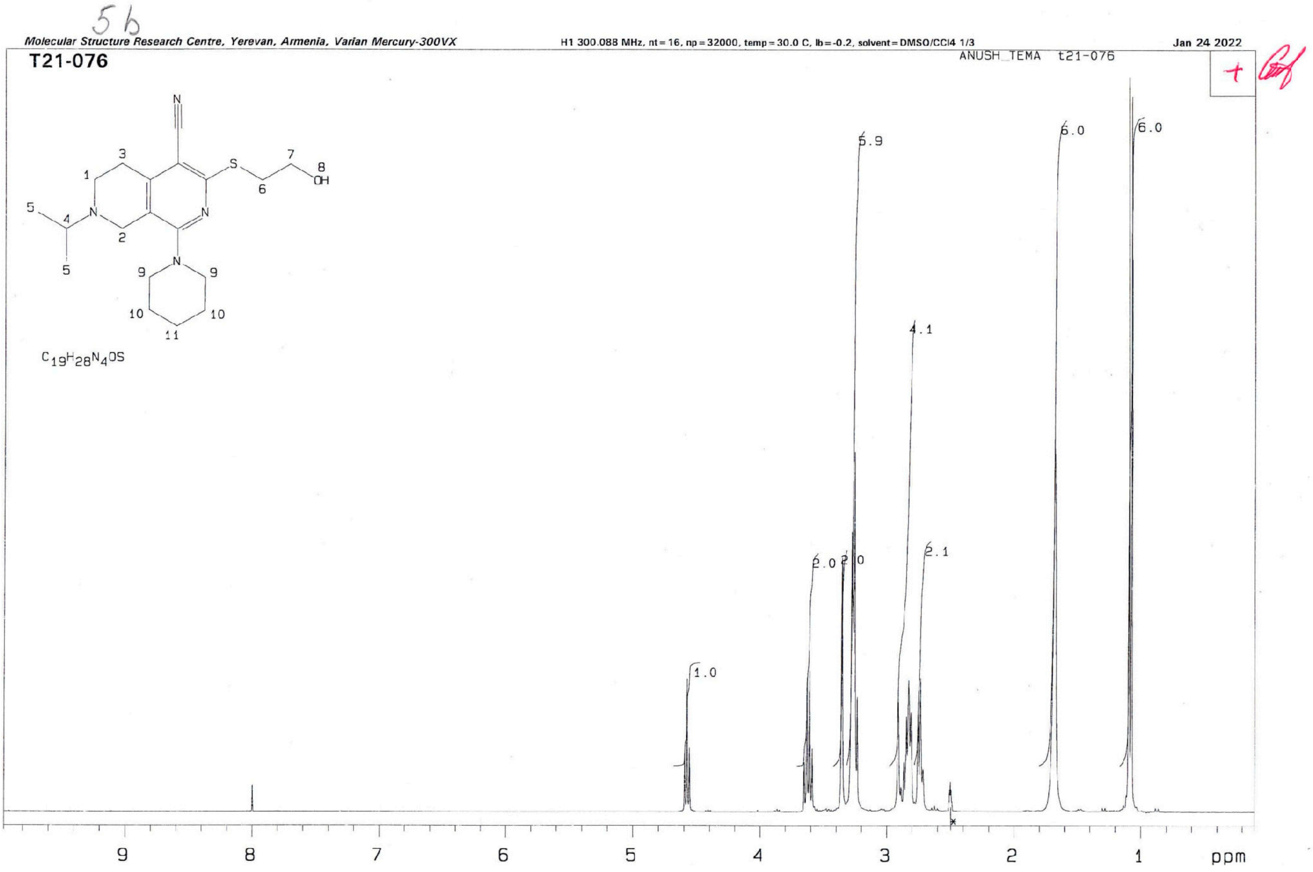

Figure S36. ^{1}H NMR spectrum of compound **5b**

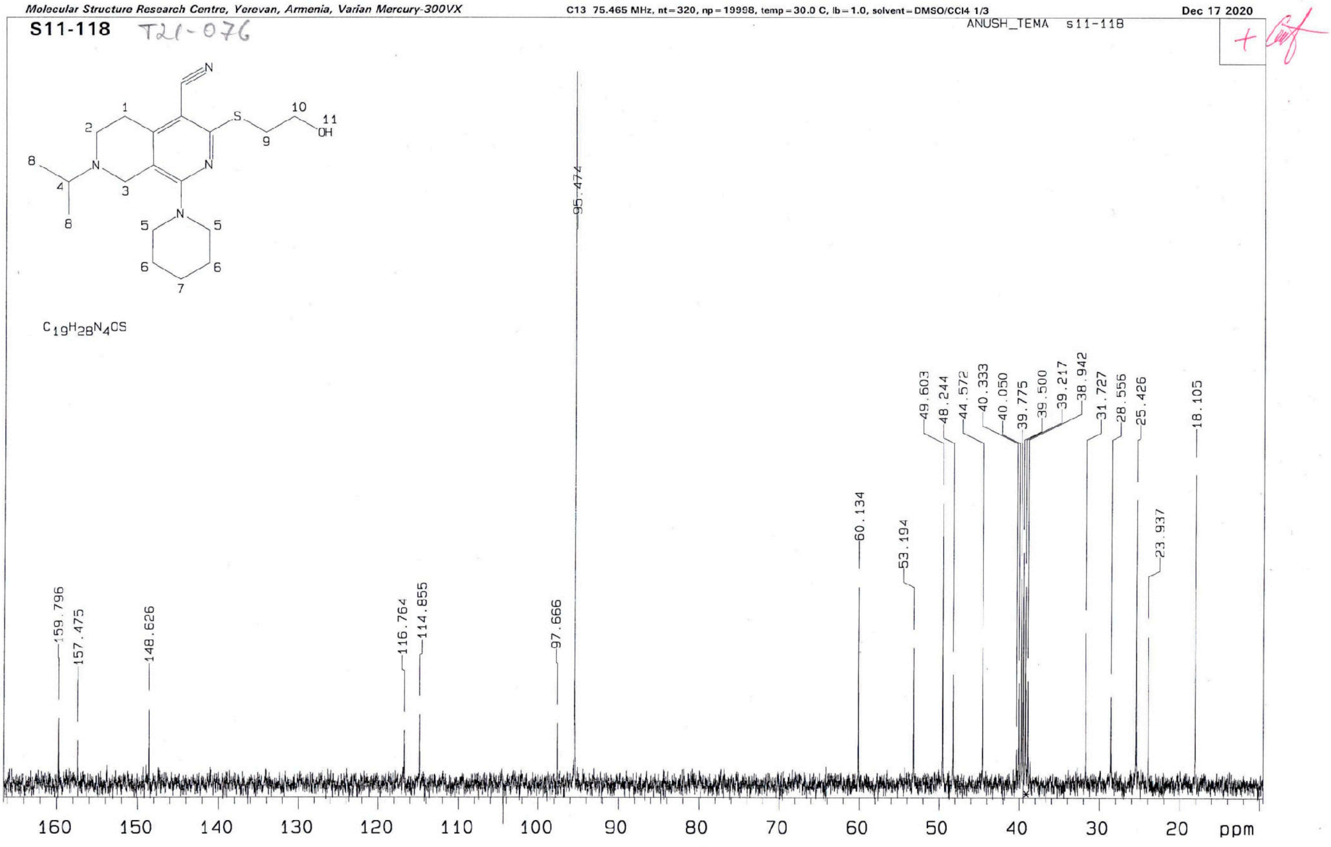

Figure S36.1. ^{13}C NMR spectrum of compound **5b**

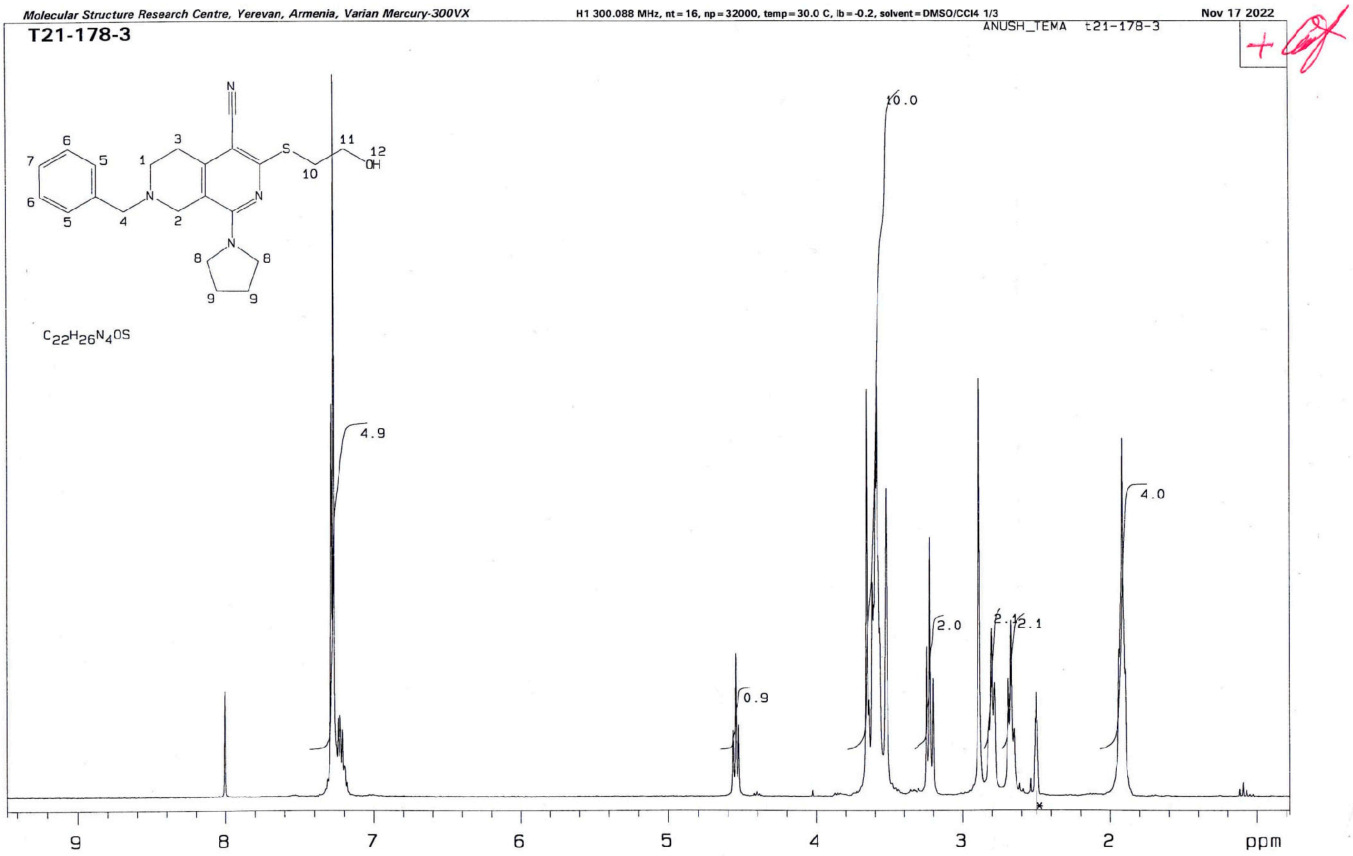

Figure S37. ^{1}H NMR spectrum of compound **5d**

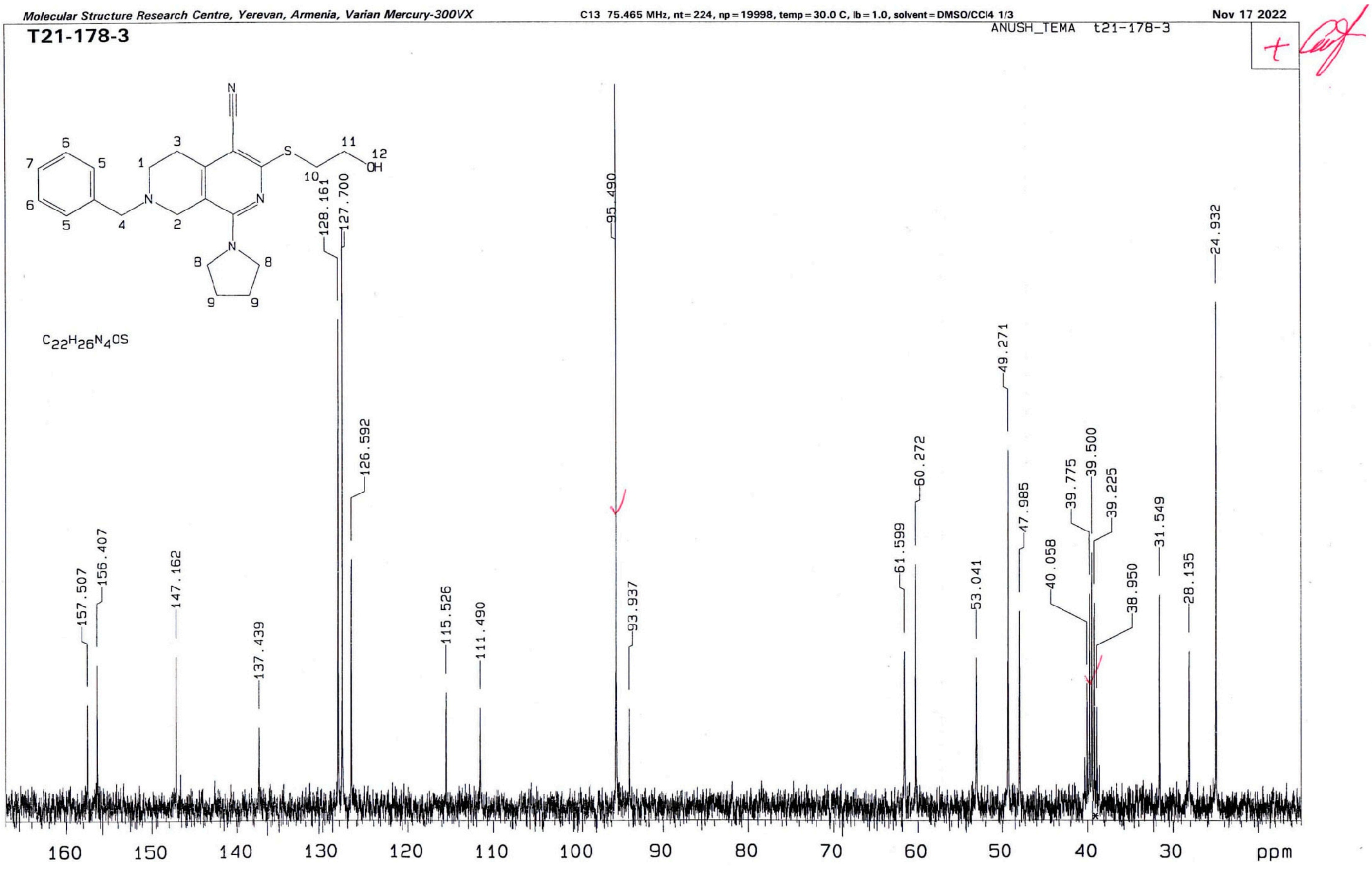

Figure S37.1. ^{13}C NMR spectrum of compound **5d**

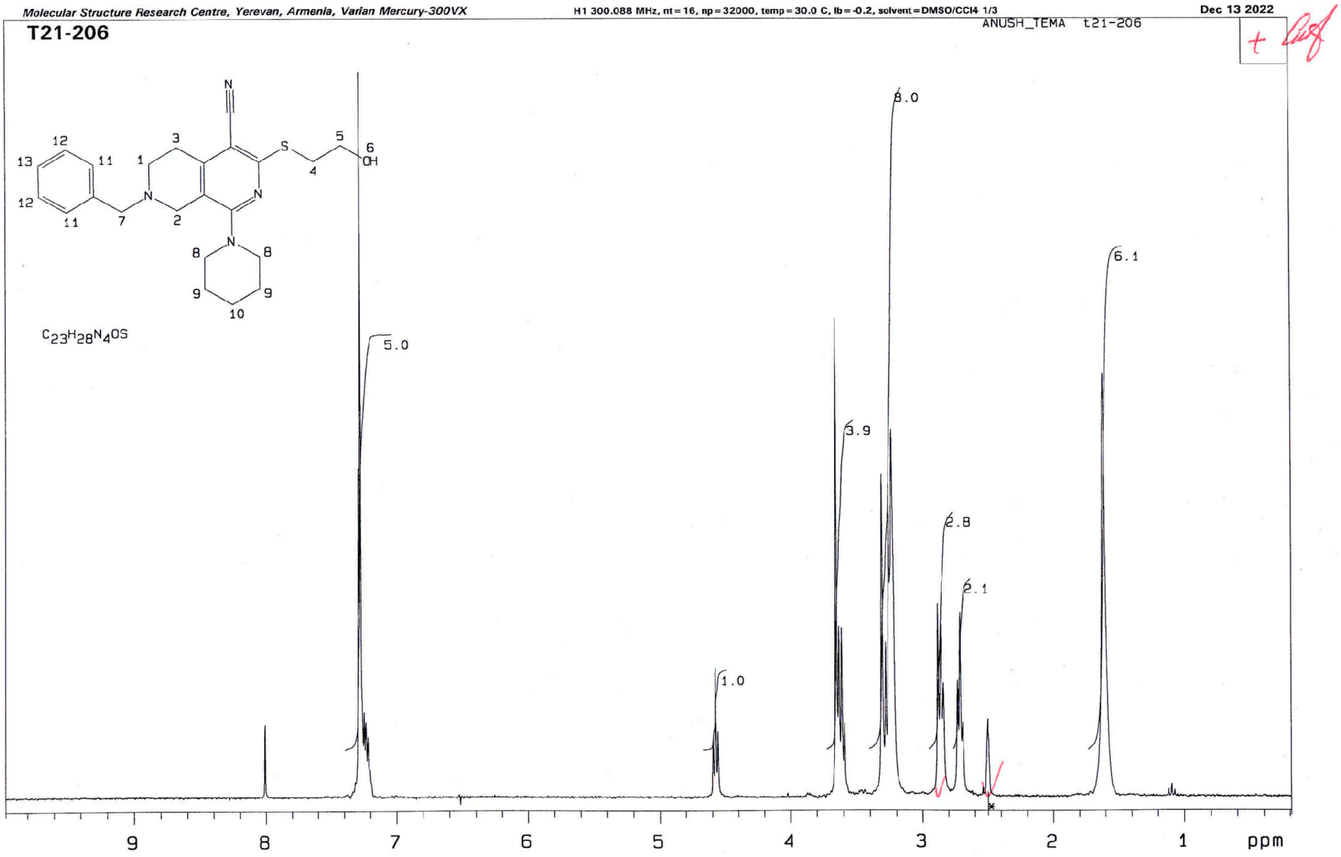

Figure S38. ^{1}H NMR spectrum of compound **5e**

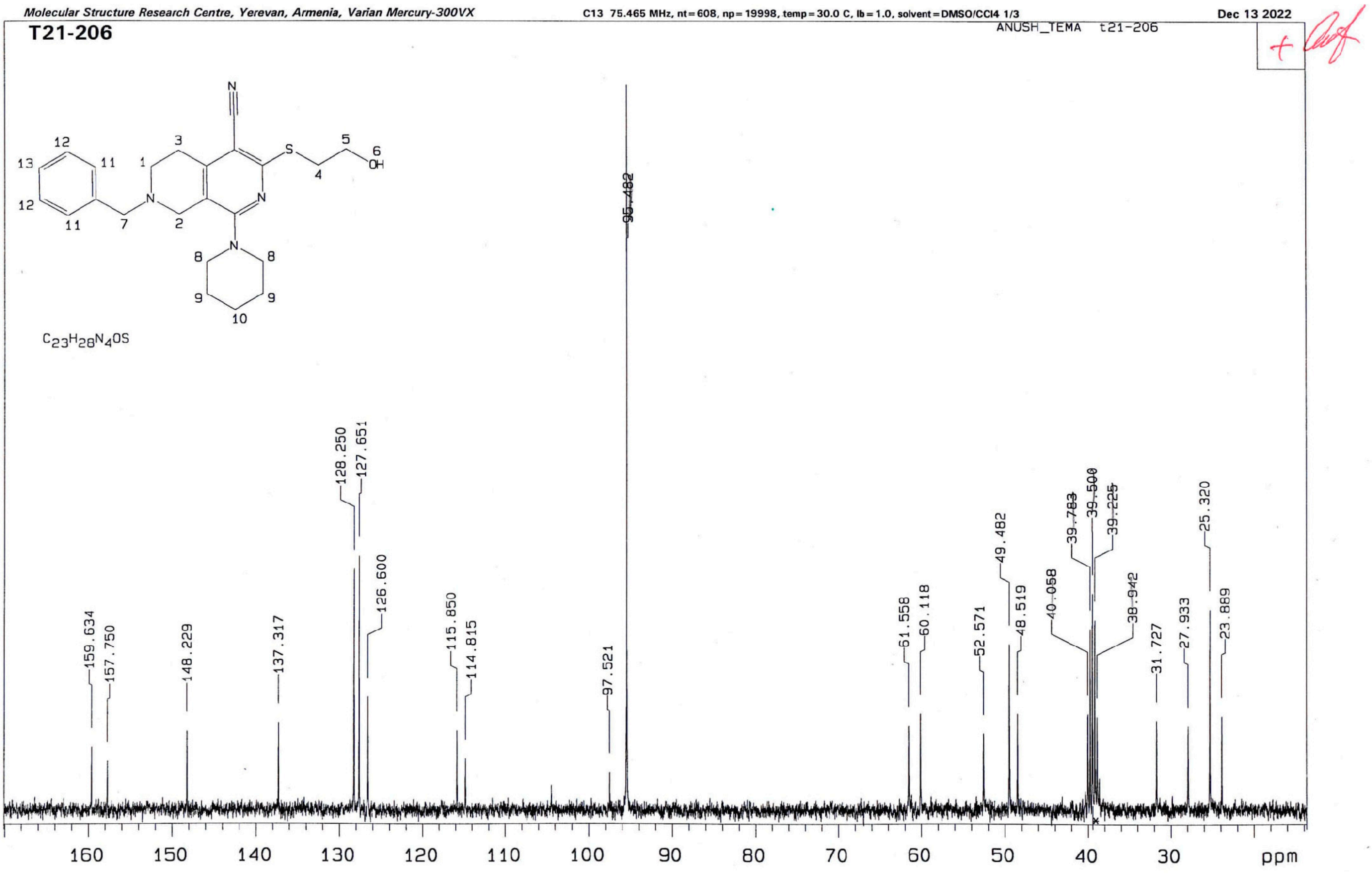

Figure S38.1. ^{13}C NMR spectrum of compound **5e**

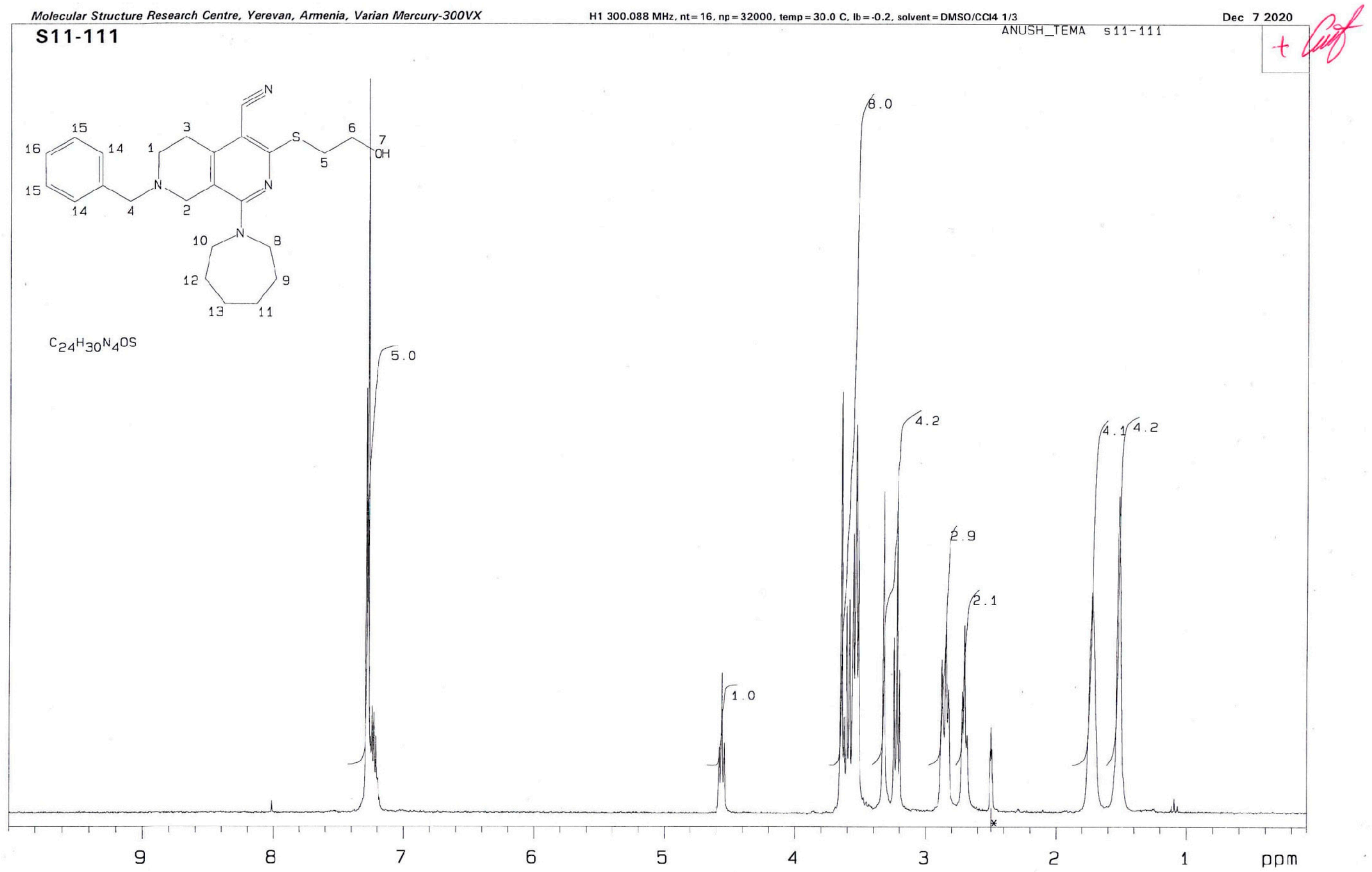

Figure S39. ^{1}H NMR spectrum of compound **5f**

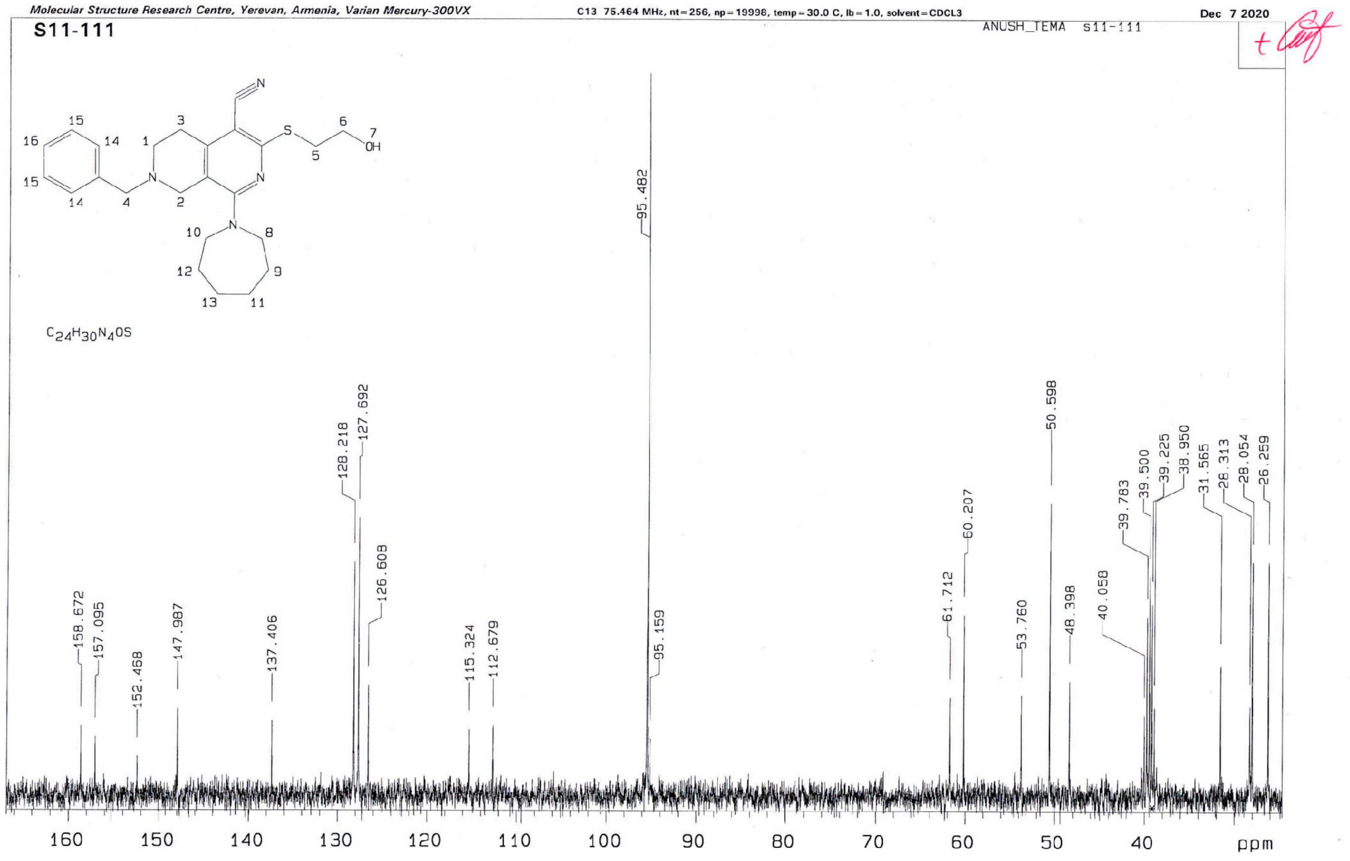

Figure S39.1. ^{13}C NMR spectrum of compound **5f**

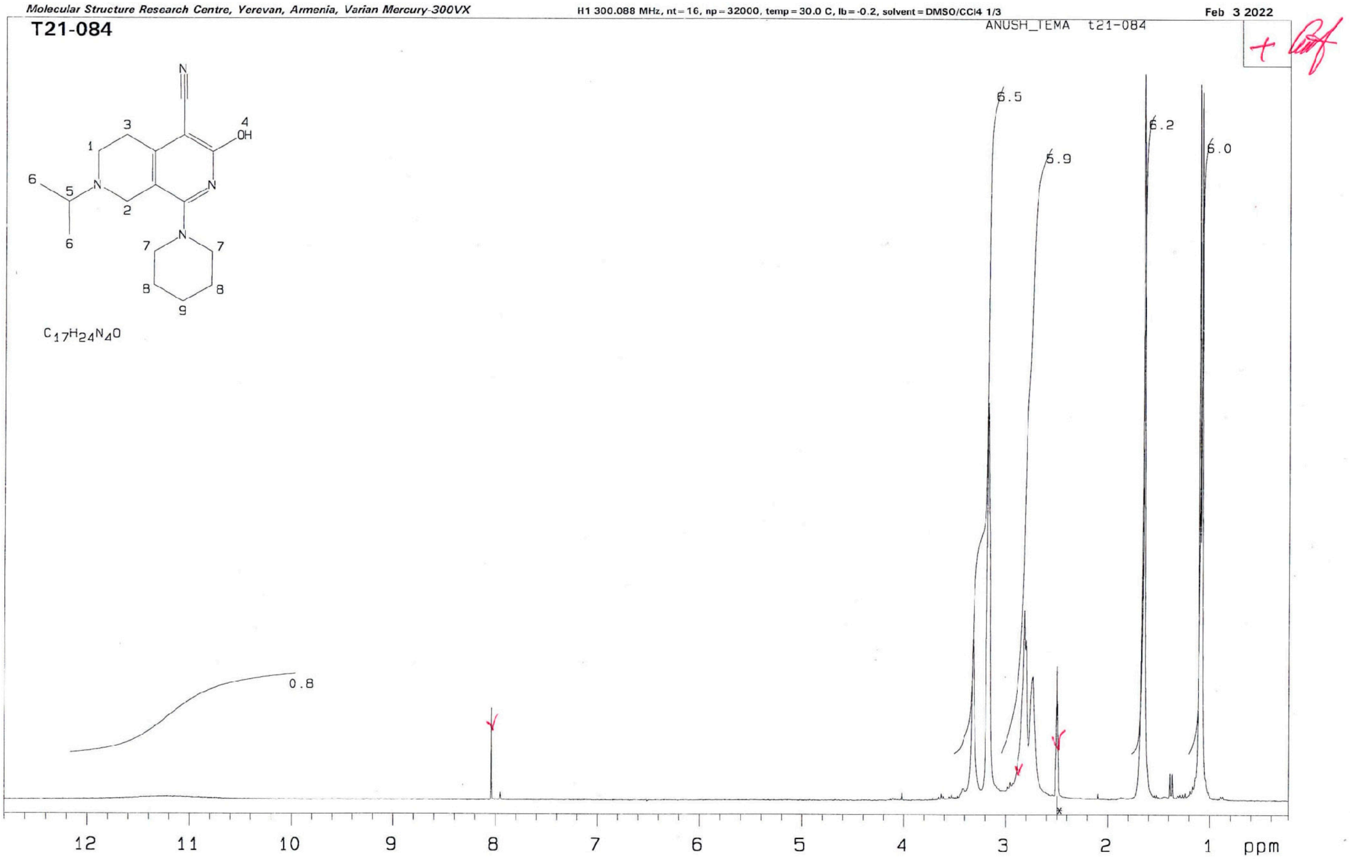

Figure S40. ^{1}H NMR spectrum of compound **6b**

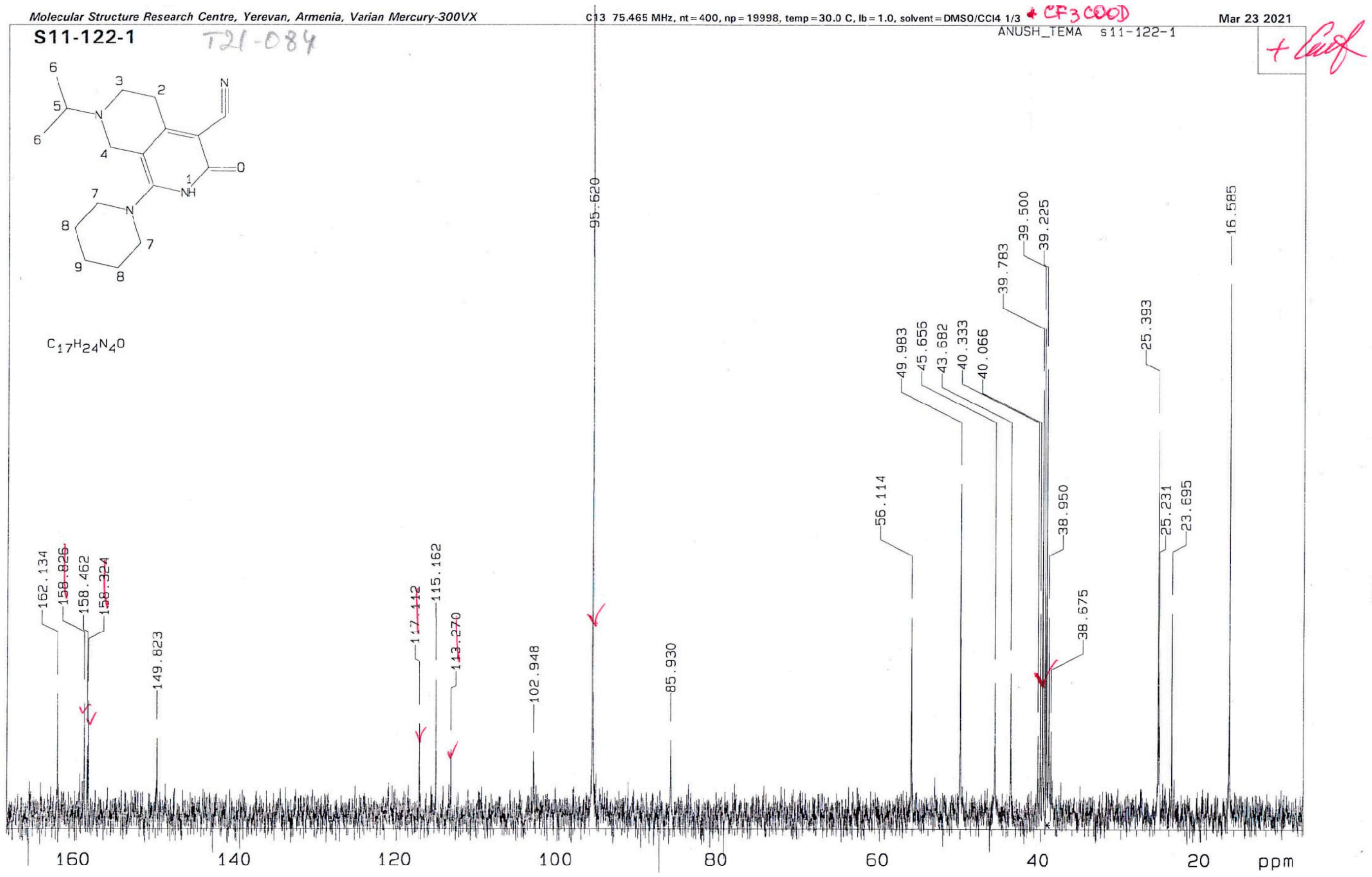

Figure S40.1. ^{13}C NMR spectrum of compound **6b**

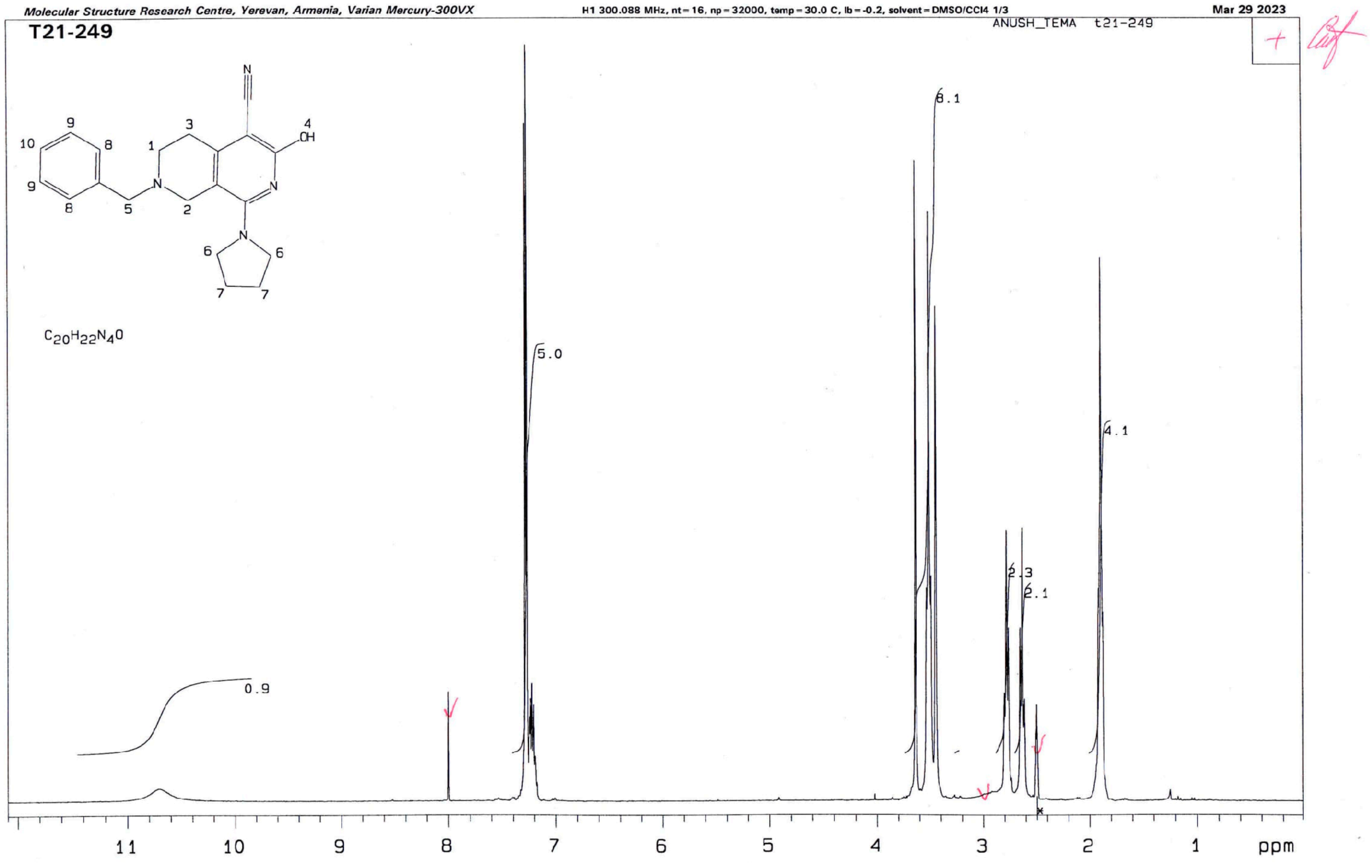

Figure S41. ^{1}H NMR spectrum of compound **6d**

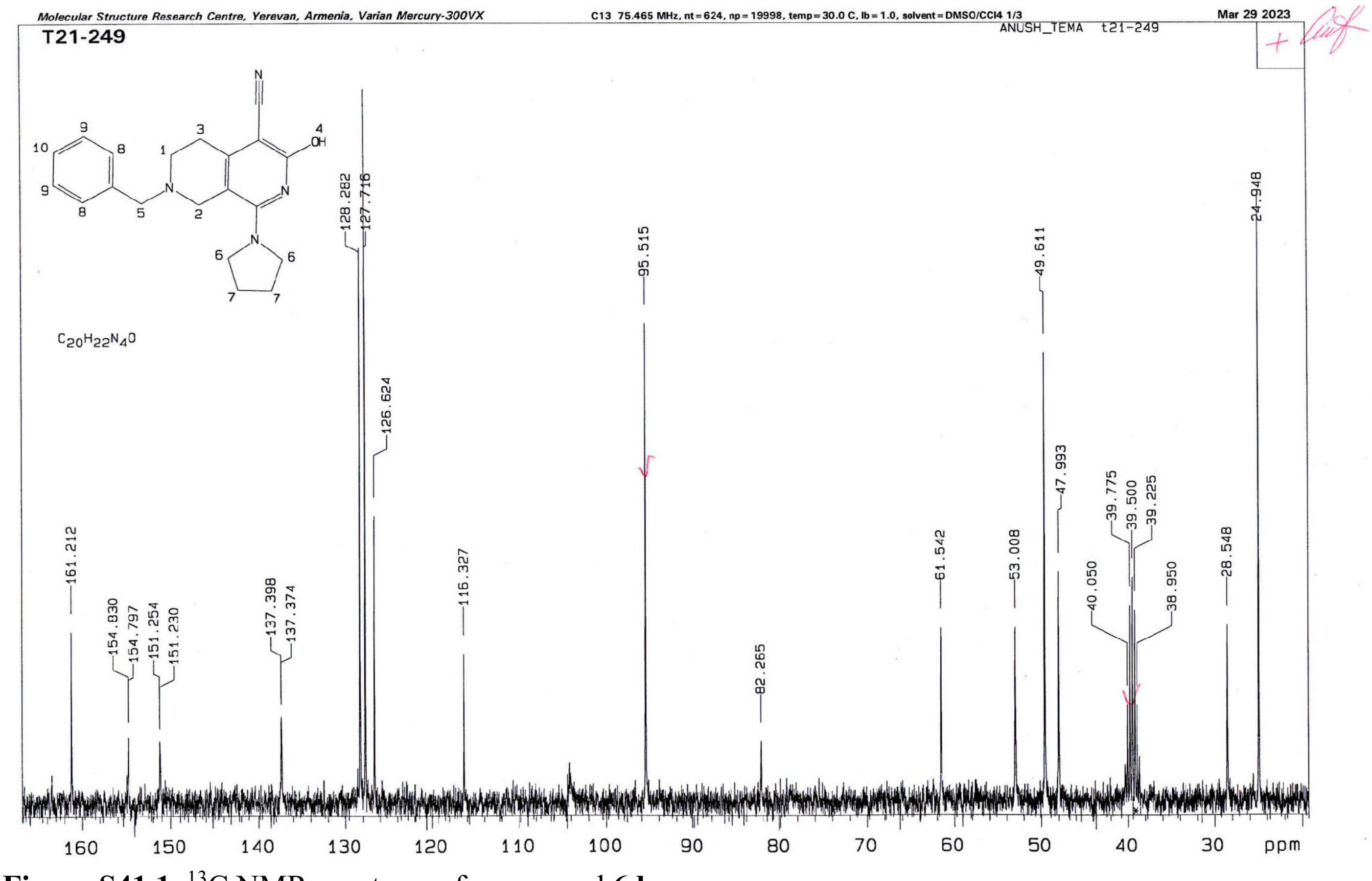

Figure S41.1. ^{13}C NMR spectrum of compound **6d**

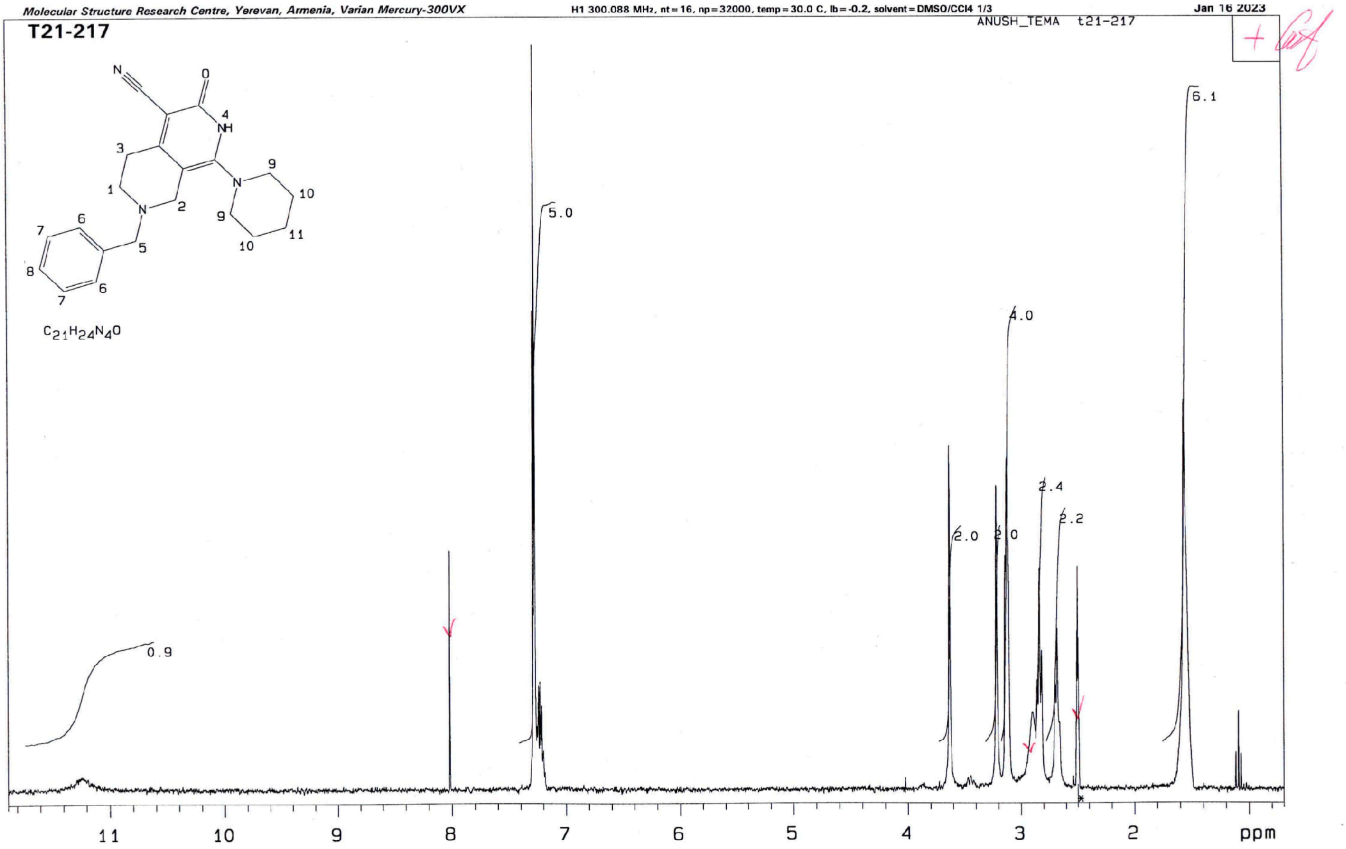

Figure S41.1. ^{13}C NMR spectrum of compound **6d**

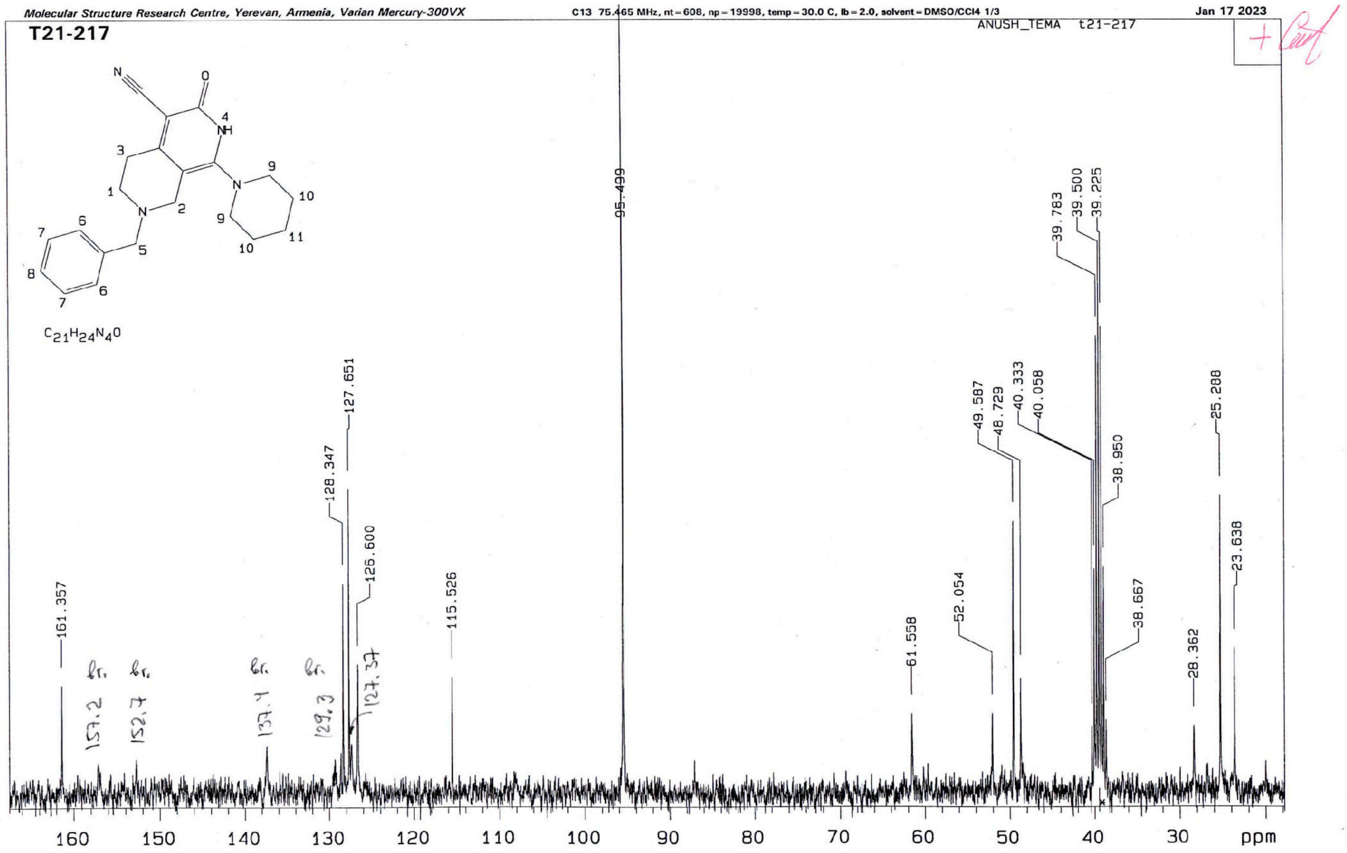

Figure S42.1. ^{13}C NMR spectrum of compound **6e**

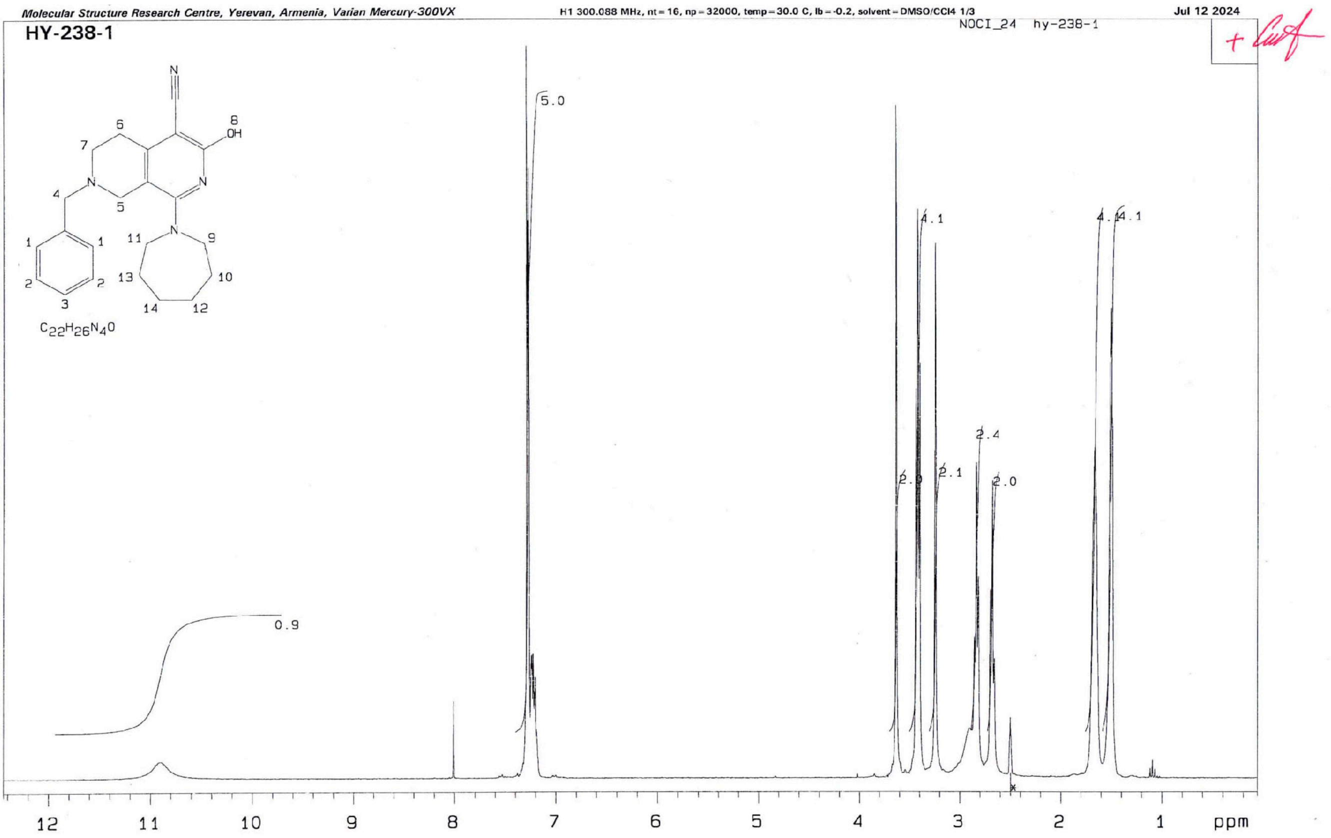

Figure S43. ^{1}H NMR spectrum of compound **6f**

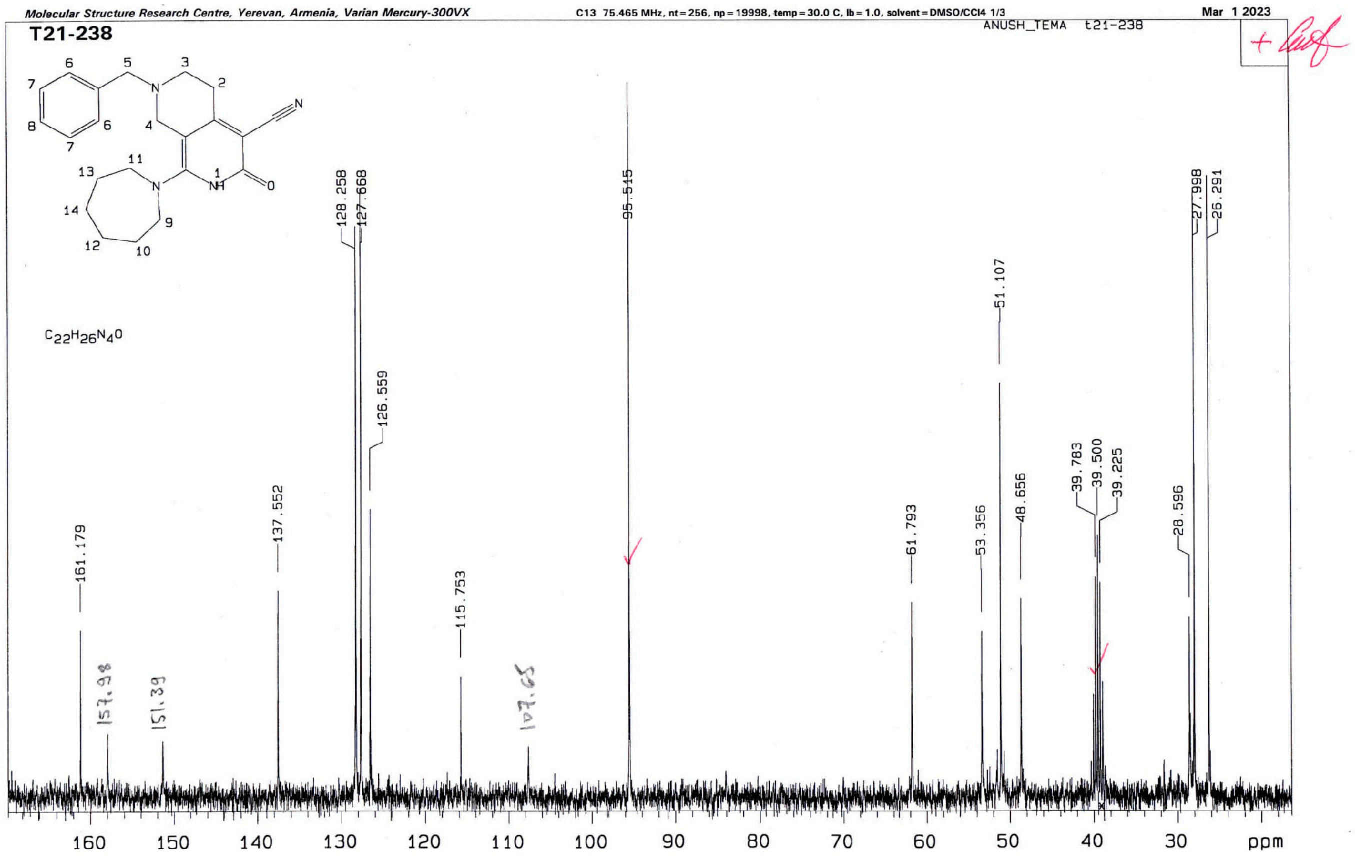

Figure S43.1. ^{13}C NMR spectrum of compound **6f**

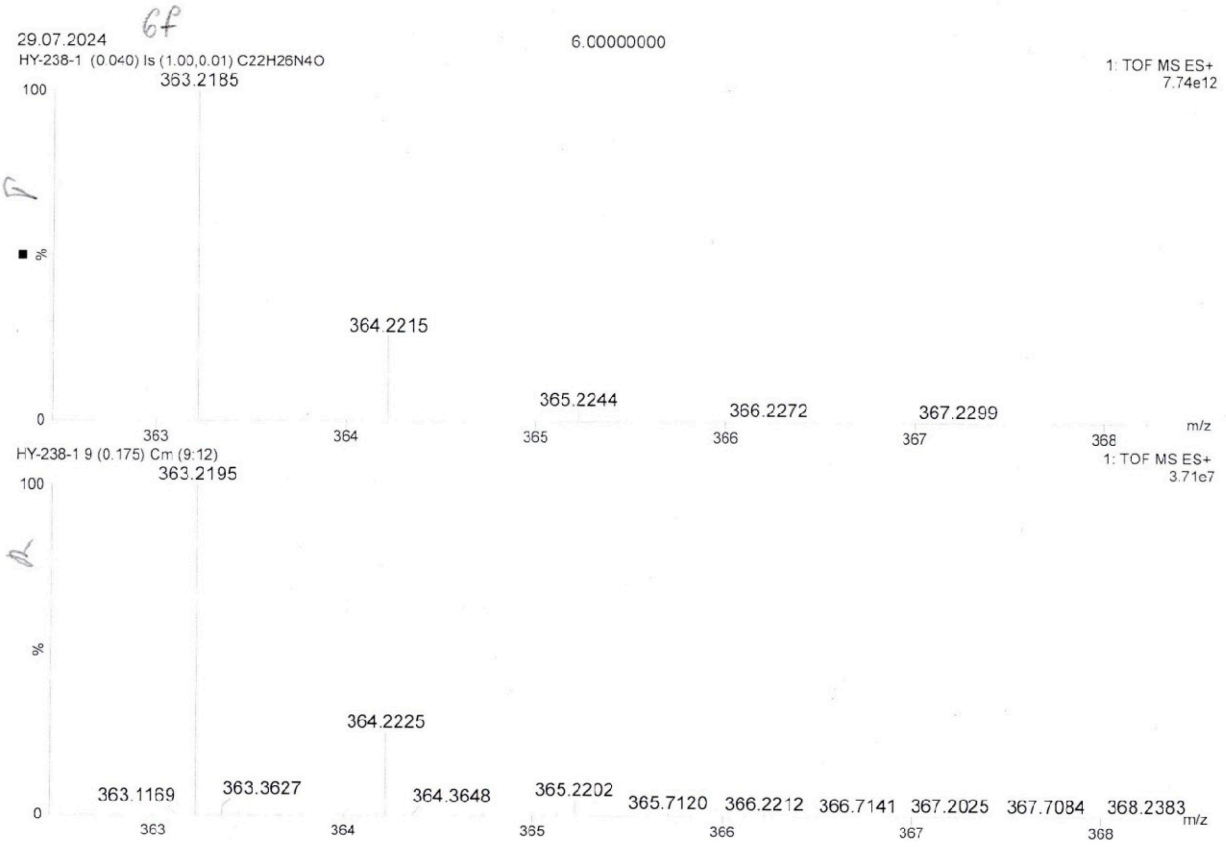

Figure S43.2. MS spectrum of compound **6f**

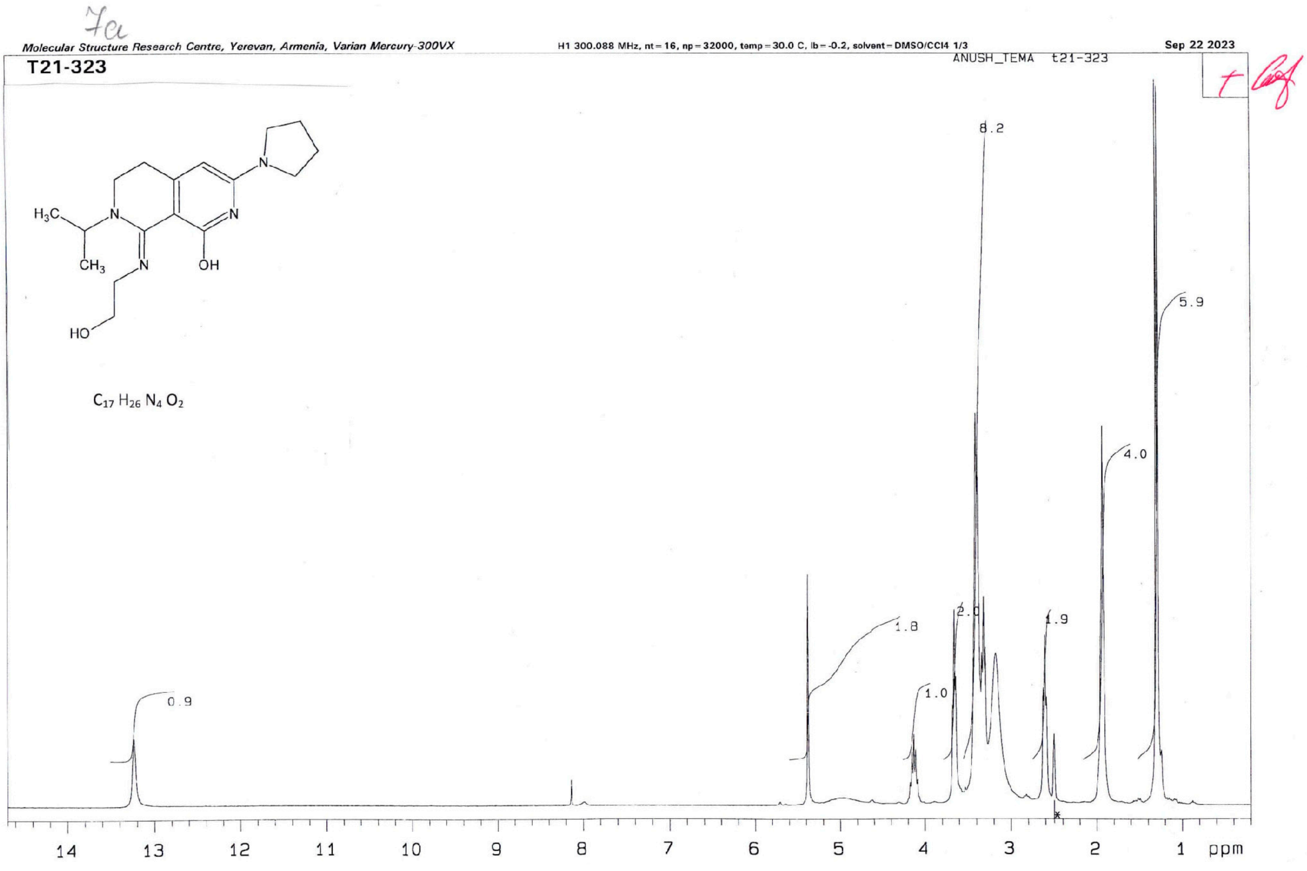

Figure S44. ^{1}H NMR spectrum of compound **7a**

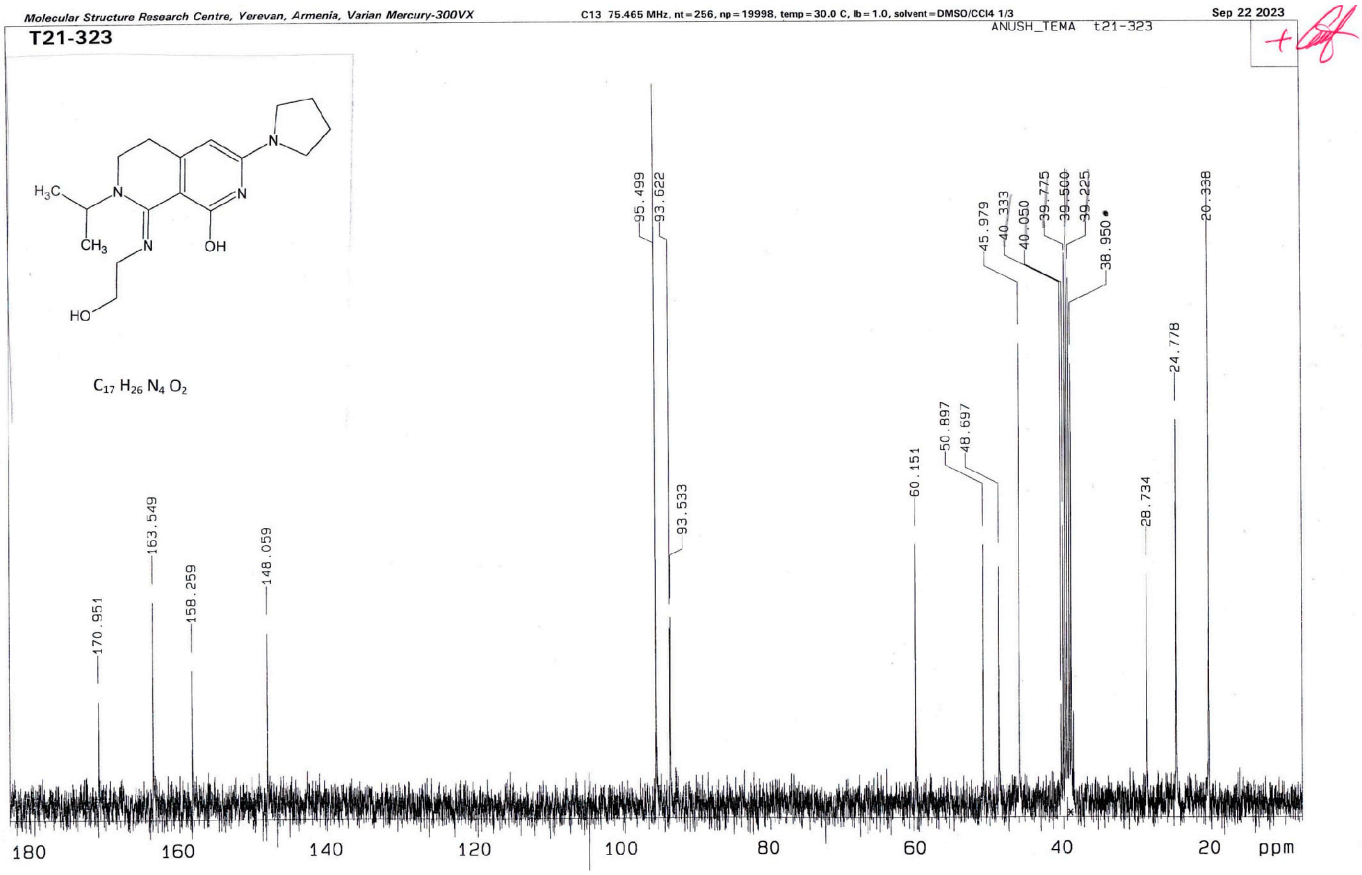

Figure S44.1. ^{13}C NMR spectrum of compound **7a**

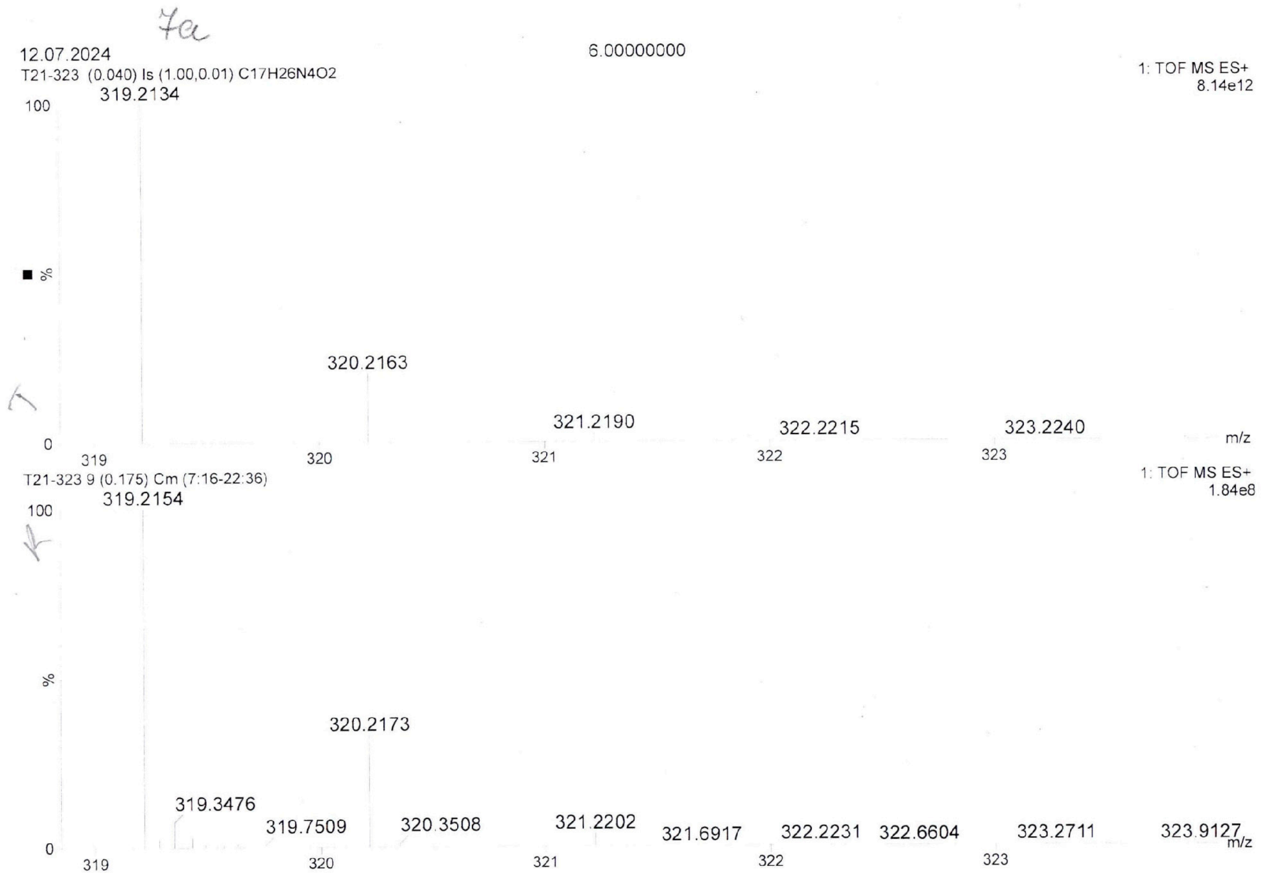

Figure S44.2. MS spectrum of compound **7a**

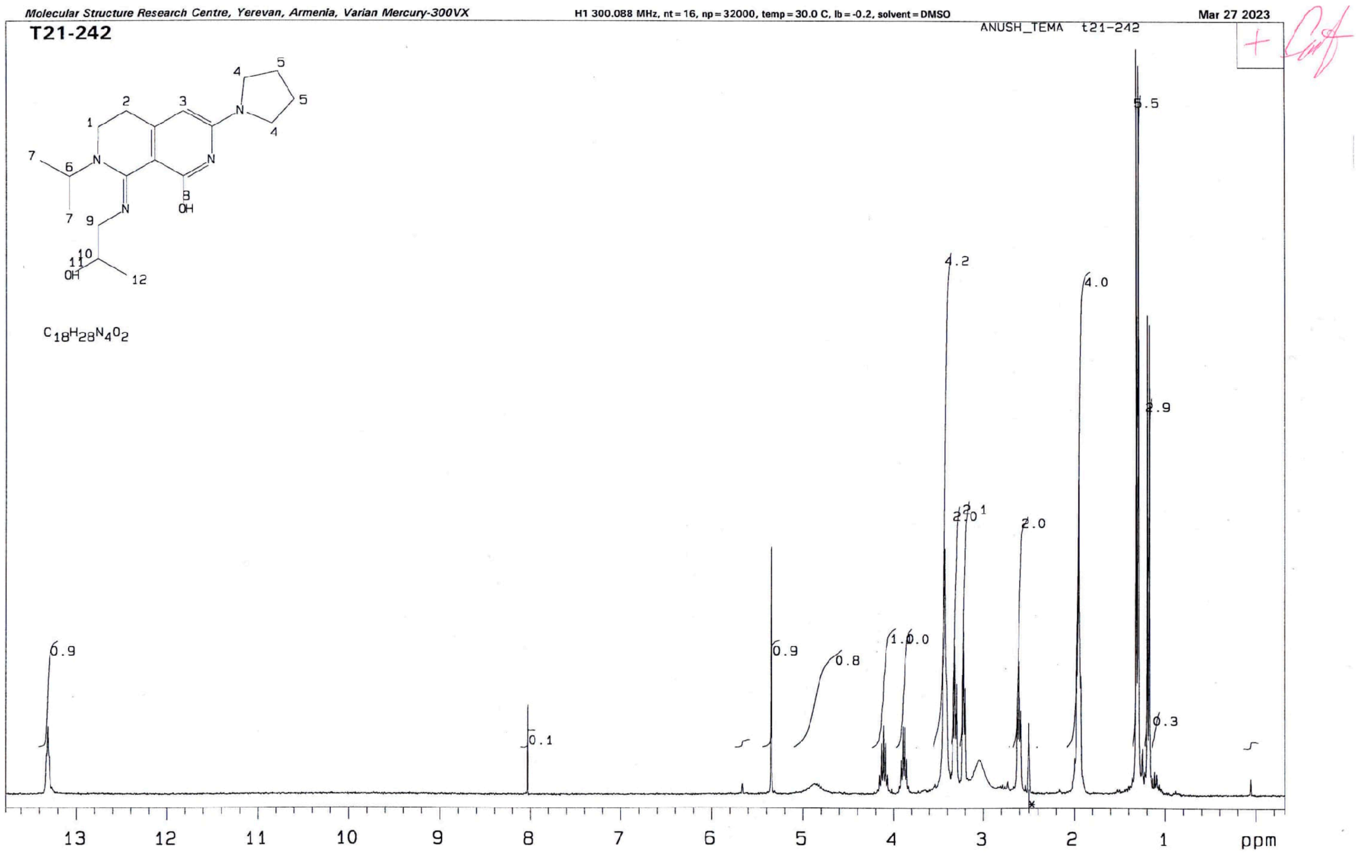

Figure S45. ^{1}H NMR spectrum of compound **7b**

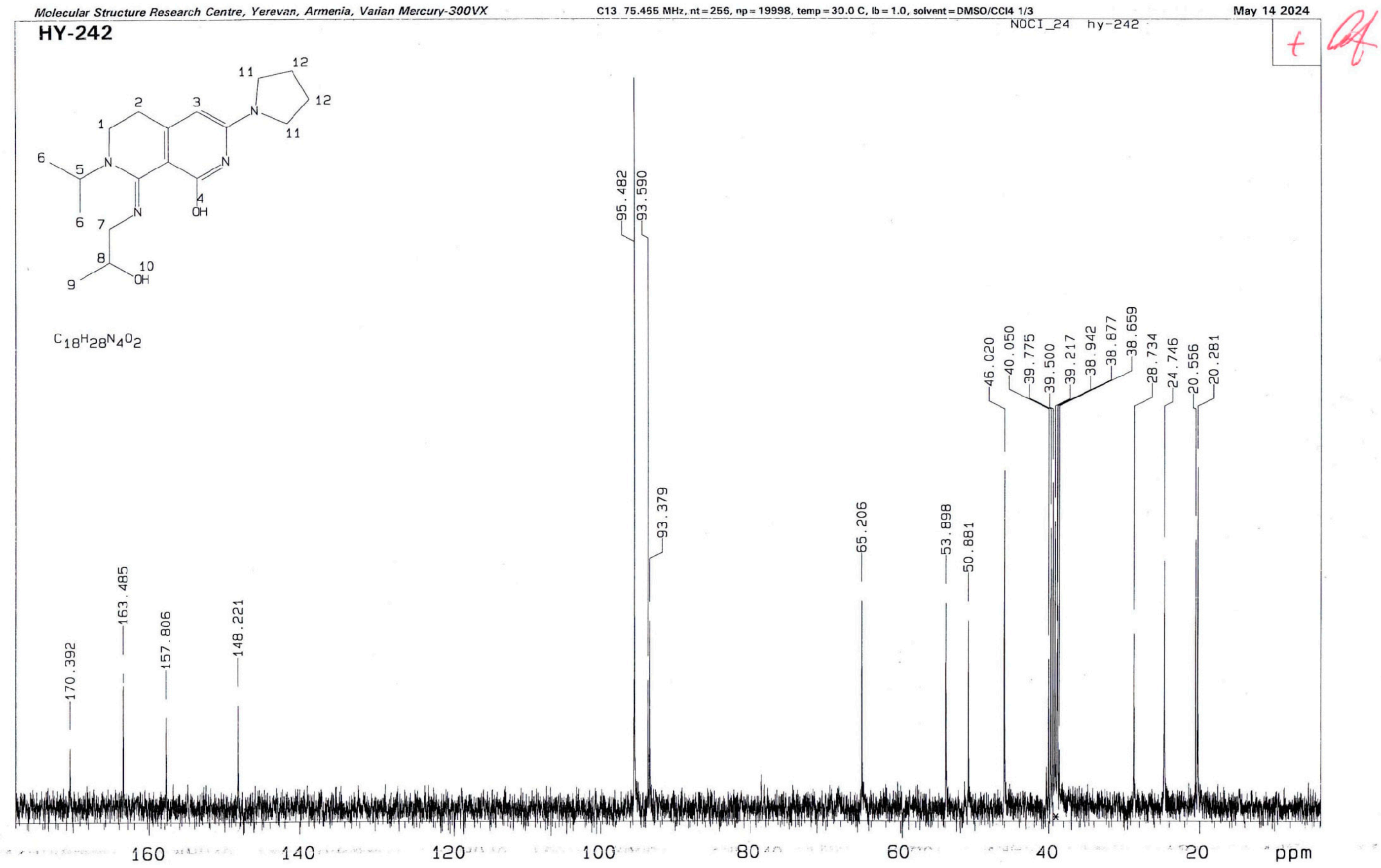

Figure S45.1. ^{13}C NMR spectrum of compound **7b**

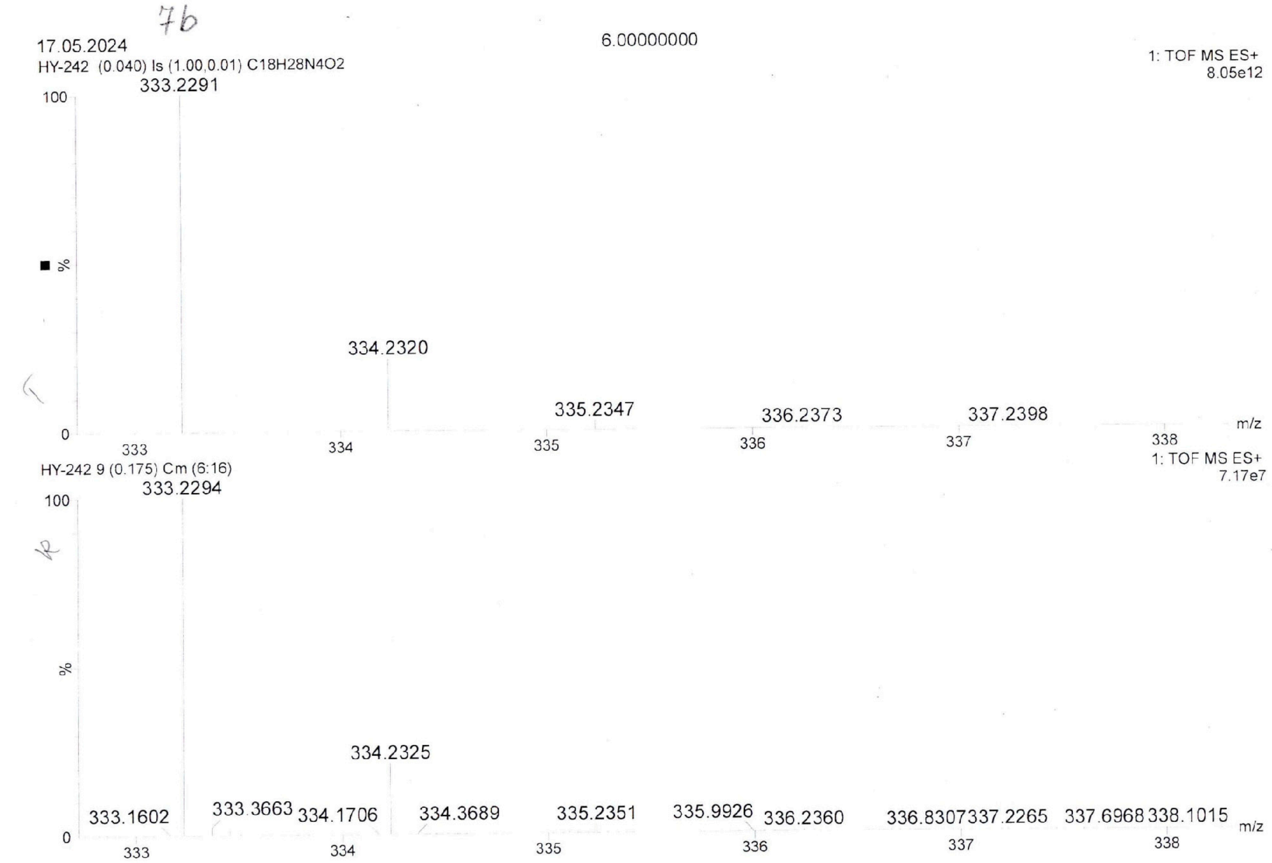

Figure S45.2. MS spectrum of compound **7b**

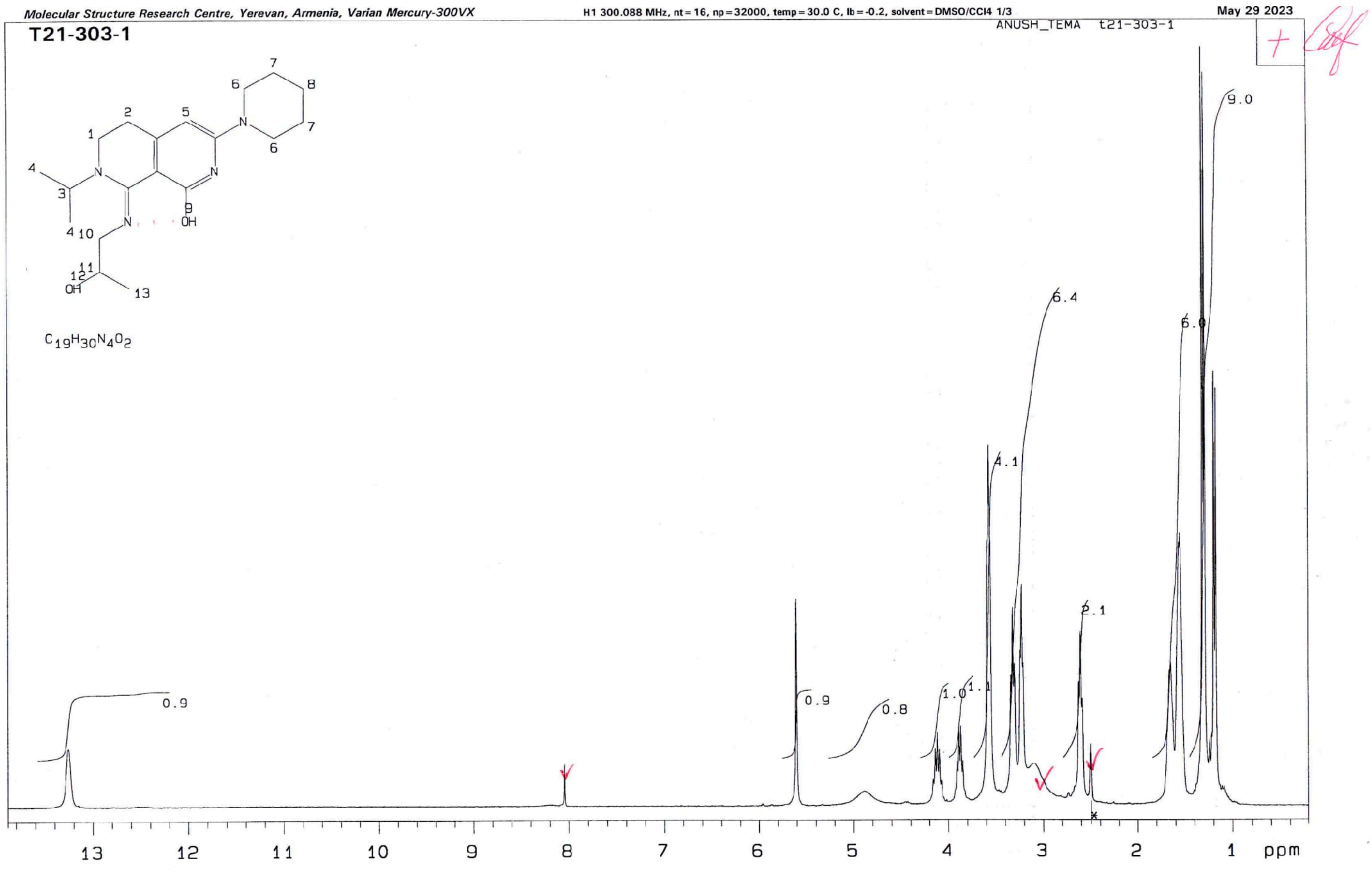

Figure S46. ^{1}H NMR spectrum of compound **7c**

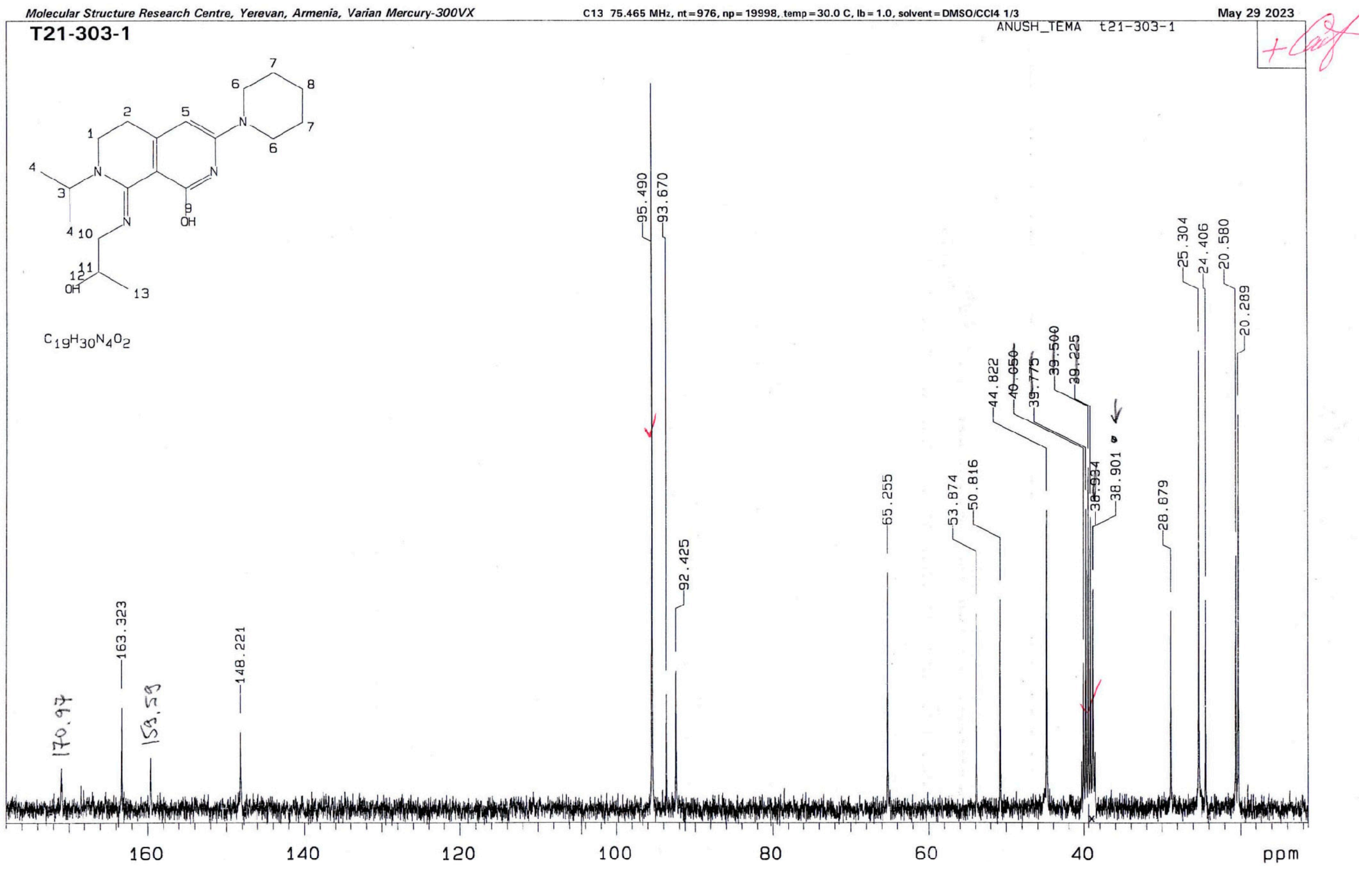

Figure S46.1. ^{13}C NMR spectrum of compound **7c**

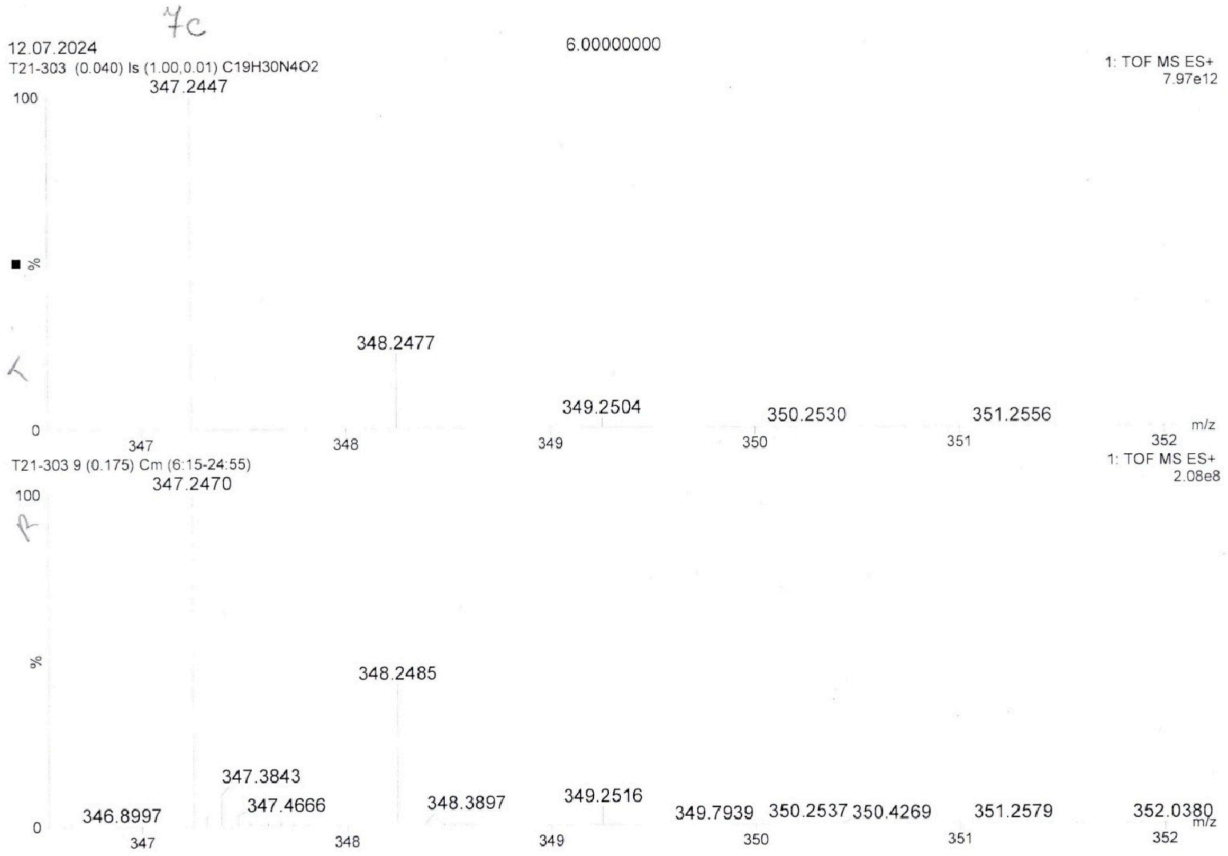

Figure S46.2. MS spectrum of compound **7c**

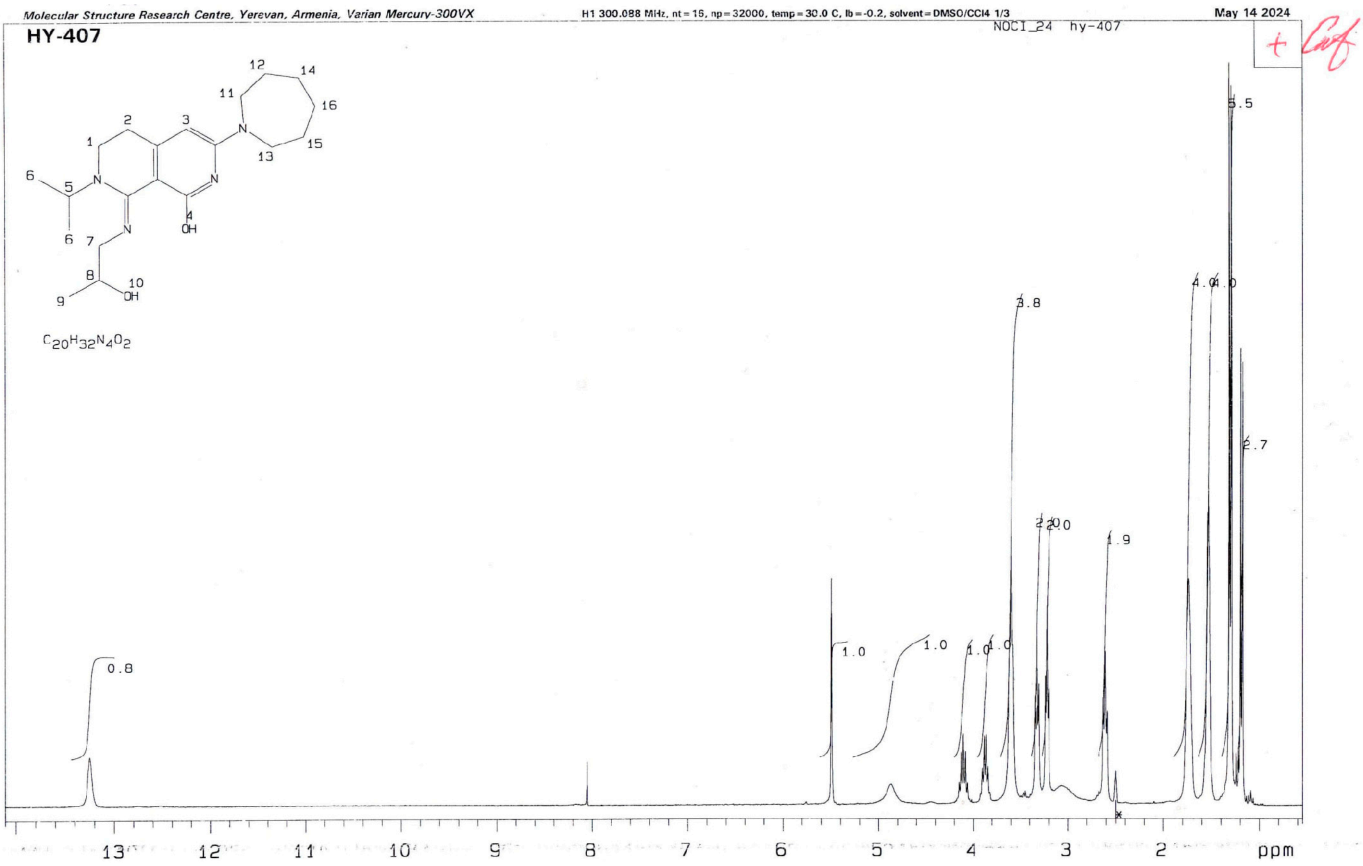

Figure S47. ^{1}H NMR spectrum of compound **7d**

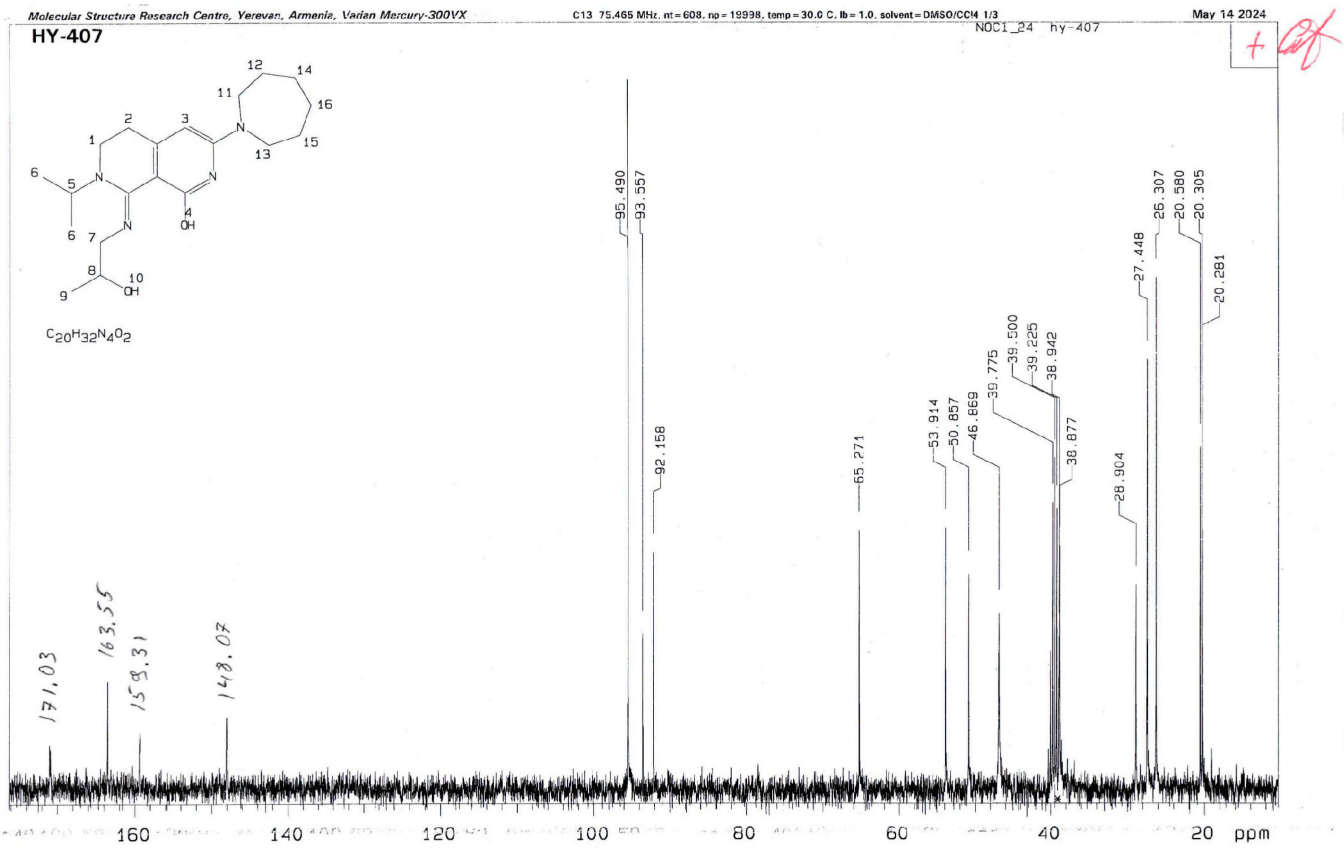

Figure S47.1. ^{13}C NMR spectrum of compound **7d**

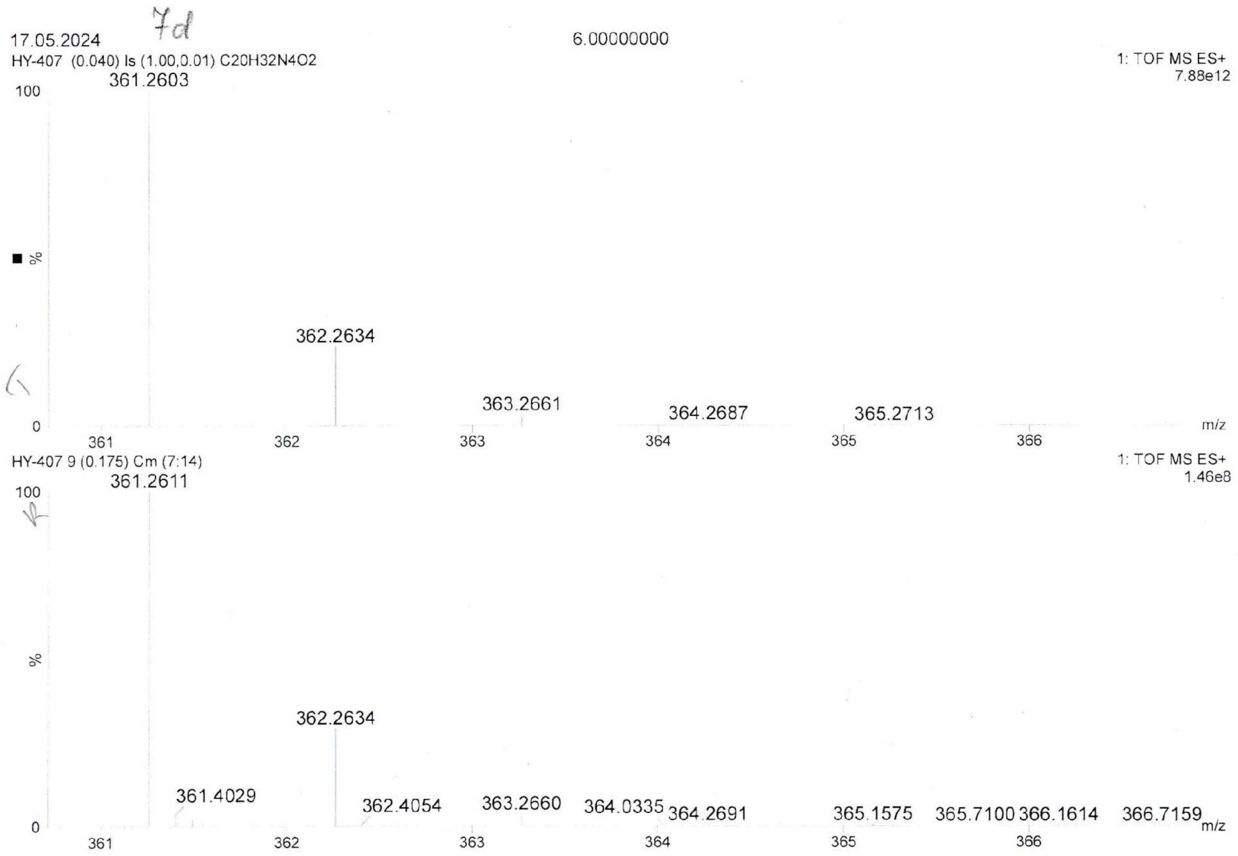

Figure S47.2. MS spectrum of compound **7d**

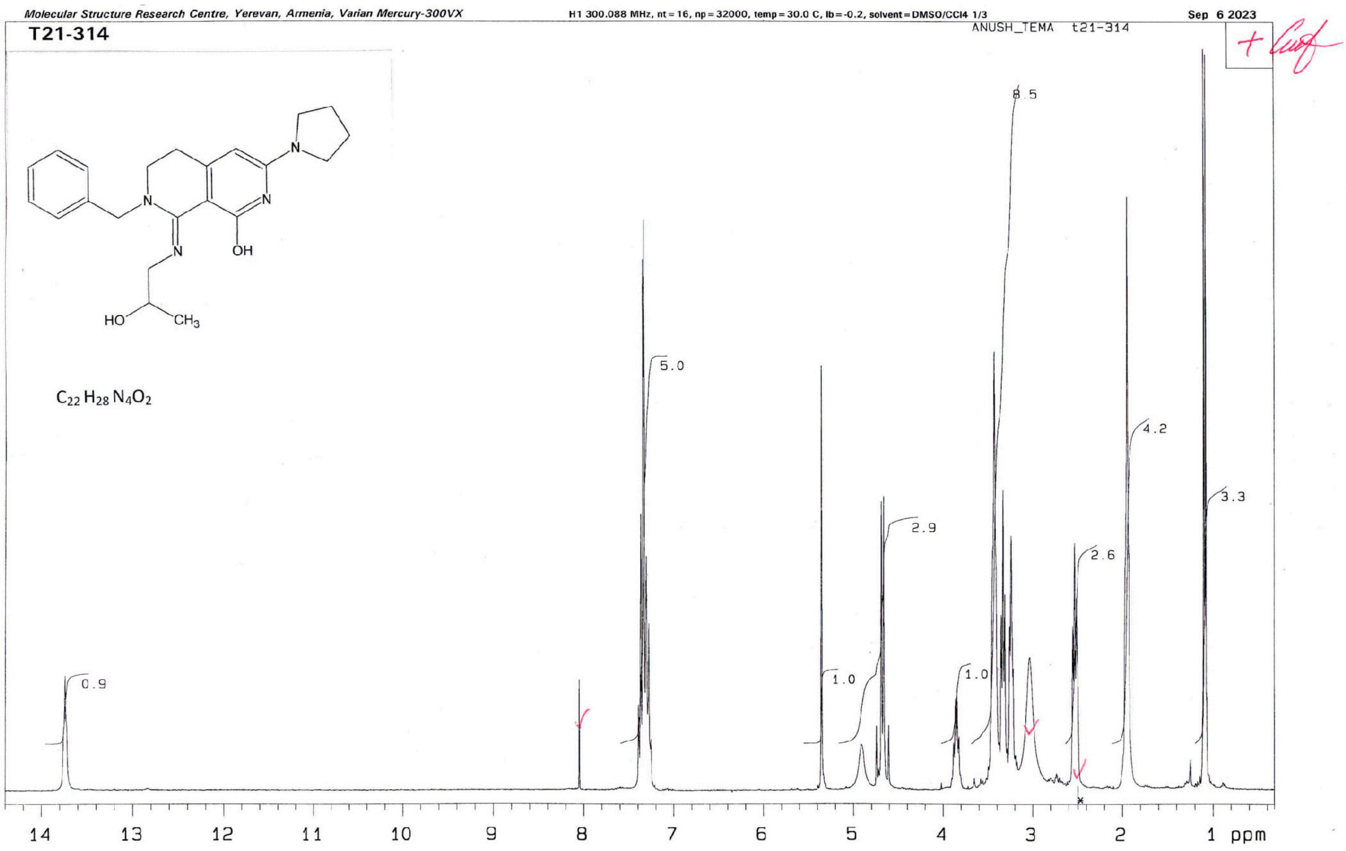

Figure S48. ^{1}H NMR spectrum of compound **7e**

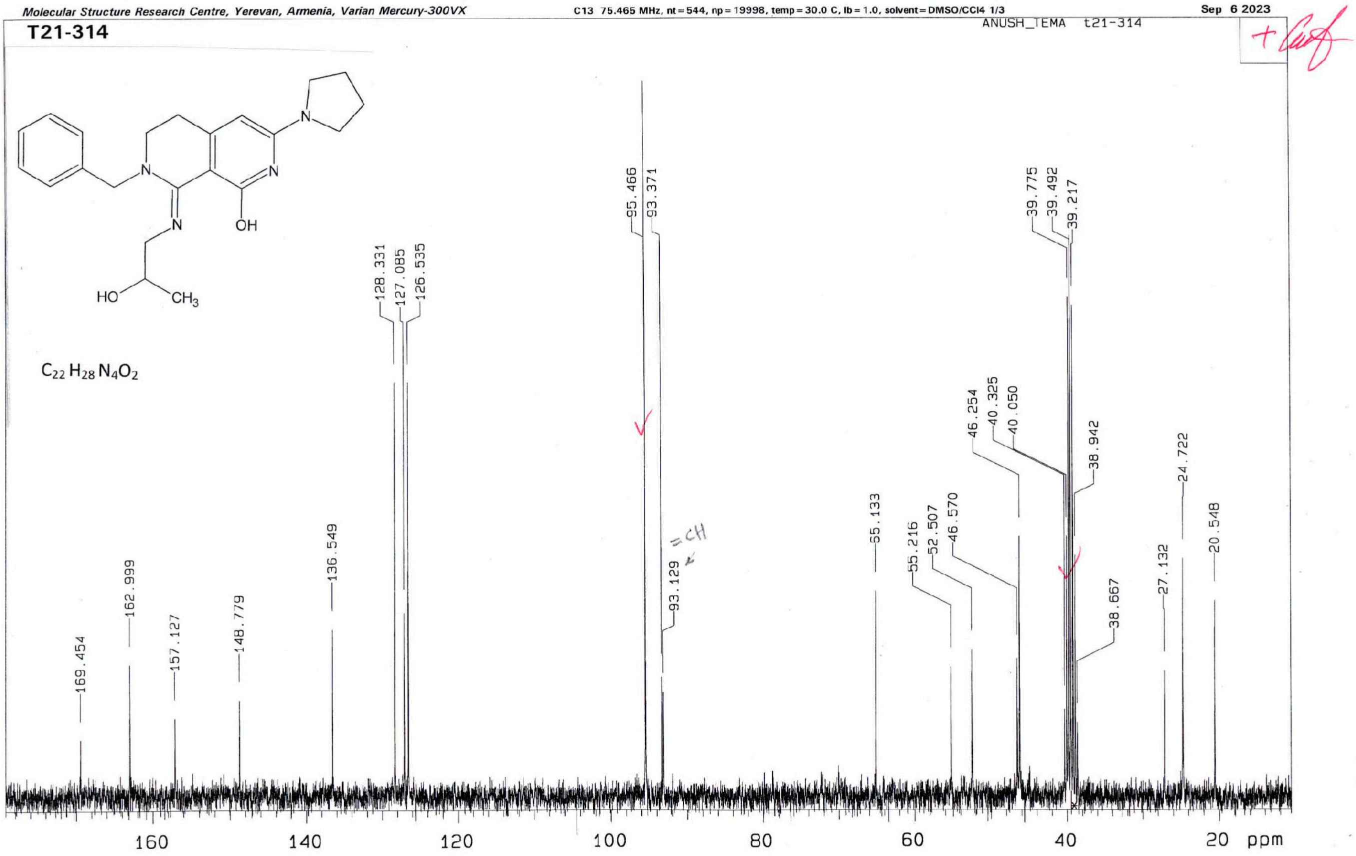

Figure S48.1. ^{13}C NMR spectrum of compound **7e**

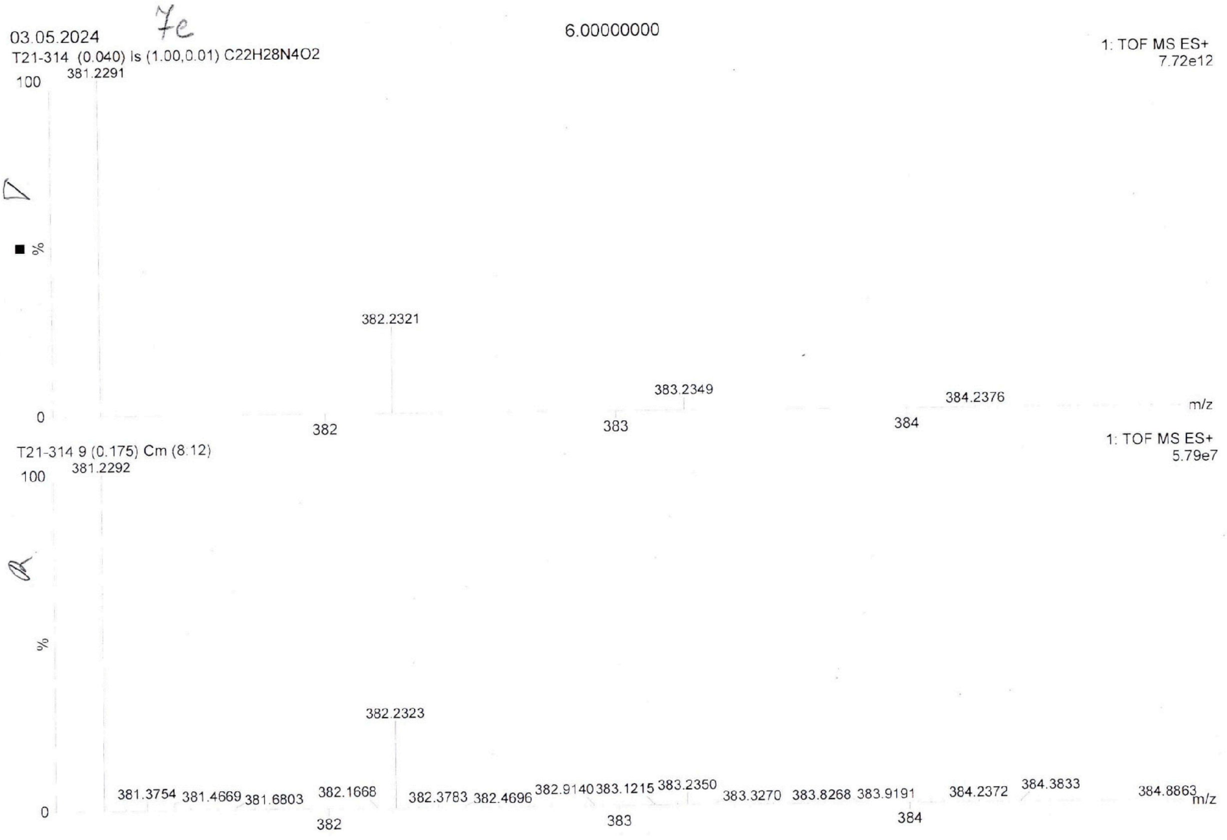

Figure S48.2. MS spectrum of compound **7e**

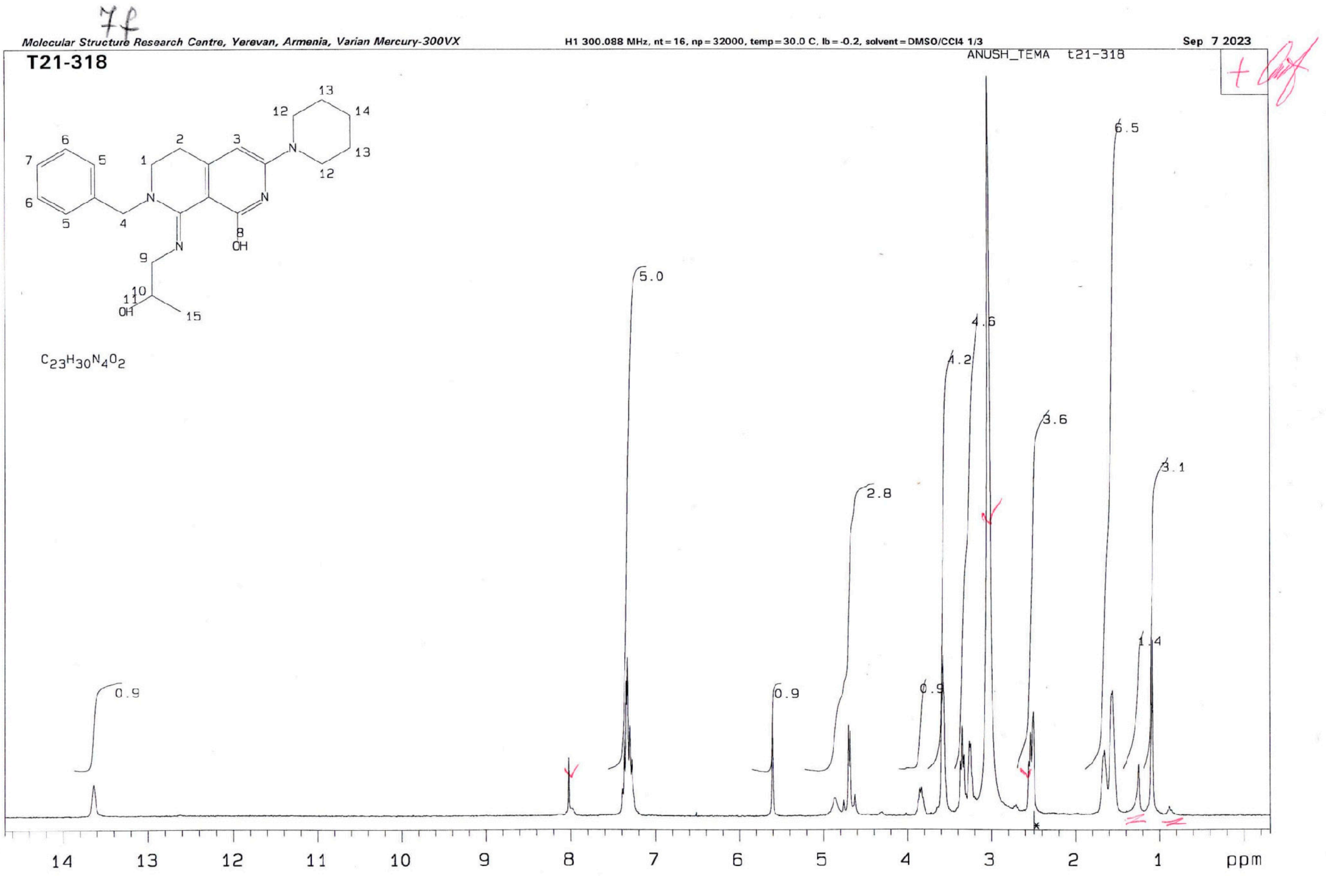

Figure S49. ^{1}H NMR spectrum of compound **7f**

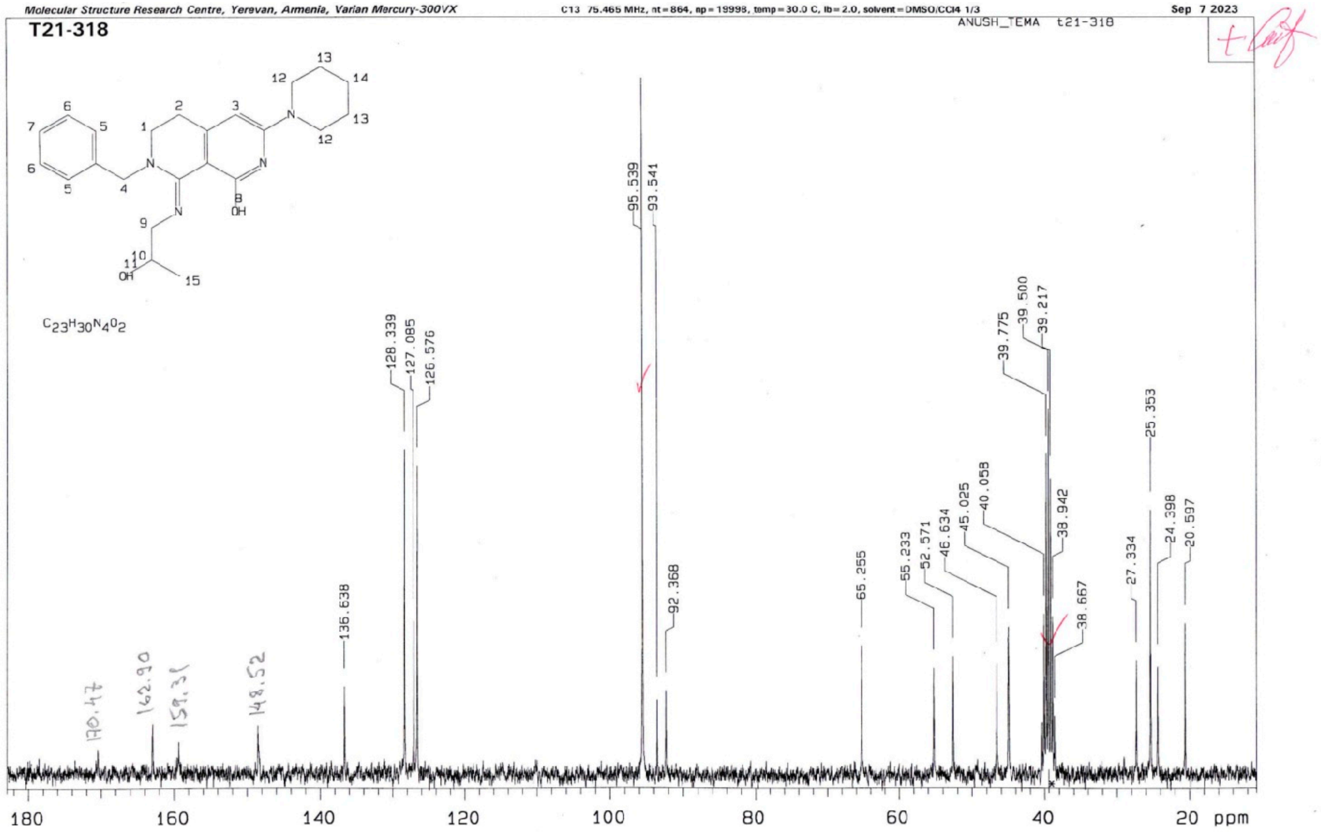

Figure S49.1. ^{13}C NMR spectrum of compound **7f**

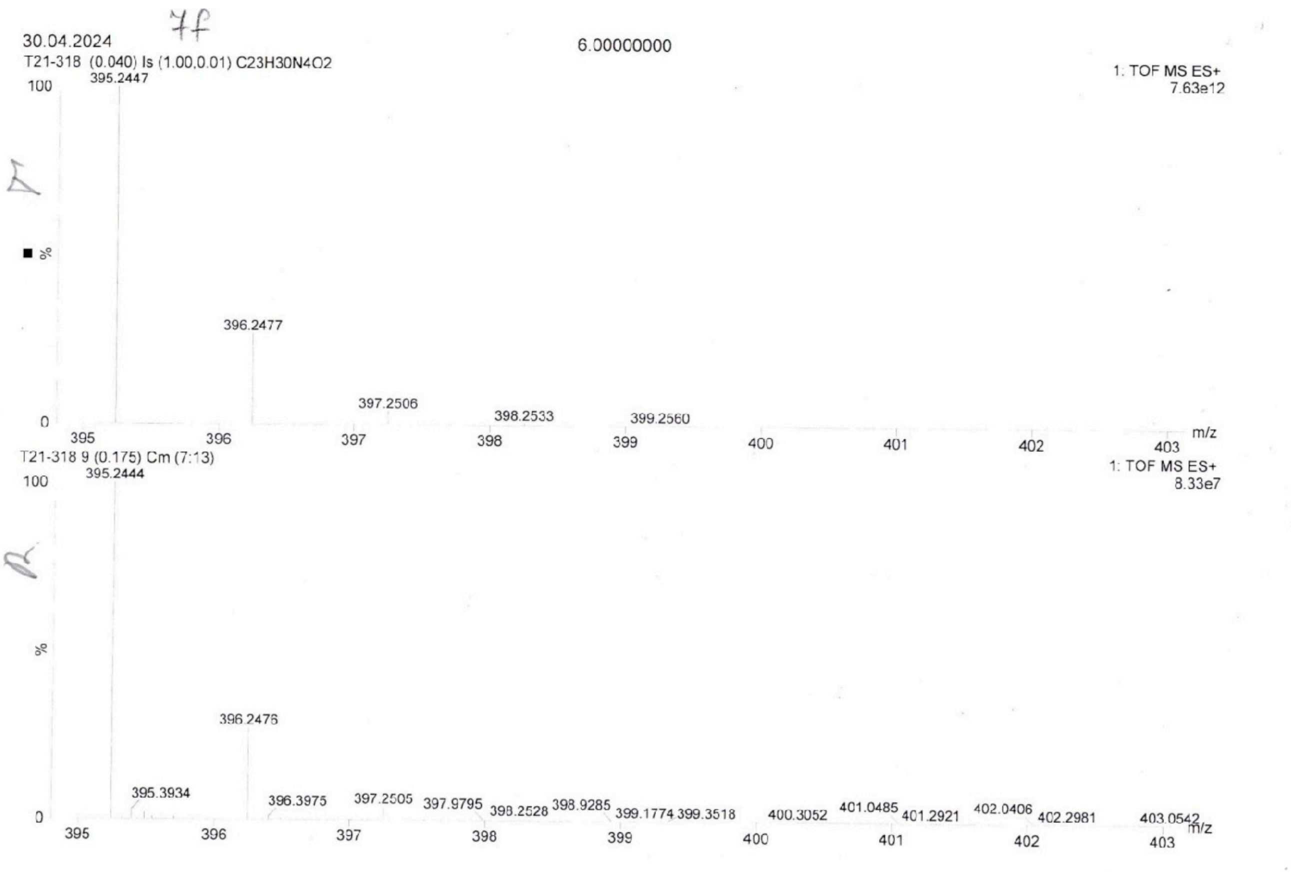

Figure S49.2. MS spectrum of compound **7f**

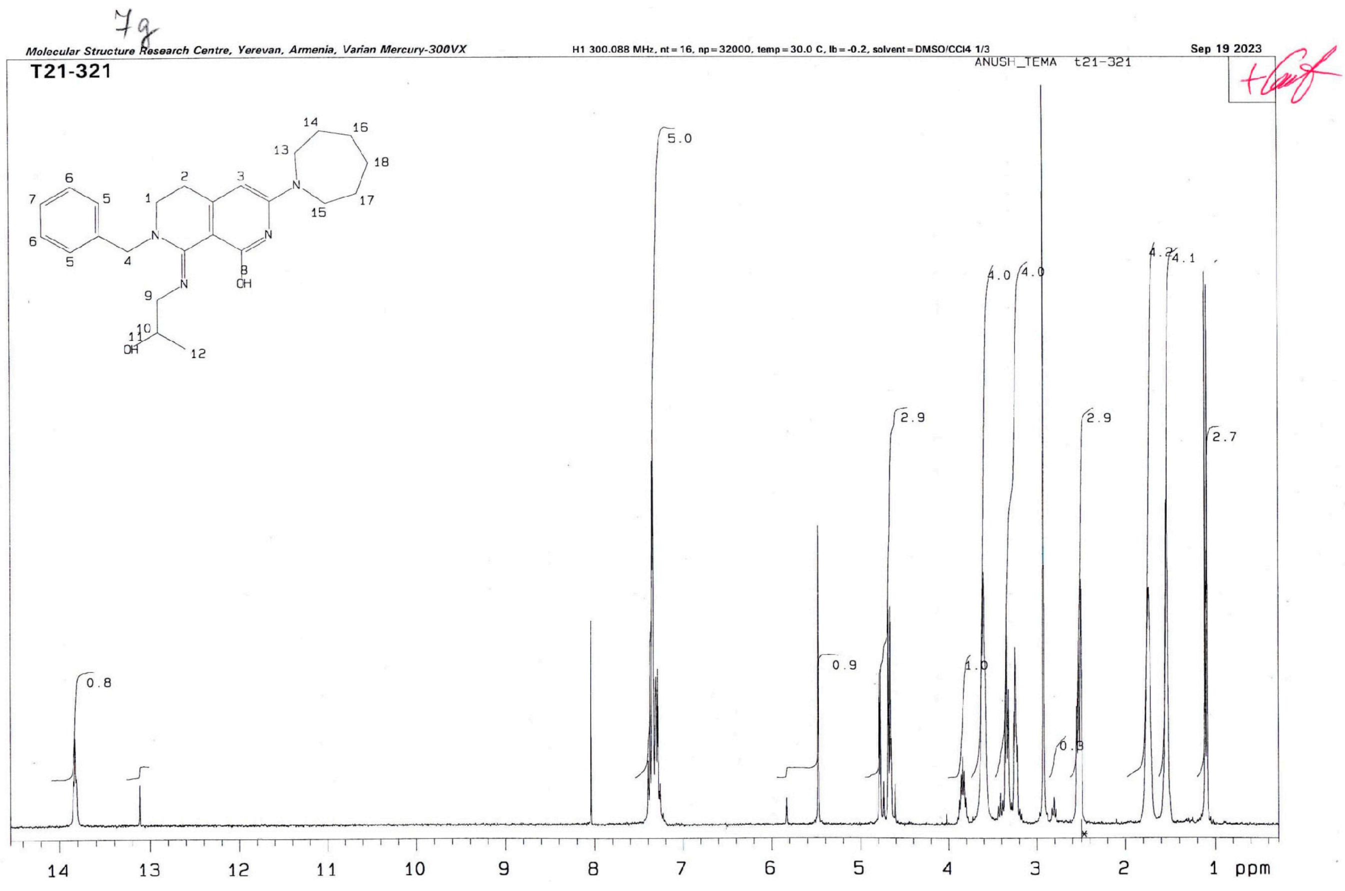

Figure S50. ^{1}H NMR spectrum of compound **7g**

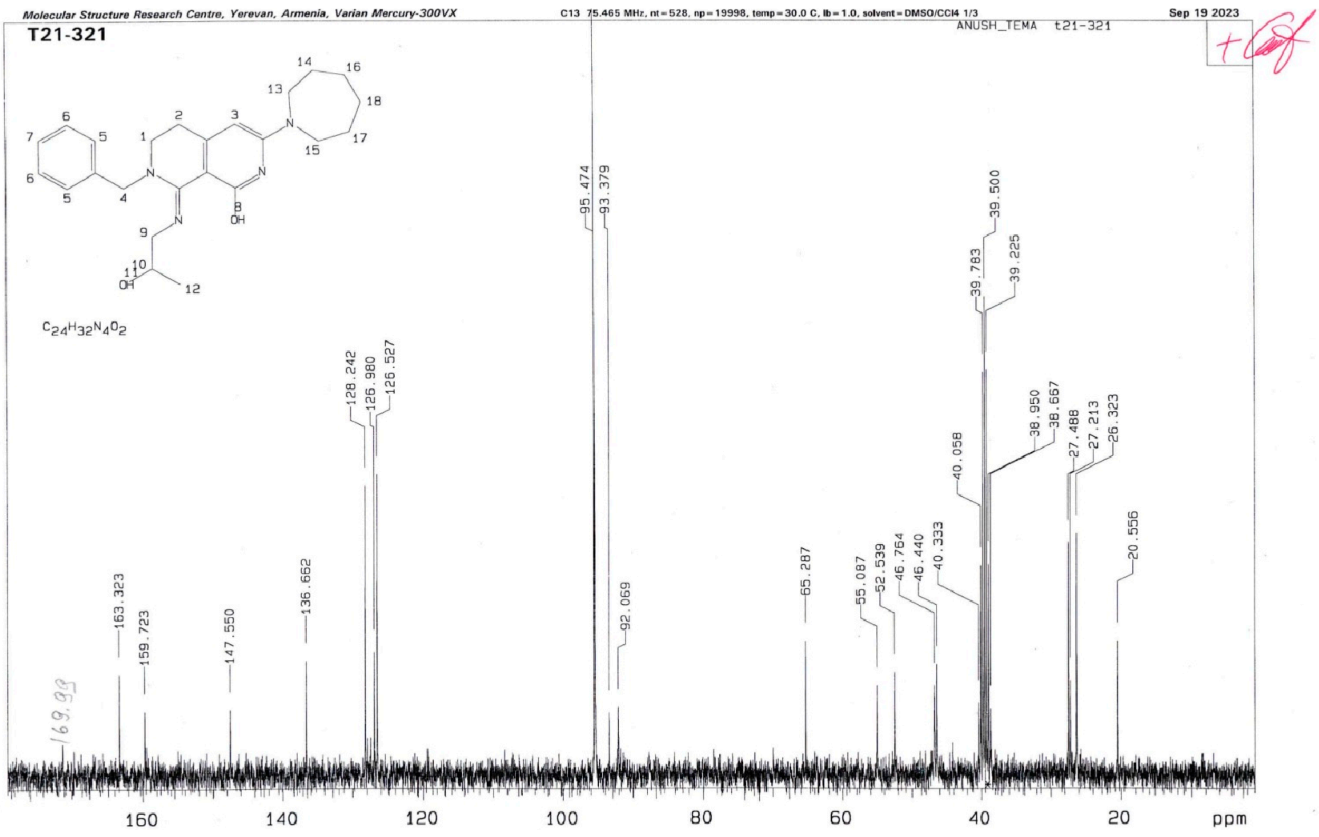

Figure S50.1. ^{13}C NMR spectrum of compound **7g**

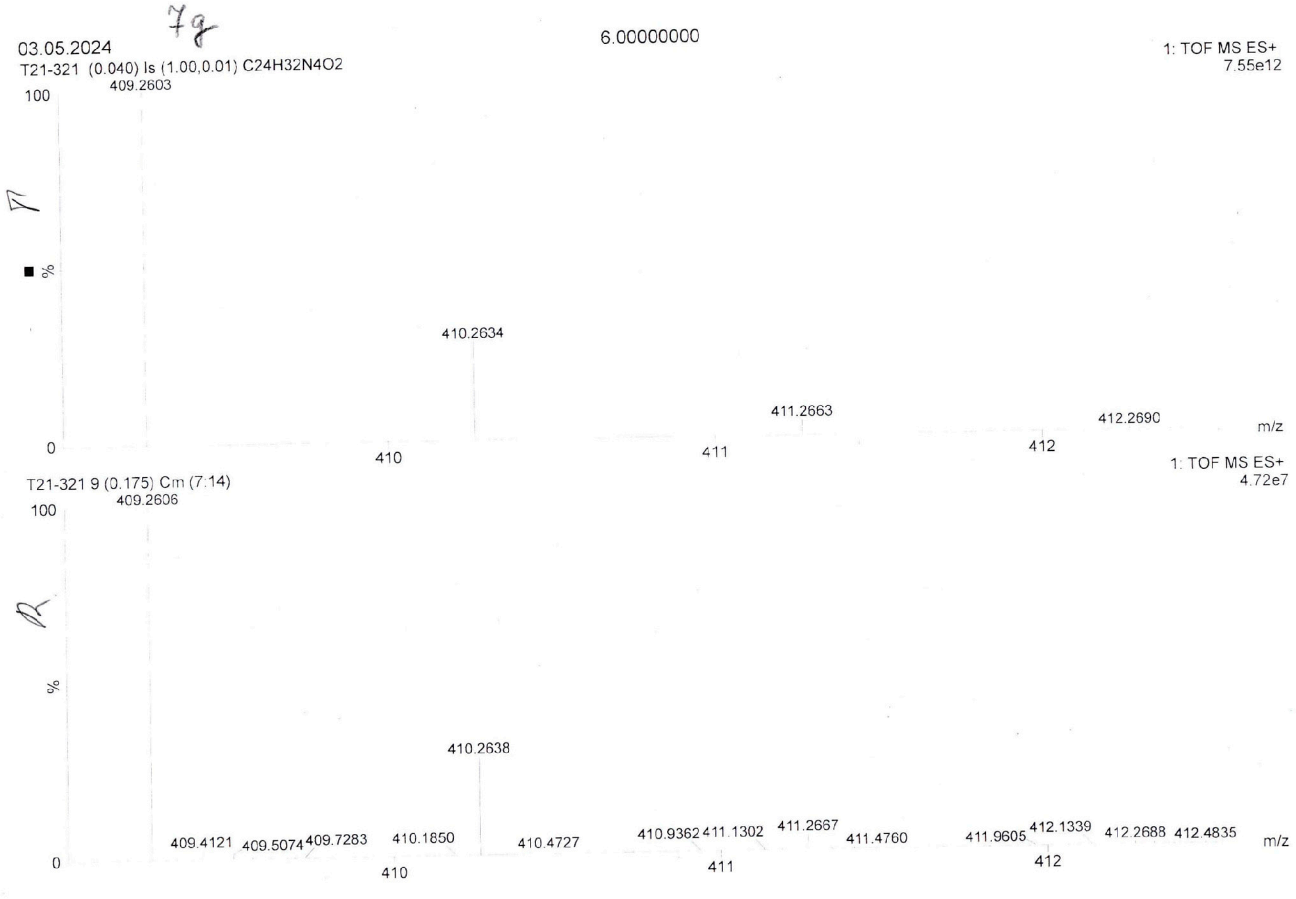

Figure S50.2. MS spectrum of compound **7g**

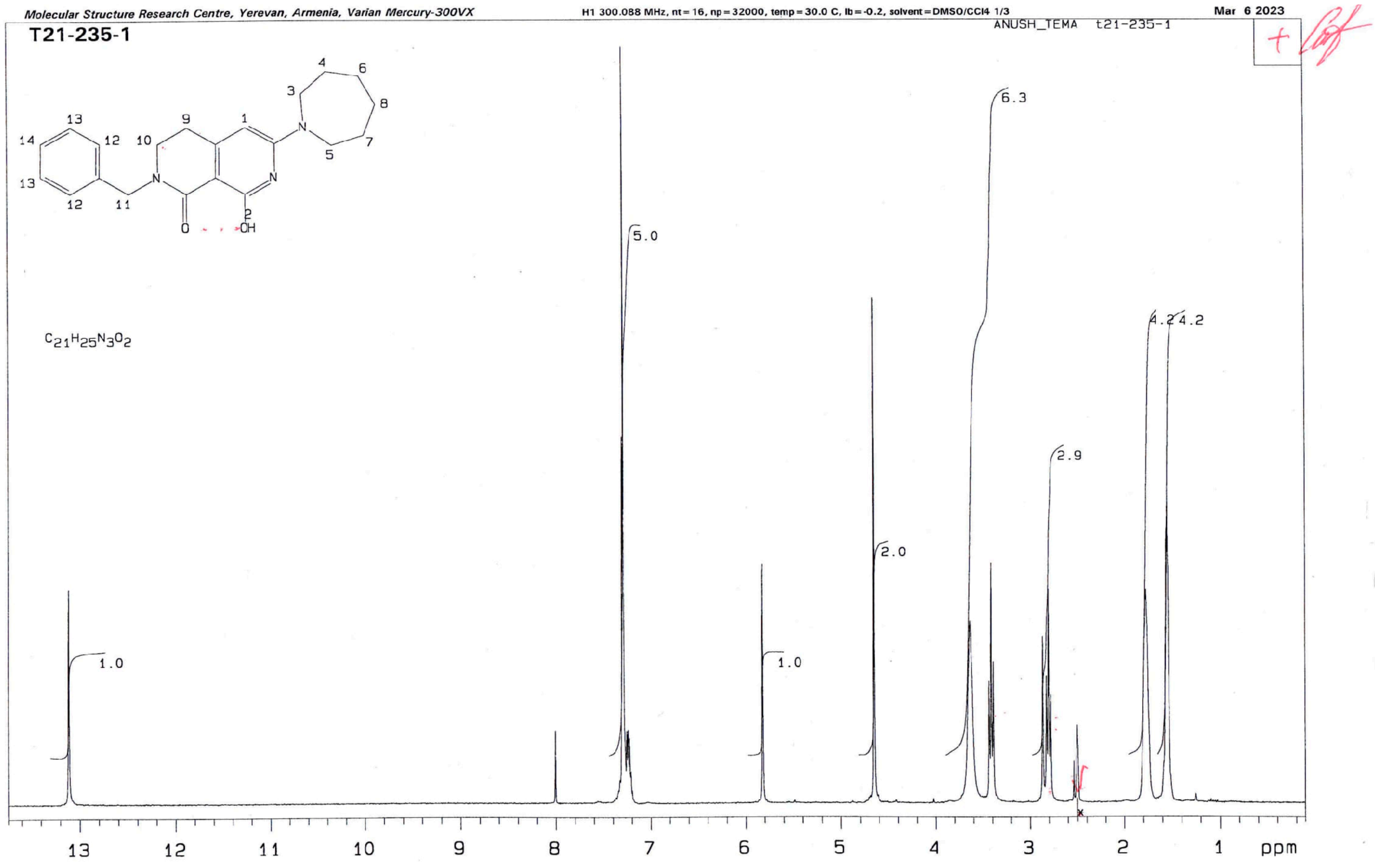

Figure S51. ^{1}H NMR spectrum of compound **8**

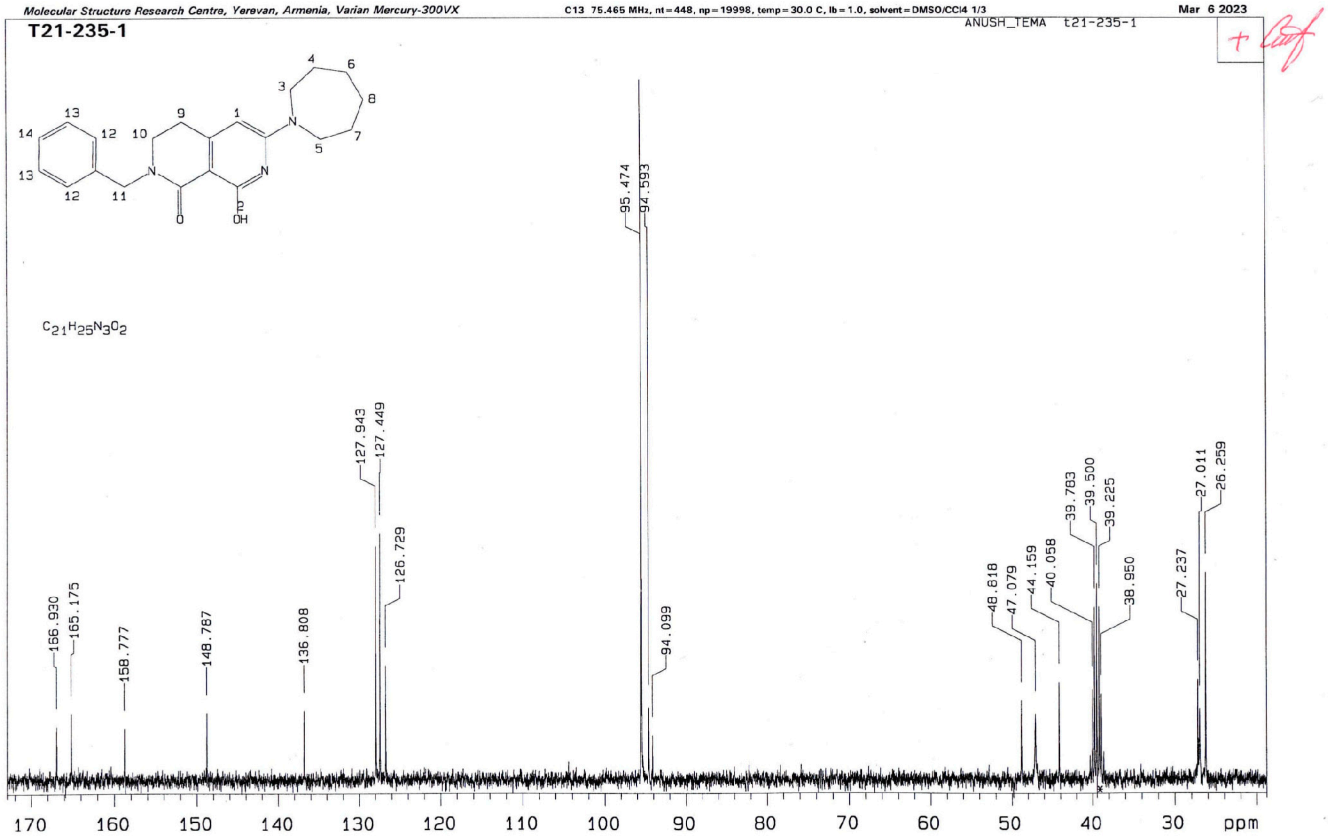

Figure S51.1. ^{13}C NMR spectrum of compound **8**

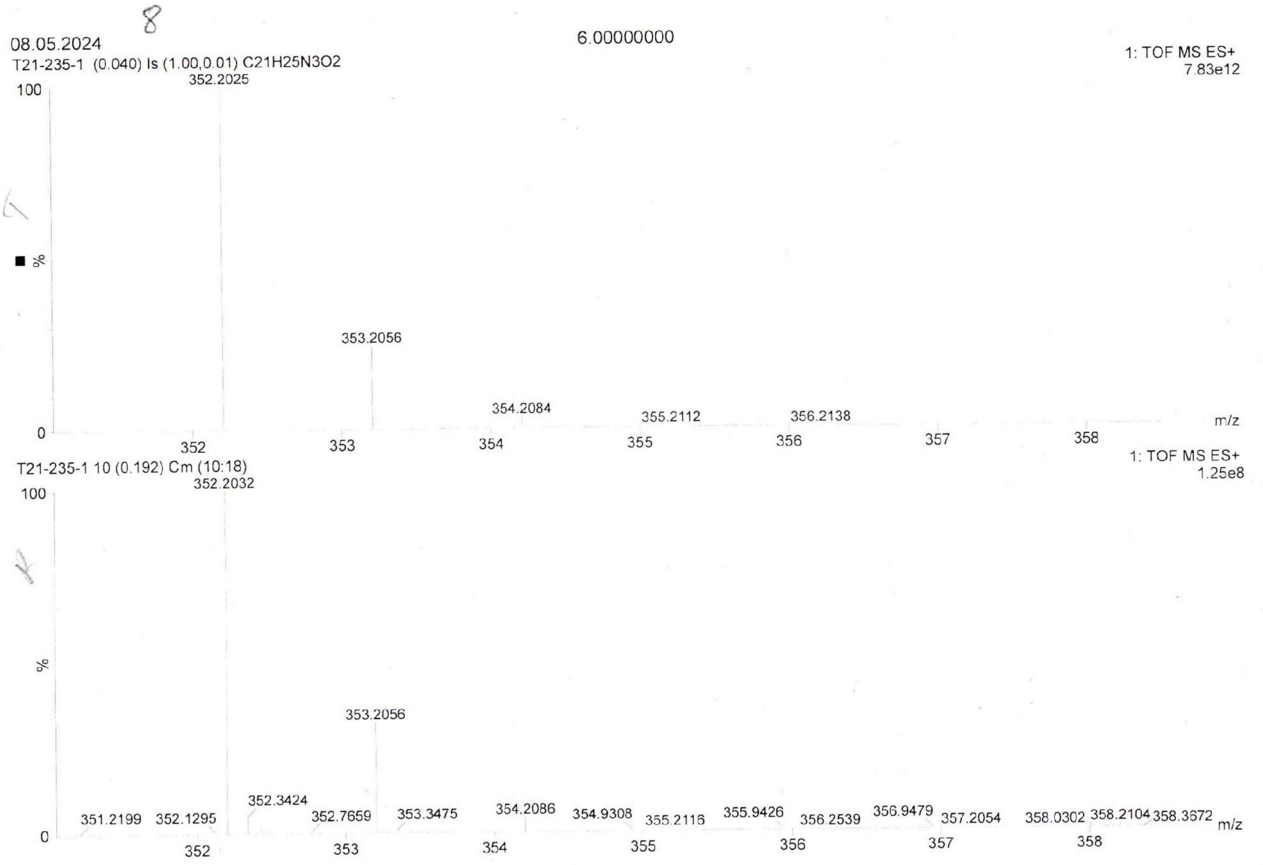

Figure S51.2. MS spectrum of compound **8**
